# Polyphenylglyoxamide-Based Amphiphilic Small Molecular Peptidomimetics as Antibacterial Agents with Anti-Biofilm Activity

**DOI:** 10.3390/ijms22147344

**Published:** 2021-07-08

**Authors:** Tsz Tin Yu, Rajesh Kuppusamy, Muhammad Yasir, Md. Musfizur Hassan, Manjulatha Sara, Junming Ho, Mark D. P. Willcox, David StC. Black, Naresh Kumar

**Affiliations:** 1 School of Chemistry, The University of New South Wales, Sydney, NSW 2052, Australia; tsztin.yu@unsw.edu.au (T.T.Y.); r.kuppusamy@unsw.edu.au (R.K.); md.m.hassan@unsw.edu.au (M.M.H.); junming.ho@unsw.edu.au (J.H.); 2 School of Optometry and Vision Science, University of New South Wales, Sydney, NSW 2052, Australia; m.yasir@unsw.edu.au (M.Y.); manjulatha.sara@unsw.edu.au (M.S.); m.willcox@unsw.edu.au (M.D.P.W.)

**Keywords:** antimicrobial peptide, peptidomimetics, terphenylglyoxamide, terphenyl, isatin, antibiofilm, membrane disruption

## Abstract

The rapid emergence of drug-resistant bacteria is a major global health concern. Antimicrobial peptides (AMPs) and peptidomimetics have arisen as a new class of antibacterial agents in recent years in an attempt to overcome antibiotic resistance. A library of phenylglyoxamide-based small molecular peptidomimetics was synthesised by incorporating an *N*-alkylsulfonyl hydrophobic group with varying alkyl chain lengths and a hydrophilic cationic group into a glyoxamide core appended to phenyl ring systems. The quaternary ammonium iodide salts **16d** and **17c** showed excellent minimum inhibitory concentration (MIC) of 4 and 8 μM (2.9 and 5.6 μg/mL) against *Staphylococcus aureus*, respectively, while the guanidinium hydrochloride salt **34a** showed an MIC of 16 μM (8.5 μg/mL) against *Escherichia coli.* Additionally, the quaternary ammonium iodide salt **17c** inhibited 70% *S. aureus* biofilm formation at 16 μM. It also disrupted 44% of pre-established *S. aureus* biofilms at 32 μM and 28% of pre-established *E. coli* biofilms 64 μM, respectively. A cytoplasmic membrane permeability study indicated that the synthesised peptidomimetics acted via disruption and depolarisation of membranes. Moreover, the quaternary ammonium iodide salts **16d** and **17c** were non-toxic against human cells at their therapeutic dosages against *S. aureus*.

## 1. Introduction

Infections caused by multidrug-resistant bacteria pose a serious threat to humans and are a major health concern [1]. This multi-drug resistance arises from the selective survival pressure exerted by the mechanism of action of conventional antibiotics [2]. Bacteria resist antibiotics through various mechanisms, such as enzymatic degradation of antibiotics, reducing the permeability of the bacterial cell membrane, removal of antibiotics via efflux, and mutation or enzymatic alteration of drug binding sites [3,4,5].

Another survival strategy displayed by bacteria is the formation of biofilms. Biofilms are communities of bacteria encased in an extracellular polymeric matrix adhering to a surface [6,7,8]. Biofilm can form on both biotic surfaces, such as on the tooth and lung, as well as abiotic surfaces, such as catheters and shunts. Due to the protective barrier of the biofilm and the lower growth rate of bacteria inside biofilms, bacteria in biofilms are more resistant to antibiotics [6,8,9,10]. Bacteria in biofilms can be 10 to 1000 times more resistant to antibiotic treatment than in their planktonic form [10]. Biofilms are accountable for approximately 80% of chronic and recurrent bacterial infections [8,11,12]. As biofilms further complicate the treatment of bacterial infections, the ideal novel antibiotic should not only target planktonic bacteria, but also have the ability to disrupt bacterial biofilms.

In response to the rapid emergence of multidrug-resistant bacteria and the formation of bacterial biofilms, attention has been drawn to the development of antimicrobial peptides (AMPs) as a novel class of antibiotics [13,14]. AMPs are a diverse group of natural bioactive peptides found in living organisms [15,16,17] and they play a significant role in the human defence system against microbial infection [18,19]. They act against bacteria by predominantly disrupting the bacterial cell membrane [18,20]. Examples of AMPs include pexiganan (MSI-78), which has reached phase III clinical trials for the topical treatment of infected diabetic foot ulcers, while cathelicidin (LL-37) is in phase IIb clinical trials for the treatment of chronic leg ulcers [13,21]. As AMPs disrupt bacterial cell membranes via non-receptor interactions, the emergence of antibacterial resistance against AMPs is less likely than for conventional antibiotics [17,22,23]. Other than disrupting bacterial cell membranes, some AMPs are known to kill bacteria by inhibiting DNA, RNA and protein synthesis of bacteria [18]. While AMPs are active against bacteria, their high manufacturing cost from solid-phase peptide synthesis and poor pharmacokinetics, such as low bioavailability and high susceptibility to proteolytic degradation, have limited their use as antibacterial agents [18,21,24].

To address the limitations of AMPs, peptidomimetics have been developed [25,26]. These peptidomimetics include α-peptides [27,28], β-peptides [29,30,31], peptoids [28,32,33,34], *N*-acylated *N*-aminoethylpeptides [35,36,37] and oligoacyllysines [38,39]. Alternatively, efforts have also been made to develop small molecular antimicrobial peptidomimetics, such as arylamide foldamers [40,41,42], phenyleneethynylenes [43], anthranilamides [44], binaphthyls [45] and sofalcones [46]. Like natural AMPs, peptidomimetics also possess an amphiphilic structure with polar and non-polar groups. The cationic groups first interact with anionic lipids on the bacterial cell membrane, followed by insertion into the bacterial cell membrane with the aid of hydrophobic groups. This leads to the disruption and formation of pores on the bacterial cell membrane, causing cell death [18,47,48,49].

**Figure 1:**
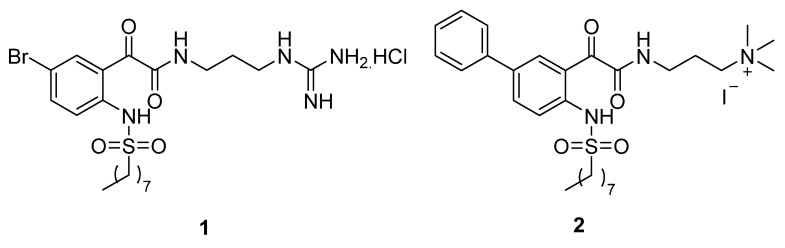
Structures of phenylglyoxamide- and biphenylglyoxamide-based small molecular antimicrobial peptidomimetics **1** and **2**.

In our previous studies, we reported phenylglyoxamide- and biphenylglyoxamide-based small molecular antimicrobial peptidomimetics **1** and **2** (Figure 1) with high antibacterial activity against *Staphylococcus aureus*, with minimum inhibitory concentration (MIC) values of 12 and 16 μM, respectively [50,51]. Moreover, peptidomimetic **2** also showed a good MIC value of 32 μM against *Escherichia coli*. The hydrophobic octanesulfonyl group was found to be important for antibacterial activity, as potency was lost when the chain was shortened.

Terphenyl derivatives possess antibacterial activities [52,53,54,55]; however, the effects of introducing a terphenyl moiety in place of the biphenyl moiety of the biphenylglyoxamide scaffold have not been explored. Additionally, lengthening the alkyl chain of the sulfonyl group has not been investigated either. In this study, we report the synthesis of new series of mono-, bi- and terphenylglyoxamide-based small molecular peptidomimetics with alkylsulfonyl groups of different alkyl chain lengths. The antibacterial activities of these synthesised peptidomimetics were evaluated against *S. aureus*, *E. coli* and *Pseudomonas aeruginosa*. The ability of the peptidomimetics to disrupt pre-established bacterial biofilms was also evaluated. The possible mechanism of action of the peptidomimetics was investigated by a membrane depolarisation assay, while their in vitro toxicity against human cells was also assessed to determine their specificity.

## 2. Results and Discussion

### 2.1. Synthesis of Polyphenylglyoxamide-Based Antimicrobial Peptidomimetics

**Scheme 1:**
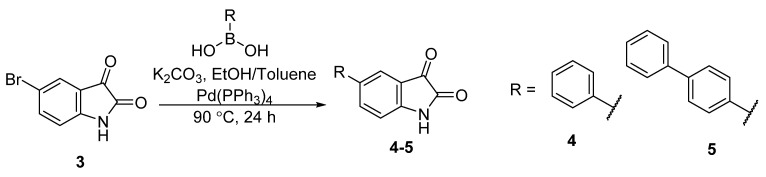
Synthesis of 5-phenylisatin **4** and 5-(biphenyl-4-yl)isatin **5**.

To incorporate the biphenyl or terphenyl group into the phenylglyoxamide backbone, 5-bromoisatin **3** was reacted with phenylboronic acid or 4-biphenylboronic acid with a catalytic amount of tetrakis(triphenylphosphine)palladium(0) via Suzuki–Miyaura cross-coupling reaction to give 5-phenylisatin **4** and 5-(biphenyl-4-yl)isatin **5** in good yields of 86% and 78%, respectively (Scheme 1) [51,56].

**Scheme 2:**
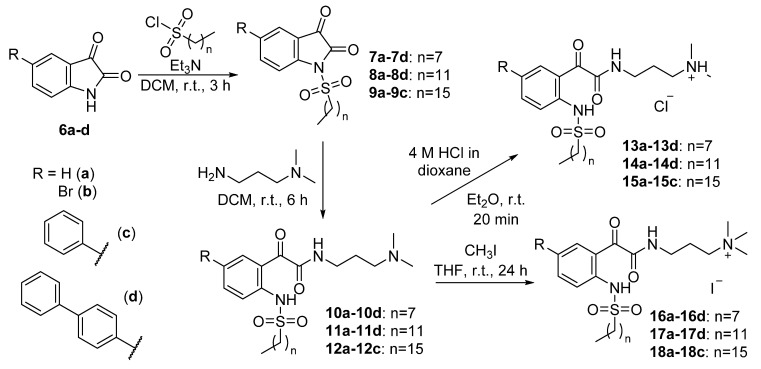
Synthesis of glyoxamides **10**–**12**, tertiary ammonium chloride salts **13**–**15** and quaternary ammonium iodide salts **16**–**18**.

Alkylsulfonyl groups with different chain lengths were then installed into the isatin scaffold by following a previously reported procedure [50,51] to provide the hydrophobic group for the target peptidomimetics. Isatins **6a**–**6d** were treated with triethylamine and various alkylsulfonyl chlorides in dichloromethane (DCM) at room temperature to afford *N*-sulfonylisatins **7a**–**7d** (*n* = 7), **8a**–**8d** (*n* = 11) and **9a**–**9c** (*n* = 15) in 28–60% yield (Scheme 2). However, the reaction between 5-(biphenyl-4-yl)isatin **6d** and 1-hexadecanesulfonyl chloride gave no product. It was initially suspected that the unsuccessful synthesis was due to the poor solubility of 5-(biphenyl-4-yl)isatin **6d** and 1-hexadecanesulfonyl chloride in DCM; however, no reaction was observed, even when the sulfonylation reaction was then attempted in other solvents, such as tetrahydrofuran (THF), dimethylformamide (DMF) and toluene. Further attempts involved heating the reaction mixture at reflux in toluene and using sodium hydride or *N*,*N*-diisopropylethylamine (DIPEA) as the base, but none of the attempted conditions were successful in the synthesis of the desired product.

The synthesised *N*-sulfonylisatins **7**–**9** were then ring-opened with 3-dimethylaminopropylamine in DCM to afford the corresponding glyoxamides **10**–**12** in excellent yields of 96–99%, except glyoxamides **10d** and **11d** which gave moderate yields of 61% and 53%, respectively. The lower yield of the latter compounds could be due to the low solubility of the starting material in DCM. The cationicity of the target molecule was then introduced by treating the glyoxamides **10**–**12** with 4 M HCl in dioxane to afford the corresponding tertiary ammonium chloride salts **13**–**15** in a moderate to good yield of 60–99%. Alternatively, a different cationic group can be installed by treating the glyoxamides **10**–**12** with iodomethane to afford the corresponding quaternary ammonium iodide salts **13**–**15** in yields of 42–91%.

**Scheme 3:**
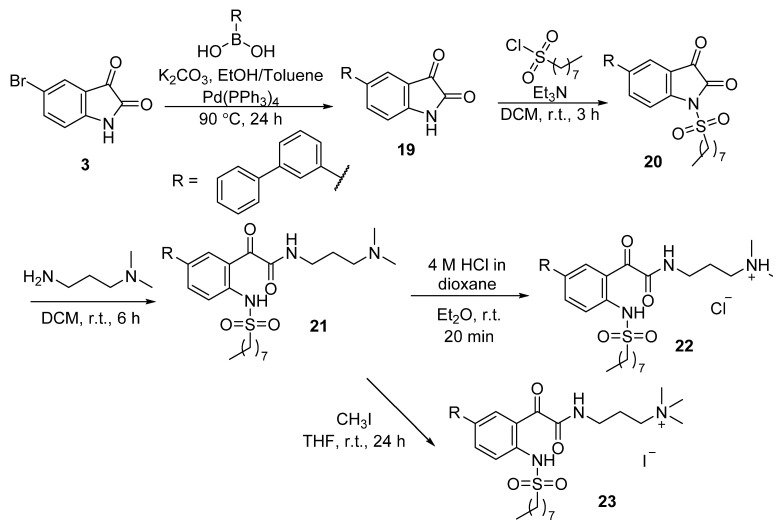
Synthesis of glyoxamide **21**, tertiary ammonium chloride salt **22** and quaternary ammonium iodide salt **23** bearing a meta-terphenyl system.

To explore whether changing the position of the terminal phenyl ring in the terphenyl system has any effect on antibacterial activity, ammonium salts **22**–**23** bearing a meta-terphenyl scaffold instead of a para-terphenyl scaffold were also synthesised following the above-mentioned synthetic route, with 3-biphenylboronic acid as the starting material in the initial Suzuki–Miyaura cross-coupling reaction (Scheme 3).

**Scheme 4:**
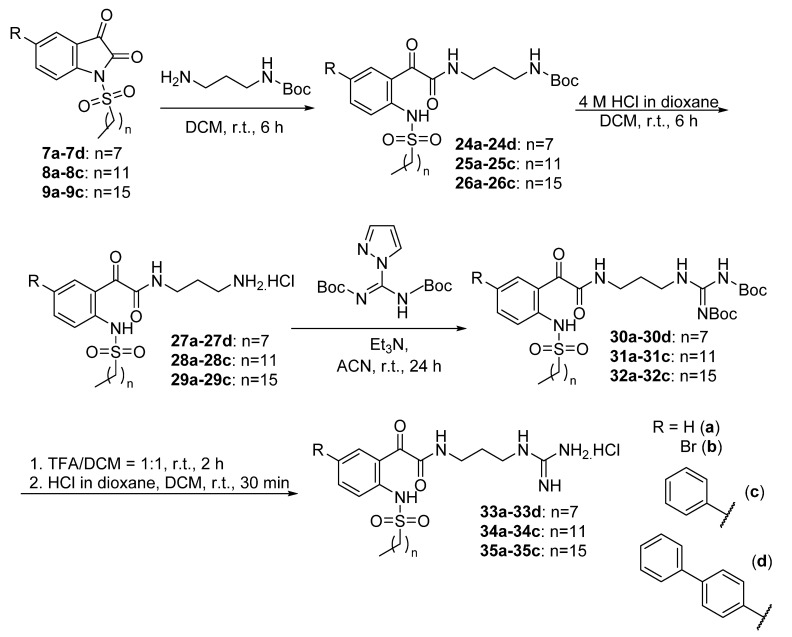
Synthesis of guanidinium hydrochloride salts **33**–**35**.

As previous studies have reported that the introduction of a guanidine as the cationic group could increase the potency of peptidomimetics against Gram-positive *S. aureus* [50,57], the guanidinium hydrochloride salts **33**–**35** were also synthesised (Scheme 4). To achieve this, *N*-sulfonylisatins **7**–**9** were ring-opened with *N*-Boc-1,3-propanediamine to afford the Boc-protected glyoxamides **24**–**26** in excellent yields of 88–98%. The Boc-protecting group was then cleaved by treatment with 4 M HCl in dioxane to give the corresponding aminoglyoxamides **27**–**29** in yields of 60–95%. The guanidine moiety was then introduced to the molecule by reacting the aminoglyoxamides **27**–**29** with *N*,*N*′-di-Boc-1*H*-pyrazole-1-carboxamidine in the presence of triethylamine in acetonitrile (ACN) to give the Boc-protected guanidine glyoxamides **30**–**32** in yields of 51–87%. Finally, the Boc-protecting group was cleaved with trifluoroacetic acid in DCM, followed by anion exchange reaction with 4 M HCl in dioxane to give the guanidinium hydrochloride salts **33**–**35** in 48–97% yield.

### 2.2. Antibacterial Activity

The synthesised peptidomimetics were tested in the minimal inhibitory concentration (MIC) assay for the determination of their antibacterial activities against Gram-positive *S. aureus* (SA38). In addition, selected peptidomimetics (**16d, 17b, 17c, 18a, 34a, 34b**), which showed high antibacterial activity against Gram-positive *S. aureus*, were also tested for their antibacterial activities against Gram-negative *E. coli* (K12) and *P. aeruginosa* (PAO1). In general, the tested peptidomimetics possess lower antibacterial activity against Gram-negative bacteria than Gram-positive *S. aureus*, especially *P. aeruginosa*, for which none of the tested peptidomimetics in this study showed any antibacterial activity at the highest concentration tested (250 μM). The MIC values of all tested peptidomimetics are shown in Table 1. There was no effect of the different growth media on MIC values, with the MIC of peptidomimetics **16d** and **17c** being 4 and 8 μM (2.9 and 5.6 μg/mL), respectively, in the trypticase soy broth (TSB), Mueller Hinton broth (MHB) or cationic adjusted MHB.

The incorporation of a biphenyl system in the glyoxamide scaffold enhanced the antibacterial activity of the peptidomimetics against both Gram-positive and Gram-negative bacteria [51]. To investigate the effect of lengthening the phenyl ring system on the antibacterial activity of the peptidomimetics, those bearing a meta- or para-terphenyl system were synthesised. Replacing the biphenyl system with a terphenyl system was generally beneficial for the antibacterial activity of the peptidomimetics against Gram-positive *S. aureus*. The previously reported quaternary ammonium iodide salt **16c** bearing a biphenyl system and an octanesulfonyl group had an MIC value of 16 μM (10 μg/mL) [51]. When an additional phenyl ring was incorporated, the corresponding quaternary ammonium iodide salts **16d** and **23** bearing a para-terphenyl or meta-terphenyl system, respectively, possessed MIC values of 4 and 8 μM (2.9 and 5.8 μg/mL) against *S. aureus*, suggesting that the terphenyl system was preferred over biphenyl. In terms of the position of the terminal phenyl ring in the terphenyl system, quaternary ammonium iodide salt **16d** (MIC = 4 μM or 2.9 μg/mL) bearing a para-terphenyl system was slightly more potent than the corresponding quaternary ammonium iodide salt **23** (MIC = 8 μM or 5.8 μg/mL) bearing a meta-terphenyl system. Against Gram-negative *E. coli* and *P. aeruginosa*, while quaternary ammonium iodide salt **16c** bearing a biphenyl system and an octanesulfonyl group possessed MIC values of 32 and 125 μM (21 and 80 μg/mL) against *E. coli* and *P. aeruginosa*, respectively, the antibacterial activity of the peptidomimetics was completely lost upon converting the biphenyl system to terphenyl as exhibited by the corresponding quaternary ammonium iodide salt **16d** (MIC > 250 μM or >180 μg/mL). This suggested that the extra phenyl ring in the terphenyl system created steric hindrance, which may reduce the antibacterial activity of the peptidomimetics against Gram-negative bacteria.

As shortening the octanesulfonyl chain was previously reported to be detrimental to the antibacterial activity of the peptidomimetics [50,51] and lengthening the octanesulfonyl chain was not previously studied, peptidomimetics with dodecanesulfonyl and hexadecanesulfonyl groups were synthesised to assess the effect of longer chain lengths on antibacterial activity. Interestingly, the effect of lengthening the alkylsulfonyl chain depended on the size of the phenyl ring system. For the monophenyl system, the previously reported unsubstituted parent (**16a**) and 5-bromosubstituted quaternary ammonium iodide salts (**16b**) bearing an octanesulfonyl chain had MICs of 250 and 63 μM (142 and 41 μg/mL) against *S. aureus*, respectively. When the octanesulfonyl chain was lengthened to a dodecanesulfonyl chain, the MIC of the corresponding quaternary ammonium iodide salts **17a** and **17b** decreased to 16 and 8 μM (10 and 5.6 μg/mL) against *S. aureus*, respectively, indicating 16- and 8-fold increases in potency. A similar trend was also observed for quaternary ammonium iodide salts bearing a biphenyl system, although the increase in potency was only two-fold when comparing the quaternary ammonium iodide salt **16c** (MIC = 16 μM or 10 μg/mL) bearing an octanesulfonyl chain with the corresponding dodecanesulfonyl compound **17c** (MIC = 8 μM or 5.6 μg/mL). However, against Gram-negative *E. coli*, lengthening the octanesulfonyl chain to dodecanesulfonyl chain gave no improvement in antibacterial activity, as both **16c** and **17c** shared the same MIC value of 32 μM (10 and 5.6 μg/mL, respectively). Moreover, for the peptidomimetics bearing a terphenyl system, lengthening the octanesulfonyl chain to dodecanesulfonyl chain significantly decreased antibacterial activity against *S. aureus*, as the quaternary ammonium iodide salt **17d** (MIC = 63 μM or 48 μg/mL) bearing a dodecanesulfonyl chain showed 16-fold lower potency than the corresponding octanesulfonyl compound **16d** (MIC = 4 μM or 2.9 μg/mL). Further lengthening the alkylsulfonyl chain from dodecanesulfonyl to hexadecanesulfonyl was detrimental to the antibacterial activity of the peptidomimetics against *S. aureus*, as significant increases in MIC values were observed regardless of the phenyl ring system. One exception was the quaternary ammonium iodide salt **18a** bearing an unsubstituted monophenyl system, where the lengthening of the alkylsulfonyl chain from dodecanesulfonyl (**17a** with MIC = 16 μM or 10 μg/mL) to hexadecanesulfonyl (**18a** with MIC = 8 μM or 5.4 μg/mL) showed a two-fold increase in antibacterial activity.

In terms of cationic group modification, previous studies have identified that guanidinium hydrochloride salts bearing an octanesulfonyl chain possesses superior antibacterial activity against *S. aureus* compared to their corresponding tertiary ammonium chloride salts, quaternary ammonium iodide salts or uncharged glyoxamide compounds [50,51]. However, this was not necessarily true in this study when the octanesulfonyl chain was lengthened to dodecansulfonyl and hexadecanesulfonyl. In fact, guanidinium hydrochloride salts bearing a dodecanesulfonyl or hexadecanesulfonyl chain showed lower antibacterial activity against *S. aureus* than their corresponding quaternary ammonium iodide salts. The only exception was the monophenyl guanidinium hydrochloride salt **34a** (MIC = 8 μM or 4.3 μg/mL) bearing a dodecanesulfonyl chain, which showed slightly higher potency against *S. aureus* than its corresponding quaternary ammonium iodide salt **17a** (MIC = 16 μM or 10 μg/mL). Against Gram-negative *E. coli*, a similar trend was observed, where the 5-bromosubstituted quaternary ammonium iodide salt **17b** with a dodecanesulfonyl chain showed good antibacterial activity (MIC = 32 μM or 22 μg/mL), while the corresponding guanidinium hydrochloride salt **34b** showed no antibacterial activity at the highest tested concentration (MIC > 250 μM or > 153 μg/mL).

Overall, the structure–activity relationship analysis for these peptidomimetics suggests that the optimal combination for Gram-positive antibacterial activity was a terphenyl system, an octanesulfonyl chain and a quaternary ammonium iodide cationic group. Compound **16d** with a combination of these was the most potent compound (MIC = 4 μM or 2.9 μg/mL) against *S. aureus* in this study. Interestingly, there appeared to be an inverse relationship between the size of the phenyl system and the length of the alkylsulfonyl chain in favour of high antibacterial activity. Specifically, peptidomimetics bearing a bulky terphenyl system preferred a shorter octanesulfonyl chain while peptidomimetics bearing a non-bulky monophenyl system preferred a longer hexadecanesulfonyl chain for high antibacterial activity. In terms of the terminal cationic group, compounds with the terphenyl system or a long dodecanesulfonyl chain favoured the quaternary ammonium iodide salts for antibacterial activity against *S. aureus*, while octanesulfonyl compounds preferred the guanidinium hydrochloride salts.

### 2.3. Lipophilicity and Antibacterial Activity Correlation

The apparent inverse relationship between the size of the phenyl system and the length of the alkylsulfonyl chain for strong antibacterial activity suggested that the lipophilicity of the peptidomimetics might be an important factor driving the potency of the compounds. Hence, the logP values of all synthesised peptidomimetics were determined computationally.

**Figure 2:**
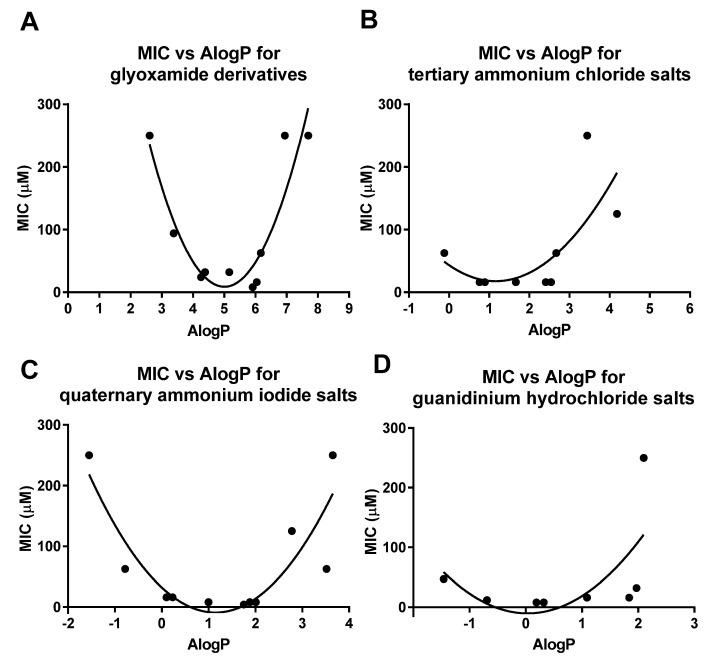
MIC vs. AlogP plot for (**A**) glyoxamide derivatives; (**B**) tertiary ammonium chloride salts; (**C**) quaternary ammonium iodide salts; and (**D**) guanidinium hydrochloride salts.

While there are different computational methods and approaches used to estimate logP values, the AlogP method under the empirical fragment-based approaches was used in this study, as empirical fragment-based approaches are highly efficient and the AlogP method was previously demonstrated to be one of the best performing methods for estimating logP (with mean absolute errors of 0.3 log unit or less), compared to other physics-based implicit and atomistic methods [58,59]. The AlogP method was developed by Ghose and Crippen in 1986 and was trained on nearly 900 molecular structures [60,61,62]. In this study, the AlogP values of all peptidomimetics were calculated, using MarvinSketch (Table 1) [63]. The MIC against *S. aureus* vs. AlogP plots for the glyoxamide derivatives, ammonium chloride salts, quaternary ammonium iodide salts and guanidinium hydrochloride salts are exhibited in Figure 2.

The plots revealed that there is an optimum range of AlogP values for each series of peptidomimetics for high antibacterial activity. Peptidomimetics with AlogP value above or below the optimum range generally show lower antibacterial activity. For example, glyoxamide derivatives with AlogP values between 4.3 and 6.0 possessed high antibacterial activity with MIC values at or below 32 μM, while those outside of the optimum AlogP range exhibited MIC values at or above 63 μM. Similar trends were also observed for tertiary ammonium chloride salts, quaternary ammonium iodide salts and guanidinium hydrochloride salts, which exhibited optimal AlogP ranges of 0.8–2.5, 0.1–2.0 and −0.7–1.8, respectively. The elucidation of the optimal AlogP range for each series of peptidomimetics could be useful in the prediction of the antibacterial activity in the future development of glyoxamide-based antimicrobial peptidomimetics.

### 2.4. Biofilm Disruption

**Figure 3:**
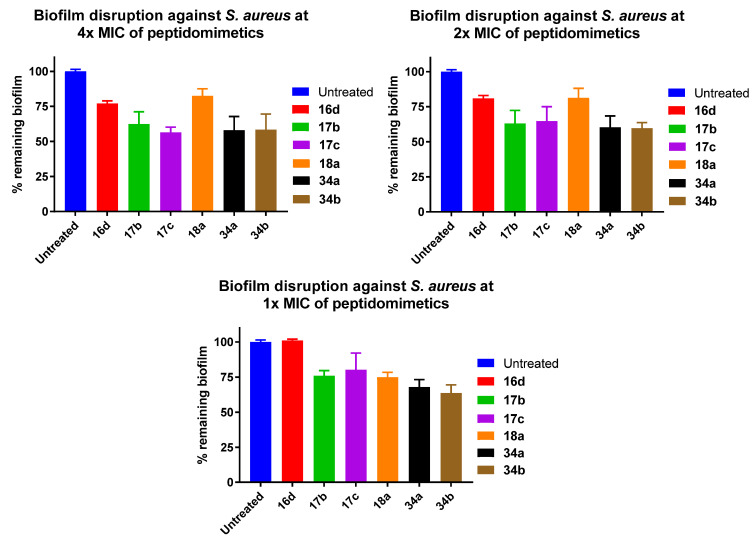
Percentage remaining *S. aureus* biofilms following 24 h treatment with peptidomimetics at 4× (**top left**), 2× (**top right**) and 1× (**bottom**) MIC. Error bars represent the standard error of at least triplicates (*n* ≥ 3).

As bacterial biofilms are one of the major challenges in treating bacterial infection, the ability of the synthesised peptidomimetics to disrupt pre-established biofilms was investigated. In this study, selected potent peptidomimetics (**16d**, **17b**, **17c**, **18a**, **34a**, **34b**) were tested against pre-established Gram-positive *S. aureus* biofilms at 1×, 2× and 4× MIC of the peptidomimetics, using the crystal violet assay (Figure 3). The ability of the tested peptidomimetics to disrupt pre-established biofilms generally increased with the concentration.

In general, guanidinium hydrochloride salts (**34a**, **34b**) were more potent *S. aureus* biofilm disruptors than quaternary ammonium iodide salts (**16d**, **17b**, **17c**, **18a**), as they were able to disrupt a higher amount of pre-established *S. aureus* biofilms at all tested concentrations. The only exception was quaternary ammonium iodide salt **17c**, which displayed a slightly higher biofilm disruption at 4× MIC. At lower concentration (1× MIC), most peptidomimetics (**17b**, **17c**, **18a**, **34a**, **34b**) showed 20–36% *S. aureus* biofilm disruption, except for quaternary ammonium iodide salt **16d**, bearing a terphenyl scaffold and octanesulfonyl group, which showed no antibiofilm activity at 1× MIC. Amongst the active peptidomimetics, the guanidinium hydrochloride salt **34b** bearing a dodecanesulfonyl group was the most potent compound, disrupting 36% of pre-established *S. aureus* biofilms at 1× MIC. It was also one of the most active compounds at the highest tested concentration (4× MIC), possessing the same biofilm disruption ability as guanidinium hydrochloride salt **34b**, disrupting 42% of pre-established *S. aureus* biofilms, slightly lower than quaternary ammonium iodide salt **17c**, which managed to disrupt 44% of pre-established *S. aureus* biofilms. This was followed by quaternary ammonium iodide salt **17b**, which disrupted 38% of pre-established *S. aureus* biofilms at 4× MIC. These compounds possessed similar antibiofilm activity as LL-37, which disrupted about 40% pre-established *S. aureus* biofilms at 4× MIC [64].

**Figure 4:**
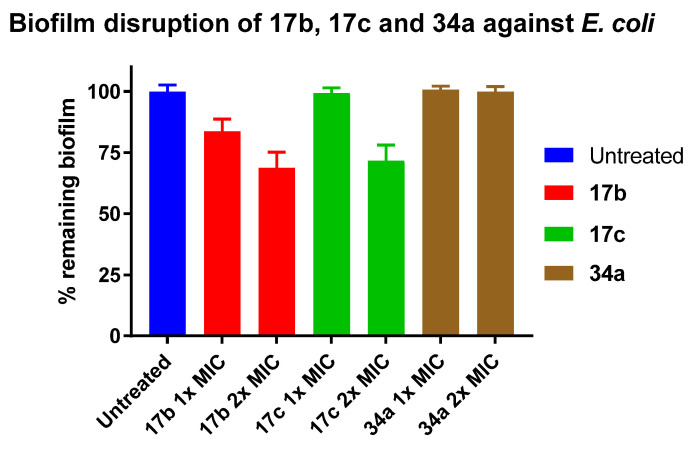
Percentage remaining *E. coli* biofilms following 24 h treatment with peptidomimetics **17b**, **17c** and **34a** at 1× and 2× MIC. Error bars represent the standard error of triplicates (*n* = 3).

The biofilm disruption activities of the synthesised peptidomimetics **17b**, **17c**, and **34a**, which possessed good antibacterial activity against *E. coli* in the MIC assay, were also investigated at 1× and 2× MIC against pre-established *E. coli* biofilms. All tested compounds showed lower biofilm disruption ability against Gram-negative *E. coli* than against Gram-positive *S. aureus*. Of the three tested compounds, the quaternary ammonium iodide salts **17b** and **17c** were able to disrupt 31% and 28% of the pre-established *E. coli* biofilms at 2× MIC (Figure 4). Although the guanidinium hydrochloride salt **34a** was the most potent compound at inhibiting *E. coli* growth in the MIC assay and was one of the best *S. aureus* biofilm disruptors, it was unable to disrupt any pre-established *E. coli* biofilms. This suggests that it can only target *E. coli* cells in their planktonic form, but not those that are embedded in the polymeric matrices of biofilms. The observed discrepancy in the antibiofilm activity of these peptidomimetics against Gram-positive *S. aureus* and Gram-negative *E. coli* could be due to the differences in the composition of their biofilms [65,66,67].

### 2.5. Biofilm Inhibition

**Figure 5:**
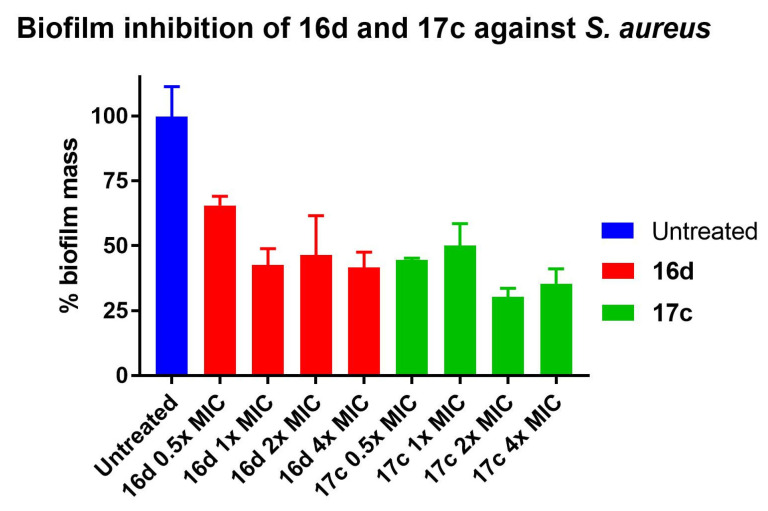
Inhibition of biofilm fomration of *S. aureus* by peptidomimetics **16d** and **17c** at 0.5×, 1×, 2× and 4× MIC. Error bars represent the standard error of triplicates (*n* = 3).

In addition to biofilm disruption assay, selected potent peptidomimetics **16d** and **17c** were also tested for their ability to inhibit the formation of *S. aureus* biofilm at 0.5×, 1×, 2× and 4× MIC of the peptidomimetics (Figure 5). In this assay, *S. aureus* was incubated in the presence of different concentrations of the tested peptidomimetics under a static condition. After overnight incubation, planktonic bacteria were removed, and the remaining biofilms were quantified, using crystal violet staining.

Both peptidomimetics **16d** and **17c** were effective in inhibiting *S. aureus* biofilm formation, as they were able to inhibit more than 50% of biofilm formation (except for peptidomimetics **16d** at 0.5× of its MIC), with **17c** showing a slightly higher inhibition than **16d**, except at 1× MIC. Of the two peptidomimetics, maximum inhibition of biofilm formation was observed for peptidomimetic **17c** at 2× and 4× of its MIC, inhibiting 70% and 65% of biofilm formation, respectively. At sub-MIC (0.5× MIC), **16d** and **17c** were able to inhibit 35% and 56% of biofilm formation, respectively.

### 2.6. Cytoplasmic Membrane Permeability

The disruption effect of selected potent peptidomimetics (**16d**, **17c**, **34a**) on bacterial cytoplasmic membrane was investigated in a membrane dye release assay, using the membrane potential-sensitive dye 3,3′-dipropylthiadicarbocyanine iodide (diSC3-5). This dye readily partitions into and aggregates in bacterial cell membranes, quenching its own fluorescence. However, structural damage to the bacterial cell membrane causes a loss in the membrane potential, which results in the leakage of dye from the bacterial cell membrane into the medium and an increase in fluorescence intensity. By detecting changes in fluorescence intensity, the integrity of the bacterial cytoplasmic membrane can be monitored.

It was hypothesised that the synthesised peptidomimetics possess similar mechanisms of action as natural AMPs, which act via the electrostatic interactions between the negatively charged bacterial cell membrane and the cationic group of the peptidomimetics. As this disrupts the bacterial membrane and/or forms pores on the membrane, causing an alteration of the membrane potential, an increase in fluorescence intensity was expected upon the treatment of bacterial cells with the test compounds.

**Figure 6:**
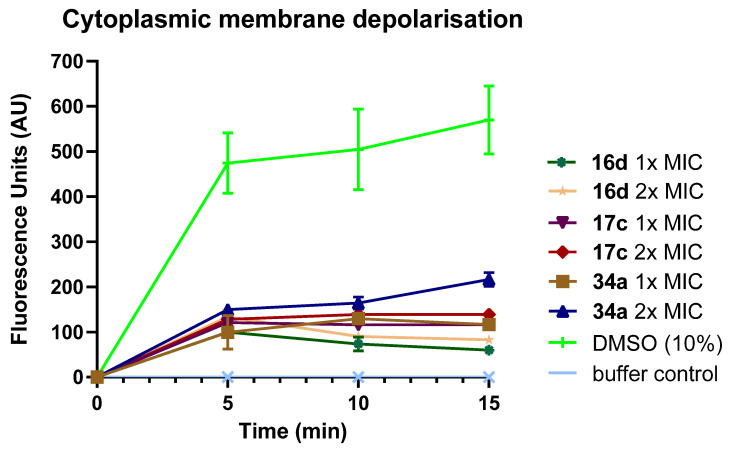
*S. aureus* cytoplasmic membrane depolarisation induced by peptidomimetics **16d**, **17c** and **34a** at 1× MIC and 2× MIC. As a positive control, 10% DMSO was used. Error bars represent the standard error of triplicates (*n* = 3).

Overall, all three tested compounds disrupted the cytoplasmic membranes of *S. aureus*, as an increase in fluorescence intensity was observed within 5 min after the deployment of the test compounds. Moreover, all test compounds disrupted cytoplasmic membranes in a concentration-dependent manner, as evidenced by a higher increase in fluorescence intensity at the higher concentration (2× MIC) of the test compound than at the lower concentration (1× MIC) (Figure 6). Amongst the three compounds, guanidinium hydrochloride salt **34a** was the most active compound at 2× MIC, as it induced the highest increase in fluorescence intensity at 15 min. However, at 1× MIC, there was no significant difference in the cytoplasmic membrane disruption ability between guanidinium hydrochloride salt **34a** and the quaternary ammonium iodide salt **17c**. The least active compound was the quaternary ammonium iodide salt **16d**, as it showed the smallest increase in fluorescence intensity at 15 min. However, it is worth mentioning that while increasing the bacterial cytoplasmic membrane permeability can be a mechanism of action of the tested compounds, these compounds can also exert their antibacterial action through other mechanisms, such as interacting with DNA, RNA or nucleotides [68,69,70,71]; these potential mechanisms of action of AMP will be investigated in future studies.

### 2.7. Cytotoxicity

The in vitro toxicity of selected potent peptidomimetics (**10d**, **13d**, **16d**, **17b**, **17c**, **18a**, **22**, **23**, **33d**, **34a**, **34b**) was assessed against MRC-5 normal human lung fibroblasts via the MTT assay. The IC_50_ values of the tested peptidomimetics were determined from a dose-response curve. TY-01 was used as the positive control in this assay with the IC_50_ value to be 14.2 μM (7.83 μg/mL) [51]. The therapeutic indices for each tested peptidomimetic against Gram-positive *S. aureus* and Gram-negative *E. coli* were calculated by dividing the IC_50_ value against human cells by the corresponding MIC value (Table 2). An antibacterial agent, which can be utilised clinically, should possess a high therapeutic index, as this indicates a high selectivity for bacterial cells over human cells.

Without the terminal cationic group, the neutral glyoxamide **10d** showed high toxicity with an IC_50_ of 3.48 μM (2.01 μg/mL) against human cells and a therapeutic index of 0.44 against *S. aureus*, indicating that it is more effective at targeting human cells rather than bacterial cells. Converting the tertiary amine group of **10d** into the tertiary ammonium chloride salt **13d** did not improve toxicity, as there was no significant difference in the IC_50_ value (~4 μM or ~2 μg/mL) between the two peptidomimetics. However, the corresponding quaternary ammonium iodide salt **16d** showed a significant improvement in the toxicity, as the IC_50_ value against human cells rose to 53.7 μM (38.6 μg/mL), giving a therapeutic index of 15.4 against *S. aureus*. On the other hand, replacing the quaternary ammonium iodide group by a guanidinium hydrochloride group (**33d**) increased toxicity, as the IC_50_ value dropped to 16.9 μM (10.6 μg/mL), giving a therapeutic index of 1.05 against *S. aureus*. A similar trend was also observed for peptidomimetics bearing different phenyl systems and alkylsulfonyl chains, with quaternary ammonium iodide salts (**17b**, **17c**, **18a**) generally having higher therapeutic indices of 2.29–7.18 against *S. aureus* than guanidinium hydrochloride salts (**33d**, **34a**, **34b**) with therapeutic indices of 1.05–3.40. Against Gram-negative *E. coli*, the therapeutic indices of tested peptidomimetics (**17b, 17c, 34a**) were between 0.57 and 1.80. The lower therapeutic indices against *E. coli* compared to those of *S. aureus* were due to the higher MIC values of the peptidomimetics against *E. coli*.

In addition to assessing the cytotoxicity of the peptidomimetic on human lung fibroblasts, the potent peptidomimetics **16d** and **17c** were tested for their cytotoxicity against human red blood cells in a haemolysis assay. Both peptidomimetics gave relatively low haemolytic activities, with **16d** and **17c** showing only 11% and 16% haemolysis, respectively, at 32 μM. This concentration corresponded to 8× and 4× MIC of **16d** and **17c**, respectively, against *S. aureus*.

Overall, the relatively low cytotoxicity (against lung fibroblast cells and erythrocytes) of peptidomimetics suggests that they are relatively non-toxic to human cells and, therefore, the corresponding high therapeutic indices indicate that the peptidomimetics **16d**, **17c**, **18a** might be suitable antibiotics to develop further for the treatment of *S. aureus* infection. However, the relatively low therapeutic indices against *E. coli* suggest that these peptidomimetics might be unsafe to be used as antibacterial agents against *E. coli*, clinically.

## 3. Materials and Methods

### 3.1. Synthesis of Analogues

#### 3.1.1. General Information

All commercially available reagents and solvents were purchased from standard suppliers (Sigma Aldrich, Alfa-Aesar, Combi-Blocks and Oakwook Chemicals) and used without further purification. All reactions were performed under anhydrous condition with an anhydrous solvent obtained using PureSolv MD Solvent Purification System unless otherwise specified. The progress of the reactions was monitored by thin layer chromatography plates precoated with Merck silica gel 60 F254 and visualised using short and/or long wavelength of ultraviolet light. Flash chromatography was carried out using Grace Davisil LC60A silica.

Melting points of the compounds were measured using an SRS MPA100 OptiMelt melting point apparatus and are reported without correction. ^1^H and ^13^C NMR spectra were acquired in the specified solvents on a Bruker Avance III HD 400 or Bruker Avance III 600 Cryo spectrometer. Chemical shifts (δ) are in parts per million (ppm) internally referenced to the solvent nuclei. Multiplicities are assigned as singlet (s), broad singlet (bs), doublet (d), triplet (t), quartet (q), multiplet (m) or a combination of these (e.g., dd, dt, td), and coupling constants (*J*) are reported in Hertz (Hz). Infrared (IR) spectra were recorded using a Cary 630 FTIR spectrometer or Nicolet^TM^ iS^TM^ 10 FTIR spectrometer fitted with a diamond attenuated total reflectance (ATR) sample interface. High-resolution mass spectrometry (HRMS) was performed, using a Thermo LTQ Orbitrap XL instrument.

#### 3.1.2. Synthetic Procedures and Experimental Characterisation Data

##### 5-Phenylindoline-2,3-dione (**4**)

The synthesis of the titled compound followed the procedure previously described [51] with slight modification. A 2 M potassium carbonate solution (7.7 mL, 15.4 mmol) was degassed for 30 min and then added into a suspension of 5-bromoisatin (1.72 g, 7.59 mmol) and phenylboronic acid (1.04 g, 8.35 mmol) in a degassed (for 30 min) 1:1 ethanol/toluene solution (30 mL). The dark brown solution mixture was further degassed for 30 min, followed by the addition of tetrakis(triphenylphosphine)palladium(0) (92 mg, 0.080 mmol). The resulting reaction mixture was heated at reflux under an atmosphere of nitrogen for 18 h. The brownish-black reaction mixture was concentrated in vacuo. Glacial acetic acid (30 mL) was added to the dark brown solid, and the resulting reaction mixture was heated at reflux for 20 min. The solution mixture was then allowed to cool to room temperature overnight. The red crystals formed was filtered and discarded. Water (50 mL) was then added to the filtrate. The organic layer was extracted thrice with dichloromethane (3 × 30 mL), washed with brine (50 mL), dried over sodium sulphate and concentrated in vacuo to give a red solid, which was purified by flash column chromatography on silica, using methanol/dichloromethane as the eluent to give the product as a red solid (1.14 g, 67%); mp 249.5–250.3 °C; ^1^H NMR (400 MHz, DMSO-*d*_6_): δ 11.12 (bs, 1H, NH), 7.91 (dd, *J* = 8.2, 2.0 Hz, 1H, ArH), 7.76 (d, *J* = 1.9 Hz, 1H, ArH), 7.67–7.63 (m, 2H, ArH), 7.48–7.43 (m. 2H, ArH), 7.39–7.33 (m, 1H, ArH), 7.01 (d, *J* = 8.2 Hz, 1H, ArH); MS (+ESI): *m*/*z* 246.12, [M+Na]^+^.

##### 5-([1,1′-Biphenyl]-4-yl)indoline-2,3-dione (**5**)

The synthesis of the titled compound followed the procedure previously described [56]. A 2 M potassium carbonate solution (9.2 mL, 18.4 mmol) was degassed for 30 min, and then added into a suspension of 5-bromoisatin (2.06 g, 9.13 mmol) and 4-biphenylboronic acid (1.89 g, 9.54 mmol) in a degassed (for 30 min) 1:1 ethanol/toluene solution (40 mL). The dark brown solution mixture was further degassed for 30 min, followed by the addition of tetrakis(triphenylphosphine)palladium(0) (109 mg, 0.094 mmol). The resulting reaction mixture was heated at reflux under an atmosphere of nitrogen for 18 h. The dark brown reaction mixture was concentrated in vacuo. Glacial acetic acid (30 mL) was added to the dark brown solid, and the resulting reaction mixture was heated at reflux for 20 min. The dark red precipitate was collected by vacuum filtration to give the crude product, which was redissolved in dimethylformamide and filtered. The filtrate was then poured into 1:1 ice-water mixture and the red precipitate formed was collected by vacuum filtration to give the product as a red solid (2.28 g, 83%); mp 239.9–240.8 °C; ^1^H NMR (400 MHz, DMSO-*d*_6_): δ 11.14 (s, 1H, NH), 7.98 (dd, *J* = 8.2, 2.1 Hz, 1H, ArH), 7.84 (d, *J* = 2.0 Hz, 1H, ArH), 7.79–7.69 (m, 6H, ArH), 7.52–7.45 (m, 2H, ArH), 7.41–7.35 (m, 1H, ArH), 7.03 (d, *J* = 8.2 Hz, 1H, ArH); HRMS (+ESI): Found *m*/*z* 322.0839 [M+Na]^+^, C_20_H_13_NO_2_Na required 322.0838.

**General synthetic procedure A for *N*-sulfonylisatins**

Triethylamine (1.1 equivalents) was added slowly to a stirring solution of an appropriate isatin (1.0 equivalent) in dichloromethane (15 mL) at 0 °C in a nitrogen atmosphere. The reaction mixture was stirred at 0 °C for 20 min, followed by the dropwise addition of an appropriate 1-alkylsulfonyl chloride (1.0 equivalent) in dichloromethane (5 mL) with stirring. The reaction mixture was then stirred at room temperature for 3 h. After the completion of the reaction, the reaction mixture was concentrated in vacuo and washed with methanol to afford the product.

##### 5-([1,1′-Biphenyl]-4-yl)-1-(octylsulfonyl)indoline-2,3-dione (**7d**)

The titled compound was synthesised from 5-([1,1′-biphenyl]-4-yl)indoline-2,3-dione **6d** (0.44 g, 1.46 mmol), triethylamine (0.23 mL, 1.65 mmol) and 1-octanesulfonyl chloride (0.30 mL, 1.53 mmol) following **general synthetic procedure A**. After washing the crude with methanol, the precipitate was collected by vacuum filtration, and the filtrate was discarded. The residue was then washed with dichloromethane (5 mL) and filtered. The filtrate was concentrated in vacuo to afford the product as an orange solid (0.28 g, 40%); mp 173.5–173.9 °C; ^1^H NMR (400 MHz, DMSO-*d*_6_): δ 8.14 (dd, *J* = 8.6, 2.0 Hz, 1H, ArH), 8.05 (d, *J* = 2.0 Hz, 1H, ArH), 7.87–7.70 (m, 7H, ArH), 7.50 (t, *J* = 7.8 Hz, 2H, ArH), 7.39 (t, *J* = 7.2 Hz, 1H, ArH), 3.67–3.60 (m, 2H, CH_2_), 1.87–1.76 (m, 2H, CH_2_), 1.44–1.15 (m, 10H, CH_2_), 0.84 (t, *J* = 7.1 Hz, 3H, CH_3_); ^13^C NMR (100 MHz, DMSO-*d*_6_): δ 178.7 (CO), 156.7 (CO), 146.2 (ArC), 139.7 (ArC), 139.4 (ArC), 136.9 (ArC), 136.4 (ArC), 135.7 (ArCH), 129.1 (ArCH), 127.7 (ArCH), 127.4 (ArCH), 127.1 (ArCH), 126.6 (ArCH), 122.3 (ArCH), 120.2 (ArC), 114.7 (ArCH), 53.7 (CH_2_), 31.1 (CH_2_), 28.4 (CH_2_), 28.4 (CH_2_), 27.3 (CH_2_), 22.2 (CH_2_), 22.0 (CH_2_), 13.9 (CH_3_); IR (ATR): *ν*_max_ 3854, 3675, 2987, 2900, 1765, 1739, 1613, 1576, 1559, 1473, 1434, 1405, 1393, 1373, 1309, 1290, 1254, 1240, 1174, 1140, 1075, 1066, 1057, 1027, 944, 892, 851, 836, 766, 729, 719, 692 cm^−1^; HRMS (+ESI): Found *m*/*z* 498.1710 [M+Na]^+^, C_28_H_29_NO_4_SNa required 498.1710.

##### 1-(Dodecylsulfonyl)indoline-2,3-dione (**8a**)

The titled compound was synthesised from isatin **6a** (0.23 g, 1.54 mmol), triethylamine (0.24 mL, 1.72 mmol) and 1-dodecanesulfonyl chloride (0.42 g, 1.56 mmol) following **general synthetic procedure A**. The product was obtained as a yellow solid (0.30 g, 51%); mp 128.1–129.6 °C; ^1^H NMR (400 MHz, DMSO-*d*_6_): δ 7.78–7.68 (m, 3H, ArH), 7.36–7.28 (m, 1H, ArH), 3.64–3.56 (m, 2H, CH_2_), 1.83–1.72 (m, 2H, CH_2_), 1.41–1.14 (m, 18H, CH_2_), 0.85 (t, *J* = 7.1 Hz, 3H, CH_3_); ^13^C NMR (100 MHz, DMSO-*d*_6_): δ 178.8 (CO), 156.5 (CO), 146.9 (ArC), 137.9 (ArCH), 125.1 (ArCH), 125.0 (ArCH), 119.4 (ArC), 114.1 (ArCH), 53.7 (CH_2_), 31.3 (CH_2_), 29.0 (CH_2_), 28.9 (CH_2_), 28.7 (CH_2_), 28.7 (CH_2_), 28.4 (CH_2_), 27.3 (CH_2_), 22.1 (CH_2_), 22.1 (CH_2_), 14.0 (CH_3_); IR (ATR): *ν*_max_ 2996, 2913. 2847, 1912, 1733, 1651, 1593, 1459, 1413, 1371, 1320, 1237, 1177, 1132, 1090, 1024, 933, 831, 784, 756 cm^−1^; HRMS (+ESI): Found *m*/*z* 380.1891 [M+H]^+^, C_20_H_30_NO_4_S required 380.1890.

##### 5-Bromo-1-(dodecylsulfonyl)indoline-2,3-dione (**8b**)

The titled compound was synthesised from 5-bromoisatin **6b** (0.35 g, 1.55 mmol), triethylamine (0.24 mL, 1.72 mmol) and 1-dodecanesulfonyl chloride (0.42 g, 1.58 mmol) following **general synthetic procedure A**. The product was obtained as a yellow solid (0.42 g, 60%); mp 141.9 °C; ^1^H NMR (400 MHz, DMSO-*d*_6_): δ 7.98–7.84 (m, 2H, ArH), 7.65 (d, *J* = 8.5 Hz, 1H, ArH), 3.65–3.54 (m, 2H, CH_2_), 1.85–1.68 (m, 2H, CH_2_), 1.44–1.10 (m, 18H, CH_2_), 0.92–0.78 (m, 3H, CH_3_); ^13^C NMR (100 MHz, DMSO-*d*_6_): δ 177.5 (CO), 156.0 (CO), 145.7 (ArC), 139.6 (ArCH), 127.1 (ArCH), 121.4 (ArC), 117.2 (ArC), 116.1 (ArCH), 53.7 (CH_2_), 31.3 (CH_2_), 29.0 (CH_2_), 28.9 (CH_2_), 28.7 (CH_2_), 28.7 (CH_2_), 28.4 (CH_2_), 27.3 (CH_2_), 22.0 (CH_2_), 14.0 (CH_3_); IR (ATR): *ν*_max_ 3854, 3675, 3104, 3066, 2969, 2913, 2848, 2360, 1763, 1742, 1717, 1596, 1578, 1461, 1427, 1413, 1393, 1372, 1290, 1270, 1234, 1191, 1177, 1140, 1116, 1095, 1077, 1065, 1057, 1027, 939, 898, 845, 786, 722, 707, 696 cm^−1^; HRMS (+ESI): Found *m*/*z* 480.0816 [M+Na]^+^, C_20_H_28_BrNO_4_SNa required 480.0815.

##### 1-(Dodecylsulfonyl)-5-phenylindoline-2,3-dione (**8c**)

The titled compound was synthesised from 5-phenylindoline-2,3-dione **6c** (1.00 g, 4.48 mmol), triethylamine (0.70 mL, 5.02 mmol) and 1-dodecanesulfonyl chloride (1.21 g, 4.51 mmol) following **general synthetic procedure A**. The product was obtained as a yellow solid (0.94 g, 46%); mp 137.9–138.2 °C; ^1^H NMR (400 MHz, DMSO-*d*_6_): δ 8.07 (dd, *J* = 8.6, 2.2 Hz, 1H, ArH), 7.98 (d, *J* = 1.9 Hz, 1H, ArH), 7.80 (d, *J* = 8.6 Hz, 1H, ArH), 7.75–7.70 (m, 2H, ArH), 7.52–7.46 (m. 2H, ArH), 7.43–7.38 (m, 1H, ArH), 3.67–3.58 (m, 2H, CH_2_), 1.86–1.74 (m, 2H, CH_2_), 1.42–1.16 (m, 18H, CH_2_), 0.84 (t, *J* = 6.9 Hz, 3H, CH_3_); ^13^C NMR (100 MHz, DMSO-*d*_6_): δ 178.7 (CO), 156.6 (CO), 146.1 (ArC), 138.0 (ArC), 137.0 (ArC), 135.8 (ArCH), 129.1 (ArCH), 128.0 (ArCH), 126.5 (ArCH), 122.4 (ArCH), 120.1 (ArC), 114.6 (ArCH), 53.7 (CH_2_), 31.3 (CH_2_), 29.0 (CH_2_), 28.9 (CH_2_), 28.7 (CH_2_), 28.7 (CH_2_), 28.4 (CH_2_), 27.3 (CH_2_), 22.2 (CH_2_), 22.1 (CH_2_), 13.9 (CH_3_); IR (ATR): *ν*_max_ 3675, 2969, 2914, 2848, 2360, 1764, 1735, 1614, 1584, 1570, 1507, 1471, 1454, 1426, 1411, 1393, 1375, 1308, 1294, 1279, 1258, 1239, 1174, 1140, 1113, 1095, 1077, 1066, 1057, 1027, 944, 907, 852, 786, 763, 722, 704, 698, 650 cm^−1^; HRMS (+ESI): Found *m*/*z* 478.2024 [M+Na]^+^, C_26_H_33_NO_4_SNa required 478.2023.

##### 5-([1,1′-Biphenyl]-4-yl)-1-(dodecylsulfonyl)indoline-2,3-dione (**8d**)

The titled compound was synthesised from 5-([1,1′-biphenyl]-4-yl)indoline-2,3-dione **6d** (0.48 g, 1.59 mmol), triethylamine (0.25 mL, 1.79 mmol) and 1-dodecanesulfonyl chloride (0.43 g, 1.60 mmol) following **general synthetic procedure A**. After washing the crude with methanol, the precipitate was collected by vacuum filtration and the filtrate was discarded. The residue was then washed with dichloromethane (5 mL) and filtered. The filtrate was concentrated in vacuo to afford the product as an orange solid (0.47 g, 56%); mp 165.5–167.7 °C; ^1^H NMR (600 MHz, DMSO-*d*_6_): δ 8.14 (dd, *J* = 8.6, 2.1 Hz, 1H, ArH), 8.05 (d, *J* = 2.0 Hz, 1H, ArH), 7.87–7.76 (m, 5H, ArH), 7.76–7.71 (m, 2H, ArH), 7.50 (t, *J* = 7.8 Hz, 2H, ArH), 7.40 (t, *J* = 7.5 Hz, 1H, ArH), 3.66–3.61 (m, 2H, CH_2_), 1.85–1.78 (m, 2H, CH_2_), 1.41–1.34 (m, 2H, CH_2_), 1.29–1.14 (m, 16H, CH_2_), 0.83 (t, *J* = 7.1 Hz, 3H, CH_3_); ^13^C NMR (150 MHz, DMSO-*d*_6_): δ 178.7 (CO), 156.6 (CO), 146.1 (ArC), 139.7 (ArC), 139.4 (ArC), 136.9 (ArC), 136.4 (ArC), 135.7 (ArCH), 129.0 (ArCH), 127.7 (ArCH), 127.3 (ArCH), 127.0 (ArCH), 126.6 (ArCH), 122.3 (ArCH), 120.2 (ArC), 114.6 (ArCH), 53.7 (CH_2_), 31.3 (CH_2_), 29.0 (CH_2_), 28.9 (CH_2_), 28.7 (CH_2_), 28.7 (CH_2_), 28.4 (CH_2_), 27.3 (CH_2_), 22.2 (CH_2_), 22.1 (CH_2_), 13.9 (CH_3_); IR (ATR): *ν*_max_ 3032, 2914, 2848, 2679, 2343, 2107, 1738, 1613, 1577, 1471, 1373, 1289, 1237, 1174, 1114, 1023, 943, 851, 765, 719, 693 cm^−1^; HRMS (+ESI): Found *m*/*z* 554.2336 [M+Na]^+^, C_32_H_37_NO_4_SNa required 554.2336.

##### 1-(Hexadecylsulfonyl)indoline-2,3-dione (**9a**)

The titled compound was synthesised from isatin **6a** (0.31 g, 2.07 mmol), triethylamine (0.32 mL, 2.30 mmol) and 1-hexadecanesulfonyl chloride (0.68 g, 2.09 mmol) following **general synthetic procedure A**. The product was obtained as a yellow solid (0.36 g, 40%); mp 122.0–123.9 °C; ^1^H NMR (400 MHz, CDCl_3_): δ 7.92 (d, *J* = 8.4 Hz, 1H, ArH), 7.78 (dd, *J* = 7.6, 0.9 Hz, 1H, ArH), 7.74–7.68 (m, 1H, ArH), 7.32 (td, *J* = 7.6, 0.6 Hz, 1H, ArH), 3.62–3.55 (m, 2H, CH_2_), 1.94–1.83 (m, 2H, CH_2_), 1.49–1.38 (m, 2H, CH_2_), 1.34–1.18 (m, 24H, CH_2_), 0.87 (t, *J* = 7.1 Hz, 3H, CH_3_); ^13^C NMR (100 MHz, CDCl_3_): δ 179.0 (CO), 156.7 (CO), 148.1 (ArC), 139.6 (ArCH), 126.3 (ArCH), 126.1 (ArCH), 118.8 (ArC), 115.4 (ArCH), 54.9 (CH_2_), 32.1 (CH_2_), 29.8 (CH_2_), 29.8 (CH_2_), 29.8 (CH_2_), 29.7 (CH_2_), 29.7 (CH_2_), 29.5 (CH_2_), 29.5 (CH_2_), 29.3 (CH_2_), 29.0 (CH_2_), 28.1 (CH_2_), 23.0 (CH_2_), 22.8 (CH_2_), 14.3 (CH_3_); IR (ATR): *ν*_max_ 2914, 2846, 2669, 2343, 1912, 1733, 1651, 1596, 1459, 1413, 1372, 1320, 1237, 1177, 1132, 1090, 981, 934, 832, 786, 756, 723, 676 cm^−1^; HRMS (+ESI): Found *m*/*z* 436.2515 [M+H]^+^, C_24_H_38_NO_4_S required 436.2516.

##### 5-Bromo-1-(hexadecylsulfonyl)indoline-2,3-dione (**9b**)

The titled compound was synthesised from 5-bromoisatin **6b** (0.50 g, 2.19 mmol), triethylamine (0.34 mL, 2.44 mmol) and 1-hexadecanesulfonyl chloride (0.72 g, 2.20 mmol) following **general synthetic procedure A**. The product was obtained as a yellow solid (0.65 g, 58%); mp 142.4–144.0 °C; ^1^H NMR (600 MHz, DMSO-*d*_6_): δ 7.92–7.89 (m, 2H, ArH), 7.67–7.64 (m, 1H, ArH), 3.62–3.56 (m, 2H, CH_2_), 1.81–1.73 (m, 2H, CH_2_), 1.39–1.31 (m, 2H, CH_2_), 1.30–1.16 (m, 24H, CH_2_), 0.85 (t, *J* = 7.2 Hz, 3H, CH_3_); ^13^C NMR (150 MHz, DMSO-*d*_6_): δ 177.5 (CO), 156.0 (CO), 145.7 (ArC), 139.6 (ArCH), 127.1 (ArCH), 121.4 (ArC), 117.2 (ArC), 116.2 (ArCH), 53.8 (CH_2_), 31.3 (CH_2_), 29.0 (CH_2_), 29.0 (CH_2_), 29.0 (CH_2_), 29.0 (CH_2_), 28.9 (CH_2_), 28.7 (CH_2_), 28.7 (CH_2_), 28.4 (CH_2_), 27.3 (CH_2_), 22.1 (CH_2_), 14.0 (CH_3_); IR (ATR): *ν*_max_ 3473, 3067, 2998, 2914, 2846, 2670, 2116, 1741, 1595, 1460, 1372, 1290, 1234, 1176, 1115, 1016, 938, 845, 786, 721 cm^−1^; HRMS (+ESI): Found *m*/*z* 536.1433 [M+Na]^+^, C_24_H_36_BrNO_4_SNa required 536.1441.

##### 1-(Hexadecylsulfonyl)-5-phenylindoline-2,3-dione (**9c**)

The titled compound was synthesised from 5-phenylindoline-2,3-dione **6c** (0.81 g, 3.61 mmol), triethylamine (0.55 mL, 3.95 mmol) and 1-hexadecanesulfonyl chloride (1.17 g, 3.61 mmol) following **general synthetic procedure A**. The product was obtained as a yellow solid (0.53 g, 28%); mp 139.6–141.3 °C; ^1^H NMR (600 MHz, CDCl_3_): δ 8.00–7.96 (m, 2H, ArH), 7.92 (dd, *J* = 8.7, 1.9 Hz, 1H, ArH), 7.57–7.54 (m, 2H, ArH), 7.50–7.46 (m, 2H, ArH), 7.44–7.40 (m, 1H, ArH), 3.63–3.58 (m, 2H, CH_2_), 1.95–1.87 (m, 2H, CH_2_), 1.50–1.42 (m, 2H, CH_2_), 1.34–1.20 (m, 24H, CH_2_), 0.88 (t, *J* = 7.2 Hz, 3H, CH_3_); ^13^C NMR (150 MHz, CDCl_3_): δ 179.2 (CO), 156.8 (CO), 147.1 (ArC), 139.6 (ArC), 138.3 (ArC), 138.0 (ArCH), 129.4 (ArCH), 128.6 (ArCH), 126.9 (ArCH), 124.4 (ArCH), 119.2 (ArC), 115.8 (ArCH), 55.0 (CH_2_), 32.1 (CH_2_), 29.8 (CH_2_), 29.8 (CH_2_), 29.8 (CH_2_), 29.8 (CH_2_), 29.7 (CH_2_), 29.6 (CH_2_), 29.5 (CH_2_), 29.3 (CH_2_), 29.0 (CH_2_), 28.2 (CH_2_), 23.0 (CH_2_), 22.8 (CH_2_), 14.3 (CH_3_); IR (ATR): *ν*_max_ 3055, 2914, 2847, 2343, 1892, 1736, 1614, 1584, 1505, 1469, 1426, 1375, 1308, 1237, 1175, 1114, 1040, 943, 853, 762, 700 cm^−1^; HRMS (+ESI): Found *m*/*z* 512.2812 [M+H]^+^, C_30_H_42_NO_4_S required 512.2829.

**General synthetic procedure B for glyoxamide derivatives**

3-Dimethylaminopropylamine (1.0 equivalent) was added to a stirring solution of *N*-sulfonylisatin (1.0 equivalent) in dichloromethane (5 mL) at 0 °C. The resulting reaction mixture was stirred at room temperature for 6 h. After the completion of the reaction, water was added to the reaction mixture, and the mixture was extracted into dichloromethane (3 × 25 mL), washed with brine, dried over anhydrous sodium sulfate and concentrated in vacuo to afford the product.

##### *N*-(3-(Dimethylamino)propyl)-2-(4-(octylsulfonamido)-[1,1′:4′,1″-terphenyl]-3-yl)-2-oxoacetamide (**10d**)

The titled compound was synthesised from 5-([1,1′-biphenyl]-4-yl)-1-(octylsulfonyl)indoline-2,3-dione **7d** (0.10 g, 0.21 mmol) and 3-dimethylaminopropylamine (27 μL, 0.21 mmol) following **general synthetic procedure B**. The crude was purified by flash column chromatography on silica, using methanol/dichloromethane (1:9) as an eluent to afford the product as a yellow oil (75 mg, 61%); ^1^H NMR (600 MHz, CDCl_3_): δ 8.57 (d, *J* = 2.1 Hz, 1H, ArH), 8.44 (bs, 1H, ArH), 7.86 (dd, *J* = 8.7, 2.2 Hz, 1H, ArH), 7.82 (d, *J* = 8.7 Hz, 1H, ArH), 7.70–7.66 (m, 2H, ArH), 7.66–7.61 (m, 4H, ArH), 7.49–7.44 (m, 2H, ArH), 7.39–7.35 (m, 1H, ArH), 3.66–3.59 (m, 2H, CH_2_), 3.23–3.17 (m, 2H, CH_2_), 3.12–3.04 (m, 2H, CH_2_), 2.76 (s, 6H, CH_3_), 2.19–2.11 (m, 2H, CH_2_), 1.87–1.80 (m, 2H, CH_2_), 1.43–1.35 (m, 2H, CH_2_), 1.30–1.18 (m, 8H, CH_2_), 0.85 (t, *J* = 7.2 Hz, 3H, CH_3_); ^13^C NMR (150 MHz, CDCl_3_): δ 191.7 (CO), 164.0 (CO), 140.8 (ArC), 140.5 (ArC), 137.9 (ArC), 135.7 (ArC), 134.7 (ArCH), 133.0 (ArCH), 129.0 (ArCH), 127.9 (ArCH), 127.7 (ArCH), 127.3 (ArCH), 127.2 (ArCH), 120.6 (ArC), 119.3 (ArCH), 55.9 (CH_2_), 52.9 (CH_2_), 43.5 (CH_3_), 36.9 (CH_2_), 31.8 (CH_2_), 29.1 (CH_2_), 29.1 (CH_2_), 28.3 (CH_2_), 24.4 (CH_2_), 23.5 (CH_2_), 22.7 (CH_2_), 14.2 (CH_3_); IR (ATR): *ν*_max_ 3674, 3384, 2970, 2921, 1767, 1740, 1646, 1615, 1481, 1405, 1393, 1374, 1333, 1241, 1196, 1142, 1075, 1066, 1057, 1027, 915, 829, 765, 729, 696, 673 cm^−1^; HRMS (+ESI): Found *m*/*z* 578.3074 [M+H]^+^, C_33_H_44_N_3_O_4_S required 578.3047.

##### *N*-(3-(Dimethylamino)propyl)-2-(2-(dodecylsulfonamido)phenyl)-2-oxoacetamide (**11a**)

The titled compound was synthesised from 1-(dodecylsulfonyl)indoline-2,3-dione **8a** (0.11 g, 0.28 mmol) and 3-dimethylaminopropylamine (36 μL, 0.28 mmol) following **general synthetic procedure B**. The product was obtained as a yellow oil (0.13 g, 98%); ^1^H NMR (400 MHz, CDCl_3_): δ 8.76 (bs, 1H, NH), 8.45 (dd, *J* = 8.1, 1.6 Hz, 1H, ArH), 7.77 (dd, *J* = 8.5, 0.9 Hz, 1H, ArH), 7.62–7.56 (m, 1H, ArH), 7.18–7.11 (m, 1H, ArH), 3.55–3.46 (m, 2H, CH_2_), 3.18–3.11 (m, 2H, CH_2_), 2.49 (t, *J* = 6.2 Hz, 2H, CH_2_), 2.28 (s, 6H, CH_3_), 1.85–1.71 (m, 4H, CH_2_), 1.40–1.16 (m, 18H, CH_2_), 0.87 (t, *J* = 7.1 Hz, 3H, CH_3_); ^13^C NMR (100 MHz, CDCl_3_): δ 192.1 (CO), 162.8 (CO), 142.0 (ArC), 136.5 (ArCH), 135.3 (ArCH), 122.6 (ArCH), 119.0 (ArC), 117.9 (ArCH), 59.0 (CH_2_), 52.6 (CH_2_), 45.4 (CH_3_), 40.1 (CH_2_), 32.0 (CH_2_), 29.7 (CH_2_), 29.7 (CH_2_), 29.6 (CH_2_), 29.5 (CH_2_), 29.4 (CH_2_), 29.1 (CH_2_), 28.2 (CH_2_), 25.3 (CH_2_), 23.5 (CH_2_), 22.8 (CH_2_), 14.3 (CH_3_); IR (ATR): *ν*_max_ 3372, 3068, 2921, 2852, 2543, 2341, 2119, 1635, 1460, 1336, 1262, 1144, 1106, 1041, 941, 754, 719 cm^−1^; HRMS (+ESI): Found *m*/*z* 482.3047 [M+H]^+^, C_25_H_44_N_3_O_4_S required 482.3047.

##### 2-(5-Bromo-2-(dodecylsulfonamido)phenyl)-*N*-(3-(dimethylamino)propyl)-2-oxoacetamide (**11b**)

The titled compound was synthesised from 5-bromo-1-(dodecylsulfonyl)indoline-2,3-dione **8b** (0.15 g, 0.33 mmol) and 3-dimethylaminopropylamine (41 μL, 0.33 mmol) following **general synthetic procedure B**. The product was obtained as a yellow oil (0.18 g, 99%); ^1^H NMR (400 MHz, CDCl_3_): δ 8.95 (bs, 1H, NH), 7.66–7.53 (m, 1H, ArH), 7.71–7.64 (m, 2H, ArH), 3.51 (t, *J* = 6.1 Hz, 2H, CH_2_), 3.16–3.08 (m, 2H, CH_2_), 2.50 (t, *J* = 6.1 Hz, 2H, CH_2_), 2.29 (s, 6H, CH_3_), 1.83–1.72 (m, 4H, CH_2_), 1.41–1.17 (m, 18H, CH_2_), 0.87 (t, *J* = 7.0 Hz, 3H, CH_3_); ^13^C NMR (100 MHz, CDCl_3_): δ 190.8 (CO), 162.0 (CO), 140.9 (ArC), 139.1 (ArCH), 137.5 (ArCH), 120.7 (ArC), 119.9 (ArCH), 115.3 (ArC), 59.1 (CH_2_), 52.8 (CH_2_), 45.4 (CH_3_), 40.3 (CH_2_), 32.0 (CH_2_), 29.7 (CH_2_), 29.7 (CH_2_), 29.6 (CH_2_), 29.5 (CH_2_), 29.4 (CH_2_), 29.1 (CH_2_), 28.2 (CH_2_), 25.1 (CH_2_), 23.5 (CH_2_), 22.8 (CH_2_), 14.3 (CH_3_); IR (ATR): *ν*_max_ 3674, 2970, 2921, 2361, 1653, 1464, 1405, 1393, 1334, 1259, 1270, 1148, 1075, 1066, 1027, 891, 800, 719, 653 cm^−1^; HRMS (+ESI): Found *m*/*z* 560.2153 [M+H]^+^, C_25_H_43_BrN_3_O_4_S required 560.2152.

##### *N*-(3-(Dimethylamino)propyl)-2-(4-(dodecylsulfonamido)-[1,1′-biphenyl]-3-yl)-2-oxoacetamide (**11c**)

The titled compound was synthesised from 1-(dodecylsulfonyl)-5-phenylindoline-2,3-dione **8c** (0.13 g, 0.28 mmol) and 3-dimethylaminopropylamine (35 μL, 0.28 mmol) following **general synthetic procedure B**. The product was obtained as a yellow oil (0.16 g, 99%); ^1^H NMR (400 MHz, CDCl_3_): δ 8.82 (bs, 1H, NH), 8.76–8.71 (m, 1H, ArH), 7.88–7.79 (m, 2H, ArH), 7.61–7.54 (m, 2H, ArH), 7.47–7.41 (m, 2H, ArH), 7.39–7.33 (m, 1H, ArH), 3.53 (t, *J* = 6.0 Hz, 2H, CH_2_), 3.22–3.14 (m, 2H, CH_2_), 2.50 (t, *J* = 6.1 Hz, 2H, CH_2_), 2.29 (s, 6H, CH_3_), 1.87–1.73 (m, 4H, CH_2_), 1.44–1.16 (m, 18H, CH_2_), 0.87 (t, *J* = 7.1 Hz, 3H, CH_3_); ^13^C NMR (100 MHz, CDCl_3_): δ 192.0 (CO), 162.7 (CO), 140.9 (ArC), 139.1 (ArC), 135.7 (ArC), 134.9 (ArCH), 133.7 (ArCH), 129.1 (ArCH), 127.9 (ArCH), 127.0 (ArCH), 119.6 (ArC), 118.5 (ArCH), 58.9 (CH_2_), 52.7 (CH_2_), 45.4 (CH_3_), 40.1 (CH_2_), 32.0 (CH_2_), 29.7 (CH_2_), 29.7 (CH_2_), 29.6 (CH_2_), 29.4 (CH_2_), 29.4 (CH_2_), 29.1 (CH_2_), 28.2 (CH_2_), 25.3 (CH_2_), 23.5 (CH_2_), 22.8 (CH_2_), 14.2 (CH_3_); IR (ATR): *ν*_max_ 3675, 3344, 2970, 2920, 2853, 2759, 2362, 1661, 1632, 1582, 1511, 1487, 1466, 1394, 1344, 1261, 1225, 1201, 1140, 1076, 1066, 1056, 928, 893, 857, 801, 761, 722, 697, 684 cm^−1^; HRMS (+ESI): Found *m*/*z* 558.3361 [M+H]^+^, C_31_H_48_N_3_O_4_S required 558.3360.

##### *N*-(3-(Dimethylamino)propyl)-2-(4-(dodecylsulfonamido)-[1,1′:4′,1″-terphenyl]-3-yl)-2-oxoacetamide (**11d**)

The titled compound was synthesised from 5-([1,1′-biphenyl]-4-yl)-1-(dodecylsulfonyl)indoline-2,3-dione **8d** (0.17 g, 0.31 mmol) and 3-dimethylaminopropylamine (40 μL, 0.31 mmol) following **general synthetic procedure B**. The crude was purified by flash column chromatography on silica, using methanol/dichloromethane (1:9) as an eluent to afford the product as a yellow oil (0.11 g, 53%); ^1^H NMR (400 MHz, CDCl_3_): δ 8.64 (d, *J* = 1.9 Hz, 1H, ArH), 8.53 (bs, 1H, NH), 7.88–7.76 (m, 2H, ArH), 7.70–7.55 (m, 6H, ArH), 7.49–7.42 (m, 2H, ArH), 7.40–7.34 (m, 1H, ArH), 3.64–3.53 (m, 2H, CH_2_), 3.22–3.14 (m, 2H, CH_2_), 2.96–2.85 (m, 2H, CH_2_), 2.62 (s, 6H, CH_3_), 2.09–1.97 (m, 2H, CH_2_), 1.87–1.77 (m, 2H, CH_2_), 1.43–1.15 (m, 18H, CH_2_), 0.87 (t, *J* = 7.1 Hz, 3H, CH_3_); ^13^C NMR (100 MHz, CDCl_3_): δ 191.8 (CO), 163.6 (CO), 140.8 (ArC), 140.6 (ArC), 140.6 (ArC), 137.9 (ArC), 135.5 (ArC), 134.7 (ArCH), 133.1 (ArCH), 129.0 (ArCH), 127.8 (ArCH), 127.7 (ArCH), 127.3 (ArCH), 127.2 (ArCH), 120.5 (ArC), 119.2 (ArCH), 58.3 (CH_2_), 52.8 (CH_2_), 44.1 (CH_3_), 39.4 (CH_2_), 32.0 (CH_2_), 29.7 (CH_2_), 29.7 (CH_2_), 29.6 (CH_2_), 29.6 (CH_2_), 29.5 (CH_2_), 29.4 (CH_2_), 29.1 (CH_2_), 28.3 (CH_2_), 24.7 (CH_2_), 23.5 (CH_2_), 22.8 (CH_2_), 14.3 (CH_3_); IR (ATR): *ν*_max_ 3341, 3190, 3030, 2921, 2851, 2527, 2342, 2111, 1911, 1610, 1481, 1390, 1333, 1276, 1196, 1141, 994, 919, 826, 764, 696 cm^−1^; HRMS (+ESI): Found *m*/*z* 634.3673 [M+H]^+^, C_37_H_52_N_3_O_4_S required 634.3673.

##### *N*-(3-(Dimethylamino)propyl)-2-(2-(hexadecylsulfonamido)phenyl)-2-oxoacetamide (**12a**)

The titled compound was synthesised from 1-(hexadecylsulfonyl)indoline-2,3-dione **9a** (0.13 g, 0.29 mmol) and 3-dimethylaminopropylamine (36 μL, 0.29 mmol) following **general synthetic procedure B**. The product was obtained as a yellow sticky solid (0.15 g, 96%); ^1^H NMR (400 MHz, CDCl_3_): δ 8.74 (bs, 1H, NH), 8.45 (dd, *J* = 8.1, 1.5 Hz, 1H, ArH), 7.77 (dd, *J* = 8.5, 0.5 Hz, 1H, ArH), 7.62–7.56 (m, 1H, ArH), 7.18–7.10 (m, 1H, ArH), 3.51 (t, *J* = 5.9 Hz, 2H, CH_2_), 3.17–3.11 (m, 2H, CH_2_), 2.48 (t, *J* = 6.2 Hz, 2H, CH_2_), 2.27 (s, 6H, CH_3_), 1.83–1.71 (m, 4H, CH_2_), 1.40–1.16 (m, 26H, CH_2_), 0.87 (t, *J* = 7.0 Hz, 3H, CH_3_); ^13^C NMR (100 MHz, CDCl_3_): δ 192.1 (CO), 162.8 (CO), 142.0 (ArC), 136.5 (ArCH), 135.3 (ArCH), 122.6 (ArCH), 119.0 (ArC), 117.9 (ArCH), 58.9 (CH_2_), 52.6 (CH_2_), 45.4 (CH_3_), 40.0 (CH_2_), 32.0 (CH_2_), 29.8 (CH_2_), 29.8 (CH_2_), 29.7 (CH_2_), 29.6 (CH_2_), 29.5 (CH_2_), 29.4 (CH_2_), 29.1 (CH_2_), 28.2 (CH_2_), 25.3 (CH_2_), 23.5 (CH_2_), 22.8 (CH_2_), 14.3 (CH_3_); IR (ATR): *ν*_max_ 3351, 3229, 3057, 2920, 2851, 2781, 2533, 2119, 1633, 1527, 1463, 1401, 1337, 1262, 1232, 1147, 1100, 1042, 984, 918, 755, 673 cm^−1^; HRMS (+ESI): Found *m*/*z* 538.3672 [M+H]^+^, C_29_H_52_N_3_O_4_S required 538.3673.

##### 2-(5-Bromo-2-(hexadecylsulfonamido)phenyl)-*N*-(3-(dimethylamino)propyl)-2-oxoacetamide (**12b**)

The titled compound was synthesised from 5-bromo-1-(hexadecylsulfonyl)indoline-2,3-dione **9b** (0.15 g, 0.30 mmol) and 3-dimethylaminopropylamine (38 μL, 0.30 mmol) following **general synthetic procedure B**. The product was obtained as a yellow oil (0.18 g, 98%); ^1^H NMR (400 MHz, CDCl_3_): δ 8.94 (bs, 1H, NH), 7.64–7.63 (m, 1H, ArH), 7.69–7.66 (m, 2H, ArH), 3.52 (t, *J* = 6.2 Hz, 2H, CH_2_), 3.16–3.08 (m, 2H, CH_2_), 2.52 (t, *J* = 6.1 Hz, 2H, CH_2_), 2.30 (s, 6H, CH_3_), 1.82–1.71 (m, 4H, CH_2_), 1.42–1.17 (m, 26H, CH_2_), 0.87 (t, *J* = 7.0 Hz, 3H, CH_3_); ^13^C NMR (100 MHz, CDCl_3_): δ 190.8 (CO), 162.1 (CO), 140.8 (ArC), 139.1 (ArCH), 137.4 (ArCH), 120.8 (ArC), 119.9 (ArCH), 115.3 (ArC), 58.9 (CH_2_), 52.8 (CH_2_), 45.3 (CH_3_), 40.2 (CH_2_), 32.1 (CH_2_), 29.8 (CH_2_), 29.8 (CH_2_), 29.8 (CH_2_), 29.8 (CH_2_), 29.8 (CH_2_), 29.7 (CH_2_), 29.6 (CH_2_), 29.5 (CH_2_), 29.4 (CH_2_), 29.1 (CH_2_), 28.2 (CH_2_), 25.1 (CH_2_), 23.5 (CH_2_), 22.8 (CH_2_), 14.3 (CH_3_); IR (ATR): *ν*_max_ 2922, 2852, 2361, 1682, 1575, 1465, 1334, 1285, 1148, 1100, 972, 868, 823, 720 cm^−1^; HRMS (+ESI): Found *m*/*z* 616.2779 [M+H]^+^, C_29_H_51_BrN_3_O_4_S required 616.2778.

##### *N*-(3-(Dimethylamino)propyl)-2-(4-(hexadecylsulfonamido)-[1,1′-biphenyl]-3-yl)-2-oxoacetamide (**12c**)

The titled compound was synthesised from 1-(hexadecylsulfonyl)-5-phenylindoline-2,3-dione **9c** (0.11 g, 0.21 mmol) and 3-dimethylaminopropylamine (26 μL, 0.21 mmol) following **general synthetic procedure B**. The product was obtained as a yellow solid (0.12 g, 96%); mp 76.7–79.4 °C; ^1^H NMR (400 MHz, CDCl_3_): δ 8.74 (bs, 1H, NH), 8.71 (d, *J* = 1.7 Hz, 1H, ArH), 7.87–7.79 (m, 2H, ArH), 7.60–7.55 (m, 2H, ArH), 7.48–7.41 (m, 2H, ArH), 7.39–7.33 (m, 1H, ArH), 3.54 (t, *J* = 5.6 Hz, 2H, CH_2_), 3.21–3.14 (m, 2H, CH_2_), 2.59 (t, *J* = 6.1 Hz, 2H, CH_2_), 2.36 (s, 6H, CH_3_), 1.89–1.75 (m, 4H, CH_2_), 1.42–1.17 (m, 26H, CH_2_), 0.87 (t, *J* = 7.0 Hz, 3H, CH_3_); ^13^C NMR (100 MHz, CDCl_3_): δ 191.9 (CO), 162.9 (CO), 140.9 (ArC), 139.1 (ArC), 135.8 (ArC), 134.9 (ArCH), 133.6 (ArCH), 129.1 (ArCH), 127.9 (ArCH), 127.0 (ArCH), 119.7 (ArC), 118.6 (ArCH), 58.4 (CH_2_), 52.7 (CH_2_), 45.1 (CH_3_), 39.6 (CH_2_), 32.1 (CH_2_), 29.8 (CH_2_), 29.8 (CH_2_), 29.8 (CH_2_), 29.8 (CH_2_), 29.7 (CH_2_), 29.6 (CH_2_), 29.5 (CH_2_), 29.4 (CH_2_), 29.1 (CH_2_), 28.3 (CH_2_), 25.2 (CH_2_), 23.5 (CH_2_), 22.8 (CH_2_), 14.3 (CH_3_); IR (ATR): *ν*_max_ 3346, 3181, 2918, 2850, 2758, 2342, 1631, 1581, 1511, 1486, 1395, 1344, 1264, 1200, 1138, 1098, 1034, 925, 892, 855, 822, 761, 684 cm^−1^; HRMS (+ESI): Found *m*/*z* 614.3986 [M+H]^+^, C_35_H_56_N_3_O_4_S required 614.3986.

**General synthetic procedure C for tertiary ammonium chloride salts**

4 M HCl in dioxane (5.0 equivalents) was added to a stirring solution of glyoxamide derivative (1.0 equivalent) in diethyl ether (5 mL). The resulting reaction mixture was stirred at room temperature for 20 min. After the completion of the reaction, the reaction solvent was removed under reduced pressure, and the residue was washed three times with diethyl ether and freeze-dried to afford the product.

##### *N*,*N*-Dimethyl-3-(2-(4-(octylsulfonamido)-[1,1′:4′,1″-terphenyl]-3-yl)-2-oxoacetamido)propan-1-aminium chloride (**13d**)

The titled compound was synthesised from *N*-(3-(dimethylamino)propyl)-2-(4-(octylsulfonamido)-[1,1′:4′,1″-terphenyl]-3-yl)-2-oxoacetamide **10d** (24 mg, 0.042 mmol) and 4 M HCl/dioxane (58 μL, 0.23 mmol) following **general synthetic procedure C**. The product was obtained as a yellow sticky solid (22 mg, 88%); ^1^H NMR (600 MHz, DMSO-*d*_6_): δ 10.28 (bs, 2H, NH), 9.04 (t, *J* = 6.0 Hz, 1H, NH), 8.09–8.03 (m, 2H, ArH), 7.83–7.79 (m, 2H, ArH), 7.78–7.75 (m, 2H, ArH), 7.75–7.71 (m, 2H, ArH), 7.66–7.61 (m, 1H, ArH), 7.52–7.47 (m, 2H, ArH), 7.42–7.37 (m, 1H, ArH), 3.26–3.21 (m, 2H, CH_2_), 3.13–3.08 (m, 2H, CH_2_), 2.74 (s, 6H, CH_3_), 3.12–3.04 (m, 2H, CH_2_), 1.99–1.91 (m, 2H, CH_2_), 1.72–1.64 (m, 2H, CH_2_), 1.37–1.30 (m, 2H, CH_2_), 1.27–1.15 (m, 8H, CH_2_), 0.83 (t, *J* = 7.2 Hz, 3H, CH_3_); ^13^C NMR (150 MHz, DMSO-*d*_6_): δ 192.1 (CO), 163.7 (CO), 139.6 (ArC), 139.4 (ArC), 138.0 (ArC), 137.2 (ArC), 135.3 (ArC), 132.7 (ArCH), 130.1 (ArCH), 129.0 (ArCH), 127.7 (ArCH), 127.4 (ArCH), 127.0 (ArCH), 126.6 (ArCH), 125.8 (ArC), 122.4 (ArCH), 54.5 (CH_2_), 51.4 (CH_2_), 42.1 (CH_3_), 36.0 (CH_2_), 31.1 (CH_2_), 28.4 (CH_2_), 28.3 (CH_2_), 28.3 (CH_2_), 23.9 (CH_2_), 22.9 (CH_2_), 22.0 (CH_2_), 13.9 (CH_3_); IR (ATR): *ν*_max_ 3366, 3030, 2925, 2854, 2697, 2360, 1647, 1527, 1481, 1395, 1332, 1272, 1196, 1142, 1076, 1006, 975, 917, 828, 764, 728, 696, 673 cm^−1^; HRMS (+ESI): Found *m*/*z* 578.3049 [M+H]^+^, C_33_H_44_N_3_O_4_S required 578.3047.

##### 3-(2-(2-(Dodecylsulfonamido)phenyl)-2-oxoacetamido)-*N*,*N*-dimethylpropan-1-aminium chloride (**14a**)

The titled compound was synthesised from *N*-(3-(dimethylamino)propyl)-2-(2-(dodecylsulfonamido) phenyl)-2-oxoacetamide **11a** (30 mg, 0.062 mmol) and 4 M HCl/dioxane (0.10 mL, 0.40 mmol) following **general synthetic procedure C**. The product was obtained as a pale yellow sticky solid (25 mg, 76%); ^1^H NMR (600 MHz, DMSO-*d*_6_): δ 10.23 (bs, 2H, NH), 8.99 (t, *J* = 5.9 Hz, 1H, NH), 7.78 (dd, *J* = 7.8, 1.5 Hz, 1H, ArH), 7.72–7.68 (m, 1H, ArH), 7.53 (d, *J* = 8.2 Hz, 1H, ArH), 7.33–7.30 (m, 1H, ArH), 3.30 (q, *J* = 6.5 Hz, 2H, CH_2_), 3.23–3.19 (m, 2H, CH_2_), 3.11–3.06 (m, 2H, CH_2_), 2.74 (s, 6H, CH_3_), 1.96–1.89 (m, 2H, CH_2_), 1.68–1.60 (m, 2H, CH_2_), 1.35–1.15 (m, 18H, CH_2_), 0.85 (t, *J* = 7.1 Hz, 3H, CH_3_); ^13^C NMR (150 MHz, DMSO-*d*_6_): δ 192.9 (CO), 164.2 (CO), 139.1 (ArC), 135.3 (ArCH), 132.9 (ArCH), 124.1 (ArCH), 124.0 (ArC), 121.0 (ArCH), 54.5 (CH_2_), 51.3 (CH_2_), 42.1 (CH_3_), 35.9 (CH_2_), 31.3 (CH_2_), 29.0 (CH_2_), 28.9 (CH_2_), 28.7 (CH_2_), 28.7 (CH_2_), 28.4 (CH_2_), 27.2 (CH_2_), 23.9 (CH_2_), 22.9 (CH_2_), 22.1 (CH_2_), 14.0 (CH_3_); IR (ATR): *ν*_max_ 3380, 3260, 3082, 2917, 2851, 2689, 2093, 1663, 1527, 1456, 1420, 1328, 1263, 1217, 1143, 1065, 989, 925, 869, 798, 774 cm^−1^; HRMS (+ESI): Found *m*/*z* 482.3046 [M+H]^+^, C_25_H_44_N_3_O_4_S required 482.3047.

##### 3-(2-(5-Bromo-2-(dodecylsulfonamido)phenyl)-2-oxoacetamido)-*N*,*N*-dimethylpropan-1-aminium chloride (**14b**)

The titled compound was synthesised from 2-(5-bromo-2-(dodecylsulfonamido)phenyl)-*N*-(3-(dimethylamino)propyl)-2-oxoacetamide **11b** (30 mg, 0.054 mmol) and 4 M HCl/dioxane (0.10 mL, 0.40 mmol) following **general synthetic procedure C**. The product was obtained as a yellow sticky solid (22 mg, 67%); ^1^H NMR (600 MHz, DMSO-*d*_6_): δ 10.10 (bs, 2H, NH), 8.96 (t, *J* = 6.0 Hz, 1H, NH), 7.88–7.83 (m, 2H, ArH), 7.43 (d, *J* = 8.6 Hz, 1H, ArH), 3.29 (q, *J* = 6.4 Hz, 2H, CH_2_), 3.17–3.05 (m, 4H, CH_2_), 2.74 (s, 6H, CH_3_), 1.94–1.88 (m, 2H, CH_2_), 1.66–1.59 (m, 2H, CH_2_), 1.34–1.16 (m, 18H, CH_2_), 0.85 (t, *J* = 7.1 Hz, 3H, CH_3_); ^13^C NMR (150 MHz, DMSO-*d*_6_): δ 190.0 (CO), 162.9 (CO), 137.2 (ArC), 136.8 (ArCH), 133.9 (ArCH), 129.1 (ArC), 124.9 (ArCH), 116.7 (ArC), 54.5 (CH_2_), 51.3 (CH_2_), 42.1 (CH_3_), 36.0 (CH_2_), 31.3 (CH_2_), 29.0 (CH_2_), 28.9 (CH_2_), 28.7 (CH_2_), 28.7 (CH_2_), 28.4 (CH_2_), 27.3 (CH_2_), 23.8 (CH_2_), 22.8 (CH_2_), 22.1 (CH_2_), 14.0 (CH_3_); IR (ATR): *ν*_max_ 3382, 2921, 2851, 2698, 2361, 1648, 1529, 1478, 1389, 1331, 1198, 1147, 1073, 975, 916, 824, 719, 655 cm^−1^; HRMS (+ESI): Found *m*/*z* 560.2150 [M+H]^+^, C_25_H_43_BrN_3_O_4_S required 560.2152.

##### 3-(2-(4-(Dodecylsulfonamido)-[1,1′-biphenyl]-3-yl)-2-oxoacetamido)-*N*,*N*-dimethylpropan-1-aminium chloride (**14c**)

The titled compound was synthesised from *N*-(3-(dimethylamino)propyl)-2-(4-(dodecylsulfonamido)-[1,1′-biphenyl]-3-yl)-2-oxoacetamide **11c** (30 mg, 0.054 mmol) and 4 M HCl/dioxane (0.10 mL, 0.40 mmol) following **general synthetic procedure C**. The product was obtained as a yellow sticky solid (19 mg, 60%); ^1^H NMR (600 MHz, DMSO-*d*_6_): δ 10.19 (bs, 2H, NH), 9.02 (t, *J* = 6.1 Hz, 1H, NH), 8.02–7.97 (m, 2H, ArH), 7.68–7.63 (m, 2H, ArH), 7.63–7.59 (m, 1H, ArH), 7.53–7.47 (m, 2H, ArH), 7.43–7.38 (m, 1H, ArH), 3.35–3.29 (m, 2H, CH_2_), 3.24–3.18 (m, 2H, CH_2_), 3.13–3.07 (m, 2H, CH_2_), 2.74 (s, 6H, CH_3_), 1.97–1.89 (m, 2H, CH_2_), 1.71–1.62 (m, 2H, CH_2_), 1.37–1.14 (m, 18H, CH_2_), 0.84 (t, *J* = 7.2 Hz, 3H, CH_3_); ^13^C NMR (150 MHz, DMSO-*d*_6_): δ 192.1 (CO), 163.7 (CO), 138.2 (ArC), 137.9 (ArC), 136.0 (ArC), 132.9 (ArCH), 130.2 (ArCH), 129.2 (ArCH), 127.9 (ArCH), 126.5 (ArCH), 125.8 (ArC), 122.4 (ArCH), 54.5 (CH_2_), 51.3 (CH_2_), 42.1 (CH_3_), 36.0 (CH_2_), 31.3 (CH_2_), 29.0 (CH_2_), 28.9 (CH_2_), 28.7 (CH_2_), 28.7 (CH_2_), 28.3 (CH_2_), 27.3 (CH_2_), 23.9 (CH_2_), 22.9 (CH_2_), 22.1 (CH_2_), 14.0 (CH_3_); IR (ATR): *ν*_max_ 3341, 2921, 2852, 2696, 2360, 1661, 1631, 1582, 1512, 1487, 1467, 1396, 1345, 1268, 1201, 1139, 1075, 1003, 973, 925, 889, 853, 826, 761, 721, 683 cm^−1^; HRMS (+ESI): Found *m*/*z* 558.3363 [M+H]^+^, C_31_H_48_N_3_O_4_S required 558.3360.

##### 3-(2-(4-(Dodecylsulfonamido)-[1,1′:4′,1″-terphenyl]-3-yl)-2-oxoacetamido)-*N*,*N*-dimethylpropan-1-aminium chloride (**14d**)

The titled compound was synthesised from *N*-(3-(dimethylamino)propyl)-2-(4-(dodecylsulfonamido)-[1,1′:4′,1″-terphenyl]-3-yl)-2-oxoacetamide **11d** (37 mg, 0.058 mmol) and 4 M HCl/dioxane (0.10 mL, 0.40 mmol) following **general synthetic procedure C**. The product was obtained as a white sticky solid (19 mg, 48%); ^1^H NMR (600 MHz, DMSO-*d*_6_): δ 10.19 (bs, 2H, ArH), 9.03 (t, *J* = 5.9 Hz, 1H, NH), 8.09–8.03 (m, 2H, ArH), 7.83–7.79 (m, 2H, ArH), 7.78–7.75 (m, 2H, ArH), 7.75–7.70 (m, 2H, ArH), 7.65–7.61 (m, 1H, ArH), 7.52–7.47 (m, 2H, ArH), 7.42–7.37 (m, 1H, ArH), 3.36–3.30 (m, 2H, CH_2_), 3.25–3.21 (m, 2H, CH_2_), 3.13–3.07 (m, 2H, CH_2_), 2.74 (s, 6H, CH_3_), 1.98–1.90 (m, 2H, CH_2_), 1.71–1.63 (m, 2H, CH_2_), 1.37–1.30 (m, 2H, CH_2_), 1.27–1.15 (m, 16H, CH_2_), 0.82 (t, *J* = 7.2 Hz, 3H, CH_3_); ^13^C NMR (150 MHz, DMSO-*d*_6_): δ 192.1 (CO), 163.7 (CO), 139.6 (ArC), 139.4 (ArC), 138.0 (ArC), 137.2 (ArC), 135.4 (ArC), 132.7 (ArCH), 130.1 (ArCH), 129.0 (ArCH), 127.7 (ArCH), 127.4 (ArCH), 127.0 (ArCH), 126.6 (ArCH), 125.8 (ArC), 122.4 (ArCH), 54.5 (CH_2_), 51.3 (CH_2_), 42.1 (CH_3_), 36.0 (CH_2_), 31.3 (CH_2_), 29.0 (CH_2_), 28.9 (CH_2_), 28.7 (CH_2_), 28.7 (CH_2_), 28.4 (CH_2_), 27.3 (CH_2_), 24.0 (CH_2_), 22.9 (CH_2_), 22.1 (CH_2_), 14.0 (CH_3_); IR (ATR): *ν*_max_ 3353, 3261, 3030, 2920, 2850, 2689, 2477, 2113, 1920, 1650, 1525, 1481, 1400, 1333, 1272, 1195, 1144, 1073, 1005, 974, 917, 826, 764, 719, 695 cm^−1^; HRMS (+ESI): Found *m*/*z* 634.3672 [M+H]^+^, C_37_H_52_N_3_O_4_S required 634.3673.

##### 3-(2-(2-(Hexadecylsulfonamido)phenyl)-2-oxoacetamido)-*N*,*N*-dimethylpropan-1-aminium chloride (**15a**)

The titled compound was synthesised from *N*-(3-(dimethylamino)propyl)-2-(2-(hexadecylsulfonamido) phenyl)-2-oxoacetamide **12a** (30 mg, 0.056 mmol) and 4 M HCl/dioxane (0.10 mL, 0.40 mmol) following **general synthetic procedure C**. The product was obtained as a white sticky solid (32 mg, 99%); ^1^H NMR (600 MHz, DMSO-*d*_6_): δ 10.20 (bs, 2H, NH), 8.99 (t, *J* = 5.9 Hz, 1H, NH), 7.77 (dd, *J* = 7.9, 1.5 Hz, 1H, ArH), 7.72–7.67 (m, 1H, ArH), 7.53 (dd, *J* = 8.3, 0.7 Hz, 1H, ArH), 7.33–7.30 (m, 1H, ArH), 3.30 (q, *J* = 6.4 Hz, 2H, CH_2_), 3.23–3.18 (m, 2H, CH_2_), 3.10–3.05 (m, 2H, CH_2_), 2.74 (s, 6H, CH_3_), 1.96–1.88 (m, 2H, CH_2_), 1.68–1.60 (m, 2H, CH_2_), 1.35–1.14 (m, 26H, CH_2_), 0.85 (t, *J* = 7.1 Hz, 3H, CH_3_); ^13^C NMR (150 MHz, DMSO-*d*_6_): δ 192.9 (CO), 164.2 (CO), 139.1 (ArC), 135.3 (ArCH), 132.9 (ArCH), 124.1 (ArCH), 124.0 (ArC), 121.0 (ArCH), 54.5 (CH_2_), 51.3 (CH_2_), 42.2 (CH_3_), 35.9 (CH_2_), 31.3 (CH_2_), 29.1 (CH_2_), 29.1 (CH_2_), 29.0 (CH_2_), 29.0 (CH_2_), 28.9 (CH_2_), 28.7 (CH_2_), 28.7 (CH_2_), 28.4 (CH_2_), 27.3 (CH_2_), 23.9 (CH_2_), 22.9 (CH_2_), 22.1 (CH_2_), 14.0 (CH_3_); IR (ATR): *ν*_max_ 3312, 3297, 3033, 2916, 2848, 2692, 2479, 2108, 1649, 1573, 1534, 1491, 1465, 1399, 1333, 1211, 1147, 1067, 973, 922, 819, 757, 721, 673 cm^−1^; HRMS (+ESI): Found *m*/*z* 538.3672 [M+H]^+^, C_29_H_52_N_3_O_4_S required 538.3673.

##### 3-(2-(5-Bromo-2-(hexadecylsulfonamido)phenyl)-2-oxoacetamido)-*N*,*N*-dimethylpropan-1-aminium chloride (**15b**)

The titled compound was synthesised from 2-(5-bromo-2-(hexadecylsulfonamido)phenyl)-*N*-(3-(dimethylamino)propyl)-2-oxoacetamide **12b** (32 mg, 0.052 mmol) and 4 M HCl/dioxane (0.10 mL, 0.40 mmol) following **general synthetic procedure C**. The product was obtained as a pale yellow sticky solid (26 mg, 77%); ^1^H NMR (600 MHz, DMSO-*d*_6_): δ 10.28 (bs, 1H, NH), 10.11 (bs, 1H, NH), 8.97 (t, *J* = 6.2 Hz, 1H, NH), 7.89–7.82 (m, 2H, ArH), 7.44 (d, *J* = 8.8 Hz, 1H, ArH), 3.29 (q, *J* = 6.5 Hz, 2H, CH_2_), 3.16–3.06 (m, 4H, CH_2_), 2.74 (s, 6H, CH_3_), 1.95–1.87 (m, 2H, CH_2_), 1.68–1.58 (m, 2H, CH_2_), 1.35–1.15 (m, 26H, CH_2_), 0.85 (t, *J* = 7.1 Hz, 3H, CH_3_); ^13^C NMR (150 MHz, DMSO-*d*_6_): δ 190.0 (CO), 162.9 (CO), 137.3 (ArC), 136.8 (ArCH), 133.9 (ArCH), 129.0 (ArC), 124.9 (ArCH), 116.7 (ArC), 54.5 (CH_2_), 51.3 (CH_2_), 42.1 (CH_3_), 36.0 (CH_2_), 31.3 (CH_2_), 29.0 (CH_2_), 29.0 (CH_2_), 29.0 (CH_2_), 29.0 (CH_2_), 28.9 (CH_2_), 28.7 (CH_2_), 28.7 (CH_2_), 28.4 (CH_2_), 27.3 (CH_2_), 23.8 (CH_2_), 22.8 (CH_2_), 22.1 (CH_2_), 14.0 (CH_3_); IR (ATR): *ν*_max_ 3378, 3251, 3067, 2916, 2848, 2710, 2104, 1741, 1644, 1595, 1526, 1461, 1372, 1337, 1289, 1234, 1193, 1142, 975, 936, 843, 786, 720 cm^−1^; HRMS (+ESI): Found *m*/*z* 616.2778 [M+H]^+^, C_29_H_51_BrN_3_O_4_S required 616.2778.

##### 3-(2-(4-(Hexadecylsulfonamido)-[1,1′-biphenyl]-3-yl)-2-oxoacetamido)-*N*,*N*-dimethylpropan-1-aminium chloride (**15c**)

The titled compound was synthesised from *N*-(3-(dimethylamino)propyl)-2-(4-(hexadecylsulfonamido)-[1,1′-biphenyl]-3-yl)-2-oxoacetamide **12c** (31 mg, 0.051 mmol) and 4 M HCl/dioxane (0.10 mL, 0.40 mmol) following **general synthetic procedure C**. The product was obtained as a yellow sticky solid (26 mg, 79%); ^1^H NMR (600 MHz, DMSO-*d*_6_): δ 10.18 (bs, 2H, NH), 9.01 (t, *J* = 5.9 Hz, 1H, NH), 8.01–7.98 (m, 2H, ArH), 7.67–7.63 (m, 2H, ArH), 7.62–7.59 (m, 1H, ArH), 7.52–7.48 (m, 2H, ArH), 7.42–7.39 (m, 1H, ArH), 3.35–3.29 (m, 2H, CH_2_), 3.24–3.19 (m, 2H, CH_2_), 3.11–3.06 (m, 2H, CH_2_), 2.73 (s, 6H, CH_3_), 1.97–1.88 (m, 2H, CH_2_), 1.71–1.63 (m, 2H, CH_2_), 1.37–1.14 (m, 26H, CH_2_), 0.85 (t, *J* = 7.0 Hz, 3H, CH_3_); ^13^C NMR (150 MHz, DMSO-*d*_6_): δ 192.1 (CO), 163.7 (CO), 138.2 (ArC), 137.9 (ArC), 135.9 (ArC), 132.9 (ArCH), 130.2 (ArCH), 129.2 (ArCH), 127.9 (ArCH), 126.5 (ArCH), 125.8 (ArC), 122.3 (ArCH), 54.5 (CH_2_), 51.3 (CH_2_), 42.1 (CH_3_), 36.0 (CH_2_), 31.3 (CH_2_), 29.0 (CH_2_), 29.0 (CH_2_), 29.0 (CH_2_), 29.0 (CH_2_), 28.9 (CH_2_), 28.7 (CH_2_), 28.7 (CH_2_), 28.4 (CH_2_), 27.3 (CH_2_), 24.0 (CH_2_), 22.9 (CH_2_), 22.1 (CH_2_), 13.9 (CH_3_); IR (ATR): *ν*_max_ 3345, 3186, 2918, 2850, 2643, 2477, 2110, 1631, 1581, 1511, 1486, 1396, 1345, 1268, 1201, 1139, 1088, 973, 926, 826, 761, 685 cm^−1^; HRMS (+ESI): Found *m*/*z* 614.3986 [M+H]^+^, C_35_H_56_N_3_O_4_S required 614.3986.

**General synthetic procedure D for quaternary ammonium iodide salts**

Iodomethane (2.5 equivalents) was added to a stirring solution of glyoxamide derivative (1.0 equivalent) in tetrahydrofuran (3 mL). The resulting reaction mixture was stirred at room temperature for 24 h. After the completion of the reaction, the reaction solvent was removed under reduced pressure, and the residue was washed three times with diethyl ether and freeze-dried to afford the product.

##### *N*,*N*,*N*-Trimethyl-3-(2-(4-(octylsulfonamido)-[1,1′:4′,1″-terphenyl]-3-yl)-2-oxoacetamido)propan-1-aminium iodide (**16d**)

The titled compound was synthesised from *N*-(3-(dimethylamino)propyl)-2-(4-(octylsulfonamido)-[1,1′:4′,1″-terphenyl]-3-yl)-2-oxoacetamide **10d** (21 mg, 0.036 mmol) and iodomethane (6.0 μL, 0.096 mmol) following **general synthetic procedure D**. The product was obtained as a yellow sticky solid (24 mg, 91%); ^1^H NMR (600 MHz, DMSO-*d*_6_): δ 10.11 (bs, 1H, ArH), 8.98 (bs, 1H, NH), 8.05 (d, *J* = 1.7 Hz, 2H, ArH), 7.82–7.75 (m, 4H, ArH), 7.75–7.71 (m, 2H, ArH), 7.62–7.57 (m, 1H, ArH), 7.52–7.47 (m, 2H, ArH), 7.42–7.37 (m, 1H, ArH), 3.41–3.29 (m, 4H, CH_2_), 3.19 (t, *J* = 7.8 Hz, 2H, CH_2_), 3.08 (s, 9H, CH_3_), 2.04–1.96 (m, 2H, CH_2_), 1.72–1.64 (m, 2H, CH_2_), 1.38–1.29 (m, 2H, CH_2_), 1.27–1.15 (m, 8H, CH_2_), 0.83 (t, *J* = 7.2 Hz, 3H, CH_3_); ^13^C NMR (150 MHz, DMSO-*d*_6_): δ 191.9 (CO), 163.7 (CO), 139.6 (ArC), 139.4 (ArC), 137.6 (ArC), 137.2 (ArC), 135.6 (ArC), 132.5 (ArCH), 129.9 (ArCH), 129.0 (ArCH), 127.7 (ArCH), 127.4 (ArCH), 127.0 (ArCH), 126.9 (ArC), 126.6 (ArCH), 122.9 (ArCH), 63.5 (CH_2_), 52.3 (CH_3_), 51.2 (CH_2_), 35.9 (CH_2_), 31.1 (CH_2_), 28.4 (CH_2_), 28.3 (CH_2_), 27.3 (CH_2_), 22.9 (CH_2_), 22.6 (CH_2_), 22.0 (CH_2_), 13.9 (CH_3_); IR (ATR): *ν*_max_ 3382, 2924, 2854, 2360, 1979, 1670, 1527, 1481, 1396, 1332, 1259, 1195, 1141, 1075, 1006, 914, 883, 829, 765, 729, 697, 673 cm^−1^; HRMS (+ESI): Found *m*/*z* 592.3206 [M]^+^, C_34_H_46_N_3_O_4_S required 592.3204.

##### 3-(2-(2-(Dodecylsulfonamido)phenyl)-2-oxoacetamido)-*N*,*N*,*N*-trimethylpropan-1-aminium iodide (**17a**)

The titled compound was synthesised from *N*-(3-(dimethylamino)propyl)-2-(2-(dodecylsulfonamido) phenyl)-2-oxoacetamide **11a** (30 mg, 0.062 mmol) and iodomethane (10.0 μL, 0.16 mmol) following **general synthetic procedure D**. The product was obtained as a yellow sticky solid (25 mg, 64%); ^1^H NMR (600 MHz, DMSO-*d*_6_): δ 10.14 (bs, 1H, NH), 8.95 (t, *J* = 5.9 Hz, 1H, NH), 7.77 (dd, *J* = 7.9, 1.6 Hz, 1H, ArH), 7.73–7.68 (m, 1H, ArH), 7.50 (dd, *J* = 8.3, 0.7 Hz, 1H, ArH), 7.35–7.31 (m, 1H, ArH), 3.37–3.27 (m, 4H, CH_2_), 3.20–3.15 (m, 2H, CH_2_), 3.07 (s, 9H, CH_3_), 2.02–1.93 (m, 2H, CH_2_), 1.68–1.60 (m, 2H, CH_2_), 1.36–1.14 (m, 18H, CH_2_), 0.85 (t, *J* = 7.2 Hz, 3H, CH_3_); ^13^C NMR (150 MHz, DMSO-*d*_6_): δ 192.5 (CO), 164.1 (CO), 138.7 (ArC), 135.1 (ArCH), 132.6 (ArCH), 125.1 (ArC), 124.4 (ArCH), 121.6 (ArCH), 63.4 (CH_2_), 52.3 (CH_3_), 51.2 (CH_2_), 35.8 (CH_2_), 31.3 (CH_2_), 29.0 (CH_2_), 28.9 (CH_2_), 28.7 (CH_2_), 28.7 (CH_2_), 28.4 (CH_2_), 27.3 (CH_2_), 22.9 (CH_2_), 22.6 (CH_2_), 22.1 (CH_2_), 14.0 (CH_3_); IR (ATR): *ν*_max_ 3510, 3448, 3258, 3175, 3038, 2918, 2851, 1666, 1602, 1524, 1453, 1327, 1261, 1214, 1141, 1107, 984, 913, 883, 772 cm^−1^; HRMS (+ESI): Found *m*/*z* 496.3203 [M]^+^, C_26_H_46_N_3_O_4_S required 496.3204.

##### 3-(2-(5-Bromo-2-(dodecylsulfonamido)phenyl)-2-oxoacetamido)-*N*,*N*,*N*-trimethylpropan-1-aminium iodide (**17b**)

The titled compound was synthesised from 2-(5-bromo-2-(dodecylsulfonamido)phenyl)-*N*-(3-(dimethylamino)propyl)-2-oxoacetamide **11b** (34 mg, 0.061 mmol) and iodomethane (10.0 μL, 0.16 mmol) following **general synthetic procedure D**. The product was obtained as a yellow sticky solid (34 mg, 80%); ^1^H NMR (600 MHz, DMSO-*d*_6_): δ 10.00 (s, 1H, NH), 8.95 (t, *J* = 6.0 Hz, 1H, NH), 7.90–7.82 (m, 2H, ArH), 7.42–7.38 (m, 1H, ArH), 3.40–3.27 (m, 4H, CH_2_), 3.13–3.03 (m, 2H, CH_2_), 3.06 (s, 9H, CH_3_), 2.02–1.93 (m, 2H, CH_2_), 1.68–1.58 (m, 2H, CH_2_), 1.37–1.14 (m, 18H, CH_2_), 0.85 (t, *J* = 7.1 Hz, 3H, CH_3_); ^13^C NMR (150 MHz, DMSO-*d*_6_): δ 189.8 (CO), 162.8 (CO), 136.9 (ArC), 136.7 (ArCH), 133.9 (ArCH), 129.9 (ArC), 125.4 (ArCH), 117.1 (ArC), 63.5 (CH_2_), 52.3 (CH_3_), 51.2 (CH_2_), 35.9 (CH_2_), 31.3 (CH_2_), 29.0 (CH_2_), 28.9 (CH_2_), 28.7 (CH_2_), 28.7 (CH_2_), 28.4 (CH_2_), 27.3 (CH_2_), 22.8 (CH_2_), 22.5 (CH_2_), 22.1 (CH_2_), 14.0 (CH_3_); IR (ATR): *ν*_max_ 3808, 3650, 3289, 2920, 2850, 2360, 1979, 1677, 1649, 1561, 1541, 1480, 1391, 1340, 1291, 1274, 1260, 1228, 1203, 1152, 1077, 967, 922, 871, 829, 721, 698, 655 cm^−1^; HRMS (+ESI): Found *m*/*z* 574.2318 [M]^+^, C_26_H_45_BrN_3_O_4_S required 574.2309.

##### 3-(2-(4-(Dodecylsulfonamido)-[1,1′-biphenyl]-3-yl)-2-oxoacetamido)-*N*,*N*,*N*-trimethylpropan-1-aminium iodide (**17c**)

The titled compound was synthesised from *N*-(3-(dimethylamino)propyl)-2-(4-(dodecylsulfonamido)-[1,1′-biphenyl]-3-yl)-2-oxoacetamide **11c** (34 mg, 0.061 mmol) and iodomethane (10.0 μL, 0.16 mmol) following **general synthetic procedure D**. The product was obtained as a yellow sticky solid (36 mg, 85%); ^1^H NMR (600 MHz, DMSO-*d*_6_): δ 10.10 (bs, 1H, NH), 8.98 (t, *J* = 6.0 Hz, 1H, NH), 8.04–7.94 (m, 2H, ArH), 7.68–7.64 (m, 2H, ArH), 7.60–7.56 (m, 1H, ArH), 7.52–7.46 (m, 2H, ArH), 7.43–7.38 (m, 1H, ArH), 3.41–3.28 (m, 4H, CH_2_), 3.23–3.14 (m, 2H, CH_2_), 3.07 (s, 9H, CH_3_), 2.04–1.93 (m, 2H, CH_2_), 1.71–1.61 (m, 2H, CH_2_), 1.39–1.14 (m, 18H, CH_2_), 0.84 (t, *J* = 7.2 Hz, 3H, CH_3_); ^13^C NMR (150 MHz, DMSO-*d*_6_): δ 191.8 (CO), 163.7 (CO), 138.3 (ArC), 137.5 (ArC), 136.3 (ArC), 132.7 (ArCH), 130.1 (ArCH), 129.2 (ArCH), 127.9 (ArCH), 126.8 (ArC), 126.5 (ArCH), 122.9 (ArCH), 63.5 (CH_2_), 52.3 (CH_3_), 51.2 (CH_2_), 35.9 (CH_2_), 31.3 (CH_2_), 29.0 (CH_2_), 28.9 (CH_2_), 28.7 (CH_2_), 28.7 (CH_2_), 28.4 (CH_2_), 27.3 (CH_2_), 22.9 (CH_2_), 22.6 (CH_2_), 22.1 (CH_2_), 13.9 (CH_3_); IR (ATR): *ν*_max_ 3410, 2922, 2852, 2359, 1979, 1670, 1648, 1582, 1509, 1483, 1395, 1330, 1264, 1195, 1140, 1075, 915, 841, 761, 698, 681 cm^−1^; HRMS (+ESI): Found *m*/*z* 572.3519 [M]^+^, C_32_H_50_N_3_O_4_S required 572.3517.

##### 3-(2-(4-(Dodecylsulfonamido)-[1,1′:4′,1″-terphenyl]-3-yl)-2-oxoacetamido)-*N*,*N*,*N*-trimethylpropan-1-aminium iodide (**17d**)

The titled compound was synthesised from *N*-(3-(dimethylamino)propyl)-2-(4-(dodecylsulfonamido)-[1,1′:4′,1″-terphenyl]-3-yl)-2-oxoacetamide **11d** (32 mg, 0.051 mmol) and iodomethane (10.0 μL, 0.16 mmol) following **general synthetic procedure D**. The product was obtained as a white sticky solid (16 mg, 42%); ^1^H NMR (600 MHz, DMSO-*d*_6_): δ 10.12 (bs, 1H, ArH), 9.00 (t, *J* = 5.9 Hz, 1H, NH), 8.09–8.03 (m, 2H, ArH), 7.82–7.75 (m, 2H, ArH), 7.74–7.71 (m, 2H, ArH), 7.62–7.58 (m, 1H, ArH), 7.52–7.61 (m, 2H, ArH), 7.42–7.37 (m, 2H, ArH), 7.42–7.37 (m, 1H, ArH), 3.40–3.30 (m, 4H, CH_2_), 3.23–3.17 (m, 2H, CH_2_), 3.07 (s, 9H, CH_3_), 2.04–1.96 (m, 2H, CH_2_), 1.71–1.63 (m, 2H, CH_2_), 1.37–1.29 (m, 2H, CH_2_), 1.26–1.14 (m, 16H, CH_2_), 0.83 (t, *J* = 7.2 Hz, 3H, CH_3_); ^13^C NMR (150 MHz, DMSO-*d*_6_): δ 191.8 (CO), 163.7 (CO), 139.6 (ArC), 139.4 (ArC), 137.6 (ArC), 137.2 (ArC), 135.7 (ArC), 132.6 (ArCH), 130.0 (ArCH), 129.0 (ArCH), 127.7 (ArCH), 127.4 (ArCH), 127.0 (ArCH), 126.8 (ArC), 126.6 (ArCH), 122.9 (ArCH), 63.5 (CH_2_), 52.3 (CH_3_), 51.2 (CH_2_), 35.9 (CH_2_), 31.3 (CH_2_), 29.0 (CH_2_), 28.9 (CH_2_), 28.7 (CH_2_), 28.7 (CH_2_), 28.4 (CH_2_), 27.3 (CH_2_), 22.9 (CH_2_), 22.6 (CH_2_), 22.1 (CH_2_), 13.9 (CH_3_); IR (ATR): *ν*_max_ 3751, 3376, 3264, 3029, 2920, 2850, 2723, 2485, 2295, 2103, 2075, 1650, 1524, 1480, 1398, 1333, 1272, 1194, 1141, 1073, 1005, 914, 827, 764, 718, 696 cm^−1^; HRMS (+ESI): Found *m*/*z* 648.3829 [M]^+^, C_38_H_54_N_3_O_4_S required 648.3830.

##### 3-(2-(2-(Hexadecylsulfonamido)phenyl)-2-oxoacetamido)-*N*,*N*,*N*-trimethylpropan-1-aminium iodide (**18a**)

The titled compound was synthesised from *N*-(3-(dimethylamino)propyl)-2-(2-(hexadecylsulfonamido) phenyl)-2-oxoacetamide **12a** (30 mg, 0.056 mmol) and iodomethane (9.0 μL, 0.15 mmol) following **general synthetic procedure D**. The product was obtained as a white sticky solid (33 mg, 87%); ^1^H NMR (400 MHz, DMSO-*d*_6_): δ 10.13 (bs, 1H, NH), 8.95 (t, *J* = 5.8 Hz, 1H, NH), 7.76 (dd, *J* = 7.9, 1.3 Hz, 1H, ArH), 7.73–7.66 (m, 1H, ArH), 7.49 (d, *J* = 8.0 Hz, 1H, ArH), 7.33 (t, *J* = 7.5 Hz, 1H, ArH), 3.39–3.26 (m, 4H, CH_2_), 3.20–3.12 (m, 2H, CH_2_), 3.06 (s, 9H, CH_3_), 2.03–1.91 (m, 2H, CH_2_), 1.70–1.57 (m, 2H, CH_2_), 1.37–1.12 (m, 26H, CH_2_), 0.84 (t, *J* = 7.0 Hz, 3H, CH_3_); ^13^C NMR (150 MHz, DMSO-*d*_6_): δ 192.6 (CO), 164.2 (CO), 138.8 (ArC), 135.1 (ArCH), 132.7 (ArCH), 125.2 (ArC), 124.4 (ArCH), 121.6 (ArCH), 63.5 (CH_2_), 52.4 (CH_3_), 51.2 (CH_2_), 35.9 (CH_2_), 31.3 (CH_2_), 29.1 (CH_2_), 29.1 (CH_2_), 29.0 (CH_2_), 29.0 (CH_2_), 28.9 (CH_2_), 28.7 (CH_2_), 28.7 (CH_2_), 28.4 (CH_2_), 27.3 (CH_2_), 23.9 (CH_2_), 22.6 (CH_2_), 22.1 (CH_2_), 14.0 (CH_3_); IR (ATR): *ν*_max_ 3448, 3253, 3234, 3009, 2916, 2848, 2284, 1663, 1527, 1490, 1452, 1401, 1329, 1262, 1214, 1145, 1066, 919, 882, 800, 757, 721, 673 cm^−1^; HRMS (+ESI): Found *m*/*z* 552.3830 [M]^+^, C_30_H_54_N_3_O_4_S required 552.3830.

##### 3-(2-(5-Bromo-2-(hexadecylsulfonamido)phenyl)-2-oxoacetamido)-*N*,*N*,*N*-trimethylpropan-1-aminium iodide (**18b**)

The titled compound was synthesised from 2-(5-bromo-2-(hexadecylsulfonamido)phenyl)-*N*-(3-(dimethylamino)propyl)-2-oxoacetamide **12b** (38 mg, 0.062 mmol) and iodomethane (10.0 μL, 0.16 mmol) following **general synthetic procedure D**. The product was obtained as a yellow sticky solid (31 mg, 65%); ^1^H NMR (600 MHz, DMSO-*d*_6_): δ 10.00 (bs, 1H, NH), 8.94 (t, *J* = 5.9 Hz, 1H, NH), 7.92–7.82 (m, 2H, ArH), 7.40 (dd, *J* = 8.1, 0.9 Hz, 1H, ArH), 3.40–3.27 (m, 4H, CH_2_), 3.14–3.02 (m, 2H, CH_2_), 3.06 (s, 9H, CH_3_), 2.03–1.91 (m, 2H, CH_2_), 1.69–1.56 (m, 2H, CH_2_), 1.38–1.07 (m, 26H, CH_2_), 0.85 (t, *J* = 7.1 Hz, 3H, CH_3_); ^13^C NMR (150 MHz, DMSO-*d*_6_): δ 189.8 (CO), 162.8 (CO), 136.7 (ArCH), 134.9 (ArC), 133.8 (ArCH), 129.8 (ArC), 125.3 (ArCH), 117.0 (ArC), 63.5 (CH_2_), 52.3 (CH_3_), 51.2 (CH_2_), 35.9 (CH_2_), 31.3 (CH_2_), 29.0 (CH_2_), 29.0 (CH_2_), 28.9 (CH_2_), 28.7 (CH_2_), 28.4 (CH_2_), 27.3 (CH_2_), 22.8 (CH_2_), 22.5 (CH_2_), 22.1 (CH_2_), 13.9 (CH_3_); IR (ATR): *ν*_max_ 3410, 3223, 3014, 2919, 2851, 2087, 1896, 1651, 1526, 1477, 1390, 1334, 1197, 1144, 918, 821, 767, 717 cm^−1^; HRMS (+ESI): Found *m*/*z* 630.2933 [M]^+^, C_30_H_53_BrN_3_O_4_S required 630.2935.

##### 3-(2-(4-(Hexadecylsulfonamido)-[1,1′-biphenyl]-3-yl)-2-oxoacetamido)-*N*,*N*,*N*-trimethylpropan-1-aminium iodide (**18c**)

The titled compound was synthesised from *N*-(3-(dimethylamino)propyl)-2-(4-(hexadecylsulfonamido)-[1,1′-biphenyl]-3-yl)-2-oxoacetamide **12c** (31 mg, 0.051 mmol) and iodomethane (10.0 μL, 0.16 mmol) following **general synthetic procedure D**. The product was obtained as a yellow sticky solid (21 mg, 53%); ^1^H NMR (600 MHz, DMSO-*d*_6_): δ 10.10 (bs, 1H, NH), 8.98 (bs, 1H, NH), 8.07–7.93 (m, 2H, ArH), 7.66 (dd, *J* = 8.5, 1.2 Hz, 2H, ArH), 7.60–7.55 (m, 1H, ArH), 7.52–7.47 (m, 2H, ArH), 7.40 (t, *J* = 7.4 Hz, 1H, ArH), 3.39–3.35 (m, 2H, CH_2_), 3.35–3.30 (m, 2H, CH_2_), 3.18 (t, *J* = 7.7 Hz, 2H, CH_2_), 3.07 (s, 9H, CH_3_), 2.02–1.95 (m, 2H, CH_2_), 1.70–1.63 (m, 2H, CH_2_), 1.37–1.14 (m, 26H, CH_2_), 0.85 (t, *J* = 7.1 Hz, 3H, CH_3_); ^13^C NMR (150 MHz, DMSO-*d*_6_): δ 191.8 (CO), 163.7 (CO), 138.3 (ArC), 137.5 (ArC), 136.3 (ArC), 132.7 (ArCH), 130.1 (ArCH), 129.1 (ArCH), 127.9 (ArCH), 126.8 (ArC), 126.5 (ArCH), 122.9 (ArCH), 63.5 (CH_2_), 52.3 (CH_3_), 51.2 (CH_2_), 35.9 (CH_2_), 31.3 (CH_2_), 29.0 (CH_2_), 29.0 (CH_2_), 28.9 (CH_2_), 28.7 (CH_2_), 28.4 (CH_2_), 27.3 (CH_2_), 22.9 (CH_2_), 22.6 (CH_2_), 22.1 (CH_2_), 13.9 (CH_3_); IR (ATR): *ν*_max_ 3317, 3189, 3009, 2919, 2851, 2299, 1907, 1632, 1511, 1486, 1395, 1343, 1266, 1200, 1139, 1088, 924, 848, 761, 683 cm^−1^; HRMS (+ESI): Found *m*/*z* 628.4142 [M]^+^, C_36_H_58_N_3_O_4_S required 628.4143.

##### 5-([1,1′-Biphenyl]-3-yl)indoline-2,3-dione (**19**)

The synthesis of the titled compound followed the procedure previously described [56]. A 2 M potassium carbonate solution (12.3 mL, 24.6 mmol) was degassed for 30 min and then added into a suspension of 5-bromoisatin (2.71 g, 11.98 mmol) and 3-biphenylboronic acid (2.70 g, 13.64 mmol) in a degassed (for 30 min) 1:1 ethanol/toluene solution (30 mL). The dark brown solution mixture was further degassed for 30 min, followed by the addition of tetrakis(triphenylphosphine)palladium(0) (145 mg, 0.125 mmol). The resulting reaction mixture was heated at reflux under an atmosphere of nitrogen for 18 h. The dark brown reaction mixture was concentrated in vacuo. Glacial acetic acid (30 mL) was added to the dark brown solid, and the resulting reaction mixture was heated at reflux for 20 min, followed by hot filtration of the red reaction mixture. The resulting red solution mixture was allowed to cool to room temperature, and the red precipitate formed was collected by vacuum filtration to give the crude product, which was redissolved in dimethylformamide. The solution mixture was then poured into a 1:1 ice-water mixture, and the red precipitate formed was collected by vacuum filtration to give the product as a red solid (2.00 g, 56%); mp 233.0–234.7 °C; ^1^H NMR (400 MHz, DMSO-*d*_6_): δ 11.14 (bs, 1H, NH), 8.02 (dd, *J* = 8.2, 2.0 Hz, 1H, ArH), 7.94–7.87 (m, 2H, ArH), 7.81–7.75 (m, 2H, ArH), 7.68–7.62 (m, 2H, ArH), 7.58–7.45 (m, 3H, ArH), 7.42–7.36 (m, 1H, ArH), 7.02 (dd, *J* = 8.2, 0.4 Hz, 1H, ArH); HRMS (+ESI): Found *m*/*z* 300.1019 [M+H]^+^, C_20_H_14_NO_2_ required 300.1019.

##### 5-([1,1′-Biphenyl]-3-yl)-1-(octylsulfonyl)indoline-2,3-dione (**20**)

Triethylamine (0.17 mL, 1.22 mmol) was added slowly to a stirring solution of 5-([1,1′-biphenyl]-3-yl)indoline-2,3-dione **19** (0.32 g, 1.06 mmol) in dichloromethane (20 mL) at 0 °C under nitrogen atmosphere. The reaction mixture was stirred at 0 °C for 20 min, followed by the dropwise addition of 1-octanesulfonyl chloride (0.21 mL, 1.07 mmol) with stirring. The reaction mixture was then stirred at room temperature for 3 h. After the completion of the reaction, the reaction mixture was concentrated in vacuo and washed with methanol to afford the product as a yellowish orange solid (0.27 g, 53%); mp 161.5–161.7 °C; ^1^H NMR (400 MHz, DMSO-*d*_6_): δ 8.22–8.11 (m, 2H, ArH), 7.97 (s, 1H, ArH), 7.85–7.76 (m, 3H, ArH), 7.71 (t, *J* = 8.3 Hz, 2H, ArH), 7.58 (t, *J* = 8.0 Hz, 1H, ArH), 7.50 (t, *J* = 7.7 Hz, 2H, ArH), 7.40 (t, *J* = 7.3 Hz, 1H, ArH), 3.69–3.59 (m, 2H, CH_2_), 1.88–1.76 (m, 2H, CH_2_), 1.45–1.15 (m, 10H, CH_2_), 0.83 (t, *J* = 6.9 Hz, 3H, CH_3_); ^13^C NMR (100 MHz, DMSO-*d*_6_): δ 178.7 (CO), 156.6 (CO), 146.2 (ArC), 141.1 (ArC), 139.9 (ArC), 138.7 (ArC), 136.9 (ArC), 136.1 (ArCH), 129.7 (ArCH), 128.9 (ArCH), 127.7 (ArCH), 127.0 (ArCH), 126.4 (ArCH), 125.6 (ArCH), 125.0 (ArCH), 122.9 (ArCH), 120.1 (ArC), 114.5 (ArCH), 53.7 (CH_2_), 31.1 (CH_2_), 28.4 (CH_2_), 28.3 (CH_2_), 27.3 (CH_2_), 22.2 (CH_2_), 22.0 (CH_2_), 13.9 (CH_3_); IR (ATR): *ν*_max_ 3854, 3675, 2970, 2922, 1779, 1744, 1616, 1589, 1496, 1471, 1441, 1404, 1394, 1368, 1298, 1232, 1161, 1140, 1075, 1066, 1057, 1027, 948, 891, 866, 840, 795, 776, 755, 725, 698, 654 cm^−1^; HRMS (+ESI): Found *m*/*z* 498.1710 [M+Na]^+^, C_28_H_29_NO_4_SNa required 498.1710.

##### *N*-(3-(Dimethylamino)propyl)-2-(4-(octylsulfonamido)-[1,1′:3′,1″-terphenyl]-3-yl)-2-oxoacetamide (**21**)

3-Dimethylaminopropylamine (39 μL, 0.31 mmol) was added to a stirring solution of 5-([1,1′-biphenyl]-3-yl)-1-(octylsulfonyl)indoline-2,3-dione **20** (0.15 g, 0.31 mmol) in dichloromethane (5 mL) at 0 °C. The resulting reaction mixture was stirred at room temperature for 3 h. After the completion of the reaction, water was added to the reaction mixture and the mixture was extracted into dichloromethane (3 × 25 mL), washed with brine, dried over anhydrous sodium sulfate and concentrated in vacuo to afford the product as a yellow oil (0.18 g, 99%); ^1^H NMR (400 MHz, CDCl_3_): δ 8.83 (bs, 1H, ArH), 8.77 (s, 1H, ArH), 7.90–7.85 (m, 2H, ArH), 7.77–7.73 (m, 1H, ArH), 7.66–7.43 (m, 7H, ArH), 7.38 (t, *J* = 7.3 Hz, 1H, ArH), 3.52 (t, *J* = 5.9 Hz, 2H, CH_2_), 3.23–3.14 (m, 2H, CH_2_), 2.50 (t, *J* = 6.2 Hz, 2H, CH_2_), 2.29 (s, 6H, CH_3_), 1.87–1.73 (m, 4H, CH_2_), 1.43–1.17 (m, 10H, CH_2_), 0.85 (t, *J* = 7.1 Hz, 3H, CH_3_); ^13^C NMR (100 MHz, CDCl_3_): δ 192.0 (CO), 162.6 (CO), 142.2 (ArC), 141.1 (ArC), 141.0 (ArC), 139.8 (ArC), 135.8 (ArC), 135.1 (ArCH), 133.8 (ArCH), 129.6 (ArCH), 129.0 (ArCH), 127.7 (ArCH), 127.4 (ArCH), 126.8 (ArCH), 126.0 (ArCH), 125.9 (ArCH), 119.7 (ArC), 118.6 (ArCH), 58.9 (CH_2_), 52.7 (CH_2_), 45.4 (CH_3_), 40.1 (CH_2_), 31.8 (CH_2_), 29.1 (CH_2_), 29.0 (CH_2_), 28.2 (CH_2_), 25.3 (CH_2_), 23.5 (CH_2_), 22.7 (CH_2_), 14.2 (CH_3_); IR (ATR): *ν*_max_ 3675, 2970, 2900, 1647, 1498, 1473, 1405, 1393, 1337, 1258, 1196, 1142, 1066, 1057, 891, 798, 757, 700 cm^−1^; HRMS (+ESI): Found *m*/*z* 578.3049 [M+H]^+^, C_33_H_44_N_3_O_4_S required 578.3047.

##### *N*,*N*-Dimethyl-3-(2-(4-(octylsulfonamido)-[1,1′:3′,1″-terphenyl]-3-yl)-2-oxoacetamido)propan-1-aminium chloride (**22**)

4 M HCl in dioxane (0.10 mL, 0.40 mmol) was added to a stirring solution of *N*-(3-(dimethylamino)propyl)-2-(4-(octylsulfonamido)-[1,1′:3′,1″-terphenyl]-3-yl)-2-oxoacetamide **21** (30 mg, 0.052 mmol) in diethyl ether (5 mL). The resulting reaction mixture was stirred at room temperature for 20 min. After the completion of the reaction, the reaction solvent was removed under reduced pressure, and the residue was washed three times with diethyl ether and freeze-dried to afford the product as a yellow sticky solid (25 mg, 67%); ^1^H NMR (600 MHz, DMSO-*d*_6_): δ 10.18 (bs, 2H, NH), 9.01 (t, *J* = 5.3 Hz, 1H, NH), 8.16–8.03 (m, 2H, ArH), 7.89 (s, 1H, ArH), 7.82–7.55 (m, 6H, ArH), 7.50 (t, *J* = 7.4 Hz, 2H, ArH), 7.41 (t, *J* = 7.0 Hz, 1H, ArH), 3.37–3.28 (m, 2H, CH_2_), 3.21 (t, *J* = 7.6 Hz, 2H, CH_2_), 3.09 (t, *J* = 7.6 Hz, 2H, CH_2_), 2.72 (s, 6H, CH_3_), 1.97–1.88 (m, 2H, CH_2_), 1.72–1.63 (m, 2H, CH_2_), 1.38–1.15 (m, 10H, CH_2_), 0.82 (t, *J* = 6.9 Hz, 3H, CH_3_); ^13^C NMR (150 MHz, DMSO-*d*_6_): δ 191.9 (CO), 163.6 (CO), 141.1 (ArC), 139.9 (ArC), 139.1 (ArC), 137.8 (ArC), 136.1 (ArC), 133.0 (ArCH), 130.4 (ArCH), 129.8 (ArCH), 129.0 (ArCH), 127.7 (ArCH), 127.0 (ArCH), 126.4 (ArC), 126.3 (ArCH), 125.7 (ArCH), 125.0 (ArCH), 122.6 (ArCH), 54.5 (CH_2_), 51.3 (CH_2_), 42.1 (CH_3_), 36.0 (CH_2_), 31.1 (CH_2_), 28.4 (CH_2_), 28.3 (CH_2_), 27.3 (CH_2_), 23.9 (CH_2_), 22.9 (CH_2_), 22.0 (CH_2_), 13.9 (CH_3_); IR (ATR): *ν*_max_ 3365, 2923, 2853, 2695, 2361, 1654, 1647, 1497, 1474, 1400, 1332, 1246, 1195, 1143, 1075, 975, 917, 890, 840, 797, 757, 720, 700, 680 cm^−1^; HRMS (+ESI): Found *m*/*z* 578.3049 [M+H]^+^, C_33_H_44_N_3_O_4_S required 578.3047.

##### *N*,*N*,*N*-Trimethyl-3-(2-(4-(octylsulfonamido)-[1,1′:3′,1″-terphenyl]-3-yl)-2-oxoacetamido)propan-1-aminium iodide (**23**)

Iodomethane (10.0 μL, 0.16 mmol) was added to a stirring solution of *N*-(3-(dimethylamino)propyl)-2-(4-(octylsulfonamido)-[1,1′:3′,1″-terphenyl]-3-yl)-2-oxoacetamide **21** (31 mg, 0.054 mmol) in tetrahydrofuran (3 mL). The resulting reaction mixture was stirred at room temperature for 24 h. After the completion of the reaction, the reaction solvent was removed under reduced pressure, and the residue was washed three times with diethyl ether and freeze-dried to afford the product as a yellow sticky solid (33 mg, 86%); ^1^H NMR (600 MHz, DMSO-*d*_6_): δ 10.09 (bs, 1H, ArH), 8.98 (t, *J* = 6.1 Hz, 1H, NH), 8.13–8.06 (m, 2H, ArH), 7.90 (t, *J* = 6.1 Hz, 1H, ArH), 7.78–7.63 (m, 4H, ArH), 7.62–7.56 (m, 2H, ArH), 7.52–7.47 (m, 2H, ArH), 7.43–7.39 (m, 1H, ArH), 3.39–3.29 (m, 4H, CH_2_), 3.18 (t, *J* = 8.0 Hz, 2H, CH_2_), 3.05 (s, 9H, CH_3_), 2.02–1.93 (m, 2H, CH_2_), 1.73–1.63 (m, 2H, CH_2_), 1.40–1.15 (m, 10H, CH_2_), 0.82 (t, *J* = 7.2 Hz, 3H, CH_3_); ^13^C NMR (150 MHz, DMSO-*d*_6_): δ 191.6 (CO), 163.6 (CO), 141.1 (ArC), 139.9 (ArC), 139.1 (ArC), 137.4 (ArC), 136.4 (ArC), 132.9 (ArCH), 130.2 (ArCH), 129.8 (ArCH), 129.0 (ArCH), 127.7 (ArCH), 127.4 (ArC), 127.0 (ArCH), 126.4 (ArCH), 125.7 (ArCH), 125.0 (ArCH), 123.2 (ArCH), 63.5 (CH_2_), 52.3 (CH_3_), 51.2 (CH_2_), 35.9 (CH_2_), 31.1 (CH_2_), 28.4 (CH_2_), 28.3 (CH_2_), 27.3 (CH_2_), 22.9 (CH_2_), 22.6 (CH_2_), 22.0 (CH_2_), 13.9 (CH_3_); IR (ATR): *ν*_max_ 3404, 2925, 2854, 2360, 1979, 1670, 1642, 1475, 1400, 1332, 1260, 1194, 1140, 1075, 916, 889, 842, 798, 758, 701, 680 cm^−1^; HRMS (+ESI): Found *m*/*z* 592.3206 [M]^+^, C_34_H_46_N_3_O_4_S required 592.3204.

**General synthetic procedure E for Boc-protected glyoxamides**

A solution of *N*-Boc-1,3-propanediamine (1.0 equivalent) in dichloromethane (3 mL) was added dropwise to a stirring solution of *N*-sulfonylisatin (1.0 equivalent) in dichloromethane (10 mL) at 0 °C. The resulting reaction mixture was stirred at room temperature for 6 h. After the completion of the reaction, water was added to the reaction mixture, and the mixture was extracted into dichloromethane (3 × 25 mL), washed with brine, dried over anhydrous sodium sulfate and concentrated in vacuo to afford the product.

##### *tert*-Butyl (3-(2-(4-(octylsulfonamido)-[1,1′:4′,1″-terphenyl]-3-yl)-2-oxoacetamido)propyl)carbamate (**24d**)

The titled compound was synthesised from 5-([1,1′-biphenyl]-4-yl)-1-(octylsulfonyl)indoline-2,3-dione **7d** (0.35 g, 0.73 mmol) and *N*-Boc-1,3-propandiamine (0.13 g, 0.75 mmol) following **general synthetic procedure E**. The product was obtained as a brown solid (0.47 g, 98%); mp 129.4–129.6 °C; ^1^H NMR (400 MHz, CDCl_3_): δ 10.50 (s, 1H, NH), 8.81 (s, 1H, ArH), 7.91–7.84 (m, 2H, ArH), 7.77–7.59 (m, 7H, NH, ArH), 7.47 (t, *J* = 7.8 Hz, 2H, ArH), 7.37 (t, *J* = 7.1 Hz, 1H, ArH), 4.81 (bs, 1H, NH), 3.49 (q, *J* = 6.3 Hz, 2H, CH_2_), 3.30–3.15 (m, 4H, CH_2_), 1.88–1.71 (m, 4H, CH_2_), 1.45 (s, 9H, CH_3_), 1.49–1.16 (m, 10H, CH_2_), 0.86 (t, *J* = 7.1 Hz, 3H, CH_3_); ^13^C NMR (100 MHz, CDCl_3_): δ 191.5 (CO), 162.9 (CO), 156.9 (CO), 141.1 (ArC), 140.8 (ArC), 140.6 (ArC), 137.9 (ArC), 135.2 (ArC), 135.0 (ArCH), 133.5 (ArCH), 129.0 (ArCH), 127.9 (ArCH), 127.7 (ArCH), 127.3 (ArCH), 127.2 (ArCH), 119.4 (ArC), 118.6 (ArCH), 79.9 (C), 52.8 (CH_2_), 37.3 (CH_2_), 36.4 (CH_2_), 31.8 (CH_2_), 30.2 (CH_2_), 29.1 (CH_2_), 29.0 (CH_2_), 28.5 (CH_3_), 28.2 (CH_2_), 23.6 (CH_2_), 22.7 (CH_2_), 14.2 (CH_3_); IR (ATR): *ν*_max_ 3350, 2924, 2869, 2360, 1685, 1666, 1641, 1521, 1481, 1449, 1387, 1364, 1341, 1267, 1245, 1213, 1195, 1154, 1070, 1039, 1006, 977, 912, 900, 883, 866, 830, 762, 716, 689, 673 cm^−1^; HRMS (+ESI): Found *m*/*z* 672.3082 [M+Na]^+^, C_36_H_47_N_3_O_6_SNa required 672.3078.

##### *tert*-Butyl (3-(2-(2-(dodecylsulfonamido)phenyl)-2-oxoacetamido)propyl)carbamate (**25a**)

The titled compound was synthesised from 1-(dodecylsulfonyl)indoline-2,3-dione **8a** (0.32 g, 0.84 mmol) and *N*-Boc-1,3-propandiamine (0.15 g, 0.84 mmol) following **general synthetic procedure E**. The product was obtained as a pale yellow solid (0.42 g, 89%); mp 77.2–80.5 °C; ^1^H NMR (400 MHz, CDCl_3_): δ 10.49 (s, 1H, NH), 8.44 (d, *J* = 7.9 Hz, 1H, ArH), 7.77 (d, *J* = 8.5 Hz, 1H, ArH), 7.68–7.55 (m, 2H, NH, ArH), 7.19–7.11 (m, 1H, ArH), 4.87 (bs, 1H, NH), 3.46 (q, *J* = 6.3 Hz, 2H, CH_2_), 3.28–3.11 (m, 4H, CH_2_), 1.85–1.69 (m, 4H, CH_2_), 1.44 (s, 9H, CH_3_), 1.41–1.15 (m, 18H, CH_2_), 0.87 (t, *J* = 7.0 Hz, 3H, CH_3_); ^13^C NMR (100 MHz, CDCl_3_): δ 191.7 (CO), 163.1 (CO), 156.9 (CO), 142.1 (ArC), 136.7 (ArCH), 135.3 (ArCH), 122.6 (ArCH), 118.8 (ArC), 117.9 (ArCH), 79.9 (C), 52.7 (CH_2_), 37.3 (CH_2_), 36.4 (CH_2_), 32.0 (CH_2_), 30.2 (CH_2_), 29.7 (CH_2_), 29.7 (CH_2_), 29.6 (CH_2_), 29.4 (CH_2_), 29.4 (CH_2_), 29.1 (CH_2_), 28.5 (CH_3_), 28.2 (CH_2_), 23.5 (CH_2_), 22.8 (CH_2_), 14.2 (CH_3_); IR (ATR): *ν*_max_ 3363, 3238, 2918, 2850, 2297, 2106, 1684, 1637, 1602, 1571, 1523, 1449, 1398, 1334, 1247, 1220, 1152, 1077, 1039, 1005, 918, 817, 753, 671 cm^−1^; HRMS (+ESI): Found *m*/*z* 576.3078 [M+Na]^+^, C_28_H_47_N_3_O_6_SNa required 576.3078.

##### *tert*-Butyl (3-(2-(5-bromo-2-(dodecylsulfonamido)phenyl)-2-oxoacetamido)propyl)carbamate (**25b**)

The titled compound was synthesised from 5-bromo-1-(dodecylsulfonyl)indoline-2,3-dione **8b** (0.28 g, 0.60 mmol) and *N*-Boc-1,3-propandiamine (0.11 g, 0.60 mmol) following **general synthetic procedure E**. The product was obtained as a yellow solid (0.34 g, 89%); mp 98.0–98.2 °C; ^1^H NMR (400 MHz, CDCl_3_): δ 10.38 (s, 1H, NH), 8.66 (s, 1H, ArH), 7.76 (bs, 1H, NH), 7.71–7.66 (m, 2H, ArH), 4.80 (bs, 1H, NH), 3.46 (q, *J* = 6.4 Hz, 2H, CH_2_), 3.29–3.19 (m, 2H, CH_2_), 3.19–3.09 (m, 2H, CH_2_), 1.83–1.70 (m, 4H, CH_2_), 1.45 (s, 9H, CH_3_), 1.41–1.17 (m, 18H, CH_2_), 0.87 (t, *J* = 7.1 Hz, 3H, CH_3_); ^13^C NMR (100 MHz, CDCl_3_): δ 190.4 (CO), 162.2 (CO), 157.0 (CO), 141.0 (ArC), 139.3 (ArCH), 137.4 (ArCH), 120.4 (ArCH), 119.8 (ArC), 115.2 (ArC), 80.0 (C), 52.9 (CH_2_), 37.2 (CH_2_), 36.4 (CH_2_), 32.0 (CH_2_), 30.2 (CH_2_), 29.7 (CH_2_), 29.7 (CH_2_), 29.6 (CH_2_), 29.5 (CH_2_), 29.4 (CH_2_), 29.1 (CH_2_), 28.5 (CH_3_), 28.2 (CH_2_), 23.5 (CH_2_), 22.8 (CH_2_), 14.3 (CH_3_); IR (ATR): *ν*_max_ 3382, 3329, 3173, 2918, 2850, 1683, 1664, 1630, 1560, 1519, 1479, 1452, 1392, 1366, 1339, 1315, 1283, 1249, 1198, 1170, 1147, 1066, 1039, 1012, 988, 916, 889, 860, 823, 776, 719, 708, 659 cm^−1^; HRMS (+ESI): Found *m*/*z* 654.2187 [M+Na]^+^, C_28_H_46_BrN_3_O_6_SNa required 654.2183.

##### *tert*-Butyl (3-(2-(4-(dodecylsulfonamido)-[1,1′-biphenyl]-3-yl)-2-oxoacetamido)propyl)carbamate (**25c**)

The titled compound was synthesised from 1-(dodecylsulfonyl)-5-phenylindoline-2,3-dione **8c** (0.32 g, 0.71 mmol) and *N*-Boc-1,3-propandiamine (0.13 g, 0.71 mmol) following **general synthetic procedure E**. The product was obtained as a yellow solid (0.39 g, 88%); mp 120.8–121.0 °C; ^1^H NMR (400 MHz, CDCl_3_): δ 10.48 (s, 1H, NH), 8.75 (s, 1H, ArH), 7.89–7.79 (m, 2H, ArH), 7.71 (bs, 1H, NH), 7.60–7.53 (m, 2H, ArH), 7.48–7.41 (m, 2H, ArH), 7.39–7.33 (m, 1H, ArH), 4.81 (bs, 1H, NH), 3.48 (q, *J* = 6.3 Hz, 2H, CH_2_), 3.29–3.14 (m, 4H, CH_2_), 1.87–1.71 (m, 4H, CH_2_), 1.44 (s, 9H, CH_3_), 1.43–1.17 (m, 18H, CH_2_), 0.87 (t, *J* = 7.1 Hz, 3H, CH_3_); ^13^C NMR (100 MHz, CDCl_3_): δ 191.5 (CO), 162.9 (CO), 156.9 (CO), 141.1 (ArC), 139.1 (ArC), 135.7 (ArC), 135.1 (ArCH), 133.6 (ArCH), 129.2 (ArCH), 127.9 (ArCH), 126.9 (ArCH), 119.3 (ArC), 118.5 (ArCH), 79.9 (C), 52.8 (CH_2_), 37.3 (CH_2_), 36.4 (CH_2_), 32.0 (CH_2_), 30.2 (CH_2_), 29.7 (CH_2_), 29.7 (CH_2_), 29.6 (CH_2_), 29.4 (CH_2_), 29.4 (CH_2_), 29.1 (CH_2_), 28.5 (CH_3_), 28.2 (CH_2_), 23.5 (CH_2_), 22.8 (CH_2_), 14.2 (CH_3_); IR (ATR): *ν*_max_ 3351, 3308, 2922, 2851, 1683, 1666, 1644, 1582, 1518, 1483, 1443, 1386, 1364, 1347, 1325, 1294, 1247, 1196, 1155, 1070, 1040, 1011, 977, 906, 895, 882, 864, 850, 804, 776, 758, 740, 716, 684 cm^−1^; HRMS (+ESI): Found *m*/*z* 652.3394 [M+Na]^+^, C_34_H_51_N_3_O_6_SNa required 652.3391.

##### *tert*-Butyl (3-(2-(2-(hexadecylsulfonamido)phenyl)-2-oxoacetamido)propyl)carbamate (**26a**)

The titled compound was synthesised from 1-(hexadecylsulfonyl)indoline-2,3-dione **9a** (0.21 g, 0.47 mmol) and *N*-Boc-1,3-propandiamine (85 mg, 0.47 mmol) following **general synthetic procedure E**. The product was obtained as a yellow solid (0.28 g, 97%); mp 86.7–90.1 °C; ^1^H NMR (400 MHz, CDCl_3_): δ 10.50 (s, 1H, NH), 8.45 (d, *J* = 7.9 Hz, 1H, ArH), 7.77 (d, *J* = 8.4 Hz, 1H, ArH), 7.68–7.56 (m, 2H, NH, ArH), 7.19–7.11 (m, 1H, ArH), 4.84 (bs, 1H, NH), 3.47 (q, *J* = 6.5 Hz, 2H, CH_2_), 3.29–3.11 (m, 4H, CH_2_), 1.85–1.69 (m, 4H, CH_2_), 1.44 (s, 9H, CH_3_), 1.40–1.16 (m, 26H, CH_2_), 0.87 (t, *J* = 7.0 Hz, 3H, CH_3_); ^13^C NMR (150 MHz, CDCl_3_): δ 191.7 (CO), 163.1 (CO), 156.9 (CO), 142.1 (ArC), 136.7 (ArCH), 135.3 (ArCH), 122.6 (ArCH), 118.8 (ArC), 118.0 (ArCH), 79.9 (C), 52.7 (CH_2_), 37.3 (CH_2_), 36.4 (CH_2_), 32.1 (CH_2_), 30.2 (CH_2_), 29.8 (CH_2_), 29.8 (CH_2_), 29.8 (CH_2_), 29.8 (CH_2_), 29.8 (CH_2_), 29.7 (CH_2_), 29.6 (CH_2_), 29.5 (CH_2_), 29.4 (CH_2_), 29.1 (CH_2_), 28.5 (CH_3_), 28.2 (CH_2_), 23.5 (CH_2_), 22.8 (CH_2_), 14.3 (CH_3_); IR (ATR): *ν*_max_ 3362, 3253, 2916, 2849, 2113, 1684, 1637, 1602, 1571, 1523, 1467, 1391, 1335, 1247, 1220, 1152, 1077, 1057, 1006, 918, 818, 753, 671 cm^−1^; HRMS (+ESI): Found *m*/*z* 632.3703 [M+Na]^+^, C_32_H_55_N_3_O_6_SNa required 632.3704.

##### *tert*-Butyl (3-(2-(5-bromo-2-(hexadecylsulfonamido)phenyl)-2-oxoacetamido)propyl)carbamate (**26b**)

The titled compound was synthesised from 5-bromo-1-(hexadecylsulfonyl)indoline-2,3-dione **9b** (0.33 g, 0.64 mmol) and *N*-Boc-1,3-propandiamine (0.12 g, 0.65 mmol) following **general synthetic procedure E**. The product was obtained as a yellow solid (0.41 g, 91%); mp 81.7–83.1 °C; ^1^H NMR (400 MHz, CDCl_3_): δ 10.38 (s, 1H, NH), 8.66 (s, 1H, ArH), 7.76 (bs, 1H, NH), 7.73–7.64 (m, 2H, NH, ArH), 4.80 (bs, 1H, NH), 3.46 (q, *J* = 6.5 Hz, 2H, CH_2_), 3.28–3.19 (m, 2H, CH_2_), 3.17–3.10 (m, 2H, CH_2_), 1.83–1.70 (m, 4H, CH_2_), 1.45 (s, 9H, CH_3_), 1.41–1.17 (m, 26H, CH_2_), 0.87 (t, *J* = 7.1 Hz, 3H, CH_3_); ^13^C NMR (100 MHz, CDCl_3_): δ 190.4 (CO), 162.2 (CO), 157.0 (CO), 141.0 (ArC), 139.3 (ArCH), 137.4 (ArCH), 120.4 (ArC), 119.8 (ArCH), 115.2 (ArC), 80.0 (C), 52.9 (CH_2_), 37.2 (CH_2_), 36.4 (CH_2_), 32.1 (CH_2_), 30.2 (CH_2_), 29.8 (CH_2_), 29.8 (CH_2_), 29.8 (CH_2_), 29.8 (CH_2_), 29.8 (CH_2_), 29.7 (CH_2_), 29.6 (CH_2_), 29.5 (CH_2_), 29.4 (CH_2_), 29.1 (CH_2_), 28.5 (CH_3_), 28.2 (CH_2_), 23.5 (CH_2_), 22.8 (CH_2_), 14.3 (CH_3_); IR (ATR): *ν*_max_ 3370, 3184, 3113, 2918, 2850, 1685, 1635, 1519, 1479, 1389, 1335, 1248, 1145, 1010, 918, 826. 782, 709 cm^−1^; HRMS (+ESI): Found *m*/*z* 710.2810 [M+Na]^+^, C_32_H_54_BrN_3_O_6_SNa required 710.2809.

##### *tert*-Butyl (3-(2-(4-(hexadecylsulfonamido)-[1,1′-biphenyl]-3-yl)-2-oxoacetamido)propyl)carbamate (**26c**)

The titled compound was synthesised from 1-(hexadecylsulfonyl)-5-phenylindoline-2,3-dione **9c** (0.26 g, 0.51 mmol) and *N*-Boc-1,3-propandiamine (91 mg, 0.51 mmol) following **general synthetic procedure E**. The product was obtained as a yellow solid (0.32 g, 93%); mp 121.6–128.3 °C; ^1^H NMR (400 MHz, CDCl_3_): δ 10.48 (s, 1H, NH), 8.75 (s, 1H, ArH), 7.89–7.80 (m, 2H, ArH), 7.70 (bs, 1H, NH), 7.60–7.53 (m, 2H, ArH), 7.48–7.41 (m, 2H, ArH), 7.39–7.34 (m, 1H, ArH), 4.80 (bs, 1H, NH), 3.48 (q, *J* = 6.4 Hz, 2H, CH_2_), 3.29–3.14 (m, 4H, CH_2_), 1.87–1.70 (m, 4H, CH_2_), 1.44 (s, 9H, CH_3_), 1.43–1.17 (m, 26H, CH_2_), 0.87 (t, *J* = 7.1 Hz, 3H, CH_3_); ^13^C NMR (100 MHz, CDCl_3_): δ 191.5 (CO), 162.9 (CO), 156.9 (CO), 141.1 (ArC), 139.1 (ArC), 135.7 (ArC), 135.1 (ArCH), 133.7 (ArCH), 129.2 (ArCH), 127.9 (ArCH), 126.9 (ArCH), 119.3 (ArC), 118.5 (ArCH), 79.9 (C), 52.8 (CH_2_), 37.3 (CH_2_), 36.4 (CH_2_), 32.0 (CH_2_), 30.2 (CH_2_), 29.8 (CH_2_), 29.8 (CH_2_), 29.8 (CH_2_), 29.8 (CH_2_), 29.8 (CH_2_), 29.7 (CH_2_), 29.6 (CH_2_), 29.5 (CH_2_), 29.4 (CH_2_), 29.1 (CH_2_), 28.5 (CH_3_), 28.3 (CH_2_), 23.6 (CH_2_), 22.8 (CH_2_), 14.3 (CH_3_); IR (ATR): *ν*_max_ 3350, 3306, 2918, 2848, 2288, 2110, 1681, 1644, 1518, 1482, 1383, 1347, 1247, 1154, 1069, 1011, 975, 906, 864, 757, 684 cm^−1^; HRMS (+ESI): Found *m*/*z* 708.4018 [M+Na]^+^, C_38_H_59_N_3_O_6_SNa required 708.4017.

**General synthetic procedure F for aminoglyoxamides**

4 M HCl in dioxane (3 mL) was added to a stirring solution of Boc-protected glyoxamide (1.0 equivalent) in dichloromethane (10 mL) at 0 °C. The reaction mixture was stirred at room temperature for 6 h. After the completion of the reaction, the reaction solvent was removed under reduced pressure, and the residue was washed three times with diethyl ether and freeze-dried to afford the product.

##### *N*-(3-Aminopropyl)-2-(4-(octylsulfonamido)-[1,1′:4′,1″-terphenyl]-3-yl)-2-oxoacetamide hydrochloride (**27d**)

The titled compound was synthesised from *tert*-butyl (3-(2-(4-(octylsulfonamido)-[1,1′:4′,1″-terphenyl]-3-yl)-2-oxoacetamido)propyl)carbamate **24d** (0.43 g, 0.67 mmol) following **general synthetic procedure F**. The product was obtained as a yellow solid (0.34 g, 88%); mp 165.1–165.4 °C; ^1^H NMR (400 MHz, DMSO-*d*_6_): δ 10.22 (s, 1H, NH), 9.07 (t, *J* = 5.9 Hz, 1H, NH), 8.12–8.04 (m, 2H, ArH), 8.00 (bs, 3H, NH), 7.82 (d, *J* = 8.3 Hz, 2H, ArH), 7.78–7.70 (m, 4H, ArH), 7.66 (d, *J* = 9.3 Hz, 1H, ArH), 7.50 (t, *J* = 7.7 Hz, 2H, ArH), 7.39 (t, *J* = 7.2 Hz, 1H, ArH), 3.39–3.31 (m, 2H, CH_2_), 3.31–3.22 (m, 2H, CH_2_), 2.93–2.81 (m, 2H, CH_2_), 1.92–1.79 (m, 2H, CH_2_), 1.74–1.62 (m, 2H, CH_2_), 1.40–1.13 (m, 10H, CH_2_), 0.82 (t, *J* = 7.0 Hz, 3H, CH_3_); ^13^C NMR (150 MHz, DMSO-*d*_6_): δ 192.4 (CO), 163.8 (CO), 139.6 (ArC), 139.4 (ArC), 138.3 (ArC), 137.1 (ArC), 135.0 (ArC), 133.0 (ArCH), 130.3 (ArCH), 129.0 (ArCH), 127.7 (ArCH), 127.5 (ArCH), 126.9 (ArCH), 126.6 (ArCH), 124.5 (ArC), 121.7 (ArCH), 51.5 (CH_2_), 36.7 (CH_2_), 35.9 (CH_2_), 31.1 (CH_2_), 28.4 (CH_2_), 28.3 (CH_2_), 27.3 (CH_2_), 26.9 (CH_2_), 22.9 (CH_2_), 22.0 (CH_2_), 13.9 (CH_3_); IR (ATR): *ν*_max_ 2924, 2854, 1719, 1648, 1497, 1396, 1332, 1255, 1195, 1139, 1051, 1006, 916, 826, 763, 717, 695, 673 cm^−1^; HRMS (+ESI): Found *m*/*z* 550.2736 [M+H]^+^, C_31_H_40_N_3_O_4_S required 550.2734.

##### *N*-(3-Aminopropyl)-2-(2-(dodecylsulfonamido)phenyl)-2-oxoacetamide hydrochloride (**28a**)

The titled compound was synthesised from *tert*-butyl (3-(2-(2-(dodecylsulfonamido)phenyl)-2-oxoacetamido)propyl)carbamate **25a** (0.38 g, 0.68 mmol) following **general synthetic procedure F**. The product was obtained as a pale yellow solid (0.29 g, 86%); mp 160.0–162.3 °C; ^1^H NMR (400 MHz, DMSO-*d*_6_): δ 10.27 (s, 1H, NH), 9.03 (t, *J* = 5.9 Hz, 1H, NH), 8.03 (bs, 3H, NH), 7.78 (dd, *J* = 7.9, 1.2 Hz, 1H, ArH), 7.73–7.67 (m, 1H, ArH), 7.56 (d, *J* = 8.0 Hz, 1H, ArH), 7.34–7.26 (m, 1H, ArH), 3.37–3.21 (m, 4H, CH_2_), 2.91–2.77 (m, 2H, CH_2_), 1.90–1.78 (m, 2H, CH_2_), 1.71–1.59 (m, 2H, CH_2_), 1.38–1.13 (m, 18H, CH_2_), 0.85 (t, *J* = 7.0 Hz, 3H, CH_3_); ^13^C NMR (100 MHz, DMSO-*d*_6_): δ 193.3 (CO), 164.3 (CO), 139.6 (ArC), 135.5 (ArCH), 133.2 (ArCH), 123.6 (ArCH), 122.5 (ArC), 120.2 (ArCH), 51.4 (CH_2_), 36.7 (CH_2_), 35.8 (CH_2_), 31.3 (CH_2_), 29.0 (CH_2_), 28.9 (CH_2_), 28.7 (CH_2_), 28.7 (CH_2_), 28.4 (CH_2_), 27.3 (CH_2_), 26.9 (CH_2_), 22.9 (CH_2_), 22.1 (CH_2_), 14.0 (CH_3_); IR (ATR): *ν*_max_ 3336, 3238, 2916, 2849, 2765, 2674, 2635, 2522, 2299, 2033, 1635, 1602, 1568, 1523, 1490, 1445, 1399, 1337, 1259, 1211, 1153, 1080, 958, 913, 849, 816, 753, 670 cm^−1^; HRMS (+ESI): Found *m*/*z* 454.2733 [M+H]^+^, C_23_H_40_N_3_O_4_S required 454.2734.

##### *N*-(3-Aminopropyl)-2-(5-bromo-2-(dodecylsulfonamido)phenyl)-2-oxoacetamide hydrochloride (**28b**)

The titled compound was synthesised from *tert*-butyl (3-(2-(5-bromo-2-(dodecylsulfonamido)phenyl)-2-oxoacetamido)propyl)carbamate **25b** (0.33 g, 0.52 mmol) following **general synthetic procedure F**. The product was obtained as a white solid (0.20 g, 66%); mp 73.7–74.2 °C; ^1^H NMR (400 MHz, DMSO-*d*_6_): δ 10.07 (s, 1H, NH), 8.98 (t, *J* = 5.9 Hz, 1H, NH), 7.92 (bs, 3H, NH), 7.90–7.82 (m, 2H, ArH), 7.46 (d, *J* = 8.6 Hz, 1H, ArH), 3.36–3.25 (m, 2H, CH_2_), 3.21–3.13 (m, 2H, CH_2_), 2.92–2.78 (m, 2H, CH_2_), 1.87–1.76 (m, 2H, CH_2_), 1.69–1.57 (m, 2H, CH_2_), 1.37–1.13 (m, 18H, CH_2_), 0.85 (t, *J* = 7.0 Hz, 3H, CH_3_); ^13^C NMR (100 MHz, DMSO-*d*_6_): δ 190.3 (CO), 162.9 (CO), 137.6 (ArC), 137.0 (ArCH), 134.2 (ArCH), 127.7 (ArC), 124.3 (ArCH), 116.2 (ArC), 51.4 (CH_2_), 36.7 (CH_2_), 35.9 (CH_2_), 31.3 (CH_2_), 29.0 (CH_2_), 28.9 (CH_2_), 28.7 (CH_2_), 28.7 (CH_2_), 28.4 (CH_2_), 27.3 (CH_2_), 26.8 (CH_2_), 22.8 (CH_2_), 22.1 (CH_2_), 14.0 (CH_3_); IR (ATR): *ν*_max_ 3375, 2915, 2849, 2048, 1667, 1641, 1565, 1516, 1485, 1437, 1389, 1339, 1323, 1293, 1274, 1256, 1194, 1141, 1075, 1021, 992, 967, 923, 872, 819, 765, 722, 662 cm^−1^; HRMS (+ESI): Found *m*/*z* 532.1841 [M+H]^+^, C_23_H_39_BrN_3_O_4_S required 532.1839.

##### *N*-(3-Aminopropyl)-2-(4-(dodecylsulfonamido)-[1,1′-biphenyl]-3-yl)-2-oxoacetamide hydrochloride (**28c**)

The titled compound was synthesised from *tert*-butyl (3-(2-(4-(dodecylsulfonamido)-[1,1′-biphenyl]-3-yl)-2-oxoacetamido)propyl)carbamate **25c** (0.35 g, 0.55 mmol) following **general synthetic procedure F**. The product was obtained as a yellow solid (0.19 g, 60%); mp 118.0 °C; ^1^H NMR (600 MHz, DMSO-*d*_6_): δ 10.19 (s, 1H, NH), 9.03 (t, *J* = 6.0 Hz, 1H, NH), 8.03–7.98 (m, 2H, ArH), 7.91 (bs, 3H, NH), 7.66–7.60 (m, 3H, ArH), 7.53–7.48 (m, 2H, ArH), 7.43–7.39 (m, 1H, ArH), 3.40–3.28 (m, 2H, CH_2_), 3.27–3.22 (m, 2H, CH_2_), 2.90–2.82 (m, 2H, CH_2_), 1.88–1.80 (m, 2H, CH_2_), 1.71–1.62 (m, 2H, CH_2_), 1.37–1.29 (m, 2H, CH_2_), 1.28–1.12 (m, 16H, CH_2_), 0.84 (t, *J* = 7.1 Hz, 3H, CH_3_); ^13^C NMR (150 MHz, DMSO-*d*_6_): δ 192.4 (CO), 163.8 (CO), 138.3 (ArC), 138.2 (ArC), 135.6 (ArC), 133.2 (ArCH), 130.5 (ArCH), 129.2 (ArCH), 127.9 (ArCH), 126.4 (ArCH), 124.6 (ArC), 121.7 (ArCH), 51.4 (CH_2_), 36.7 (CH_2_), 35.9 (CH_2_), 31.3 (CH_2_), 29.0 (CH_2_), 28.9 (CH_2_), 28.7 (CH_2_), 28.7 (CH_2_), 28.4 (CH_2_), 27.3 (CH_2_), 26.9 (CH_2_), 22.9 (CH_2_), 22.1 (CH_2_), 14.0 (CH_3_); IR (ATR): *ν*_max_ 3351, 3307, 2921, 2851, 1682, 1666, 1644, 1582, 1518, 1483, 1444, 1386, 1364, 1347, 1326, 1294, 1247, 1197, 1155, 1138, 1070, 1011, 978, 907, 895, 881, 864, 851, 830, 805, 758, 741, 716, 684 cm^−1^; HRMS (+ESI): Found *m*/*z* 530.3048 [M+H]^+^, C_29_H_44_N_3_O_4_S required 530.3047.

##### *N*-(3-Aminopropyl)-2-(2-(hexadecylsulfonamido)phenyl)-2-oxoacetamide hydrochloride (**29a**)

The titled compound was synthesised from *tert*-butyl (3-(2-(2-(hexadecylsulfonamido)phenyl)-2-oxoacetamido)propyl)carbamate **26a** (0.24 g, 0.39 mmol) following **general synthetic procedure F**. The product was obtained as a pale yellow solid (0.19 g, 86%); mp 157.3–160.0 °C; ^1^H NMR (400 MHz, DMSO-*d*_6_): δ 10.26 (s, 1H, NH), 9.02 (t, *J* = 5.9 Hz, 1H, NH), 7.95 (bs, 3H, NH), 7.77 (dd, *J* = 7.9, 1.3 Hz, 1H, ArH), 7.73–7.67 (m, 1H, ArH), 7.55 (d, *J* = 8.2 Hz, 1H, ArH), 7.30 (d, *J* = 7.3 Hz, 1H, ArH), 3.31 (q, *J* = 6.6 Hz, 2H, CH_2_), 3.28–3.21 (m, 2H, CH_2_), 2.90–2.79 (m, 2H, CH_2_), 1.87–1.77 (m, 2H, CH_2_), 1.70–1.59 (m, 2H, CH_2_), 1.37–1.13 (m, 26H, CH_2_), 0.85 (t, *J* = 7.0 Hz, 3H, CH_3_); ^13^C NMR (100 MHz, DMSO-*d*_6_): δ 193.3 (CO), 164.3 (CO), 139.5 (ArC), 135.5 (ArCH), 133.2 (ArCH), 123.7 (ArCH), 122.7 (ArC), 120.3 (ArCH), 51.4 (CH_2_), 36.7 (CH_2_), 35.8 (CH_2_), 31.3 (CH_2_), 29.1 (CH_2_), 29.1 (CH_2_), 29.0 (CH_2_), 29.0 (CH_2_), 28.9 (CH_2_), 28.7 (CH_2_), 28.7 (CH_2_), 28.4 (CH_2_), 27.3 (CH_2_), 26.9 (CH_2_), 22.9 (CH_2_), 22.1 (CH_2_), 14.0 (CH_3_); IR (ATR): *ν*_max_ 3336, 3237, 2915, 2848, 2780, 2673, 2636, 2521, 2322, 2033, 1634, 1602, 1569, 1524, 1491, 1446, 1399, 1338, 1259, 1211, 1154, 1080, 958, 913, 850, 818, 753, 739, 670 cm^−1^; HRMS (+ESI): Found *m*/*z* 510.3361 [M+H]^+^, C_27_H_48_N_3_O_4_S required 510.3360.

##### *N*-(3-Aminopropyl)-2-(5-bromo-2-(hexadecylsulfonamido)phenyl)-2-oxoacetamide hydrochloride (**29b**)

The titled compound was synthesised from *tert*-butyl (3-(2-(5-bromo-2-(hexadecylsulfonamido) phenyl)-2-oxoacetamido)propyl)carbamate **26b** (0.36 g, 0.53 mmol) following **general synthetic procedure F**. The product was obtained as a yellow solid (0.31 g, 95%); mp 81.4–83.9 °C; ^1^H NMR (400 MHz, DMSO-*d*_6_): δ 10.07 (s, 1H, NH), 8.98 (t, *J* = 6.0 Hz, 1H, NH), 8.13–7.79 (m, 5H, NH, ArH), 7.45 (d, *J* = 8.7 Hz, 1H, ArH), 3.29 (q, *J* = 6.8 Hz, 2H, CH_2_), 3.21–3.13 (m, 2H, CH_2_), 2.91–2.79 (m, 2H, CH_2_), 1.87–1.76 (m, 2H, CH_2_), 1.68–1.57 (m, 2H, CH_2_), 1.36–1.13 (m, 26H, CH_2_), 0.85 (t, *J* = 7.0 Hz, 3H, CH_3_); ^13^C NMR (100 MHz, DMSO-*d*_6_): δ 190.3 (CO), 162.9 (CO), 137.6 (ArC), 137.0 (ArCH), 134.2 (ArCH), 127.7 (ArC), 124.3 (ArCH), 116.2 (ArC), 51.4 (CH_2_), 36.7 (CH_2_), 35.9 (CH_2_), 31.3 (CH_2_), 29.0 (CH_2_), 29.0 (CH_2_), 28.9 (CH_2_), 28.7 (CH_2_), 28.3 (CH_2_), 27.3 (CH_2_), 26.8 (CH_2_), 22.8 (CH_2_), 22.1 (CH_2_), 14.0 (CH_3_); IR (ATR): *ν*_max_ 3379, 3174, 2916, 2848, 2342, 2051, 1920, 1642, 1525, 1479, 1384, 1337, 1197, 1146, 1074, 1017, 908, 823, 718 cm^−1^; HRMS (+ESI): Found *m*/*z* 588.2466 [M+H]^+^, C_27_H_47_BrN_3_O_4_S required 588.2465.

##### *N*-(3-Aminopropyl)-2-(4-(hexadecylsulfonamido)-[1,1′-biphenyl]-3-yl)-2-oxoacetamide hydrochloride (**29c**)

The titled compound was synthesised from *tert*-butyl (3-(2-(4-(hexadecylsulfonamido)-[1,1′-biphenyl]-3-yl)-2-oxoacetamido)propyl)carbamate **26c** (0.30 g, 0.43 mmol) following **general synthetic procedure F**. The product was obtained as a yellow solid (0.24 g, 89%); mp 81.9–84.1 °C; ^1^H NMR (400 MHz, DMSO-*d*_6_): δ 10.20 (s, 1H, NH), 9.05 (t, *J* = 6.0 Hz, 1H, NH), 8.08–7.90 (m, 5H, NH, ArH), 7.66–7.60 (m, 3H, ArH), 7.50 (t, *J* = 7.8 Hz, 2H, ArH), 7.43–7.37 (m, 1H, ArH), 3.40–3.29 (m, 2H, CH_2_), 3.29–3.21 (m, 2H, CH_2_), 2.92–2.80 (m, 2H, CH_2_), 1.89–1.79 (m, 2H, CH_2_), 1.72–1.61 (m, 2H, CH_2_), 1.39–1.12 (m, 26H, CH_2_), 0.84 (t, *J* = 7.0 Hz, 3H, CH_3_); ^13^C NMR (100 MHz, DMSO-*d*_6_): δ 192.4 (CO), 163.8 (CO), 138.3 (ArC), 138.2 (ArC), 135.6 (ArC), 133.1 (ArCH), 130.5 (ArCH), 129.2 (ArCH), 127.9 (ArCH), 126.4 (ArCH), 124.4 (ArC), 121.7 (ArCH), 51.5 (CH_2_), 36.7 (CH_2_), 35.9 (CH_2_), 31.3 (CH_2_), 29.0 (CH_2_), 29.0 (CH_2_), 28.9 (CH_2_), 28.7 (CH_2_), 28.7 (CH_2_), 28.4 (CH_2_), 27.3 (CH_2_), 26.9 (CH_2_), 22.9 (CH_2_), 22.1 (CH_2_), 13.9 (CH_3_); IR (ATR): *ν*_max_ 3346, 3184, 2917, 2849, 2648, 2529, 2342, 2053, 1631, 1525, 1484, 1392, 1342, 1265, 1199, 1140, 1078, 1022, 922, 865, 840, 761, 682 cm^−1^; HRMS (+ESI): Found *m*/*z* 586.3673 [M+H]^+^, C_33_H_52_N_3_O_4_S required 586.3673.

**General synthetic procedure G for Boc-protected guanidine glyoxamides**

A solution of triethylamine (2.5 equivalents) in acetonitrile (3 mL) was added dropwise to a stirring solution of aminoglyoxamide (1.0 equivalent) and *N*,*N*′-di-Boc-1*H*-pyrazole-1-carboxamidine (1.3 equivalents) in acetonitrile (10 mL) at 0 °C under an atmosphere of nitrogen. The resulting reaction mixture was stirred at room temperature for 24 h. After the completion of the reaction, the reaction solvent was removed under reduced pressure. The resulting residue was purified by flash column chromatography on silica, using ethyl acetate/*n*-hexane (1:4) as an eluent to afford the product.

##### (*E*)-1-*tert*-Butyl-*N*-(*N*′-((*tert*-butyloxidanyl)carbonyl)-*N*-(3-(2-(4-(octylsulfonamido)-[1,1′:4′,1″-terphenyl]-3-yl)-2-oxoacetamido)propyl)carbamimidoyl)-1-oxidanecarboxamide (**30d**)

The titled compound was synthesised from *N*-(3-aminopropyl)-2-(4-(octylsulfonamido)-[1,1′:4′,1″-terphenyl]-3-yl)-2-oxoacetamide hydrochloride **27d** (0.29 g, 0.49 mmol), *N*,*N*′-di-Boc-1*H*-pyrazole-1-carboxamidine (0.20 g, 0.64 mmol) and triethylamine (0.17 mL, 1.23 mmol) following **general synthetic procedure G**. The product was obtained as a yellow solid (0.27 g, 70%); mp 81.5–81.8 °C; ^1^H NMR (400 MHz, CDCl_3_): δ 11.47 (s, 1H, NH), 10.64 (s, 1H, NH), 8.68–8.60 (m, 2H, NH, ArH), 8.54 (bs, 1H, NH), 7.91–7.85 (m, 2H, ArH), 7.71–7.60 (m, 6H, ArH), 7.50–7.43 (m, 2H, ArH), 7.40–7.34 (m, 1H, ArH), 3.57 (q, *J* = 6.3 Hz, 2H, CH_2_), 3.47 (q, *J* = 6.3 Hz, 2H, CH_2_), 3.22–3.14 (m, 2H, CH_2_), 1.87–1.77 (m, 4H, CH_2_), 1.50 (s, 9H, CH_3_), 1.38 (s, 9H, CH_3_), 1.43–1.17 (m, 10H, CH_2_), 0.86 (t, *J* = 7.1 Hz, 3H, CH_3_); ^13^C NMR (100 MHz, CDCl_3_): δ 192.5 (CO), 163.7 (CO), 163.0 (CN), 157.4 (CO), 153.3 (CO), 141.2 (ArC), 140.8 (ArC), 140.6 (ArC), 138.0 (ArC), 135.1 (ArC), 134.8 (ArCH), 133.4 (ArCH), 129.0 (ArCH), 127.9 (ArCH), 127.7 (ArCH), 127.3 (ArCH), 127.2 (ArCH), 119.0 (ArC), 118.4 (ArCH), 83.8 (C), 79.8 (C), 52.8 (CH_2_), 37.3 (CH_2_), 35.9 (CH_2_), 31.8 (CH_2_), 30.1 (CH_2_), 29.1 (CH_2_), 29.0 (CH_2_), 28.4 (CH_2_), 28.3 (CH_3_), 28.2 (CH_3_), 23.6 (CH_2_), 22.7 (CH_2_), 14.2 (CH_3_); IR (ATR): *ν*_max_ 3322, 2929, 1720, 1638, 1572, 1482, 1408, 1367, 1328, 1285, 1254, 1228, 1194, 1130, 1051, 1026, 1007, 979, 907, 857, 828, 808, 764, 697, 673 cm^−1^; HRMS (+ESI): Found *m*/*z* 792.4009 [M+H]^+^, C_42_H_58_N_5_O_8_S required 792.4001.

##### (*E*)-1-*tert*-Butyl-*N*-(*N*′-((*tert*-butyloxidanyl)carbonyl)-*N*-(3-(2-(2-(dodecylsulfonamido)phenyl)-2-oxoacetamido)propyl)carbamimidoyl)-1-oxidanecarboxamide (**31a**)

The titled compound was synthesised from *N*-(3-aminopropyl)-2-(2-(dodecylsulfonamido)phenyl)-2-oxoacetamide hydrochloride **28a** (0.25 g, 0.50 mmol), *N*,*N*′-di-Boc-1*H*-pyrazole-1-carboxamidine (0.19 g, 0.63 mmol) and triethylamine (0.18 mL, 1.26 mmol) following **general synthetic procedure G**. The product was obtained as a yellow oil (0.31 g, 87%); ^1^H NMR (600 MHz, CDCl_3_): δ 11.46 (s, 1H, NH), 10.62 (s, 1H, NH), 8.65 (bs, 1H, NH), 8.55 (t, *J* = 6.4 Hz, 1H, NH), 8.24 (dd, *J* = 8.1, 1.5 Hz, 1H, ArH), 7.77 (dd, *J* = 8.4, 0.7 Hz, 1H, ArH), 8.60–8.56 (m, 1H, ArH), 7.16–7.12 (m, 1H, ArH), 3.67–3.56 (m, 2H, CH_2_), 3.46 (q, *J* = 6.3 Hz, 2H, CH_2_), 3.16–3.10 (m, 2H, CH_2_), 1.86–1.75 (m, 4H, CH_2_), 1.50 (s, 9H, CH_3_), 1.37 (s, 9H, CH_3_), 1.40–1.19 (m, 18H, CH_2_), 0.87 (t, *J* = 7.1 Hz, 3H, CH_3_); ^13^C NMR (150 MHz, CDCl_3_): δ 192.8 (CO), 164.0 (CO), 162.3 (CN), 157.0 (CO), 153.2 (CO), 142.0 (ArC), 136.4 (ArCH), 135.1 (ArCH), 122.6 (ArCH), 118.8 (ArC), 118.0 (ArCH), 84.2 (C), 80.5 (C), 52.7 (CH_2_), 37.9 (CH_2_), 35.8 (CH_2_), 32.0 (CH_2_), 29.9 (CH_2_), 29.7 (CH_2_), 29.7 (CH_2_), 29.6 (CH_2_), 29.4 (CH_2_), 29.4 (CH_2_), 29.1 (CH_2_), 28.3 (CH_2_), 28.2 (CH_3_), 28.2 (CH_3_), 23.5 (CH_2_), 22.8 (CH_2_), 14.2 (CH_3_); IR (ATR): *ν*_max_ 3327, 3194, 2926, 2855, 1722, 1640, 1574, 1491, 1452, 1410, 1328, 1283, 1219, 1131, 1050, 978, 913, 753, 671 cm^−1^; HRMS (+ESI): Found *m*/*z* 696.4000 [M+H]^+^, C_34_H_58_N_5_O_8_S required 696.4001.

##### (*E*)-1-*tert*-Butyl-*N*-(*N*′-((*tert*-butyloxidanyl)carbonyl)-*N*-(3-(2-(5-bromo-2-(dodecylsulfonamido) phenyl)-2-oxoacetamido)propyl)carbamimidoyl)-1-oxidanecarboxamide (**31b**)

The titled compound was synthesised from *N*-(3-aminopropyl)-2-(5-bromo-2-(dodecylsulfonamido) phenyl)-2-oxoacetamide hydrochloride **28b** (0.17 g, 0.29 mmol), *N*,*N*′-di-Boc-1*H*-pyrazole-1-carboxamidine (0.13 g, 0.43 mmol) and triethylamine (0.10 mL, 0.73 mmol) following **general synthetic procedure G**. The product was obtained as a yellow oil (0.12 g, 55%); ^1^H NMR (400 MHz, CDCl_3_): δ 11.46 (s, 1H, NH), 10.51 (s, 1H, NH), 8.68 (t, *J* = 6.4 Hz, 1H, NH), 8.52 (t, *J* = 6.4 Hz, 1H, NH), 8.45 (d, *J* = 2.1 Hz, 1H, ArH), 7.71–7.64 (m, 2H, ArH), 3.54 (q, *J* = 6.4 Hz, 2H, CH_2_), 3.44 (q, *J* = 6.2 Hz, 2H, CH_2_), 3.15–3.07 (m, 2H, CH_2_), 1.83–1.72 (m, 4H, CH_2_), 1.49 (s, 9H, CH_3_), 1.37 (s, 9H, CH_3_), 1.41–1.17 (m, 18H, CH_2_), 0.86 (t, *J* = 7.1 Hz, 3H, CH_3_); ^13^C NMR (100 MHz, CDCl_3_): δ 191.4 (CO), 163.0 (CO), 157.4 (CO), 156.3 (CN), 153.3 (CO), 141.1 (ArC), 139.1 (ArCH), 137.3 (ArCH), 120.1 (ArC), 119.6 (ArCH), 115.1 (ArC), 83.8 (C), 79.8 (C), 52.9 (CH_2_), 37.2 (CH_2_), 35.9 (CH_2_), 32.0 (CH_2_), 30.0 (CH_2_), 29.7 (CH_2_), 29.7 (CH_2_), 29.6 (CH_2_), 29.4 (CH_2_), 29.3 (CH_2_), 29.1 (CH_2_), 28.2 (CH_3_), 28.2 (CH_2_), 28.2 (CH_3_), 23.5 (CH_2_), 22.8 (CH_2_), 14.2 (CH_3_); IR (ATR): *ν*_max_ 3327, 2925, 2854, 2323, 1718, 1637, 1617, 1560, 1541, 1522, 1476, 1457, 1411, 1390, 1367, 1327, 1285, 1253, 1227, 1153, 1132, 1050, 1026, 979, 906, 808, 768, 712, 653 cm^−1^; HRMS (+ESI): Found *m*/*z* 774.3108 [M+H]^+^, C_34_H_57_BrN_5_O_8_S required 774.3106.

##### (*E*)-1-*tert*-Butyl-*N*-(*N*′-((*tert*-butyloxidanyl)carbonyl)-*N*-(3-(2-(4-(dodecylsulfonamido)-[1,1′-biphenyl]-3-yl)-2-oxoacetamido)propyl)carbamimidoyl)-1-oxidanecarboxamide (**31c**)

The titled compound was synthesised from *N*-(3-aminopropyl)-2-(4-(dodecylsulfonamido)-[1,1′-biphenyl]-3-yl)-2-oxoacetamide hydrochloride **28c** (0.15 g, 0.27 mmol), *N*,*N*′-di-Boc-1*H*-pyrazole-1-carboxamidine (0.17 g, 0.55 mmol) and triethylamine (92 μL, 0.66 mmol) following **general synthetic procedure G**. The product was obtained as a yellow solid (0.17 g, 83%); mp 63.6–63.8 °C; ^1^H NMR (600 MHz, CDCl_3_): δ 11.47 (s, 1H, NH), 10.62 (s, 1H, NH), 8.63 (t, *J* = 6.6 Hz, 1H, NH), 8.56 (d, *J* = 2.2 Hz, 1H, ArH), 8.52 (t, *J* = 6.2 Hz, 1H, NH), 7.86 (d, *J* = 8.7 Hz, 1H, ArH), 7.82 (dd, *J* = 8.7, 2.2 Hz, 1H, ArH), 7.58–7.55 (m, 2H, ArH), 7.46–7.42 (m, 2H, ArH), 7.38–7.34 (m, 1H, ArH), 3.55 (q, *J* = 6.4 Hz, 2H, CH_2_), 3.46 (q, *J* = 6.3 Hz, 2H, CH_2_), 3.19–3.15 (m, 2H, CH_2_), 1.84–1.77 (m, 4H, CH_2_), 1.49 (s, 9H, CH_3_), 1.37 (s, 9H, CH_3_), 1.41–1.34 (m, 2H, CH_2_), 1.31–1.19 (m, 16H, CH_2_), 0.87 (t, *J* = 7.1 Hz, 3H, CH_3_); ^13^C NMR (150 MHz, CDCl_3_): δ 192.6 (CO), 163.7 (CO), 163.1 (CN), 157.4 (CO), 153.3 (CO), 141.2 (ArC), 139.1 (ArC), 135.6 (ArC), 135.0 (ArCH), 133.5 (ArCH), 129.1 (ArCH), 127.9 (ArCH), 126.9 (ArCH), 118.9 (ArC), 118.3 (ArCH), 83.7 (C), 79.8 (C), 52.8 (CH_2_), 37.2 (CH_2_), 35.8 (CH_2_), 32.0 (CH_2_), 30.1 (CH_2_), 29.7 (CH_2_), 29.7 (CH_2_), 29.6 (CH_2_), 29.4 (CH_2_), 29.4 (CH_2_), 29.1 (CH_2_), 28.3 (CH_3_), 28.2 (CH_2_), 28.2 (CH_3_), 23.6 (CH_2_), 22.8 (CH_2_), 14.2 (CH_3_); IR (ATR): *ν*_max_ 3326, 2926, 2854, 1719, 1636, 1615, 1570, 1507, 1483, 1411, 1366, 1326, 1285, 1253, 1228, 1130, 1051, 1025, 979, 907, 854, 807, 760, 698, 681 cm^−1^; HRMS (+ESI): Found *m*/*z* 772.4320 [M+H]^+^, C_40_H_62_N_5_O_8_S required 772.4314.

##### (*E*)-1-*tert*-Butyl-*N*-(*N*′-((*tert*-butyloxidanyl)carbonyl)-*N*-(3-(2-(2-(hexadecylsulfonamido)phenyl)-2-oxoacetamido)propyl)carbamimidoyl)-1-oxidanecarboxamide (**32a**)

The titled compound was synthesised from *N*-(3-aminopropyl)-2-(2-(hexadecylsulfonamido)phenyl)-2-oxoacetamide hydrochloride **29a** (0.16 g, 0.28 mmol), *N*,*N*′-di-Boc-1*H*-pyrazole-1-carboxamidine (0.11 g, 0.36 mmol) and triethylamine (0.10 mL, 0.72 mmol) following **general synthetic procedure G**. The product was obtained as a yellow oil (0.14 g, 67%); ^1^H NMR (400 MHz, CDCl_3_): δ 11.46 (s, 1H, NH), 10.63 (s, 1H, NH), 8.58 (t, *J* = 6.4 Hz, 1H, NH), 8.52 (t, *J* = 6.3 Hz, 1H, NH), 8.26 (dd, *J* = 8.0, 1.5 Hz, 1H, ArH), 7.77 (dd, *J* = 8.4, 0.7 Hz, 1H, ArH), 7.62–7.55 (m, 1H, ArH), 7.17–7.11 (m, 1H, ArH), 3.55 (q, *J* = 6.6 Hz, 2H, CH_2_), 3.44 (q, *J* = 6.1 Hz, 2H, CH_2_), 3.17–3.10 (m, 2H, CH_2_), 1.84–1.73 (m, 4H, CH_2_), 1.49 (s, 9H, CH_3_), 1.35 (s, 9H, CH_3_), 1.39–1.18 (m, 26H, CH_2_), 0.87 (t, *J* = 7.0 Hz, 3H, CH_3_); ^13^C NMR (100 MHz, CDCl_3_): δ 192.8 (CO), 163.9 (CO), 163.1 (CN), 157.4 (CO), 153.4 (CO), 142.2 (ArC), 136.5 (ArCH), 135.2 (ArCH), 122.5 (ArCH), 118.5 (ArC), 117.8 (ArCH), 83.7 (C), 79.7 (C), 52.7 (CH_2_), 37.2 (CH_2_), 35.8 (CH_2_), 32.1 (CH_2_), 30.1 (CH_2_), 29.8 (CH_2_), 29.8 (CH_2_), 29.8 (CH_2_), 29.8 (CH_2_), 29.8 (CH_2_), 29.7 (CH_2_), 29.6 (CH_2_), 29.5 (CH_2_), 29.4 (CH_2_), 29.1 (CH_2_), 28.3 (CH_2_), 28.2 (CH_3_), 28.2 (CH_3_), 23.5 (CH_2_), 22.8 (CH_2_), 14.3 (CH_3_); IR (ATR): *ν*_max_ 3327, 3195, 2923, 2853, 2340, 1723, 1640, 1614, 1574, 1491, 1452, 1409, 1330, 1283, 1221, 1133, 1050, 914, 753, 672 cm^−1^; HRMS (+ESI): Found *m*/*z* 752.4626 [M+H]^+^, C_38_H_66_N_5_O_8_S required 752.4627.

##### (*E*)-1-*tert*-Butyl-*N*-(*N*′-((*tert*-butyloxidanyl)carbonyl)-*N*-(3-(2-(5-bromo-2-(hexadecylsulfonamido) phenyl)-2-oxoacetamido)propyl)carbamimidoyl)-1-oxidanecarboxamide (**32b**)

The titled compound was synthesised from *N*-(3-aminopropyl)-2-(5-bromo-2-(hexadecylsulfonamido) phenyl)-2-oxoacetamide hydrochloride **29b** (0.24 g, 0.38 mmol), *N*,*N*′-di-Boc-1*H*-pyrazole-1-carboxamidine (0.14 g, 0.46 mmol) and triethylamine (0.13 mL, 0.95 mmol) following **general synthetic procedure G**. The product was obtained as a yellow oil (0.16 g, 51%); ^1^H NMR (600 MHz, CDCl_3_): δ 11.47 (s, 1H, NH), 10.52 (s, 1H, NH), 8.69 (t, *J* = 6.4 Hz, 1H, NH), 8.53 (t, *J* = 6.4 Hz, 1H, NH), 8.46 (d, *J* = 2.2 Hz, 1H, ArH), 7.71–7.65 (m, 2H, ArH), 3.54 (q, *J* = 6.4 Hz, 2H, CH_2_), 3.44 (q, *J* = 6.2 Hz, 2H, CH_2_), 3.15–3.08 (m, 2H, CH_2_), 1.82–1.73 (m, 4H, CH_2_), 1.50 (s, 9H, CH_3_), 1.37 (s, 9H, CH_3_), 1.40–1.32 (m, 2H, CH_2_), 1.31–1.19 (m, 24H, CH_2_), 0.87 (t, *J* = 7.1 Hz, 3H, CH_3_); ^13^C NMR (150 MHz, CDCl_3_): δ 191.4 (CO), 163.0 (CO), 163.0 (CN), 157.5 (CO), 153.3 (CO), 141.1 (ArC), 139.2 (ArCH), 137.3 (ArCH), 120.0 (ArC), 119.6 (ArCH), 115.1 (ArC), 83.8 (C), 79.8 (C), 52.9 (CH_2_), 37.2 (CH_2_), 35.9 (CH_2_), 32.1 (CH_2_), 30.0 (CH_2_), 29.8 (CH_2_), 29.8 (CH_2_), 29.8 (CH_2_), 29.8 (CH_2_), 29.8 (CH_2_), 29.7 (CH_2_), 29.6 (CH_2_), 29.5 (CH_2_), 29.4 (CH_2_), 29.1 (CH_2_), 28.3 (CH_3_), 28.2 (CH_2_), 28.2 (CH_3_), 23.5 (CH_2_), 22.8 (CH_2_), 14.3 (CH_3_); IR (ATR): *ν*_max_ 3326, 2924, 2854, 2097, 1721, 1642, 1569, 1477, 1413, 1327, 1285, 1227, 1131, 1050, 979, 906, 808, 767, 713 cm^−1^; HRMS (+ESI): Found *m*/*z* 830.3731 [M+H]^+^, C_36_H_65_BrN_5_O_8_S required 830.3732.

##### (*E*)-1-*tert*-Butyl-*N*-(*N*′-((*tert*-butyloxidanyl)carbonyl)-*N*-(3-(2-(4-(hexadecylsulfonamido)-[1,1′-biphenyl]-3-yl)-2-oxoacetamido)propyl)carbamimidoyl)-1-oxidanecarboxamide (**32c**)

The titled compound was synthesised from *N*-(3-aminopropyl)-2-(4-(hexadecylsulfonamido)-[1,1′-biphenyl]-3-yl)-2-oxoacetamide hydrochloride **29c** (0.20 g, 0.31 mmol), *N*,*N*′-di-Boc-1*H*-pyrazole-1-carboxamidine (0.12 g, 0.39 mmol) and triethylamine (0.10 mL, 0.79 mmol) following **general synthetic procedure G**. The product was obtained as a yellow oil (0.17 g, 66%); ^1^H NMR (600 MHz, CDCl_3_): δ 11.47 (s, 1H, NH), 10.62 (s, 1H, NH), 8.62 (t, *J* = 6.3 Hz, 1H, NH), 8.60 (bs, 1H, NH), 8.55 (d, *J* = 2.1 Hz, 1H, ArH), 7.86 (d, *J* = 8.7 Hz, 1H, ArH), 7.82 (dd, *J* = 8.7, 2.2 Hz, 1H, ArH), 7.58–7.55 (m, 2H, ArH), 7.46–7.42 (m, 2H, ArH), 7.38–7.34 (m, 1H, ArH), 3.64–3.54 (m, 2H, CH_2_), 3.46 (q, *J* = 6.4 Hz, 2H, CH_2_), 3.19–3.15 (m, 2H, CH_2_), 1.85–1.78 (m, 4H, CH_2_), 1.50 (s, 9H, CH_3_), 1.38 (s, 9H, CH_3_), 1.41–1.33 (m, 2H, CH_2_), 1.32–1.20 (m, 24H, CH_2_), 0.87 (t, *J* = 7.2 Hz, 3H, CH_3_); ^13^C NMR (150 MHz, CDCl_3_): δ 192.6 (CO), 163.8 (CO), 163.8 (CN), 157.2 (CO), 153.3 (CO), 141.1 (ArC), 139.1 (ArC), 135.7 (ArC), 134.9 (ArCH), 133.5 (ArCH), 129.1 (ArCH), 127.9 (ArCH), 127.0 (ArCH), 119.2 (ArC), 118.4 (ArCH), 84.0 (C), 80.3 (C), 52.8 (CH_2_), 37.6 (CH_2_), 35.9 (CH_2_), 32.1 (CH_2_), 30.0 (CH_2_), 29.8 (CH_2_), 29.8 (CH_2_), 29.8 (CH_2_), 29.7 (CH_2_), 29.6 (CH_2_), 29.5 (CH_2_), 29.4 (CH_2_), 29.1 (CH_2_), 28.3 (CH_2_), 28.2 (CH_3_), 28.2 (CH_3_), 23.5 (CH_2_), 22.8 (CH_2_), 14.3 (CH_3_); IR (ATR): *ν*_max_ 3326, 3194, 2922, 2852, 2321, 2090, 1720, 1638, 1575, 1482, 1410, 1326, 1283, 1130, 1050, 978, 907, 854, 806, 759, 697 cm^−1^; HRMS (+ESI): Found *m*/*z* 828.4939 [M+H]^+^, C_44_H_70_N_5_O_8_S required 828.4940.

**General synthetic procedure H for guanidinium hydrochloride salts**

Trifluoroacetic acid (2 mL) was added to a stirring solution of Boc-protected guanidine glyoxamide (1.0 equivalent) in dichloromethane (2 mL). The resulting reaction mixture was stirred at room temperature for 3 h. After the completion of the reaction, the reaction solvent was removed under reduced pressure and the residue was washed thrice with diethyl ether. To the residue redissolved in dichloromethane (2 mL) was added 4 M HCl in dioxane (2 mL), and the resulting reaction mixture was stirred at room temperature for 30 min. After the completion of the reaction, the reaction solvent was removed under reduced pressure. The residue was washed thrice with diethyl ether and freeze-dried to afford the product.

##### *N*-(3-Guanidinopropyl)-2-(4-(octylsulfonamido)-[1,1′:4′,1″-terphenyl]-3-yl)-2-oxoacetamide hydrochloride (**33d**)

The titled compound was synthesised from (*E*)-1-*tert*-butyl-*N*-(*N*′-((*tert*-butyloxidanyl)carbonyl)-*N*-(3-(2-(4-(octylsulfonamido)-[1,1′:4′,1″-terphenyl]-3-yl)-2-oxoacetamido)propyl)carbamimidoyl)-1-oxidanecarboxamide **30d** (0.15 g, 0.19 mmol) following **general synthetic procedure H**. The product was obtained as a yellow solid (58 mg, 48%); mp 49.6–50.1 °C; ^1^H NMR (600 MHz, DMSO-*d*_6_): δ 10.22 (s, 1H, NH), 9.03 (t, *J* = 5.8 Hz, 1H, NH), 8.12–8.02 (m, 2H, ArH), 7.81 (d, *J* = 8.5 Hz, 2H, ArH), 7.76–7.69 (m, 5H, NH, ArH), 7.66 (d, *J* = 8.7 Hz, 1H, ArH), 7.57–6.83 (m, 7H, NH, ArH), 3.32 (q, *J* = 6.7 Hz, 2H, CH_2_)_,_ 3.29–3.25 (m, 2H, CH_2_), 3.21 (q, *J* = 6.7 Hz, 2H, CH_2_), 1.79–1.72 (m, 2H, CH_2_), 1.72–1.65 (m, 2H, CH_2_), 1.38–1.31 (m, 2H, CH_2_), 1.27–1.16 (m, 8H, CH_2_), 0.82 (t, *J* = 7.1 Hz, 3H, CH_3_); ^13^C NMR (150 MHz, DMSO-*d*_6_): δ 192.5 (CO), 163.8 (CO), 156.4 (CN), 139.6 (ArC), 139.4 (ArC), 138.4 (ArC), 137.2 (ArC), 135.0 (ArC), 133.0 (ArCH), 130.4 (ArCH), 129.0 (ArCH), 127.7 (ArCH), 127.4 (ArCH), 126.9 (ArCH), 126.6 (ArCH), 124.4 (ArC), 121.7 (ArCH), 51.5 (CH_2_), 38.4 (CH_2_), 36.1 (CH_2_), 31.1 (CH_2_), 28.4 (CH_2_), 28.4 (CH_2_), 28.3 (CH_2_), 27.3 (CH_2_), 22.9 (CH_2_), 22.0 (CH_2_), 13.9 (CH_3_); IR (ATR): *ν*_max_ 3808, 3273, 3162, 2924, 2854, 2359, 1650, 1529, 1497, 1482, 1400, 1333, 1272, 1196, 1142, 1076, 1006, 917, 827, 764, 719, 695, 674 cm^−1^; HRMS (+ESI): Found *m*/*z* 592.2956 [M+H]^+^, C_32_H_42_N_5_O_4_S required 592.2952.

##### 2-(2-(Dodecylsulfonamido)phenyl)-*N*-(3-guanidinopropyl)-2-oxoacetamide hydrochloride (**34a**)

The titled compound was synthesised from (*E*)-1-*tert*-butyl-*N*-(*N*′-((*tert*-butyloxidanyl)carbonyl)-*N*-(3-(2-(2-(dodecylsulfonamido)phenyl)-2-oxoacetamido)propyl)carbamimidoyl)-1-oxidanecarboxamide **31a** (0.11 g, 0.16 mmol) following **general synthetic procedure H**. The product was obtained as a pale yellow solid (71 g, 82%); mp 70.0–72.8 °C; ^1^H NMR (600 MHz, DMSO-*d*_6_): δ 10.27 (s, 1H, NH), 8.98 (t, *J* = 5.7 Hz, 1H, NH), 7.78 (dd, *J* = 7.9, 1.5 Hz, 1H, ArH), 7.74 (t, *J* = 5.8 Hz, 1H, NH), 7.72–7.68 (m, 1H, ArH), 7.63–6.78 (m, 6H, NH, ArH), 3.31–3.23 (m, 4H, CH_2_), 3.18 (q, *J* = 6.7 Hz, 2H, CH_2_), 1.76–1.69 (m, 2H, CH_2_), 1.68–1.61 (m, 2H, CH_2_), 1.35–1.15 (m, 18H, CH_2_), 0.85 (t, *J* = 7.2 Hz, 3H, CH_3_); ^13^C NMR (150 MHz, DMSO-*d*_6_): δ 193.4 (CO), 164.3 (CO), 157.0 (CN), 139.6 (ArC), 135.5 (ArCH), 133.2 (ArCH), 123.6 (ArCH), 122.4 (ArC), 120.1 (ArCH), 51.4 (CH_2_), 38.4 (CH_2_), 36.0 (CH_2_), 31.3 (CH_2_), 29.0 (CH_2_), 28.9 (CH_2_), 28.7 (CH_2_), 28.7 (CH_2_), 28.4 (CH_2_), 28.3 (CH_2_), 27.2 (CH_2_), 22.9 (CH_2_), 22.1 (CH_2_), 14.0 (CH_3_); IR (ATR): *ν*_max_ 3329, 3249, 3157, 2917, 2850, 1635, 1572, 1528, 1492, 1451, 1398, 1337, 1224, 1149, 1065, 912, 822, 750, 673 cm^−1^; HRMS (+ESI): Found *m*/*z* 496.2951 [M+H]^+^, C_24_H_42_N_5_O_4_S required 496.2952.

##### 2-(5-Bromo-2-(dodecylsulfonamido)phenyl)-*N*-(3-guanidinopropyl)-2-oxoacetamide hydrochloride (**34b**)

The titled compound was synthesised from (*E*)-1-*tert*-butyl-*N*-(*N*′-((*tert*-butyloxidanyl)carbonyl)-*N*-(3-(2-(5-bromo-2-(dodecylsulfonamido)phenyl)-2-oxoacetamido)propyl)carbamimidoyl)-1-oxidanecarboxamide **31b** (71 mg, 0.092 mmol) following **general synthetic procedure H**. The product was obtained as a white solid (47 mg, 84%); mp 50.0–50.1 °C; ^1^H NMR (600 MHz, DMSO-*d*_6_): δ 10.08 (s, 1H, NH), 8.94 (bs, 1H, NH), 7.93–7.79 (m, 2H, ArH), 7.70–6.72 (m, 6H, NH, ArH), 3.26 (q, *J* = 6.8 Hz, 2H, CH_2_), 3.21–3.14 (m, 4H, CH_2_), 1.76–1.68 (m, 2H, CH_2_), 1.67–1.59 (m, 2H, CH_2_), 1.34–1.15 (m, 18H, CH_2_), 0.85 (t, *J* = 7.2 Hz, 3H, CH_3_); ^13^C NMR (150 MHz, DMSO-*d*_6_): δ 190.4 (CO), 163.0 (CO), 156.9 (CN), 137.6 (ArC), 137.0 (ArCH), 134.2 (ArCH), 127.6 (ArC), 124.2 (ArCH), 116.2 (ArC), 51.4 (CH_2_), 38.3 (CH_2_), 36.1 (CH_2_), 31.3 (CH_2_), 29.0 (CH_2_), 28.9 (CH_2_), 28.7 (CH_2_), 28.7 (CH_2_), 28.4 (CH_2_), 28.3 (CH_2_), 27.3 (CH_2_), 22.8 (CH_2_), 22.1 (CH_2_), 14.0 (CH_3_); IR (ATR): *ν*_max_ 3171, 2919, 2851, 2360, 1642, 1562, 1530, 1479, 1385, 1337, 1293, 1223, 1198, 1147, 907, 823, 710, 654 cm^−1^; HRMS (+ESI): Found *m*/*z* 574.2061 [M+H]^+^, C_24_H_41_BrN_5_O_4_S required 574.2057.

##### 2-(4-(Dodecylsulfonamido)-[1,1′-biphenyl]-3-yl)-*N*-(3-guanidinopropyl)-2-oxoacetamide hydrochloride (**34c**)

The titled compound was synthesised from (*E*)-1-*tert*-butyl-*N*-(*N*′-((*tert*-butyloxidanyl)carbonyl)-*N*-(3-(2-(4-(dodecylsulfonamido)-[1,1′-biphenyl]-3-yl)-2-oxoacetamido)propyl)carbamimidoyl)-1-oxidanecarboxamide **31c** (82 mg, 0.11 mmol) following **general synthetic procedure H**. The product was obtained as a yellow solid (41 mg, 63%); mp 73.2–73.5 °C; ^1^H NMR (600 MHz, DMSO-*d*_6_): δ 10.21 (s, 1H, NH), 9.02 (bs, 1H, NH), 8.03–7.98 (m, 2H, ArH), 7.71 (bs, 1H, NH), 7.66–7.60 (m, 3H, ArH), 7.56–6.78 (m, 7H, NH, ArH), 3.30 (q, *J* = 6.7 Hz, 2H, CH_2_), 3.28–3.23 (m, 2H, CH_2_), 2.22–2.15 (m, 2H, CH_2_), 1.78–1.71 (m, 2H, CH_2_), 1.70–1.63 (m, 2H, CH_2_), 1.36–1.30 (m, 2H, CH_2_), 1.28–1.14 (m, 16H, CH_2_), 0.84 (t, *J* = 7.2 Hz, 3H, CH_3_); ^13^C NMR (150 MHz, DMSO-*d*_6_): δ 192.6 (CO), 163.8 (CO), 157.0 (CN), 138.3 (ArC), 138.2 (ArC), 135.5 (ArC), 133.2 (ArCH), 130.5 (ArCH), 129.2 (ArCH), 127.9 (ArCH), 126.4 (ArCH), 124.3 (ArC), 121.6 (ArCH), 51.4 (CH_2_), 38.4 (CH_2_), 36.1 (CH_2_), 31.3 (CH_2_), 29.0 (CH_2_), 28.9 (CH_2_), 28.7 (CH_2_), 28.7 (CH_2_), 28.4 (CH_2_), 28.4 (CH_2_), 27.3 (CH_2_), 22.9 (CH_2_), 22.1 (CH_2_), 14.0 (CH_3_); IR (ATR): *ν*_max_ 3365, 3167, 2919, 2850, 2359, 1767, 1736, 1676, 1626, 1582, 1527, 1512, 1487, 1467, 1396, 1376, 1347, 1309, 1266, 1214, 1200, 1175, 1139, 1094, 925, 910, 853, 824, 780, 759, 720, 683, 651 cm^−1^; HRMS (+ESI): Found *m*/*z* 572.3267 [M+H]^+^, C_30_H_46_N_5_O_4_S required 572.3265.

##### *N*-(3-Guanidinopropyl)-2-(2-(hexadecylsulfonamido)phenyl)-2-oxoacetamide hydrochloride (**35a**)

The titled compound was synthesised from (*E*)-1-*tert*-butyl-*N*-(*N*′-((*tert*-butyloxidanyl)carbonyl)-*N*-(3-(2-(2-(hexadecylsulfonamido)phenyl)-2-oxoacetamido)propyl)carbamimidoyl)-1-oxidanecarboxamide **32a** (61 mg, 0.081 mmol) following **general synthetic procedure H**. The product was obtained as a white sticky solid (31 mg, 65%); ^1^H NMR (600 MHz, DMSO-*d*_6_): δ 10.27 (s, 1H, NH), 8.97 (t, *J* = 5.8 Hz, 1H, NH), 7.78 (dd, *J* = 7.9, 1.5 Hz, 1H, ArH), 7.74–7.68 (m, 2H, NH, ArH), 7.62–6.78 (m, 6H, NH, ArH), 3.31–3.23 (m, 4H, CH_2_), 3.18 (q, *J* = 6.7 Hz, 2H, CH_2_), 1.77–1.70 (m, 2H, CH_2_), 1.68–1.61 (m, 2H, CH_2_), 1.36–1.15 (m, 26H, CH_2_), 0.85 (t, *J* = 7.1 Hz, 3H, CH_3_); ^13^C NMR (150 MHz, DMSO-*d*_6_): δ 193.4 (CO), 164.3 (CO), 156.9 (CN), 139.6 (ArC), 135.5 (ArCH), 133.2 (ArCH), 123.6 (ArCH), 122.5 (ArC), 120.1 (ArCH), 51.4 (CH_2_), 38.4 (CH_2_), 36.0 (CH_2_), 31.3 (CH_2_), 29.0 (CH_2_), 29.0 (CH_2_), 29.0 (CH_2_), 29.0 (CH_2_), 29.0 (CH_2_), 28.9 (CH_2_), 28.7 (CH_2_), 28.7 (CH_2_), 28.4 (CH_2_), 28.3 (CH_2_), 27.3 (CH_2_), 22.9 (CH_2_), 22.1 (CH_2_), 14.0 (CH_3_); IR (ATR): *ν*_max_ 3343, 3245, 3173, 2915, 2849, 2290, 2106, 1626, 1573, 1543, 1493, 1453, 1398, 1339, 1219, 1150, 1070, 913, 885, 821, 750, 675 cm^−1^; HRMS (+ESI): Found *m*/*z* 552.3578 [M+H]^+^, C_28_H_50_N_5_O_4_S required 552.3578.

##### 2-(5-Bromo-2-(hexadecylsulfonamido)phenyl)-*N*-(3-guanidinopropyl)-2-oxoacetamide hydrochloride (**35b**)

The titled compound was synthesised from (*E*)-1-*tert*-butyl-*N*-(*N*′-((*tert*-butyloxidanyl)carbonyl)-*N*-(3-(2-(5-bromo-2-(hexadecylsulfonamido)phenyl)-2-oxoacetamido)propyl)carbamimidoyl)-1-oxidanecarboxamide **32b** (85 mg, 0.10 mmol) following **general synthetic procedure H**. The product was obtained as a yellow solid (66 mg, 97%); mp 64.3–66.6 °C; ^1^H NMR (400 MHz, DMSO-*d*_6_): δ 10.08 (s, 1H, NH), 8.95 (t, *J* = 5.8 Hz, 1H, NH), 7.90–7.82 (m, 2H, ArH), 7.66 (bs, 1H, NH), 7.57–6.81 (m, 5H, NH, ArH), 3.31–3.11 (m, 6H, CH_2_), 1.79–1.57 (m, 4H, CH_2_), 1.39–1.13 (m, 26H, CH_2_), 0.85 (t, *J* = 7.1 Hz, 3H, CH_3_); ^13^C NMR (100 MHz, DMSO-*d*_6_): δ 190.4 (CO), 163.0 (CO), 156.9 (CN), 137.7 (ArC), 137.0 (ArCH), 134.2 (ArCH), 127.6 (ArC), 124.2 (ArCH), 116.2 (ArC), 51.4 (CH_2_), 38.3 (CH_2_), 36.1 (CH_2_), 31.3 (CH_2_), 29.0 (CH_2_), 29.0 (CH_2_), 28.9 (CH_2_), 28.7 (CH_2_), 28.7 (CH_2_), 28.4 (CH_2_), 28.3 (CH_2_), 27.3 (CH_2_), 22.8 (CH_2_), 22.1 (CH_2_), 14.0 (CH_3_); IR (ATR): *ν*_max_ 3342, 3165, 2918, 2850, 2342, 2104, 1642, 1529, 1478, 1385, 1337, 1197, 1148, 905, 822, 718 cm^−1^; HRMS (+ESI): Found *m*/*z* 630.2683 [M+H]^+^, C_28_H_49_BrN_5_O_4_S required 630.2683.

##### *N*-(3-Guanidinopropyl)-2-(4-(hexadecylsulfonamido)-[1,1′-biphenyl]-3-yl)-2-oxoacetamide hydrochloride (**35c**)

The titled compound was synthesised from (*E*)-1-*tert*-butyl-*N*-(*N*′-((*tert*-butyloxidanyl)carbonyl)-*N*-(3-(2-(4-(hexadecylsulfonamido)-[1,1′-biphenyl]-3-yl)-2-oxoacetamido)propyl)carbamimidoyl)-1-oxidanecarboxamide **32c** (75 mg, 0.091 mmol) following **general synthetic procedure H**. The product was obtained as a yellow solid (42 mg, 71%); mp 74.5–77.1 °C; ^1^H NMR (400 MHz, DMSO-*d*_6_): δ 10.21 (s, 1H, NH), 9.01 (t, *J* = 5.8 Hz, 1H, NH), 8.03–7.96 (m, 2H, ArH), 7.71 (t, *J* = 5.9 Hz, 1H, NH), 7.67–7.60 (m, 3H, ArH), 7.55–6.78 (m, 7H, NH, ArH), 3.35–3.16 (m, 6H, CH_2_), 1.80–1.61 (m, 4H, CH_2_), 1.38–1.13 (m, 26H, CH_2_), 0.84 (t, *J* = 7.0 Hz, 3H, CH_3_); ^13^C NMR (100 MHz, DMSO-*d*_6_): δ 192.6 (CO), 163.8 (CO), 156.9 (CN), 138.3 (ArC), 138.2 (ArC), 135.5 (ArC), 133.2 (ArCH), 130.5 (ArCH), 129.2 (ArCH), 127.9 (ArCH), 126.4 (ArCH), 124.3 (ArC), 121.6 (ArCH), 51.4 (CH_2_), 38.4 (CH_2_), 36.1 (CH_2_), 31.3 (CH_2_), 29.0 (CH_2_), 29.0 (CH_2_), 28.9 (CH_2_), 28.7 (CH_2_), 28.7 (CH_2_), 28.4 (CH_2_), 27.3 (CH_2_), 22.8 (CH_2_), 22.1 (CH_2_), 13.9 (CH_3_); IR (ATR): *ν*_max_ 3365, 3164, 2917, 2849, 2343, 2084, 1625, 1525, 1486, 1395, 1345, 1266, 1200, 1138, 924, 851, 759, 720, 682 cm^−1^; HRMS (+ESI): Found *m*/*z* 628.3891 [M+H]^+^, C_34_H_54_N_5_O_4_S required 628.3891.

### 3.2. Minimum Inhibitory Concentration (MIC)

A MIC assay was conducted to evaluate the antimicrobial activity of the synthesised peptidomimetics. In brief, a single colony of bacteria (*S. aureus*, *E. coli* and *P. aeruginosa*) was cultured in trypticase soy broth (TSB; Oxoid, Basingstoke, U.K.) and Muller Hinton broth (MHB; Oxoid, Basingstoke, UK) at 37 °C with shaking at 120 rpm for 18–24 h. The resulting bacterial culture was washed twice with the media with centrifugation after each wash. The bacterial solution was then diluted with fresh media to a turbidity of OD_660_ = 0.1 in TSB (equivalent to 10^8^ colony forming unit (CFU)/mL of bacteria), followed by diluting to 10^6^ CFU/mL in media. A total of 100 µL of the bacterial solution was added to wells of a 96-well polystyrene plate (Costar; Sigma-Aldrich, St. Louis, MO, USA) containing 100 µL serially diluted peptidomimetics, with final concentrations ranging from 1–250 µM. The final concentration of DMSO was at or below 1.0%, and wells with 1.0% DMSO was used as the solvent control. Wells with only media were set as blank, while wells with bacteria and media but no compounds were used as a negative control. Plates wrapped with parafilm were then incubated at 37 °C with shaking at 120 rpm for 18–24 h, and the OD value at 660 nm was measured, using a FLUOstar Omega (BMG Labtech) microplate reader. The MIC value of each compound was determined as the lowest concentration that completely inhibited the growth of bacteria. Each experiment was performed in triplicate and was repeated in three independent experiments.

### 3.3. Biofilm Disruption

Bacterial cultures (*S. aureus* and *E. coli*) were cultured in MHB at 37 °C with shaking at 120 rpm for 24 h. The resulting bacterial culture was washed twice with MHB with centrifugation after each wash. The bacterial solution was then diluted with fresh MHB to a turbidity of OD_660_ = 0.1 in MHB (equivalent to 10^8^ colony forming unit (CFU)/mL of bacteria), followed by diluting to 10^6^ CFU/mL in MHB. A total of 200 µL of the bacterial solution was dispensed to wells in a flat-bottom 96-well plate (Costar). The biofilm was then grown in the plate at 37 °C for 24 h. After incubation, loosely bound cells were washed away with 1× phosphate-buffered saline (PBS, pH 7.4). The biofilms were then supplemented with different concentrations of peptidomimetics dissolved in DMSO and incubated at 37 °C with shaking at 120 rpm for a further 24 h. Biofilms adhered on the plate substratum were quantified, using crystal violet staining as described previously [72,73]. The experiment was performed in triplicate.

### 3.4. Biofilm Inhibition

*S. aureus* was cultured in Muller Hinton broth (MHB; Oxoid) at 37 °C with shaking at 120 rpm for 24 h. The resulting bacterial culture was washed twice with MHB with centrifugation after each wash. The bacterial solution was then diluted with fresh MHB to a turbidity of OD_660_ = 0.1 in MHB (equivalent to 10^8^ colony forming unit (CFU)/mL of bacteria), followed by diluting to 10^6^ CFU/mL in MHB. A total of 100 µL of the bacterial solution was added to wells of a 96-well plate (Costar; Sigma-Aldrich, St. Louis, MO, USA) containing 100 µL serially diluted peptidomimetics. After incubation at 37 °C for 24 h, loosely bound cells were washed away with 1× phosphate-buffered saline (PBS, pH 7.4). Biofilms adhered on the plate substratum were quantified, using crystal violet staining as described previously [72,73]. Untreated bacteria were used as a negative control, where the percentage of biofilm mass reflected 100% biofilm growth. The experiment was performed in triplicate.

### 3.5. Cytoplasmic Membrane Permeability Assay

Membrane potential sensitive dye diSC3–5 (3,3′-dipropylthiadicarbocyanine iodide), which penetrates inside bacterial cells depending on the membrane potential gradient of the cytoplasmic membrane, was used to determine the bacterial cytoplasmic membrane permeability following the procedure previously described [71,74] with slight modification. Bacteria (*S. aureus*) were incubated in MHB with shaking at 37 °C for 16 h. After incubation, the bacteria were washed with 5 mM HEPES, supplemented with 20 mM glucose at pH 7.2 and diluted in the same buffer to an OD_600_ 0.05–0.06 (equivalent to a final concentration of 10^7^ CFU/mL bacteria). The diSC3–5 dye was added at 4 μM to the bacterial suspension. The suspensions were incubated at room temperature in darkness for 1 h for maximum dye take-up by the bacterial cells. A total of 100 mM KCl was then added to balance the K^+^ outside and inside the bacterial cell to prevent the release or further uptake of the dye. Then, 100 μL of bacterial suspension was added to a 96-well microtiter plate containing 100 µL of serially diluted peptidomimetics. The final concentration of DMSO was at or below 1.0%, and wells with 1.0% DMSO were used as a solvent control. 10% DMSO was used as a positive control, while wells with only dye and bacterial cells were set as the negative control. Fluorescence due to the release of dye was measured with a FLUOstar Omega (BMG Labtech) microplate reader at 5 min intervals at an excitation wavelength of 621 nm and an emission wavelength of 670 nm.

### 3.6. Toxicity Assays

The toxicity of the peptidomimetics against normal human lung fibroblasts MRC-5 cells was measured, using an MTT (3-(4,5-dimethylthiazol-2-yl)-2,5-diphenyltetrazolium bromide) (Sigma Aldrich, St. Louis, MO, USA) colorimetric assay. The cells were cultured in minimal essential medium (MEM, Invitrogen) containing 10% foetal bovine serum (FBS). The cells were maintained at 37 °C in 5% CO_2_ as an adherent monolayer and was passaged upon reaching confluence by standard cell culture techniques. MRC-5 cells were seeded at 5000 cells/well in 96-well plates to ensure full confluence (quiescence) and left overnight to adhere to the plate wells. The adherent cells were treated with 1–500 μM of compounds by dissolving in DMSO and serially diluting with media. TY-01 was used as the positive control. The final concentration of DMSO was 0.5% (*v*/*v*). After the 72 h drug incubation, 20 mL stock MTT solution (5 mg/mL) was added, and the cells were incubated for another 3.5–4 h. The MTT solution containing media was carefully aspirated without displacing the purple crystals from the bottom, and 80 mL acid-isopropanol was added to all wells and mixed slowly by shaking with an orbital shaker to dissolve the dark blue crystals. The metabolic activity was detected by spectrophotometric analysis by assessing the read absorbance (590/620 nm) on the EnSight plate reader (PerkinElmer, Waltham, MA, USA), and cell viability was expressed as a percentage of the untreated control cells. The determination of IC_50_ values was performed using GraphPad Prism 7 (GraphPad, San Diego, CA, USA). Each experiment was performed in triplicate and was repeated in three independent experiments.

A total of 20 mL of human blood collected in EDTA coated tubes was transferred into a 50 mL centrifuge tube and centrifuged at 500× *g* (700) for 5 min. The supernatant plasma was discarded and an equal volume of 1× PBS was added. The washing was repeated five times with cells mixed by inversion. The washed pallet was then resuspended in 1:10 with PBS to yield an RBC concentration of ~5 × 10^8^ RBC/mL. The peptidomimetics were serially diluted with PBS, with final concentrations ranging from 8 to 500 µM. Milli-Q water and 1× PBS were used as positive and negative controls. The cells were then incubated at 37 °C for 3 h. Following incubation, the suspension was centrifuged at 1100× *g* for 5 min. The final optical density was measured at 540 nm. The percentage of haemolysis was calculated, using the following equation.

$$\%\;haemolysis=\frac{\left(absorbance\;of\;test\;sample-absorbance\;of\;diluent\right)}{\left(absorbance\;of\;positive\;control-absorbance\;of\;diluent\right)}\times 100\%$$

## 4. Conclusions

A library of glyoxamide-based small molecular peptidomimetics was synthesised. These peptidomimetics contained different phenyl ring systems appended to the glyoxamide group core, various *N*-alkylsulfonyl chains as the hydrophobic group, and either a tertiary ammonium hydrochloride, quaternary ammonium iodide or guanidinium hydrochloride cationic group. In the MIC assay, quaternary ammonium iodide salts **16d**, **17b**, **17c**, **18a** and guanidinium hydrochloride salt **34a** showed excellent antibacterial activity against Gram-positive *S. aureus*. Moreover, quaternary ammonium iodide salts **17b–17c** and guanidinium hydrochloride salt **34a** also showed good antibacterial activity against Gram-negative *E. coli*. The SAR studies suggested an inverse relationship between the bulkiness of the phenyl system and the length of the alkylsulfonyl chain for the high antibacterial activity of peptidomimetics. This observation was also supported by analysis of AlogP values, which suggested that there is an optimum AlogP range for a peptidomimetic to show high antibacterial activity. The more potent peptidomimetics **17c**, **34a**, **34b** and **17b** managed to disrupt 44%, 42%, 42% and 38%, respectively, of pre-established *S. aureus* biofilms at 4× MIC. Additionally, peptidomimetics **17b** and **17c** were also able to disrupt 31% and 28%, respectively, of the pre-established *E. coli* biofilms at 2× MIC. Moreover, peptidomimetic **17c** also demonstrated its ability to inhibit 70% and 65% *S. aureus* biofilm formation at 2× and 4× of its MIC, respectively. Cytoplasmic membrane permeability studies suggested that the disruption and depolarisation of the bacterial cell membrane could be a possible mechanism of action of the peptidomimetics. Furthermore, in vitro toxicity assays against human MRC-5 fibroblast cells and human red blood cells demonstrated the relatively low cytotoxicity of quaternary ammonium iodide salts **16d**, **17c** and **18a**. Overall, the quaternary ammonium iodide salt **17c** possesses good antibacterial activity against both Gram-positive and Gram-negative planktonic bacteria and their biofilms, and is identified to be a new lead in this study.

## Figures and Tables

**Table 1:** Minimum inhibitory concentration (MIC) of peptidomimetics against *S. aureus*, *E. coli* and *P. aeruginosa* and the calculated AlogP values of peptidomimetics.

	Compound	AlogP	MIC (μM/μg/mL)		
			*S. aureus*	*E. coli*	*P. aeruginosa*
Glyoxamide derivatives	**10a**	2.61	250/106 [50]	ND	ND
	**10b**	3.38	94/47 [50]	ND	ND
	**10c**	4.26	24/12 [51]	ND	ND
	**10d**	5.91	8/4.6	ND	ND
	**11a**	4.39	32/15	ND	ND
	**11b**	5.16	32/18	ND	ND
	**11c**	6.04	16/8.9	ND	ND
	**11d**	7.69	250/158	ND	ND
	**12a**	6.17	63/34	ND	ND
	**12b**	6.94	250/154	ND	ND
	**12c**	7.82	>250/>153	ND	ND
	**21**	5.91	16/9.2	ND	ND
Tertiary ammonium chloride salts	**13a**	−0.89	>250/>116 [50]	ND	ND
	**13b**	−0.12	63/34 [50]	ND	ND
	**13c**	0.76	16/8.6 [51]	ND	ND
	**13d**	2.41	16/9.8	ND	ND
	**14a**	0.89	16/8.3	ND	ND
	**14b**	1.66	16/9.6	ND	ND
	**14c**	2.54	16/9.5	ND	ND
	**14d**	4.18	125/84	ND	ND
	**15a**	2.67	63/36	ND	ND
	**15b**	3.44	250/163	ND	ND
	**15c**	4.32	>250/>163	ND	ND
	**22**	2.41	16/9.8	ND	ND
Quaternary ammonium iodide salts	**16a**	−1.55	250/142 [50]	ND	ND
	**16b**	−0.78	63/41 [50]	ND	ND
	**2**, **16c**	0.10	16/10 [51]	32/21 [51]	125/80 [51]
	**16d**	1.75	4/2.9	>250/>180	>250/>180
	**17a**	0.23	16/10	ND	ND
	**17b**	1.00	8/5.6	32/22	>250/>176
	**17c**	1.88	8/5.6	32/22	>250/>175
	**17d**	3.52	63/48	ND	ND
	**18a**	2.01	8/5.4	>250	>250
	**18b**	2.78	125/95	ND	ND
	**18c**	3.65	250/189	ND	ND
	**23**	1.75	8/5.8	ND	ND
Guanidinium hydrochloride salts	**33a**	−1.46	47/22 [50]	ND	ND
	**1**, **33b**	−0.69	12/6.7 [50]	ND	ND
	**33c**	0.19	8/4.4 [51]	63/35 [51]	63/35 [51]
	**33d**	1.84	16/10	ND	ND
	**34a**	0.32	8/4.3	16/8.5	>250/>133
	**34b**	1.09	16/9.8	>250/>153	>250/>153
	**34c**	1.97	32/19	ND	ND
	**35a**	2.10	250/147	ND	ND
	**35b**	2.87	>250/>167	ND	ND
	**35c**	3.75	>250/>166	ND	ND

ND = Not determined.

**Table 2:** IC_50_ value of peptidomimetics against MRC-5 normal human lung fibroblasts and their therapeutic indices with respect to *S. aureus* and *E. coli*.

	Compound	IC_50_ (μM/μg/mL)	Therapeutic Index	
			*S. aureus*	*E. coli*
Glyoxamide derivatives	**10d**	3.48/2.01	0.44	N/A
Tertiary ammonium chloride salts	**13d**	3.76/2.31	0.24	N/A
	**22**	3.46/2.13	0.22	N/A
Quaternary ammonium iodide salts	**16d**	53.7/38.6	15.4	N/A
	**17b**	18.3/12.9	2.29	0.57
	**17c**	57.5/40.2	7.18	1.80
	**18a**	52.4/35.6	6.54	N/A
	**23**	15.4/11.1	1.93	N/A
Guanidinium hydrochloride salts	**33d**	16.9/10.6	1.05	N/A
	**34a**	27.2/14.5	3.40	1.70
	**34b**	21.1/14.8	1.32	N/A
Positive control	**TY-01**	14.2/7.83	N/A	N/A

N/A = Not applicable.

## Data Availability

Data is contained within the article and Supplementary Materials.

## Supplementary Materials

The following are available online at https://www.mdpi.com/article/10.3390/ijms22147344/s1, ^1^H and ^13^C NMR spectra of the synthesised compounds.
