# Supplementary material for: Polyphenylglyoxamide-Based Amphiphilic Small Molecular Peptidomimetics as Antibacterial Agents with Anti-Biofilm Activity
Source: Int J Mol Sci. 2021 Jul 8;22(14):7344. doi: 10.3390/ijms22147344 (PMC8303886; doi:10.3390/ijms22147344)

Supporting information

Polyphenylglyoxamide-based Amphiphilic Small Molecular Peptidomimetics as Antibacterial Agents with Anti-biofilm Activity

Tsz Tin Yu [1], Rajesh Kuppusamy [1,2], Muhammad Yasir [2], Md. Musfizur Hassan [1], Manjulatha Sara [2], Junming Ho [1], Mark D.P. Willcox [2], David StC. Black [1,*] and Naresh Kumar [1,*]

[1] School of Chemistry, The University of New South Wales, Sydney, NSW 2052, Australia
[2] School of Optometry and Vision Science, University of New South Wales, Sydney, NSW 2052, Australia
* Correspondence: d.black@unsw.edu.au; n.kumar@unsw.edu.au

Contents

^{1}H NMR and ^{13}C NMR spectra of synthesised compounds 5
5-Phenylindoline-2,3-dione (4) 5
5-([1,1'-Biphenyl]-4-yl)indoline-2,3-dione (5) 5
5-([1,1'-Biphenyl]-4-yl)-1-(octylsulfonyl)indoline-2,3-dione (7d) 6
1-(Dodecylsulfonyl)indoline-2,3-dione (8a) 7
5-Bromo-1-(dodecylsulfonyl)indoline-2,3-dione (8b) 8
1-(Dodecylsulfonyl)-5-phenylindoline-2,3-dione (8c) 9
5-([1,1'-Biphenyl]-4-yl)-1-(dodecylsulfonyl)indoline-2,3-dione (8d) 10
1-(Hexadecylsulfonyl)indoline-2,3-dione (9a) 11
5-Bromo-1-(hexadecylsulfonyl)indoline-2,3-dione (9b) 12
1-(Hexadecylsulfonyl)-5-phenylindoline-2,3-dione (9c) 13
N-(3-(Dimethylamino)propyl)-2-(4-(octylsulfonamido)-[1,1':4',1''-terphenyl]-3-yl)-2-oxoacetamide (10d) 14
N-(3-(Dimethylamino)propyl)-2-(2-(dodecylsulfonamido)phenyl)-2-oxoacetamide (11a) 15
2-(5-Bromo-2-(dodecylsulfonamido)phenyl)-*N*-(3-(dimethylamino)propyl)-2-oxoacetamide (11b) 16
N-(3-(Dimethylamino)propyl)-2-(4-(dodecylsulfonamido)-[1,1'-biphenyl]-3-yl)-2-oxoacetamide (11c) 17
N-(3-(Dimethylamino)propyl)-2-(4-(dodecylsulfonamido)-[1,1':4',1''-terphenyl]-3-yl)-2-oxoacetamide (11d) 18
N-(3-(Dimethylamino)propyl)-2-(2-(hexadecylsulfonamido)phenyl)-2-oxoacetamide (12a) 19
2-(5-Bromo-2-(hexadecylsulfonamido)phenyl)-*N*-(3-(dimethylamino)propyl)-2-oxoacetamide (12b) 20
N-(3-(Dimethylamino)propyl)-2-(4-(hexadecylsulfonamido)-[1,1'-biphenyl]-3-yl)-2-oxoacetamide (12c) 21

N,*N*-Dimethyl-3-(2-(4-(octylsulfonamido)-[1,1':4',1''-terphenyl]-3-yl)-2-oxoacetamido)propan-1-aminium chloride (13d) 22
3-(2-(2-(Dodecylsulfonamido)phenyl)-2-oxoacetamido)-*N*,*N*-dimethylpropan-1-aminium chloride (14a) 23
3-(2-(5-Bromo-2-(dodecylsulfonamido)phenyl)-2-oxoacetamido)-*N*,*N*-dimethylpropan-1-aminium chloride (14b) 24
3-(2-(4-(Dodecylsulfonamido)-[1,1'-biphenyl]-3-yl)-2-oxoacetamido)-*N*,*N*-dimethylpropan-1-aminium chloride (14c) 25
3-(2-(4-(Dodecylsulfonamido)-[1,1':4',1''-terphenyl]-3-yl)-2-oxoacetamido)-*N*,*N*-dimethylpropan-1-aminium chloride (14d) 26
3-(2-(2-(Hexadecylsulfonamido)phenyl)-2-oxoacetamido)-*N*,*N*-dimethylpropan-1-aminium chloride (15a) 27
3-(2-(5-Bromo-2-(hexadecylsulfonamido)phenyl)-2-oxoacetamido)-*N*,*N*-dimethylpropan-1-aminium chloride (15b) 28
3-(2-(4-(Hexadecylsulfonamido)-[1,1'-biphenyl]-3-yl)-2-oxoacetamido)-*N*,*N*-dimethylpropan-1-aminium chloride (15c) 29
N,*N*,*N*-Trimethyl-3-(2-(4-(octylsulfonamido)-[1,1':4',1''-terphenyl]-3-yl)-2-oxoacetamido)propan-1-aminium iodide (16d) 30
3-(2-(2-(Dodecylsulfonamido)phenyl)-2-oxoacetamido)-*N*,*N*,*N*-trimethylpropan-1-aminium iodide (17a) 31
3-(2-(5-Bromo-2-(dodecylsulfonamido)phenyl)-2-oxoacetamido)-*N*,*N*,*N*-trimethylpropan-1-aminium iodide (17b) 32
3-(2-(4-(Dodecylsulfonamido)-[1,1'-biphenyl]-3-yl)-2-oxoacetamido)-*N*,*N*,*N*-trimethylpropan-1-aminium iodide (17c) 33
3-(2-(4-(Dodecylsulfonamido)-[1,1':4',1''-terphenyl]-3-yl)-2-oxoacetamido)-*N*,*N*,*N*-trimethylpropan-1-aminium iodide (17d) 34
3-(2-(2-(Hexadecylsulfonamido)phenyl)-2-oxoacetamido)-*N*,*N*,*N*-trimethylpropan-1-aminium iodide (18a) 35
3-(2-(5-Bromo-2-(hexadecylsulfonamido)phenyl)-2-oxoacetamido)-*N*,*N*,*N*-trimethylpropan-1-aminium iodide (18b) 36
3-(2-(4-(Hexadecylsulfonamido)-[1,1'-biphenyl]-3-yl)-2-oxoacetamido)-*N*,*N*,*N*-trimethylpropan-1-aminium iodide (18c) 37
5-([1,1'-Biphenyl]-3-yl)indoline-2,3-dione (19) 38
5-([1,1'-Biphenyl]-3-yl)-1-(octylsulfonyl)indoline-2,3-dione (20) 39
N-(3-(Dimethylamino)propyl)-2-(4-(octylsulfonamido)-[1,1':3',1''-terphenyl]-3-yl)-2-oxoacetamide (21) 40
N,*N*-Dimethyl-3-(2-(4-(octylsulfonamido)-[1,1':3',1''-terphenyl]-3-yl)-2-oxoacetamido)propan-1-aminium chloride (22) 41
N,*N*,*N*-Trimethyl-3-(2-(4-(octylsulfonamido)-[1,1':3',1''-terphenyl]-3-yl)-2-oxoacetamido)propan-1-aminium iodide (23) 42

tert-Butyl (3-(2-(4-(octylsulfonamido)-[1,1':4',1''-terphenyl]-3-yl)-2-oxoacetamido)propyl)carbamate (24d) ... 43
tert-Butyl (3-(2-(2-(dodecylsulfonamido)phenyl)-2-oxoacetamido)propyl)carbamate (25a) ... 44
tert-Butyl (3-(2-(5-bromo-2-(dodecylsulfonamido)phenyl)-2-oxoacetamido)propyl)carbamate (25b) ... 45
tert-Butyl (3-(2-(4-(dodecylsulfonamido)-[1,1'-biphenyl]-3-yl)-2-oxoacetamido)propyl)carbamate (25c) ... 46
tert-Butyl (3-(2-(2-(hexadecylsulfonamido)phenyl)-2-oxoacetamido)propyl)carbamate (26a) ... 47
tert-Butyl (3-(2-(5-bromo-2-(hexadecylsulfonamido)phenyl)-2-oxoacetamido)propyl)carbamate (26b) ... 48
tert-Butyl (3-(2-(4-(hexadecylsulfonamido)-[1,1'-biphenyl]-3-yl)-2-oxoacetamido)propyl)carbamate (26c) ... 49
N-(3-aminopropyl)-2-(4-(octylsulfonamido)-[1,1':4',1''-terphenyl]-3-yl)-2-oxoacetamide hydrochloride (27d) ... 50
N-(3-Aminopropyl)-2-(2-(dodecylsulfonamido)phenyl)-2-oxoacetamide hydrochloride (28a) ... 51
N-(3-Aminopropyl)-2-(5-bromo-2-(dodecylsulfonamido)phenyl)-2-oxoacetamide hydrochloride (28b) ... 52
N-(3-aminopropyl)-2-(4-(dodecylsulfonamido)-[1,1'-biphenyl]-3-yl)-2-oxoacetamide hydrochloride (28c) ... 53
N-(3-Aminopropyl)-2-(2-(hexadecylsulfonamido)phenyl)-2-oxoacetamide hydrochloride (29a) 54
N-(3-Aminopropyl)-2-(5-bromo-2-(hexadecylsulfonamido)phenyl)-2-oxoacetamide hydrochloride (29b) ... 55
N-(3-Aminopropyl)-2-(4-(hexadecylsulfonamido)-[1,1'-biphenyl]-3-yl)-2-oxoacetamide hydrochloride (29c) ... 56
(*E*)-1-*tert*-Butyl-*N*-(*N*'-((*tert*-butyloxidanyl)carbonyl)-*N*-(3-(2-(4-(octylsulfonamido)-[1,1':4',1''-terphenyl]-3-yl)-2-oxoacetamido)propyl)carbamimidoyl)-1-oxidanecarboxamide (30d) ... 57
(*E*)-1-*tert*-Butyl-*N*-(*N*'-((tert-butyloxidanyl)carbonyl)-*N*-(3-(2-(2-(dodecylsulfonamido)phenyl)-2-oxoacetamido)propyl)carbamimidoyl)-1-oxidanecarboxamide (31a) ... 58
(*E*)-1-*tert*-Butyl-*N*-(*N*'-((*tert*-butyloxidanyl)carbonyl)-*N*-(3-(2-(5-bromo-2-(dodecylsulfonamido) phenyl)-2-oxoacetamido)propyl)carbamimidoyl)-1-oxidanecarboxamide (31b) ... 59
(*E*)-1-*tert*-Butyl-*N*-(*N*'-((*tert*-butyloxidanyl)carbonyl)-*N*-(3-(2-(4-(dodecylsulfonamido)-[1,1'-biphenyl]-3-yl)-2-oxoacetamido)propyl)carbamimidoyl)-1-oxidanecarboxamide (31c) ... 60
(*E*)-1-*tert*-Butyl-*N*-(*N*'-((*tert*-butyloxidanyl)carbonyl)-*N*-(3-(2-(2-(hexadecylsulfonamido)phenyl)-2-oxoacetamido)propyl)carbamimidoyl)-1-oxidanecarboxamide (32a) ... 61
(*E*)-1-*tert*-Butyl-*N*-(*N*'-((*tert*-butyloxidanyl)carbonyl)-*N*-(3-(2-(5-bromo-2-(hexadecylsulfonamido) phenyl)-2-oxoacetamido)propyl)carbamimidoyl)-1-oxidanecarboxamide (32b) ... 62
(*E*)-1-*tert*-Butyl-*N*-(*N*'-((*tert*-butyloxidanyl)carbonyl)-*N*-(3-(2-(4-(hexadecylsulfonamido)-[1,1'-biphenyl]-3-yl)-2-oxoacetamido)propyl)carbamimidoyl)-1-oxidanecarboxamide (32c) ... 63

N-(3-Guanidinopropyl)-2-(4-(octylsulfonamido)-[1,1':4',1''-terphenyl]-3-yl)-2-oxoacetamide hydrochloride (33d) 64
2-(2-(Dodecylsulfonamido)phenyl)-*N*-(3-guanidinopropyl)-2-oxoacetamide hydrochloride (34a) 65
2-(5-Bromo-2-(dodecylsulfonamido)phenyl)-*N*-(3-guanidinopropyl)-2-oxoacetamide hydrochloride (34b) 66
2-(4-(Dodecylsulfonamido)-[1,1'-biphenyl]-3-yl)-*N*-(3-guanidinopropyl)-2-oxoacetamide hydrochloride (34c) 67
N-(3-Guanidinopropyl)-2-(2-(hexadecylsulfonamido)phenyl)-2-oxoacetamide hydrochloride (35a) 68
2-(5-Bromo-2-(hexadecylsulfonamido)phenyl)-*N*-(3-guanidinopropyl)-2-oxoacetamide hydrochloride (35b) 69
N-(3-Guanidinopropyl)-2-(4-(hexadecylsulfonamido)-[1,1'-biphenyl]-3-yl)-2-oxoacetamide hydrochloride (35c) 70

^{1}H NMR and ^{13}C NMR spectra of synthesised compounds

5-Phenylindoline-2,3-dione (**4**)

^{1}H NMR (400 MHz, DMSO-d_6):

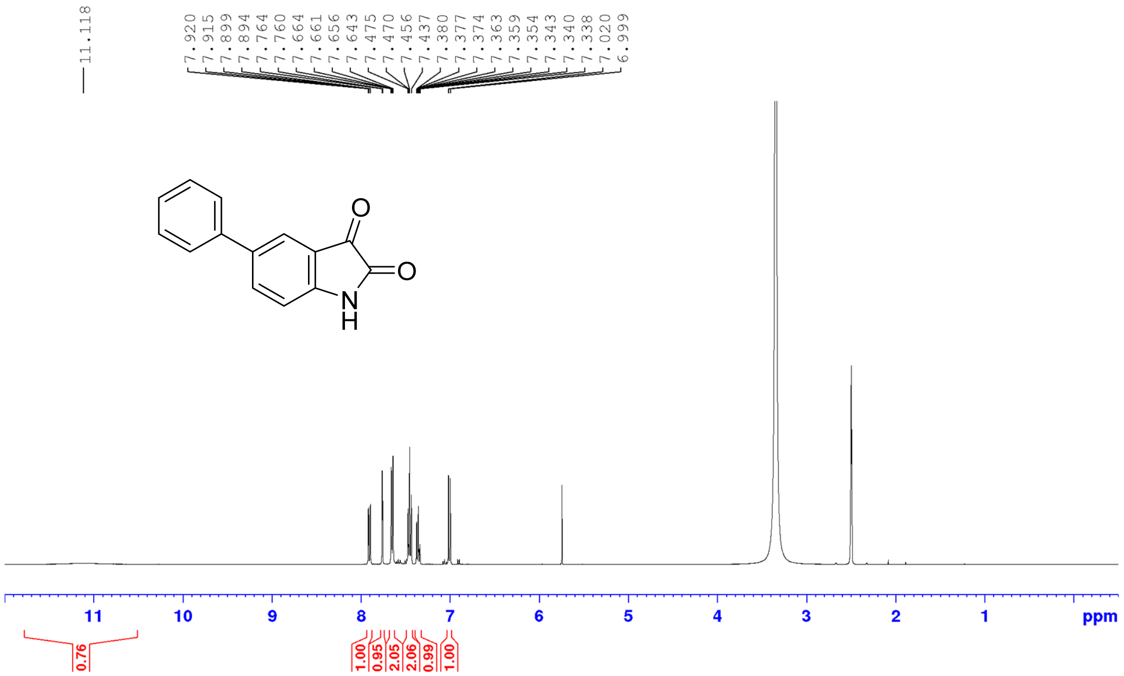

5-([1,1'-Biphenyl]-4-yl)indoline-2,3-dione **(5)**

^{1}H NMR (400 MHz, DMSO-d_6):

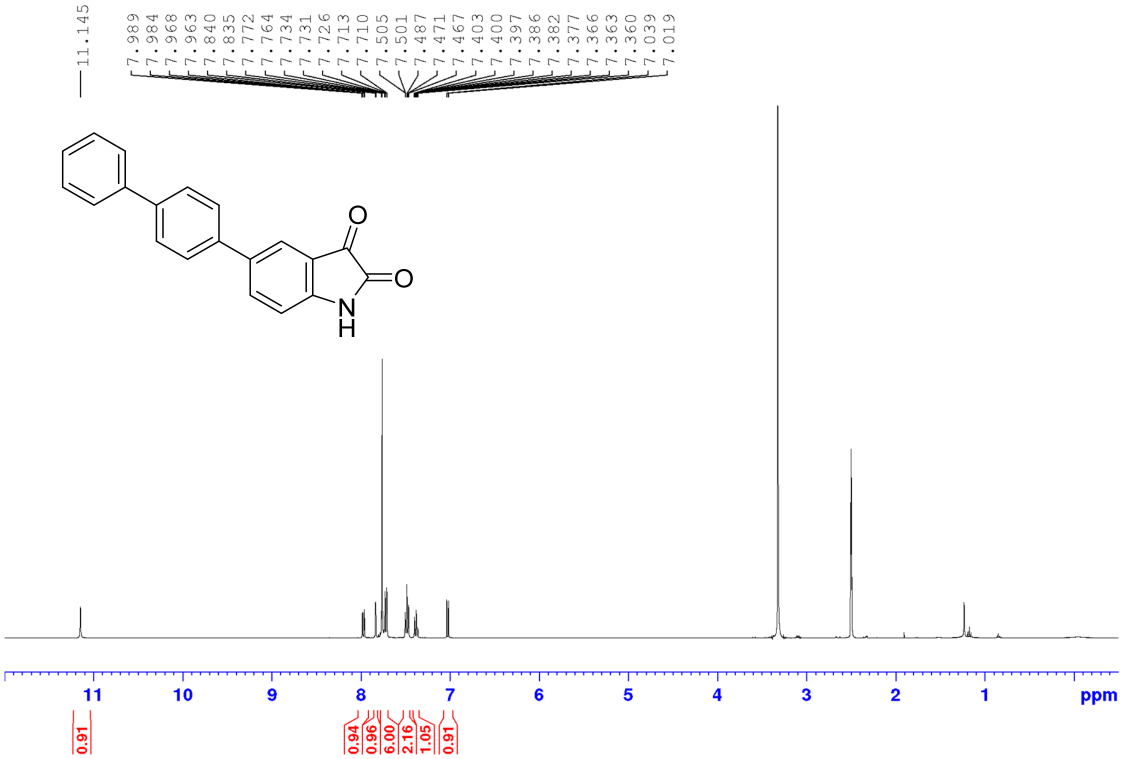

5-([1,1'-Biphenyl]-4-yl)-1-(octylsulfonyl)indoline-2,3-dione (**7d**)

^{1}H NMR (400 MHz, DMSO-d_6):

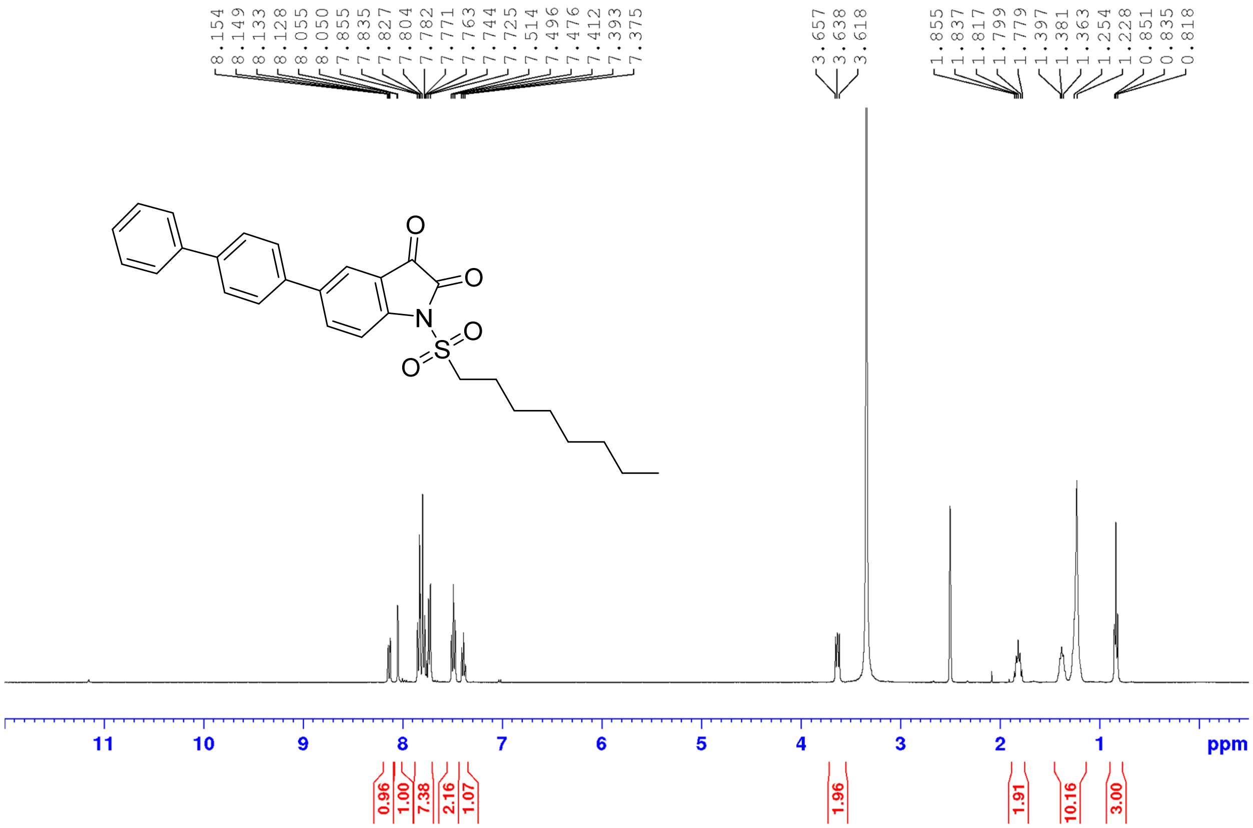

^{13}C NMR (100 MHz, DMSO-d_6):

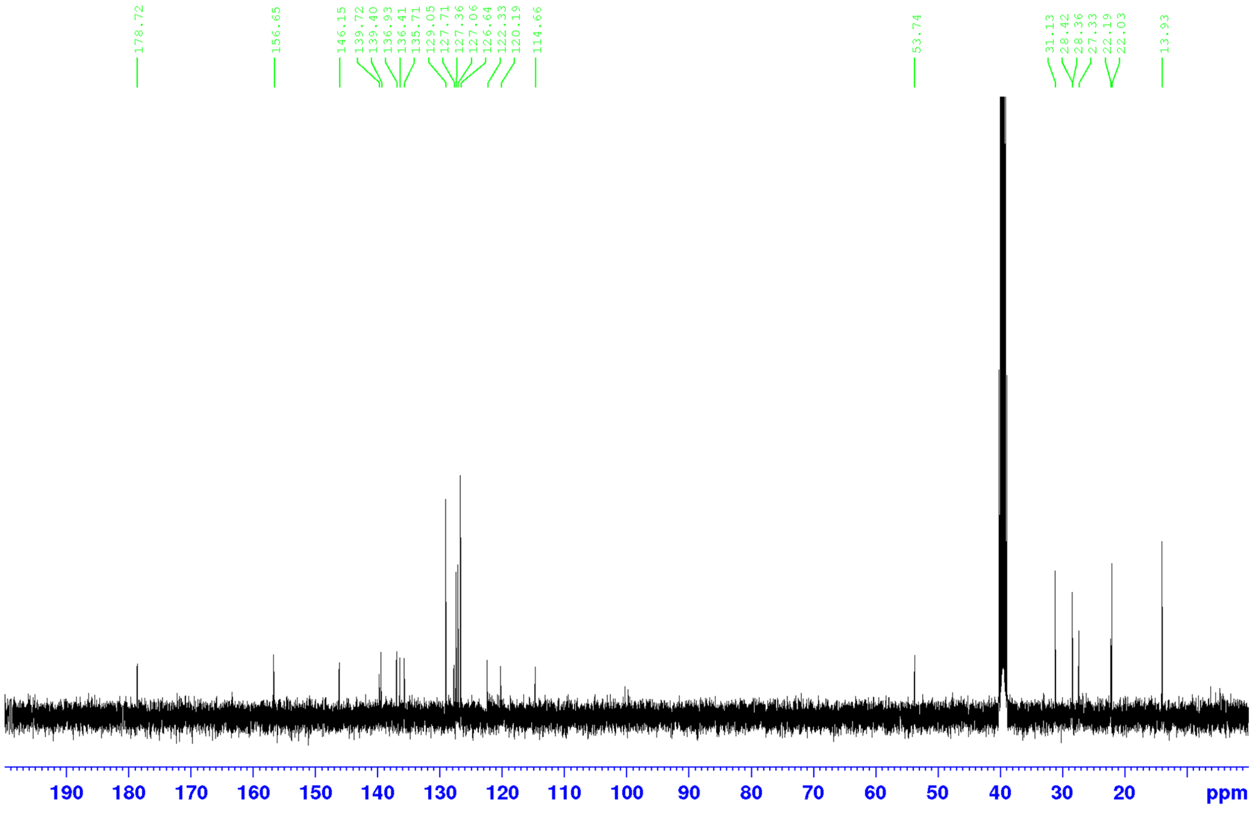

1-(Dodecylsulfonyl)indoline-2,3-dione (**8a**)

^{1}H NMR (400 MHz, DMSO-d_6):

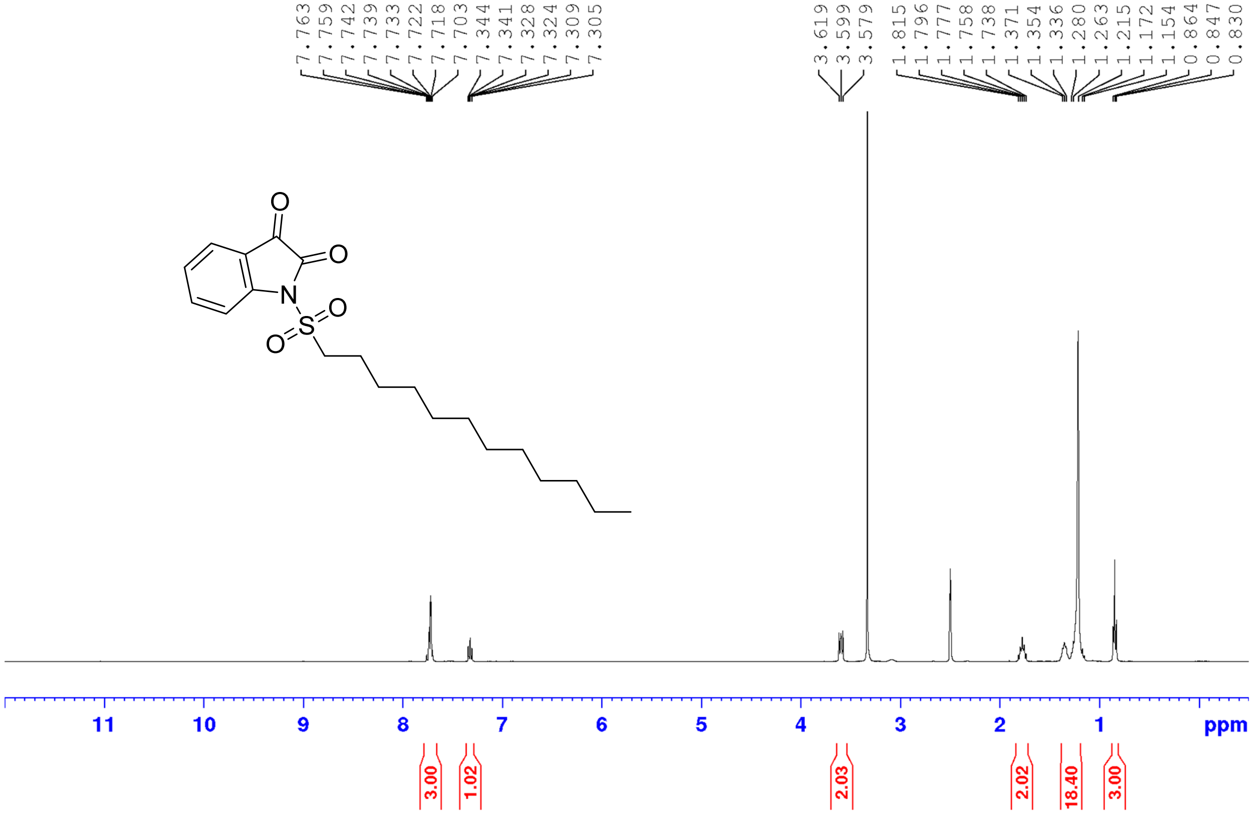

^{13}C NMR (100 MHz, DMSO-d_6):

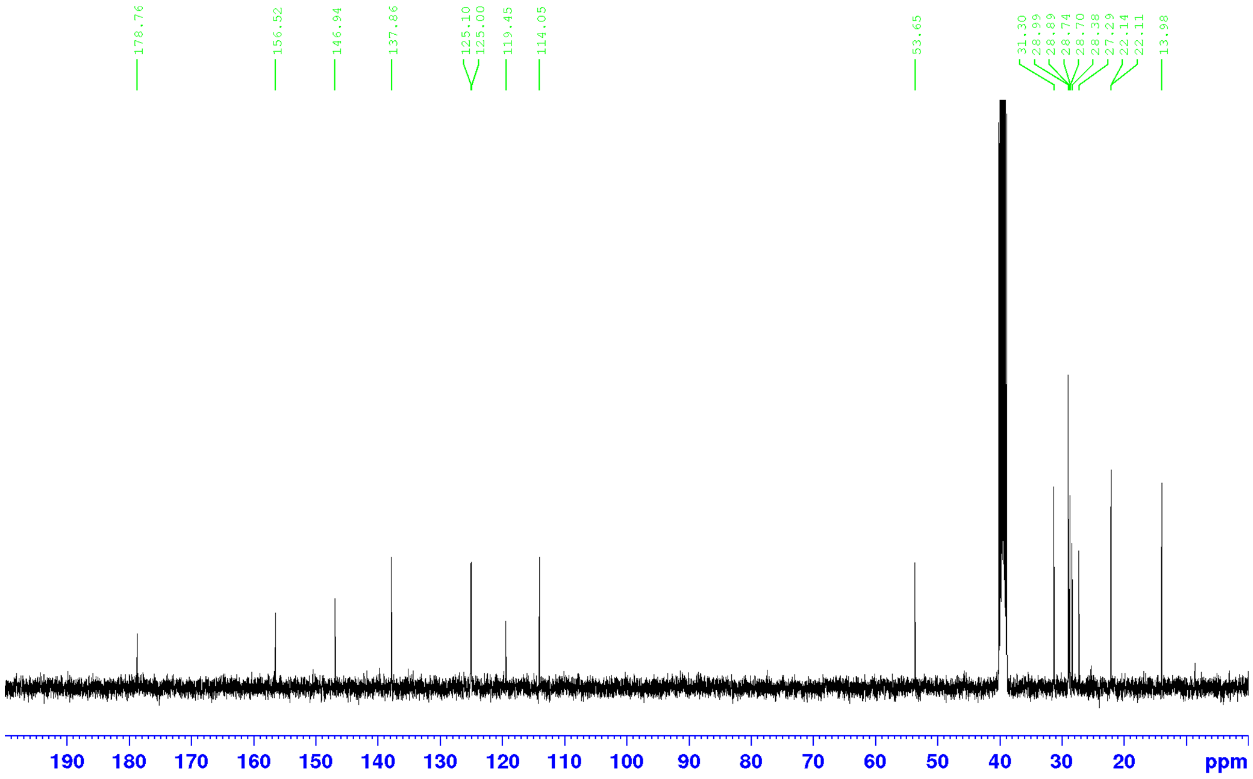

5-Bromo-1-(dodecylsulfonyl)indoline-2,3-dione (**8b**)

^{1}H NMR (400 MHz, DMSO-d_6):

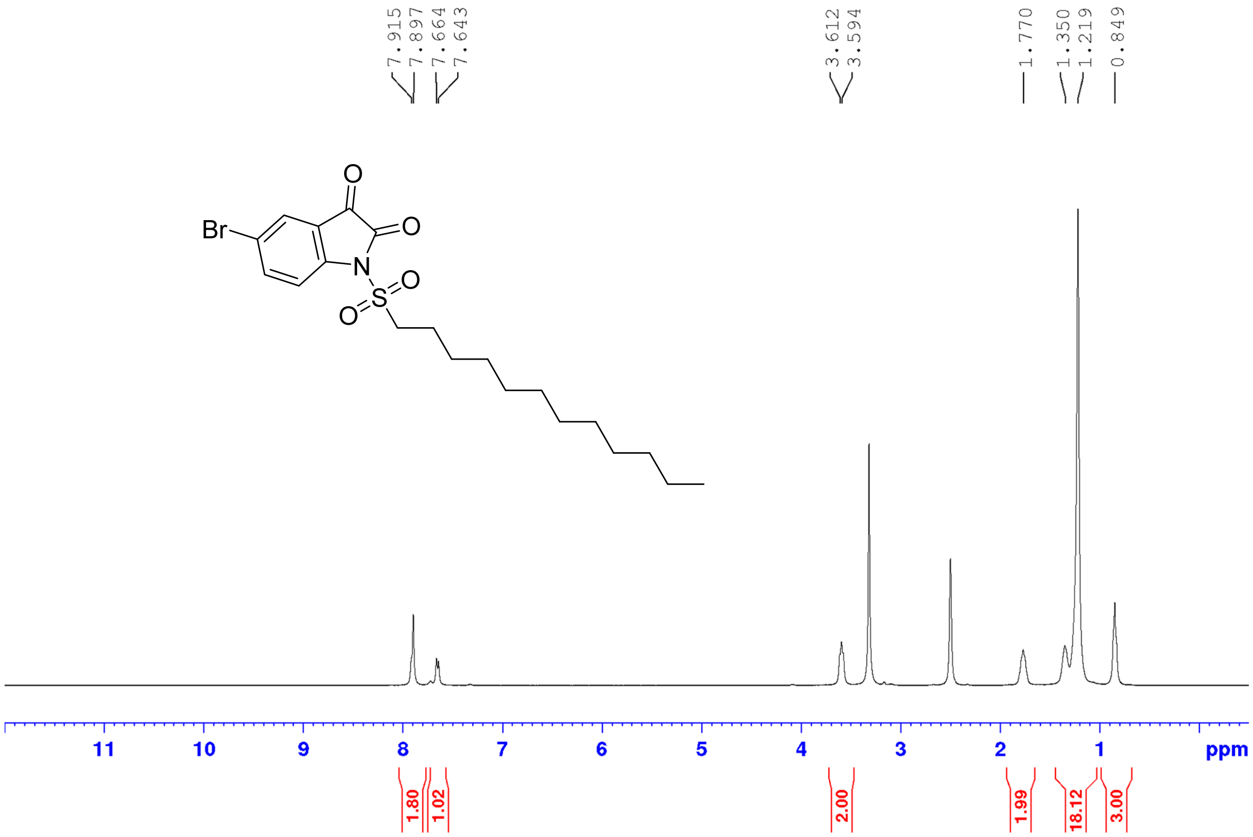

^{13}C NMR (100 MHz, DMSO-d_6):

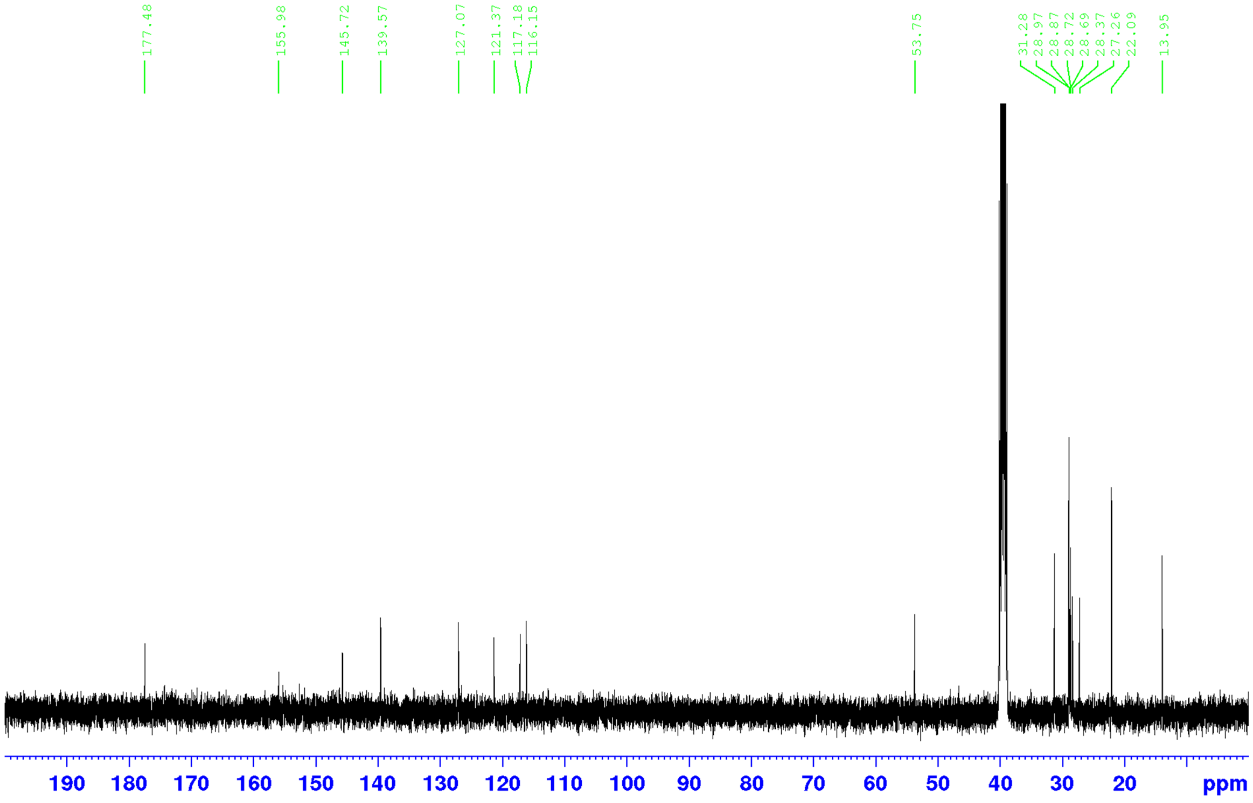

1-(Dodecylsulfonyl)-5-phenylindoline-2,3-dione (**8c**)

^{1}H NMR (400 MHz, DMSO-d_6):

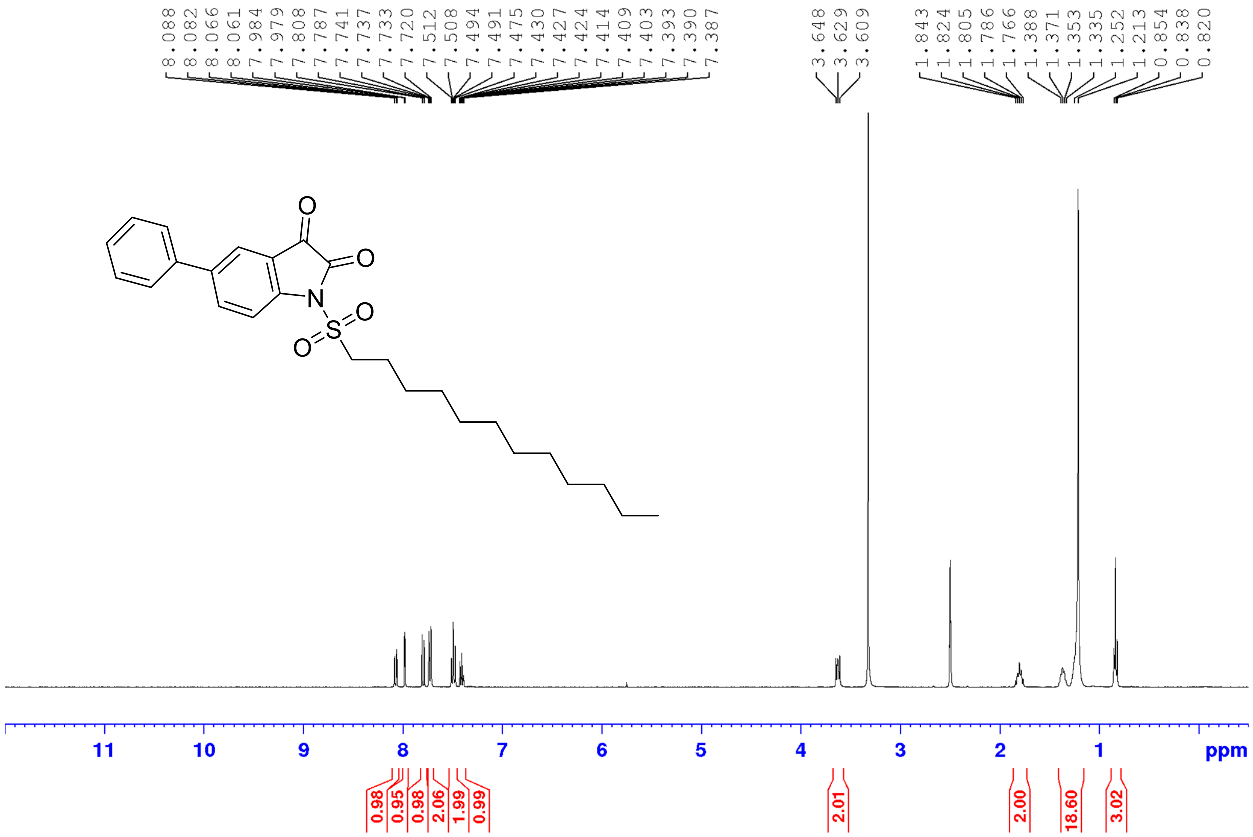

^{13}C NMR (100 MHz, DMSO-d_6):

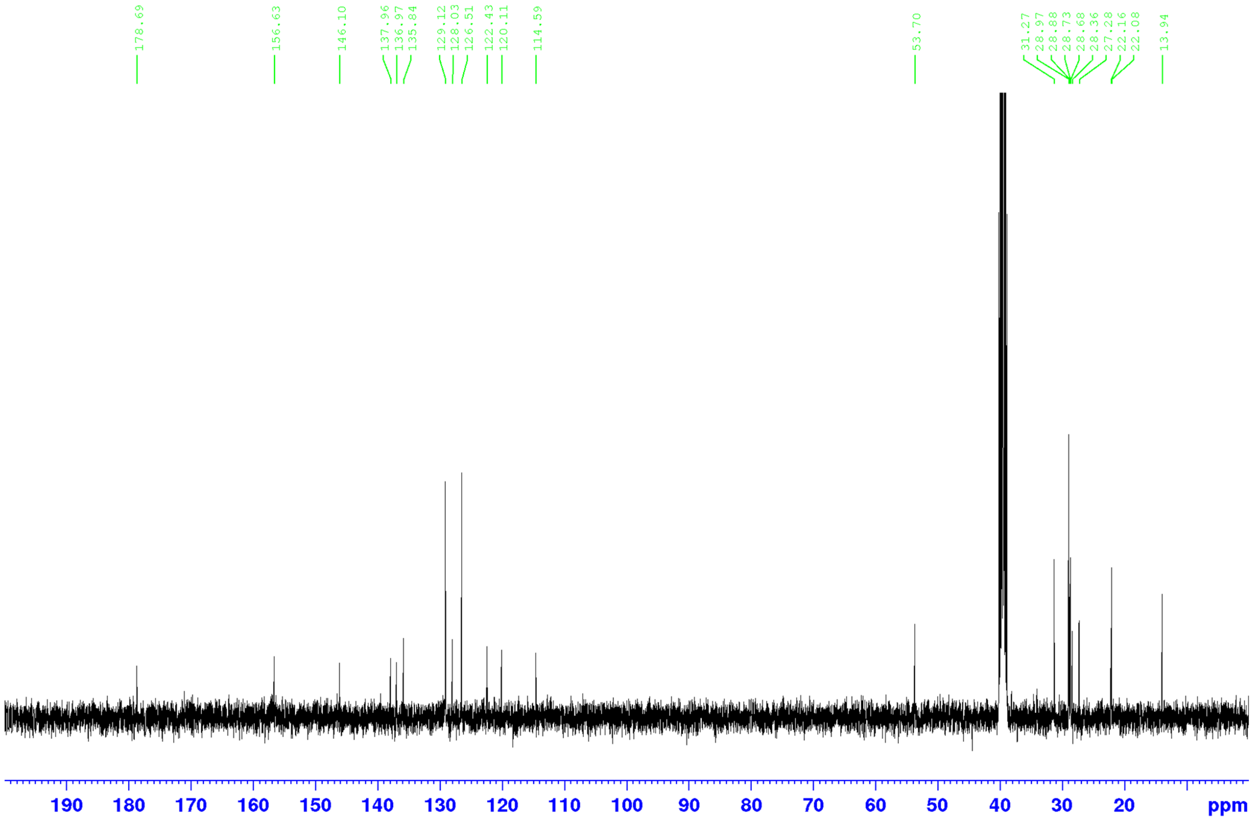

5-([1,1'-Biphenyl]-4-yl)-1-(dodecylsulfonyl)indoline-2,3-dione (**8d**)

^{1}H NMR (600 MHz, DMSO-d_6):

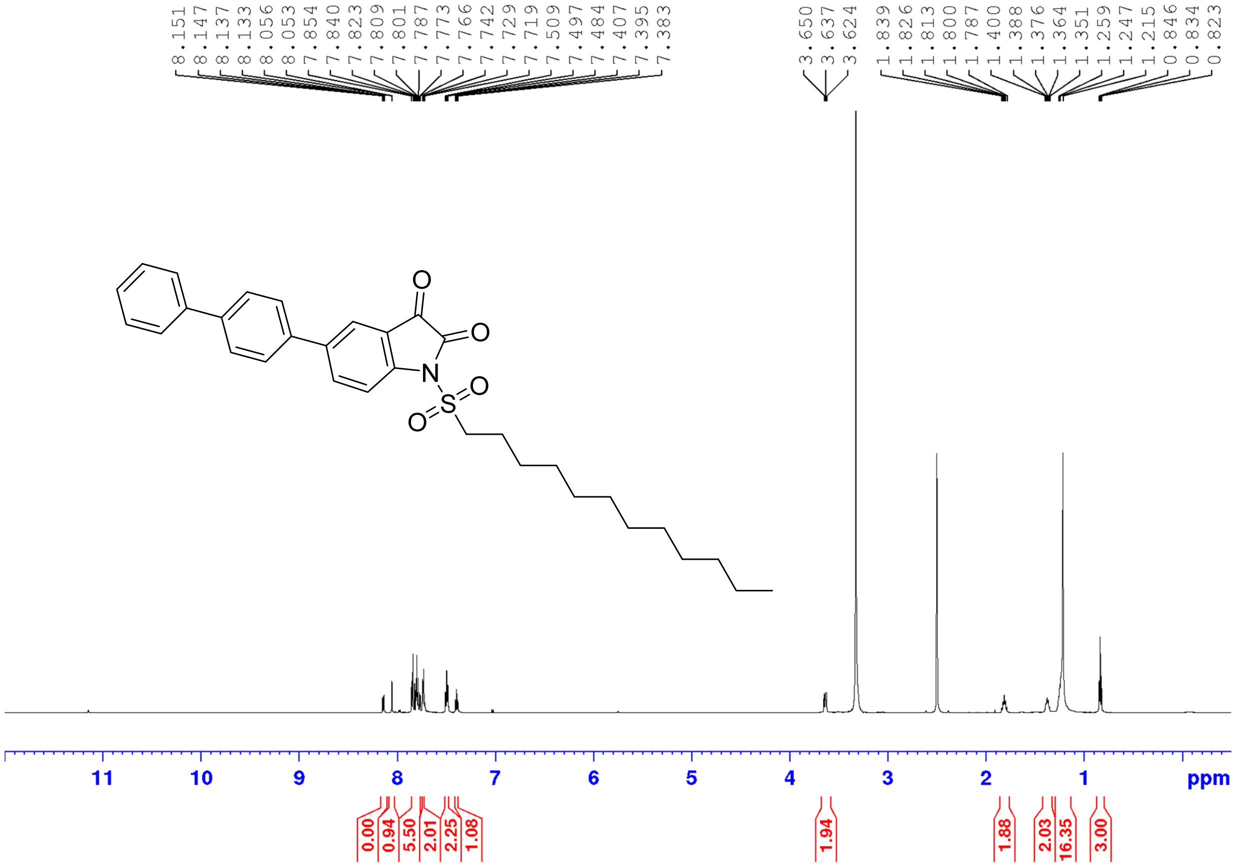

^{13}C NMR (150 MHz, DMSO-d_6):

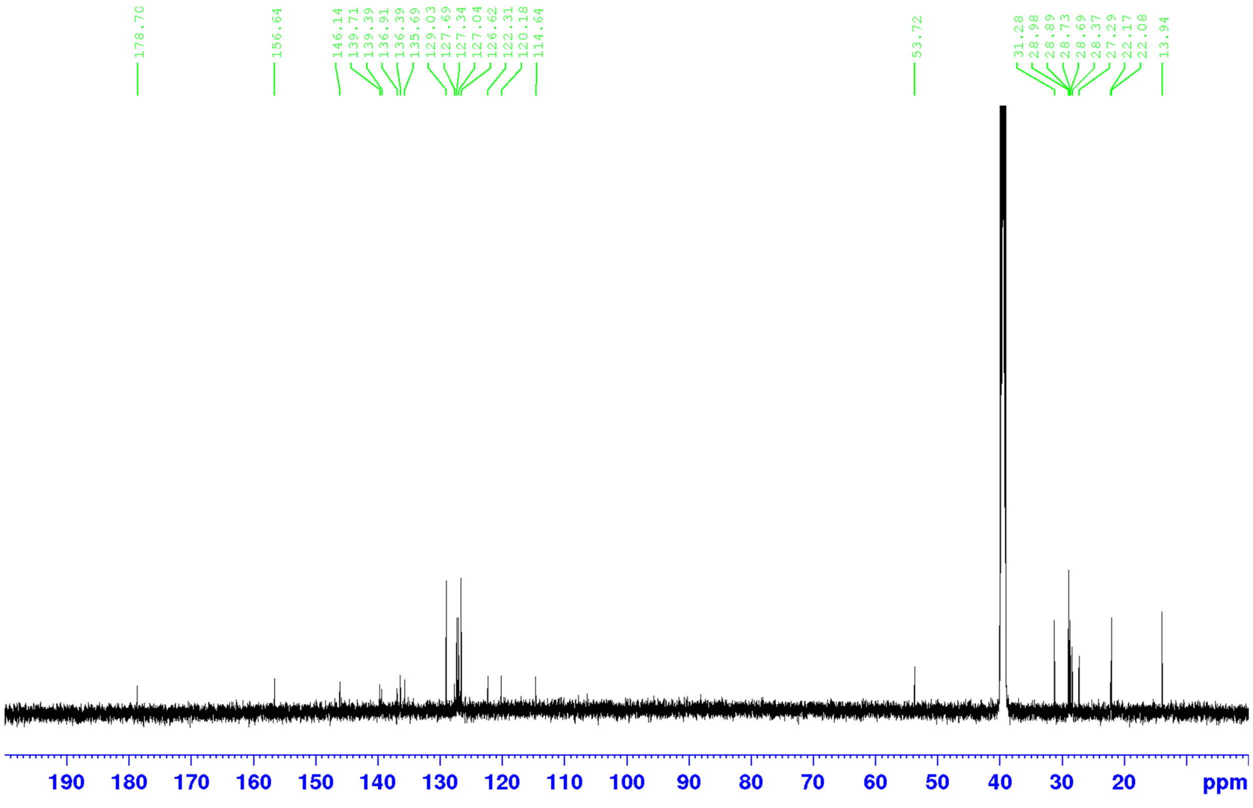

1-(Hexadecylsulfonyl)indoline-2,3-dione (**9a**)

^{1}H NMR (400 MHz, $CDCl_3$):

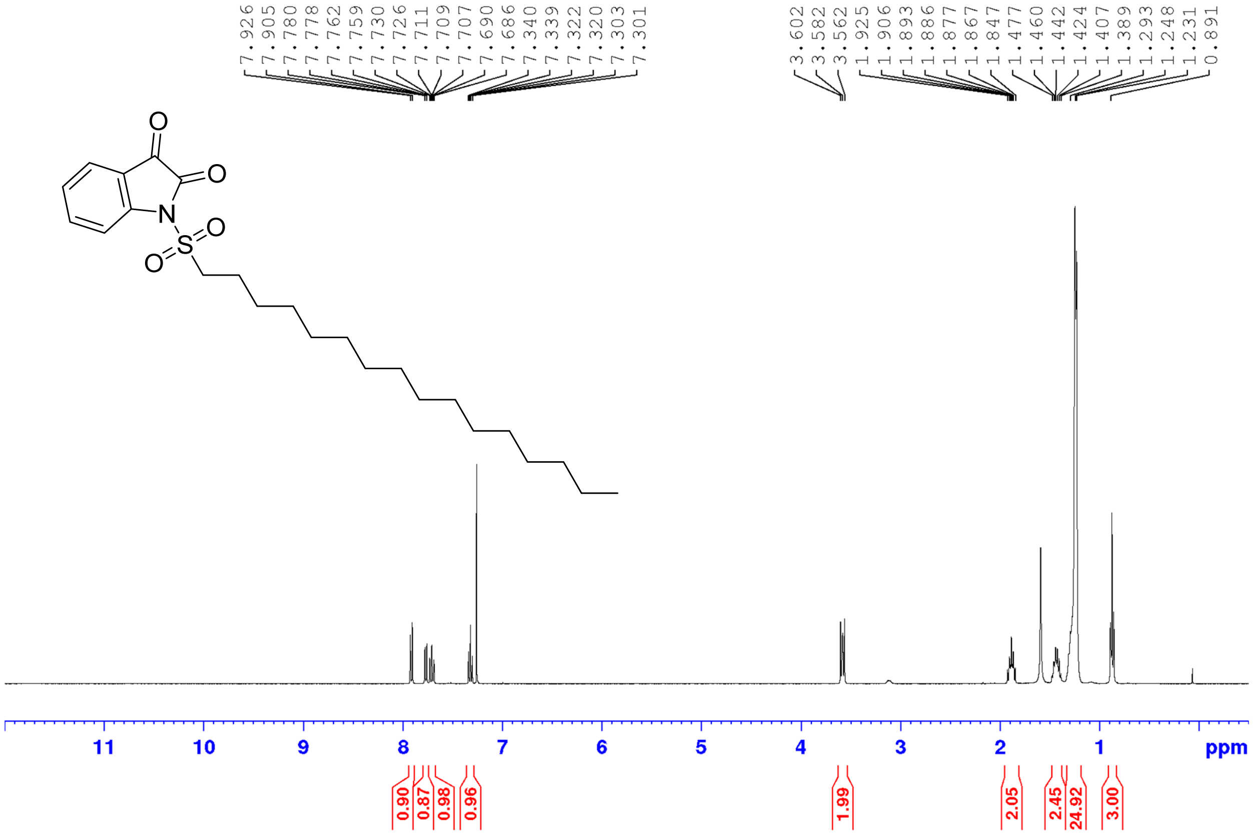

^{13}C NMR (100 MHz, $CDCl_3$):

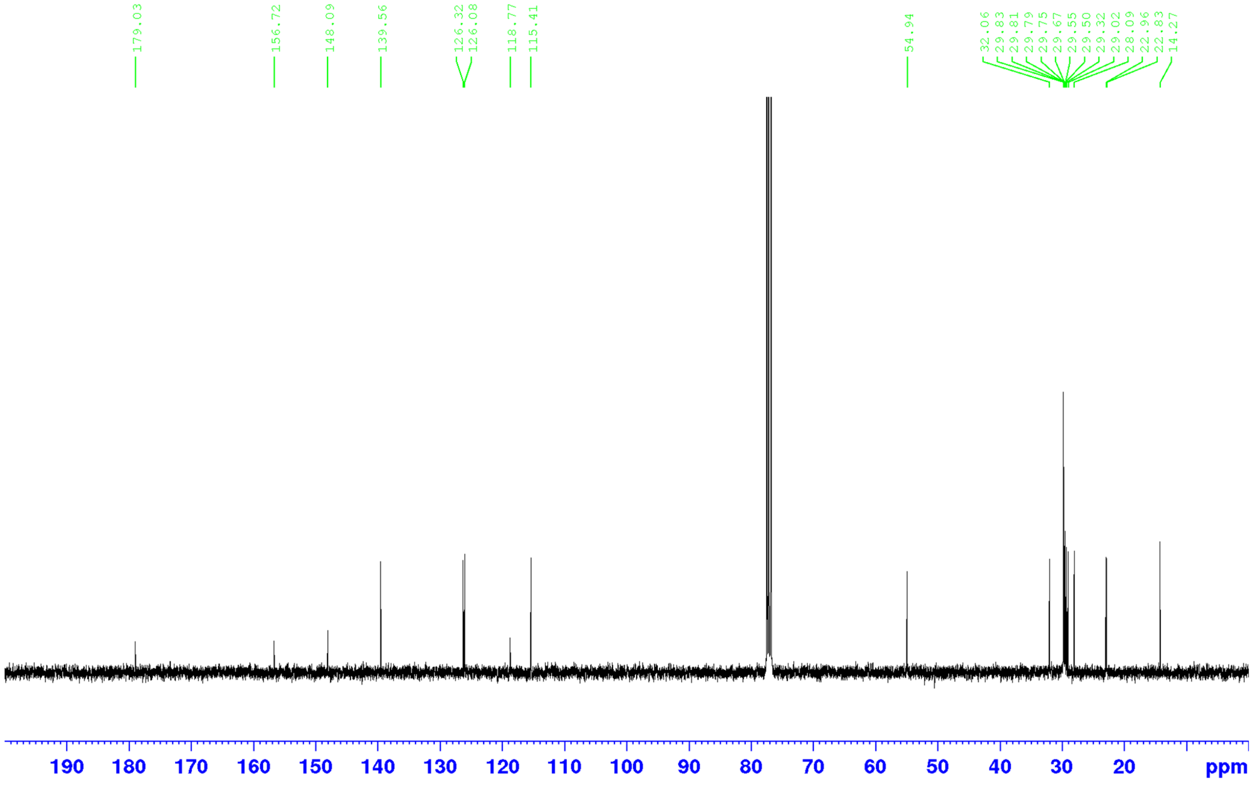

5-Bromo-1-(hexadecylsulfonyl)indoline-2,3-dione (**9b**)

^{1}H NMR (600 MHz, DMSO-d_6):

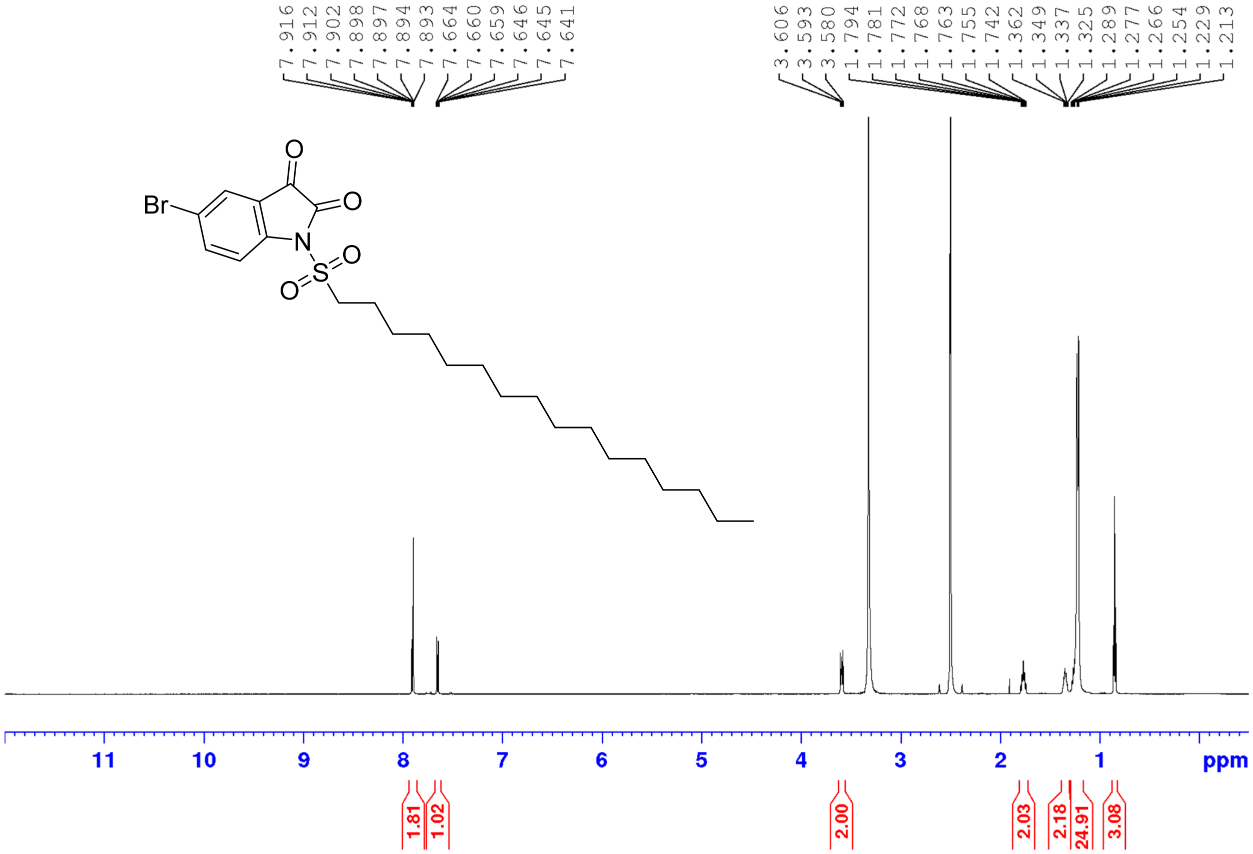

^{13}C NMR (150 MHz, DMSO-d_6):

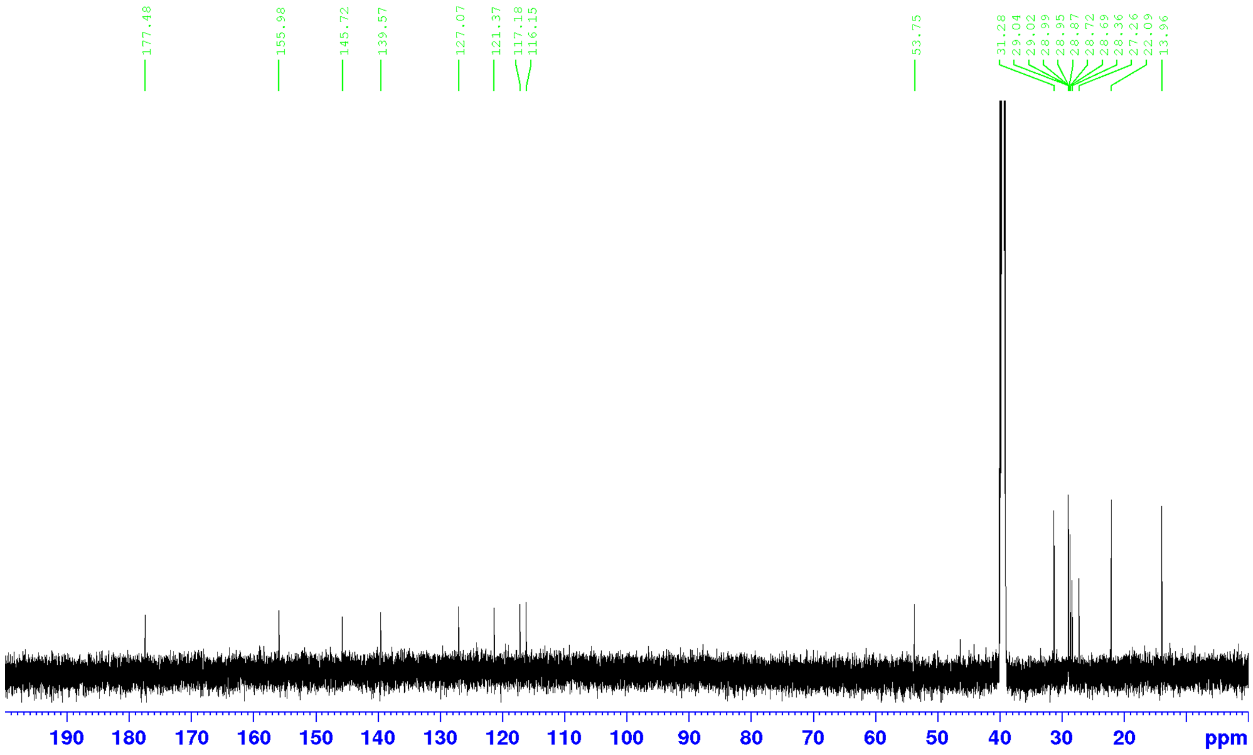

1-(Hexadecylsulfonyl)-5-phenylindoline-2,3-dione (**9c**)

^{1}H NMR (600 MHz, $CDCl_3$):

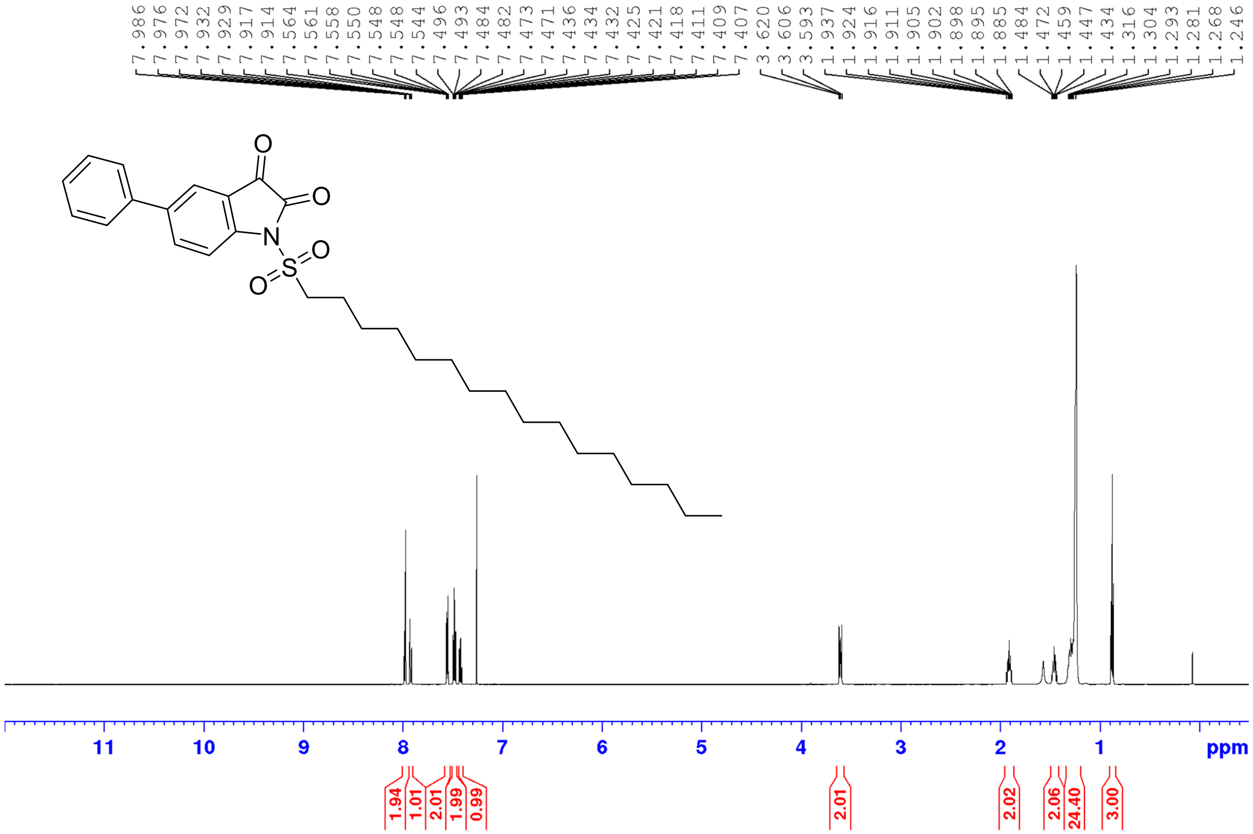

^{13}C NMR (150 MHz, $CDCl_3$):

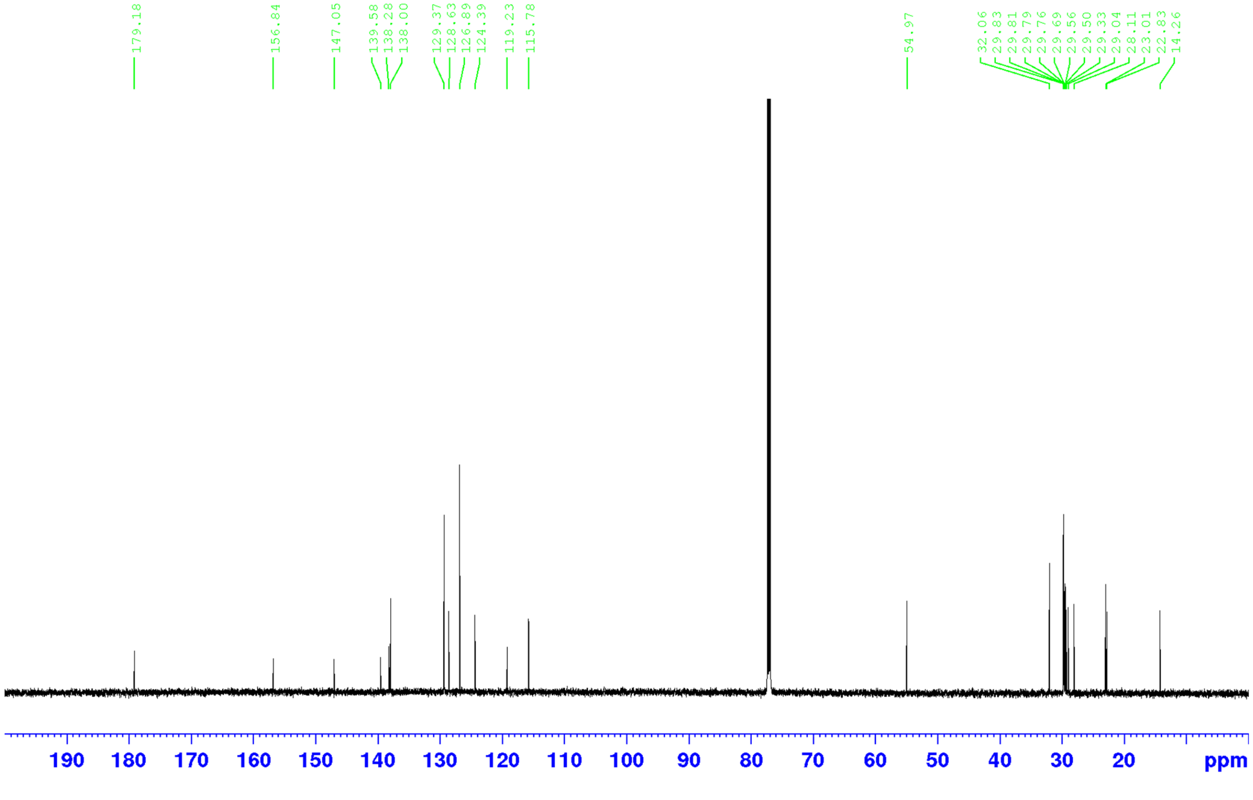

N-(3-(Dimethylamino)propyl)-2-(4-(octylsulfonamido)-[1,1':4',1"-terphenyl]-3-yl)-2-oxoacetamide (**10d**)

^{1}H NMR (600 MHz, $CDCl_3$):

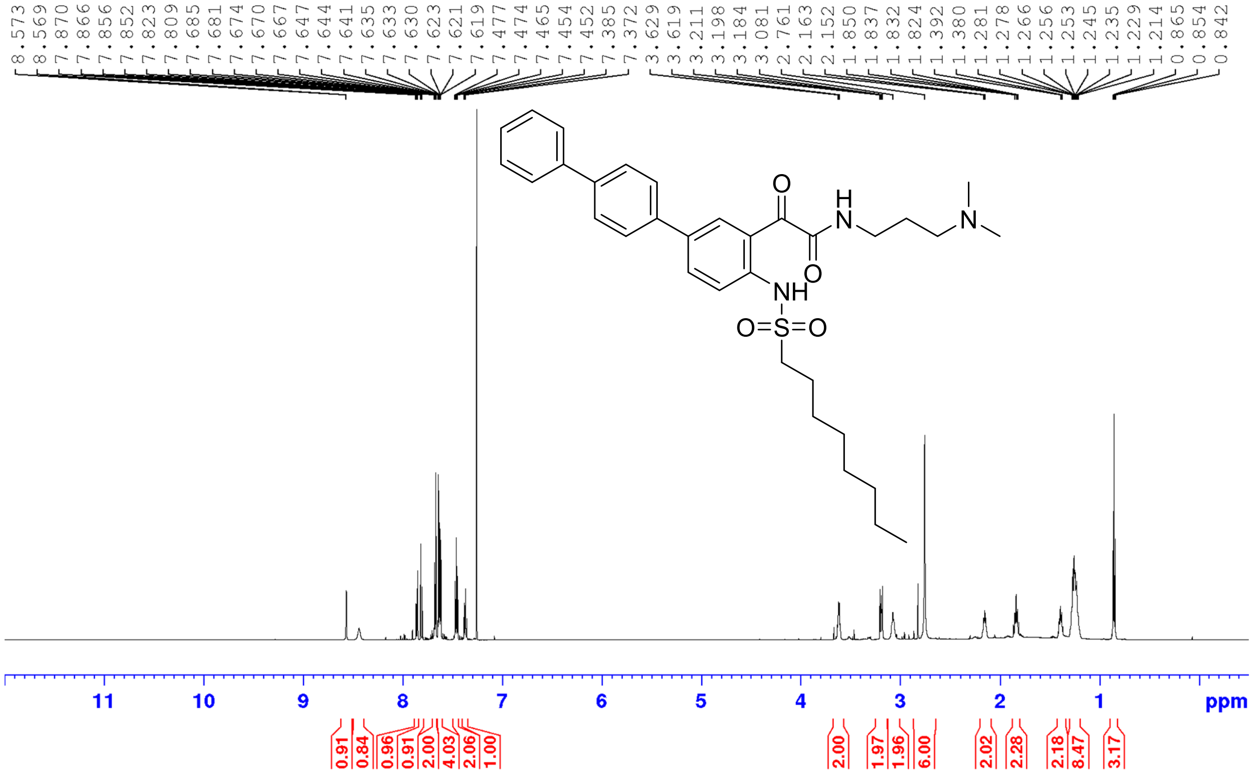

^{13}C NMR (150 MHz, $CDCl_3$):

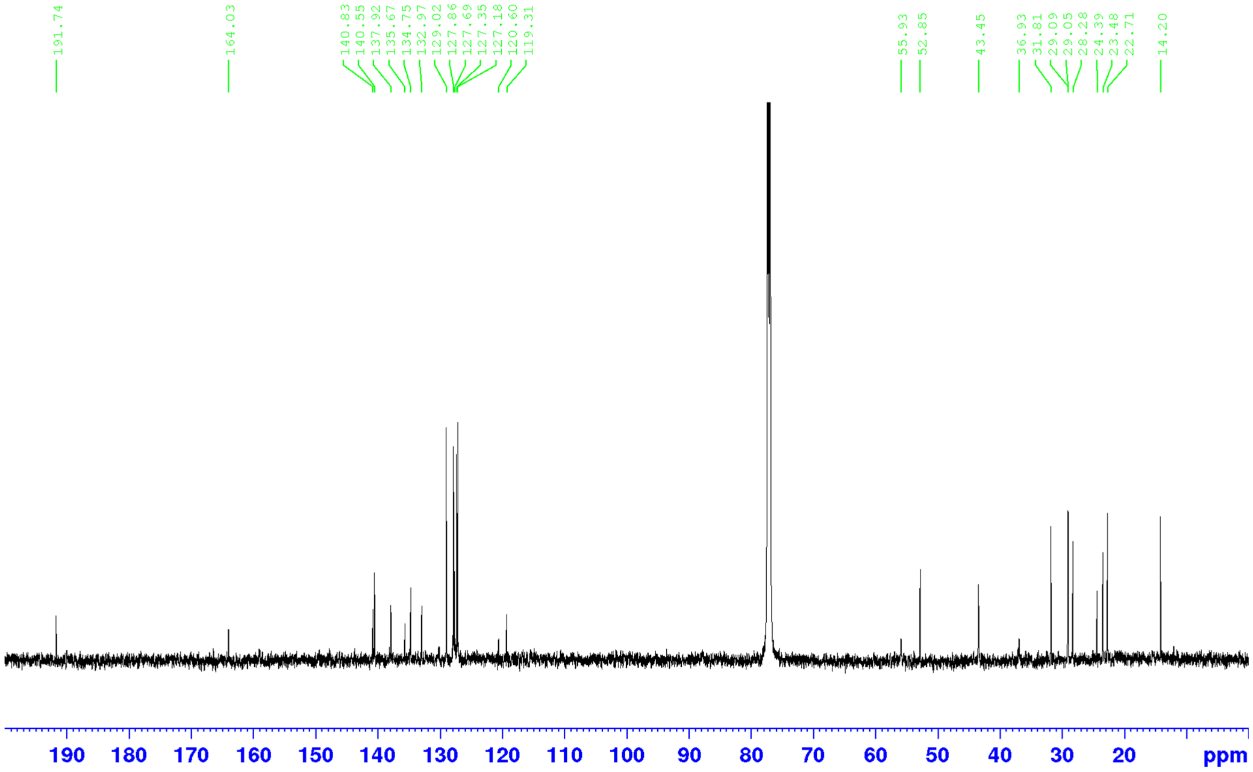

N-(3-(Dimethylamino)propyl)-2-(2-(dodecylsulfonamido)phenyl)-2-oxoacetamide (**11a**)

^{1}H NMR (400 MHz, $CDCl_3$):

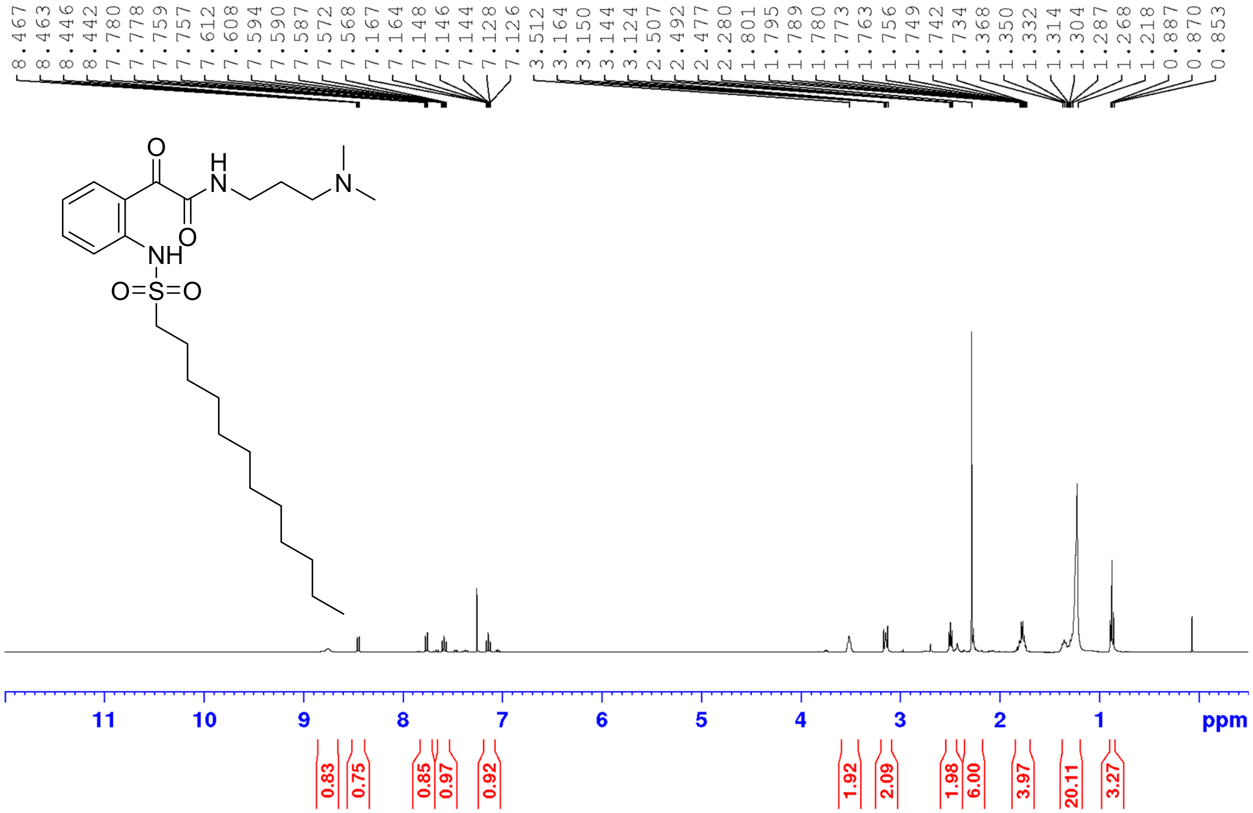

^{13}C NMR (100 MHz, $CDCl_3$):

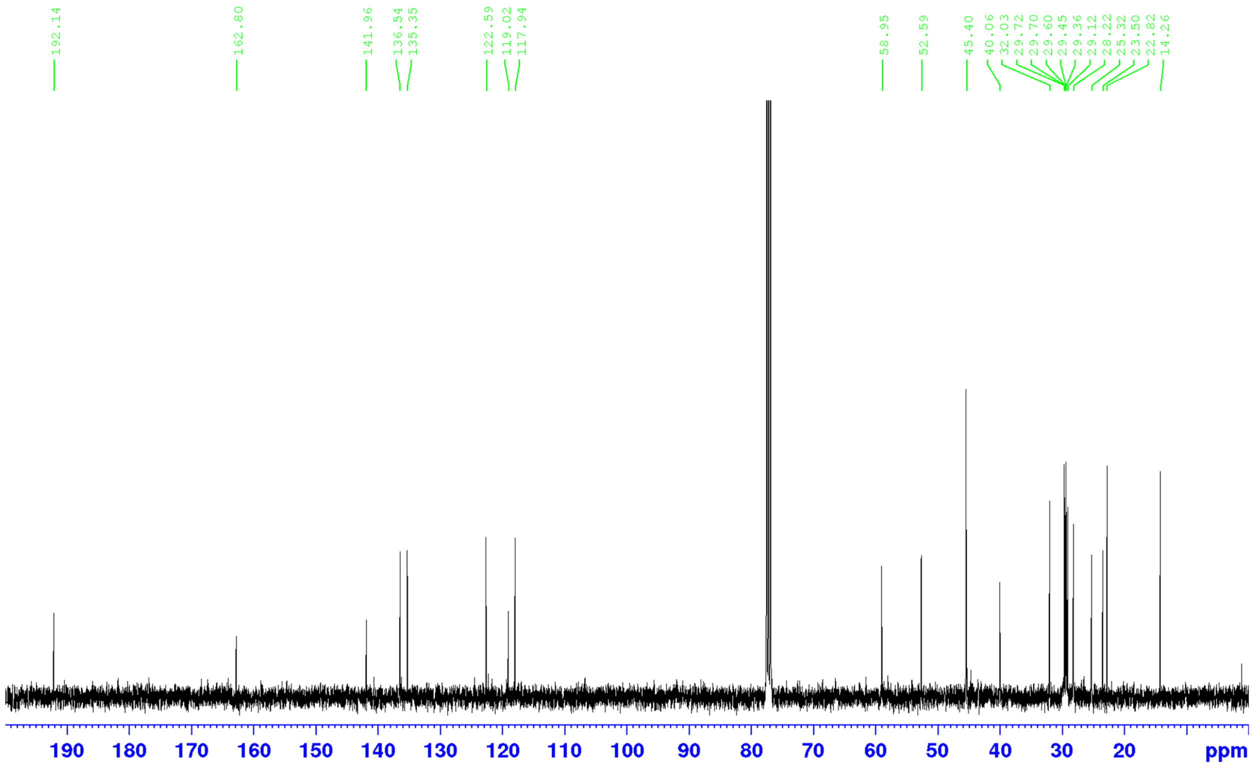

2-(5-Bromo-2-(dodecylsulfonamido)phenyl)-*N*-(3-(dimethylamino)propyl)-2-oxoacetamide (**11b**)

^{1}H NMR (400 MHz, $CDCl_3$):

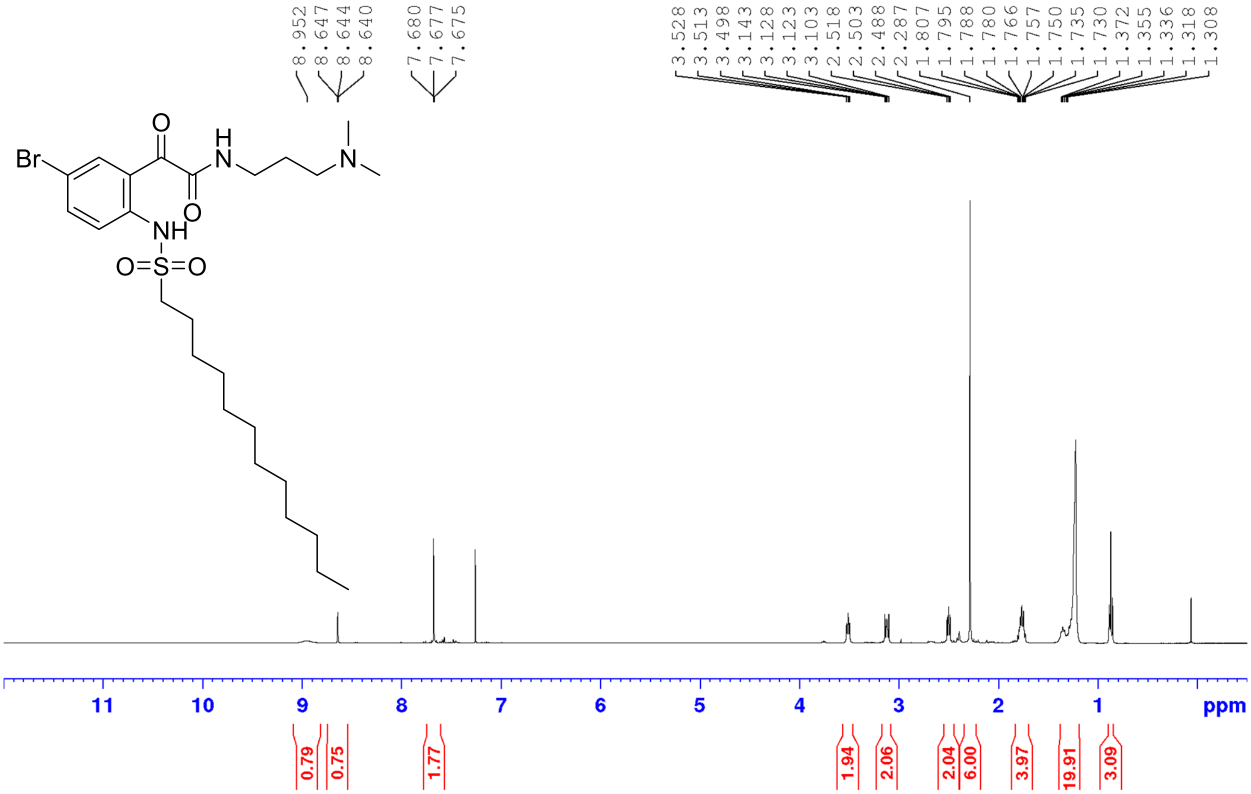

^{13}C NMR (100 MHz, $CDCl_3$):

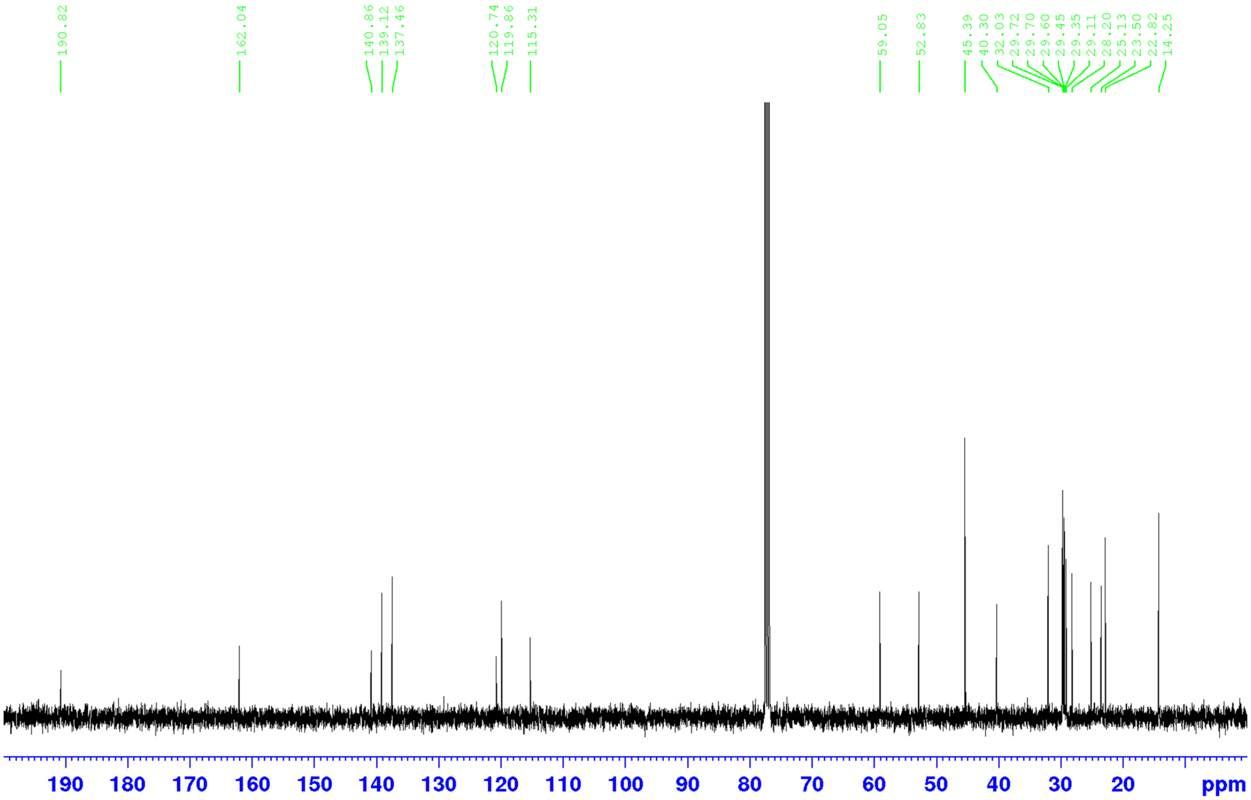

N-(3-(Dimethylamino)propyl)-2-(4-(dodecylsulfonamido)-[1,1'-biphenyl]-3-yl)-2-oxoacetamide (**11c**)

^{1}H NMR (400 MHz, $CDCl_3$):

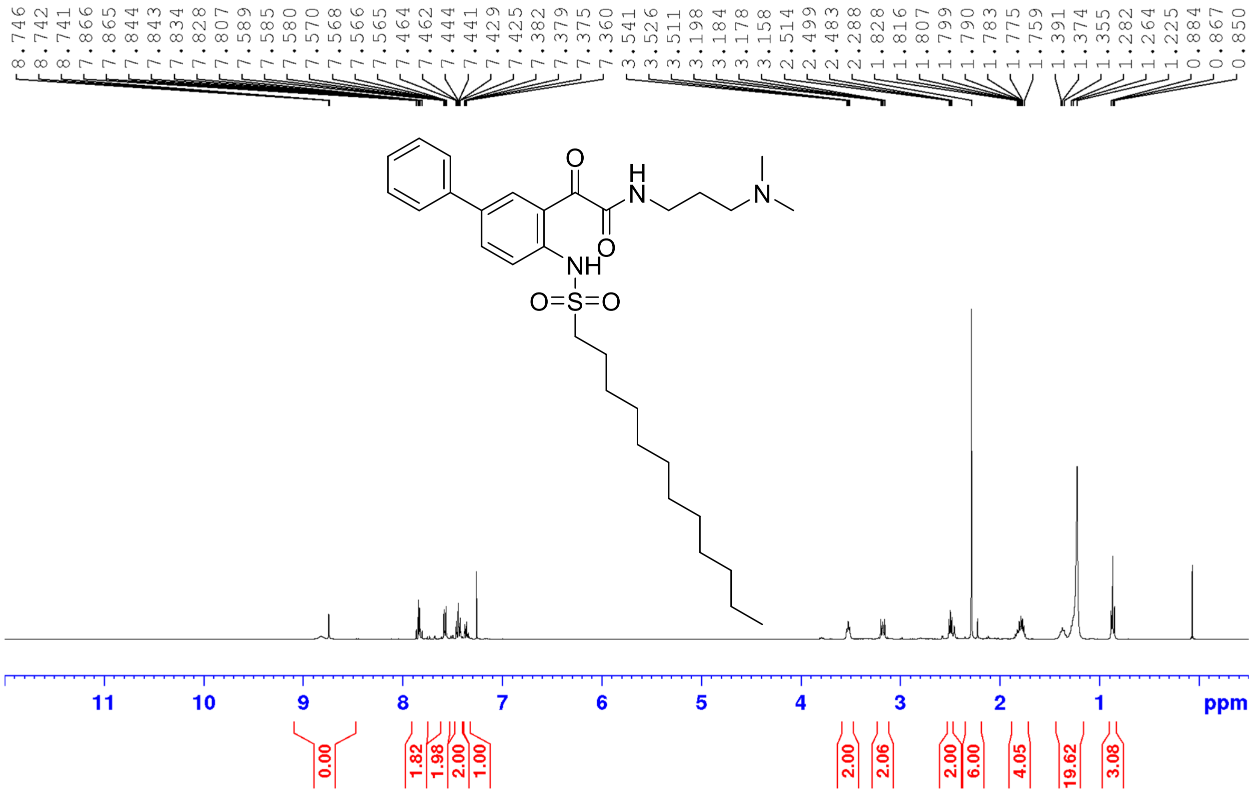

^{13}C NMR (100 MHz, $CDCl_3$):

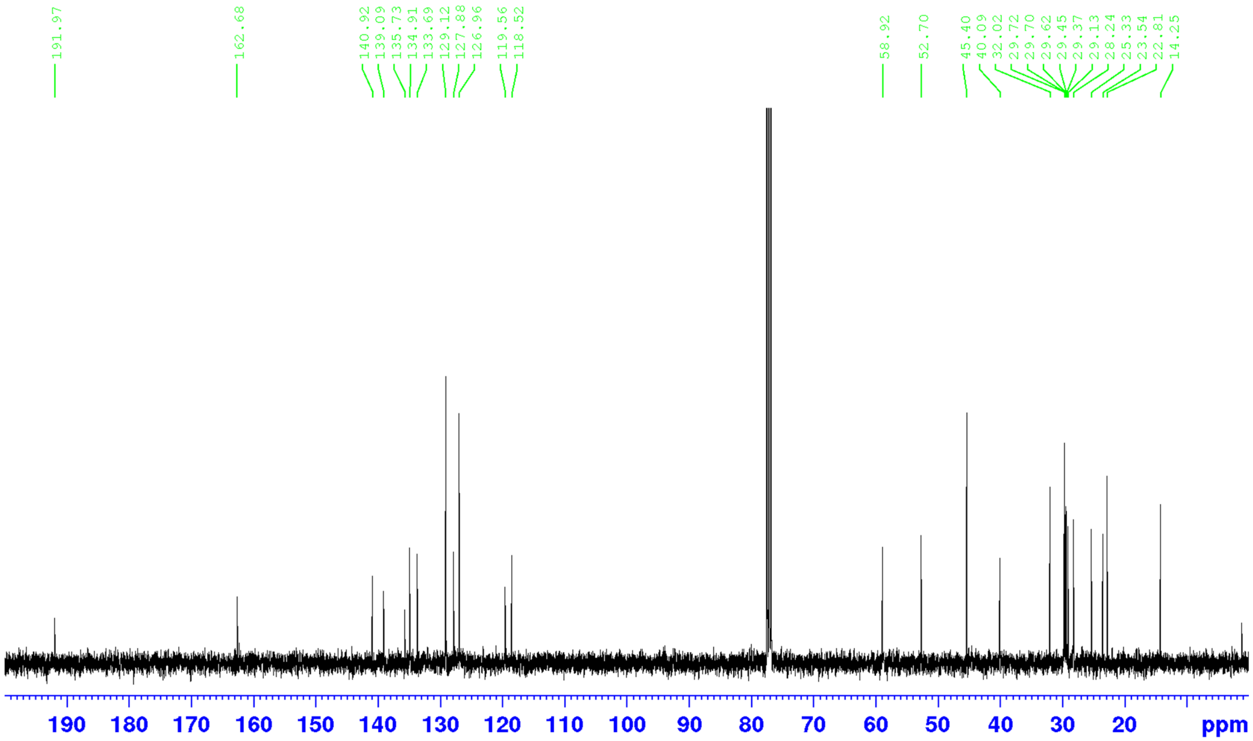

N-(3-(Dimethylamino)propyl)-2-(4-(dodecylsulfonamido)-[1,1':4',1"-terphenyl]-3-yl)-2-oxoacetamide (**11d**)

^{1}H NMR (400 MHz, $CDCl_3$):

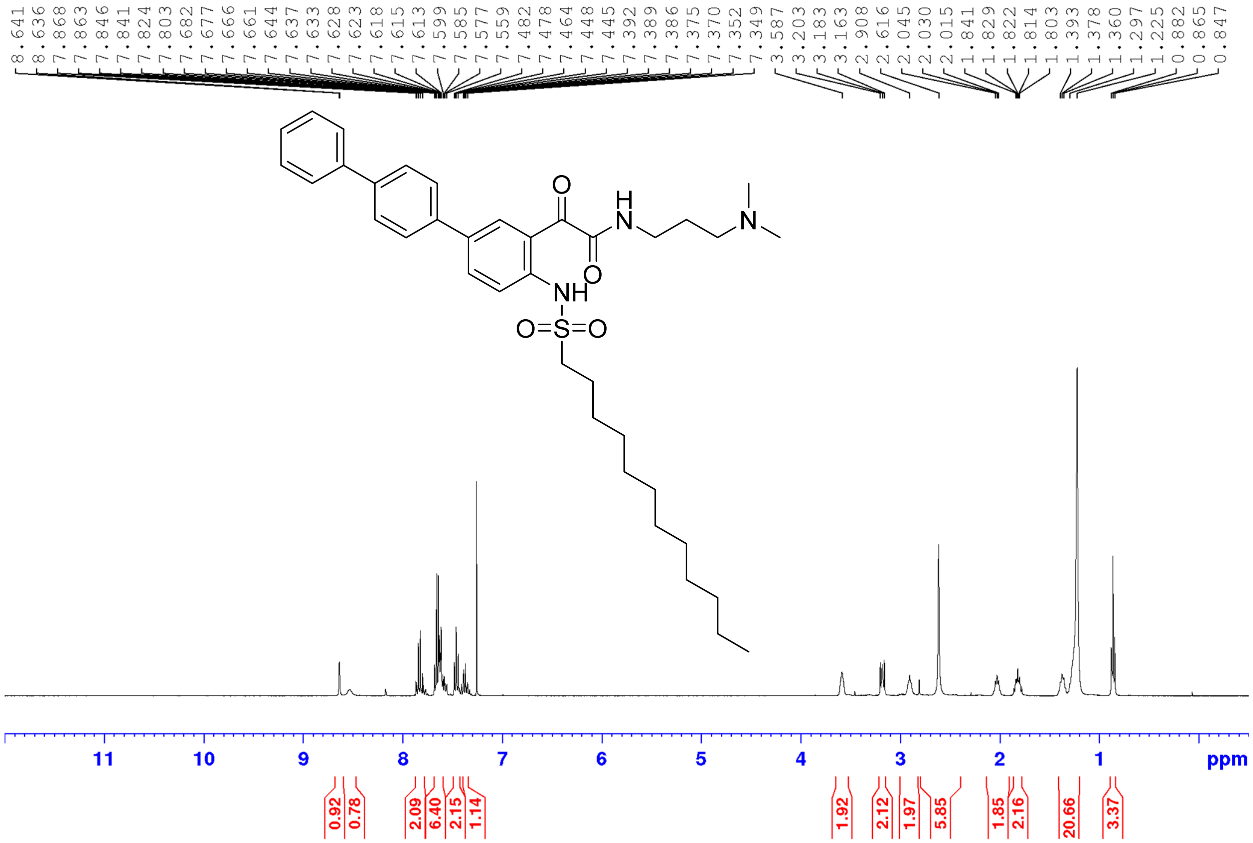

^{13}C NMR (100 MHz, $CDCl_3$):

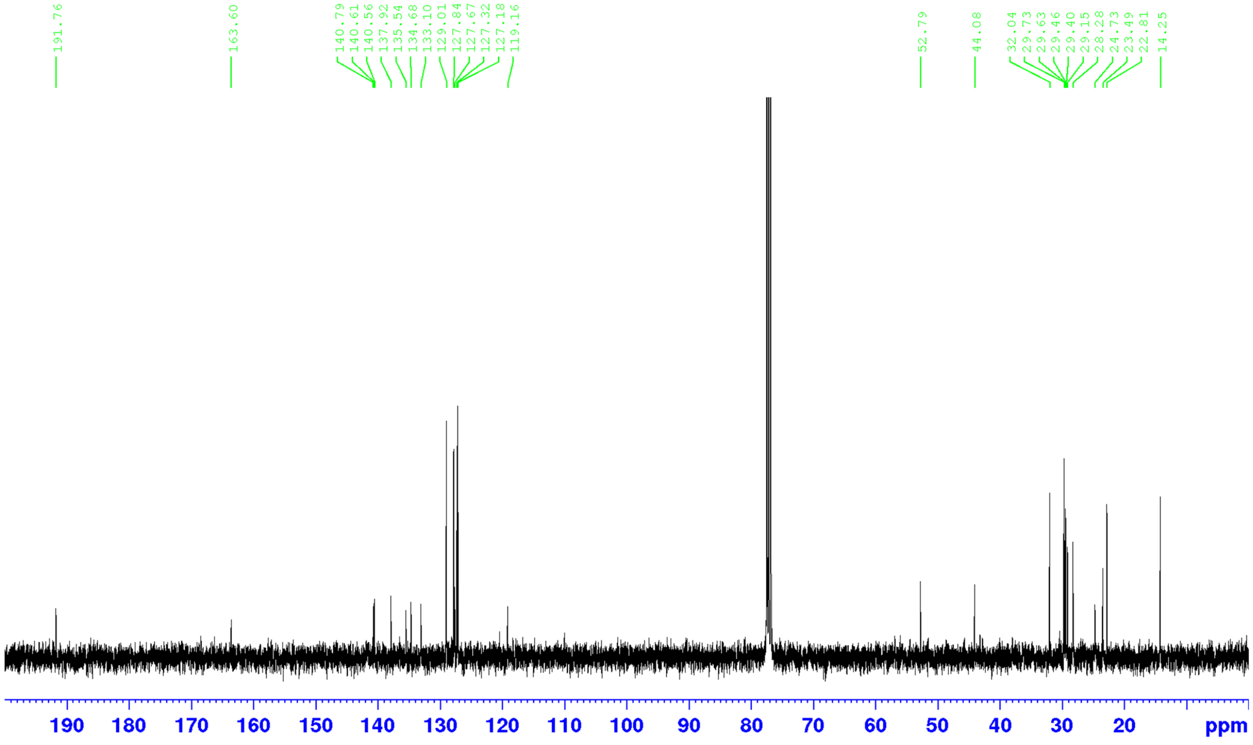

N-(3-(Dimethylamino)propyl)-2-(2-(hexadecylsulfonamido)phenyl)-2-oxoacetamide (**12a**)

^{1}H NMR (400 MHz, $CDCl_3$):

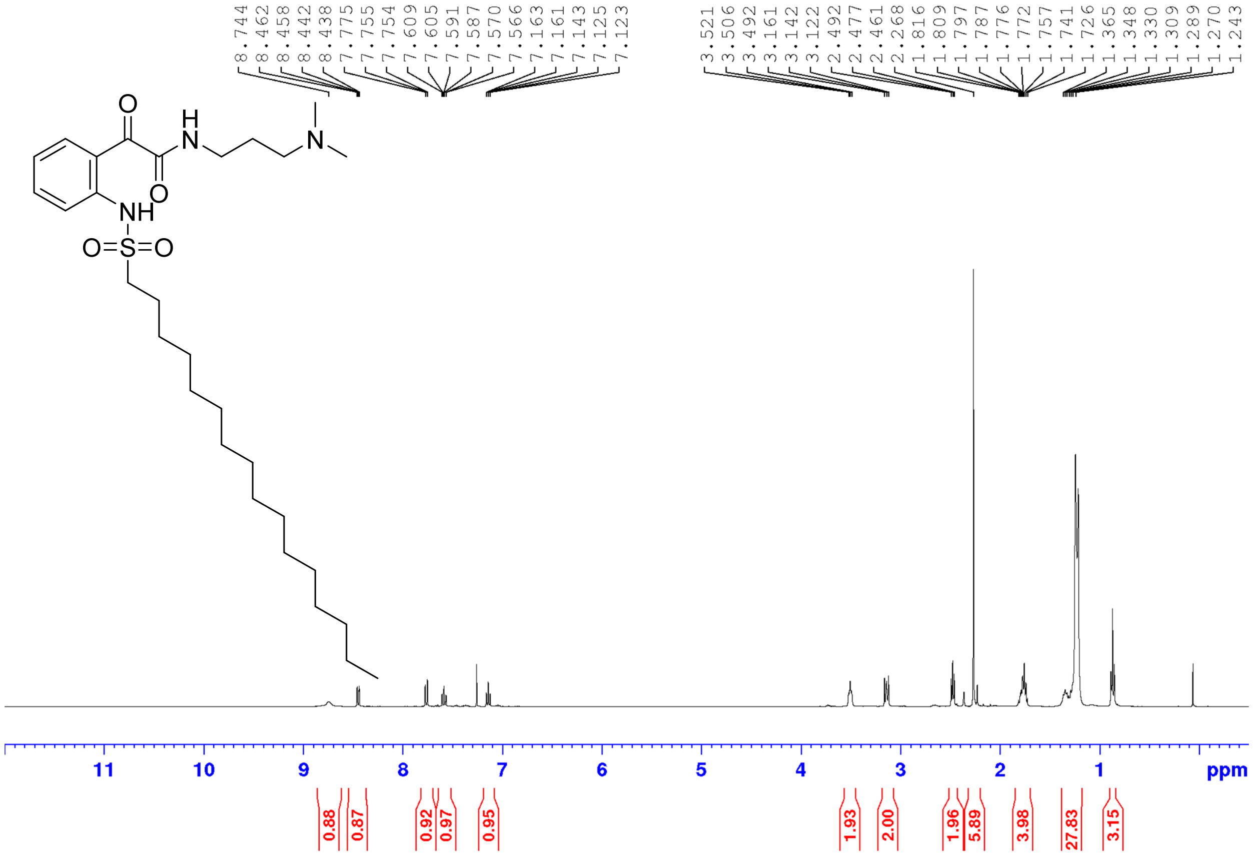

^{13}C NMR (100 MHz, $CDCl_3$):

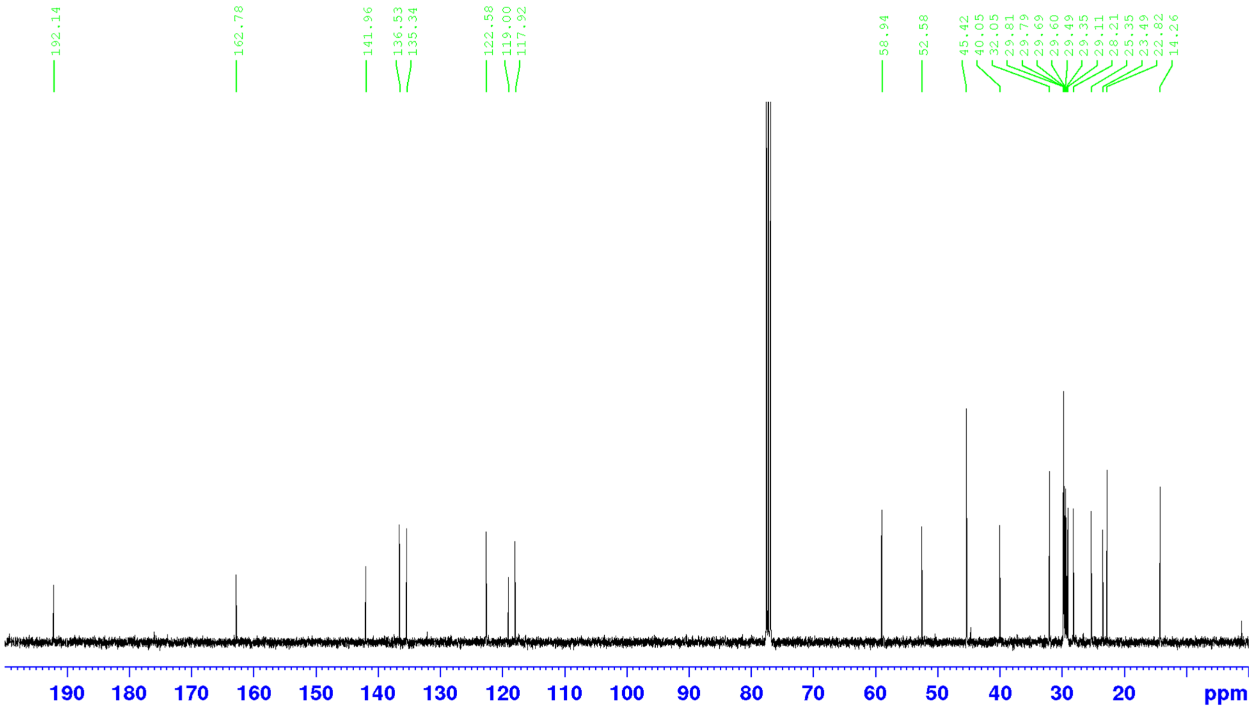

2-(5-Bromo-2-(hexadecylsulfonamido)phenyl)-*N*-(3-(dimethylamino)propyl)-2-oxoacetamide (**12b**)

^{1}H NMR (400 MHz, $CDCl_3$):

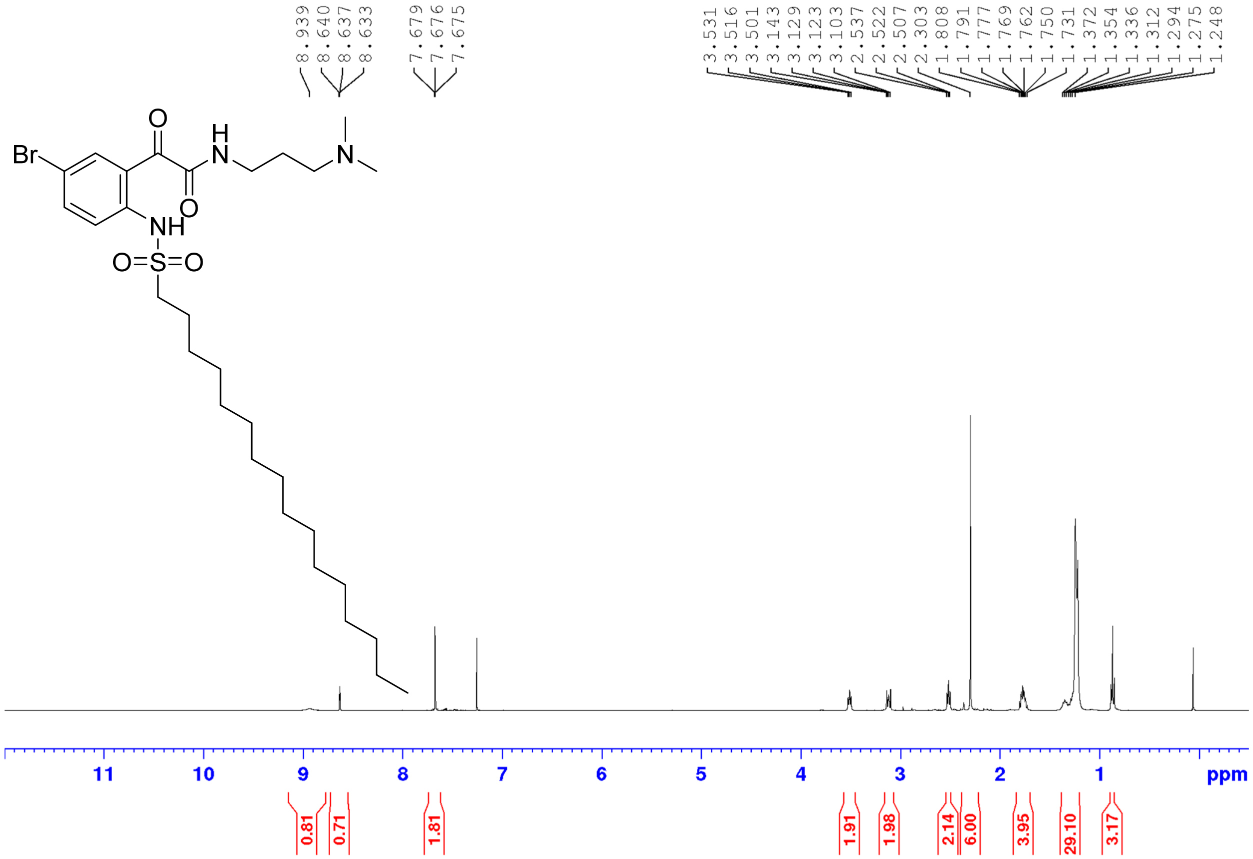

^{13}C NMR (100 MHz, $CDCl_3$):

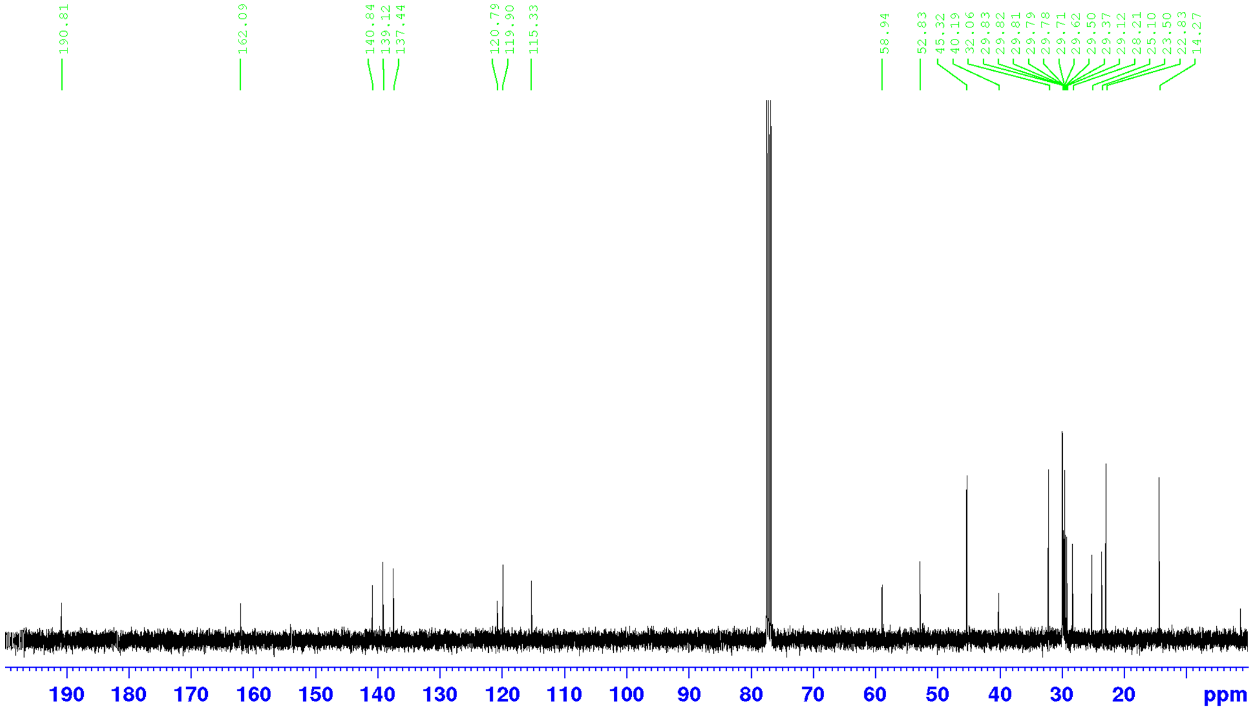

N-(3-(Dimethylamino)propyl)-2-(4-(hexadecylsulfonamido)-[1,1'-biphenyl]-3-yl)-2-oxoacetamide (**12c**)

^{1}H NMR (400 MHz, $CDCl_3$):

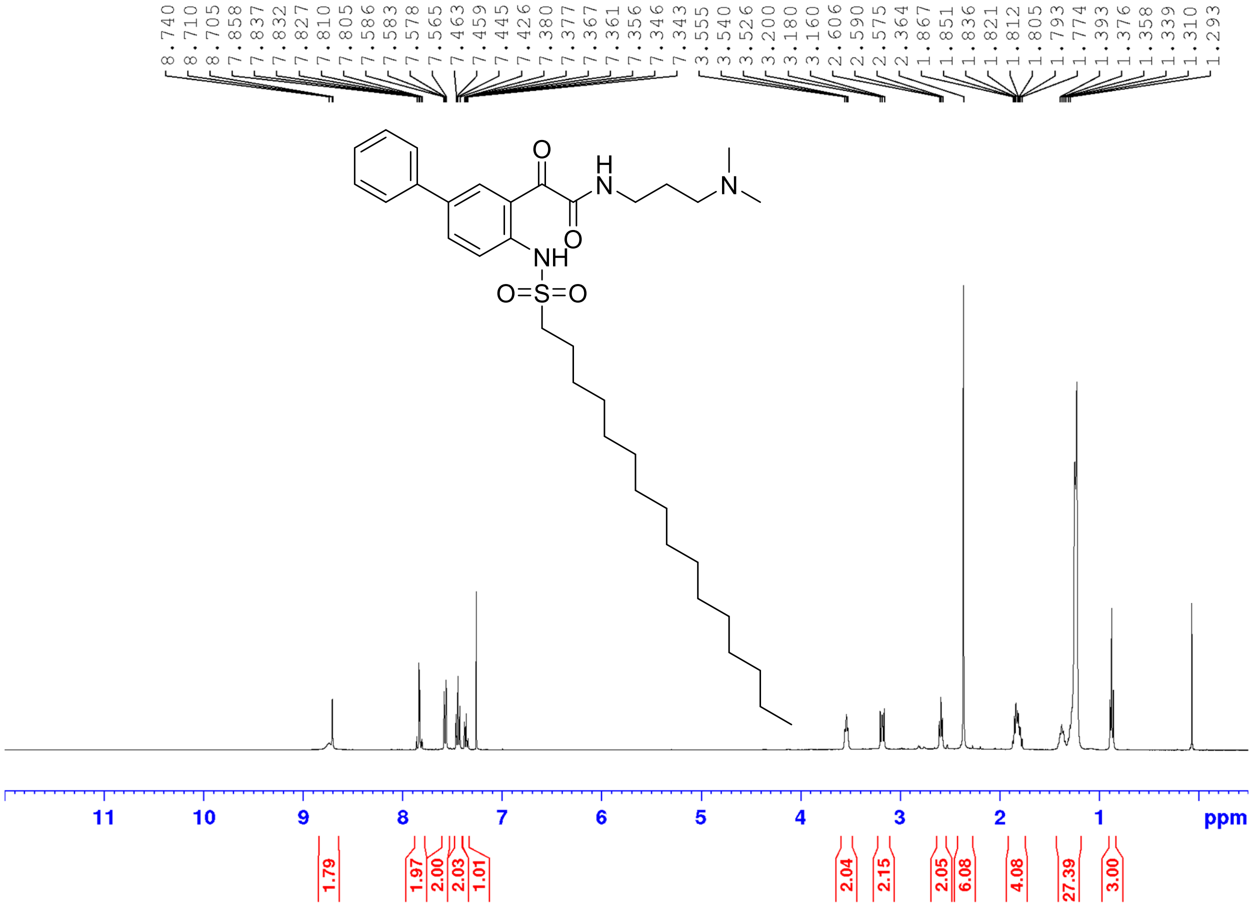

^{13}C NMR (100 MHz, $CDCl_3$):

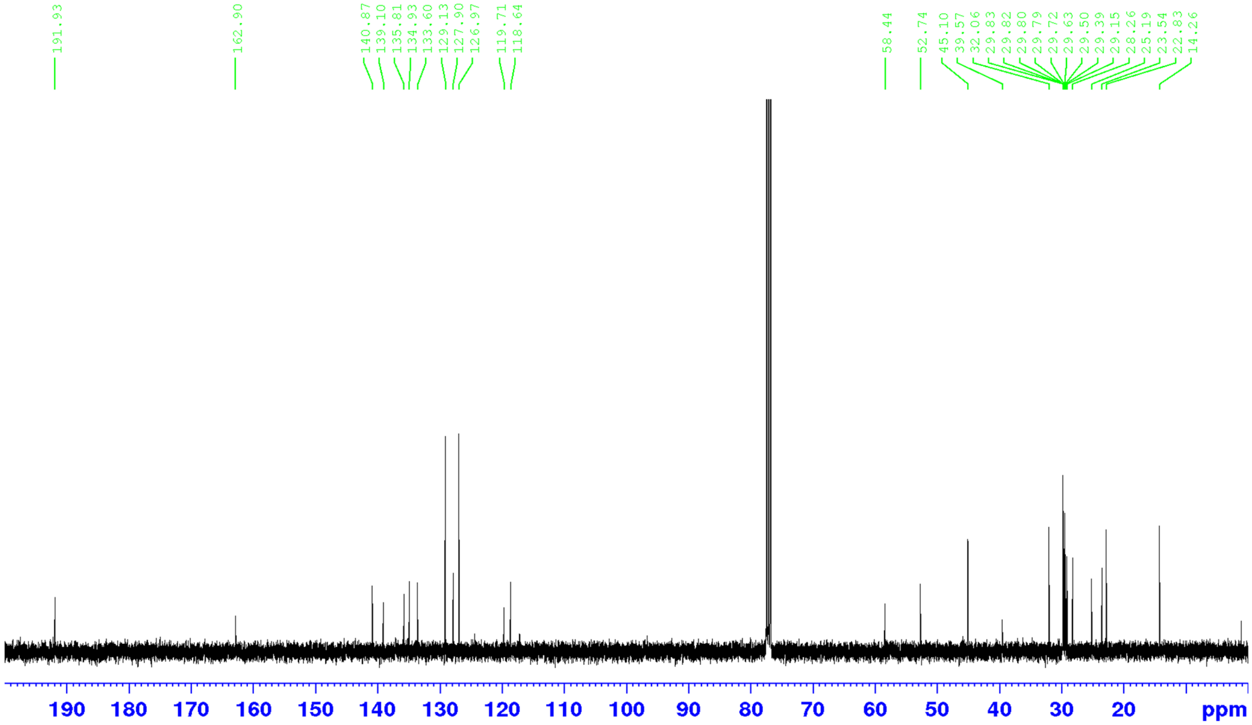

N,*N*-Dimethyl-3-(2-(4-(octylsulfonamido)-[1,1':4',1''-terphenyl]-3-yl)-2-oxoacetamido)propan-1-aminium chloride (**13d**)

^{1}H NMR (600 MHz, DMSO-d_6):

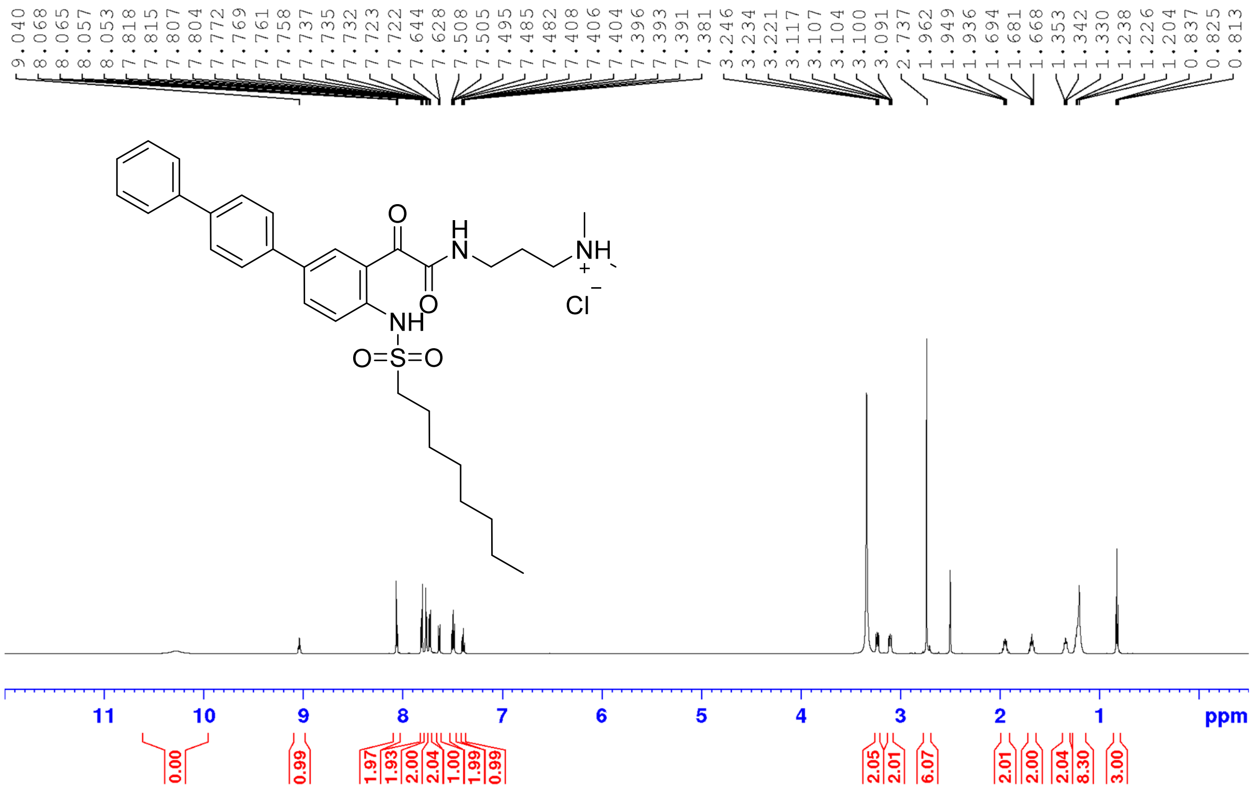

^{13}C NMR (150 MHz, DMSO-d_6):

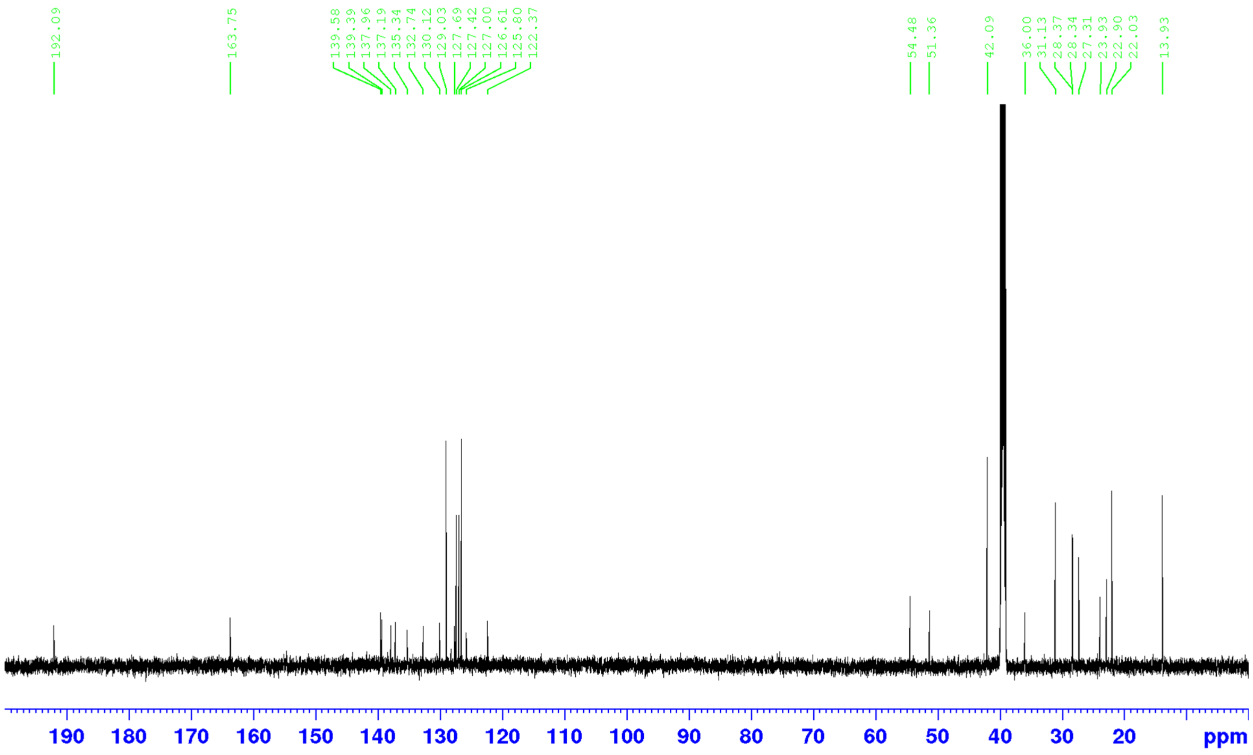

3-(2-(2-(Dodecylsulfonamido)phenyl)-2-oxoacetamido)-*N*,*N*-dimethylpropan-1-aminium chloride (**14a**)

^{1}H NMR (600 MHz, DMSO-d_6):

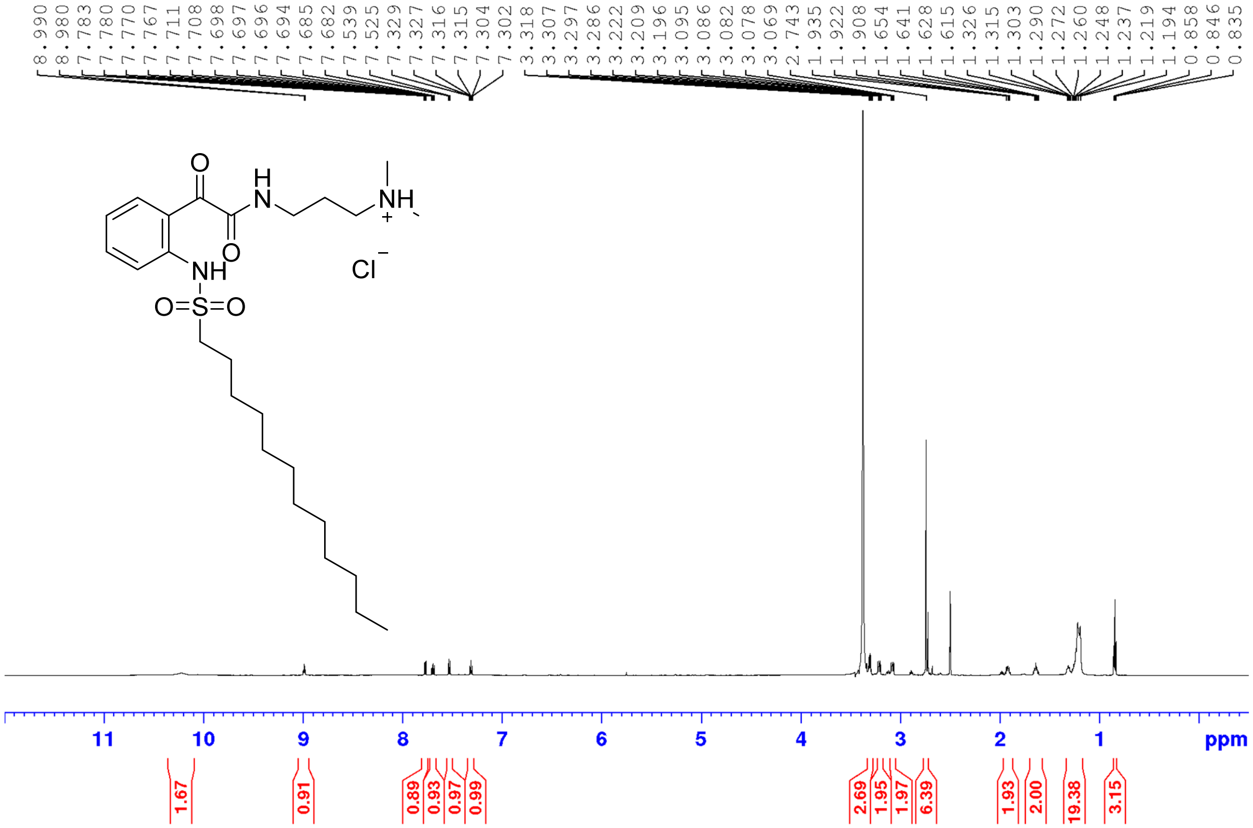

^{13}C NMR (150 MHz, DMSO-d_6):

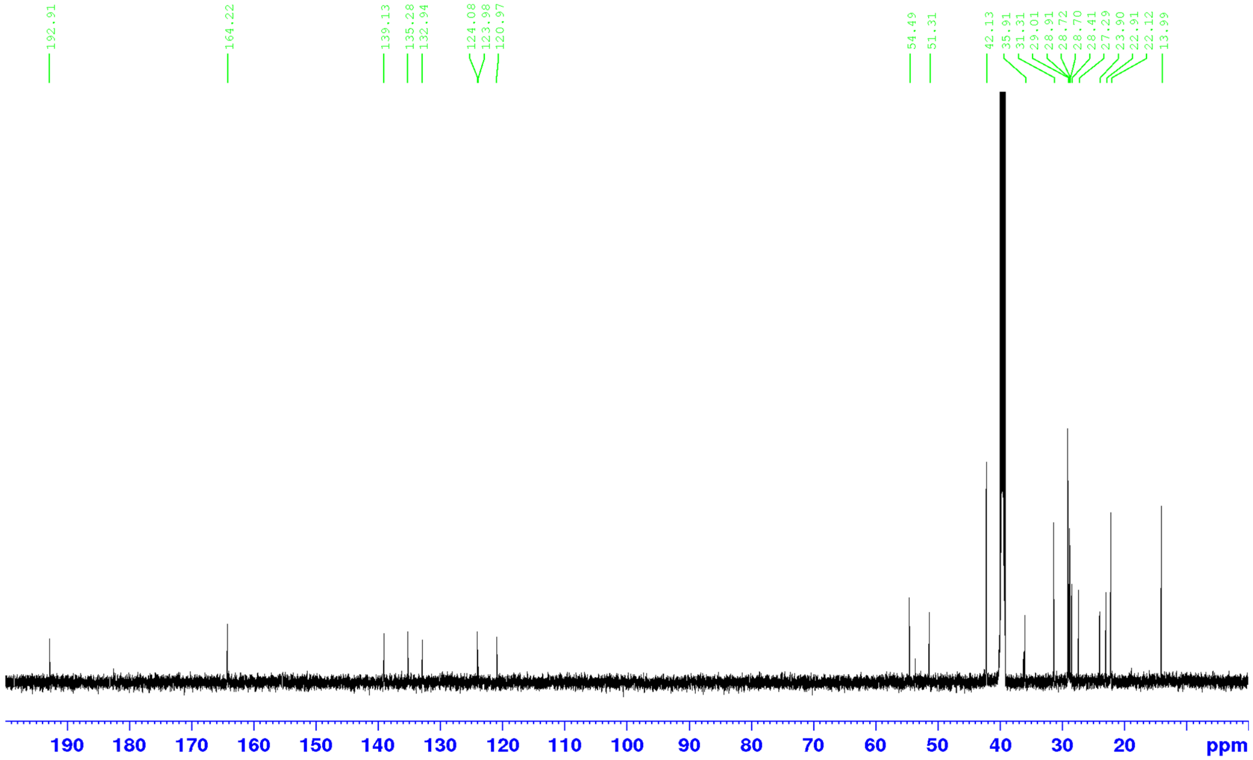

3-(2-(5-Bromo-2-(dodecylsulfonamido)phenyl)-2-oxoacetamido)-*N*,*N*-dimethylpropan-1-aminium chloride (**14b**)

^{1}H NMR (600 MHz, DMSO-d_6):

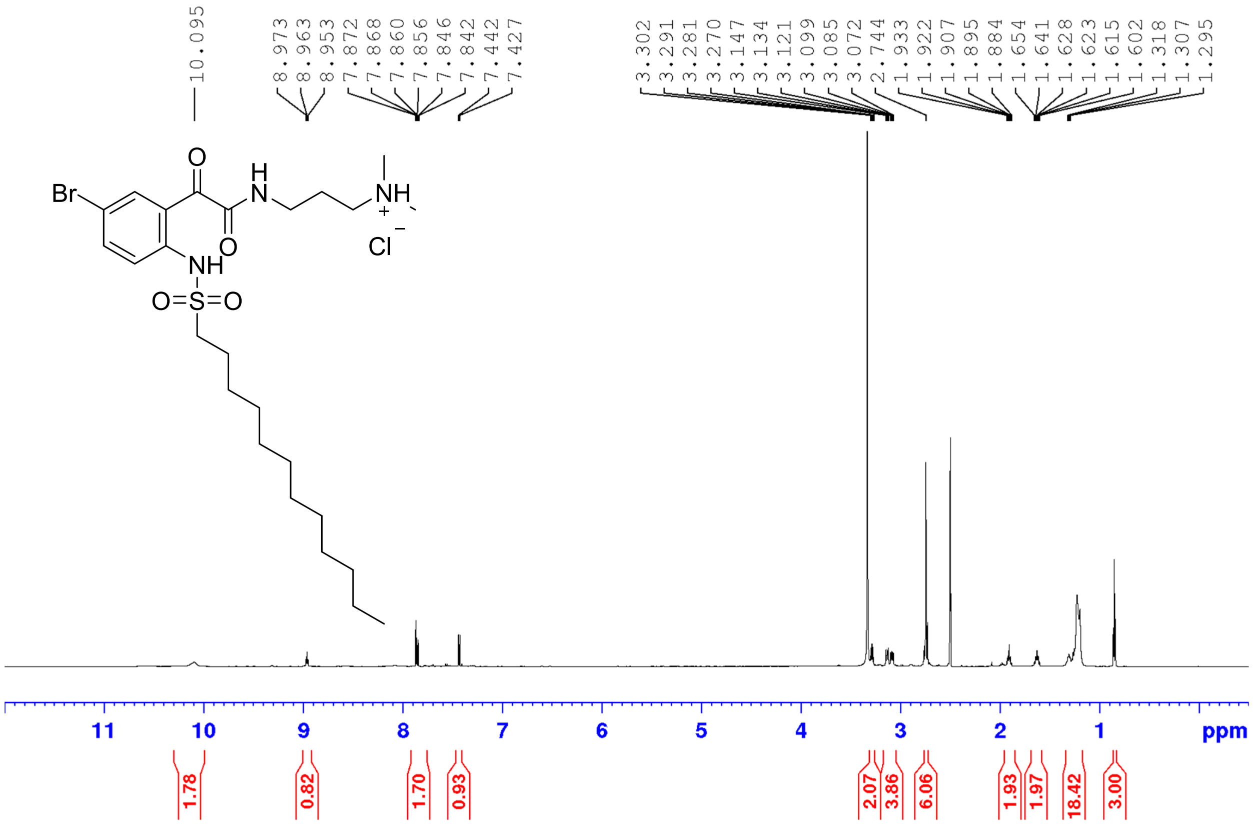

^{13}C NMR (150 MHz, DMSO-d_6):

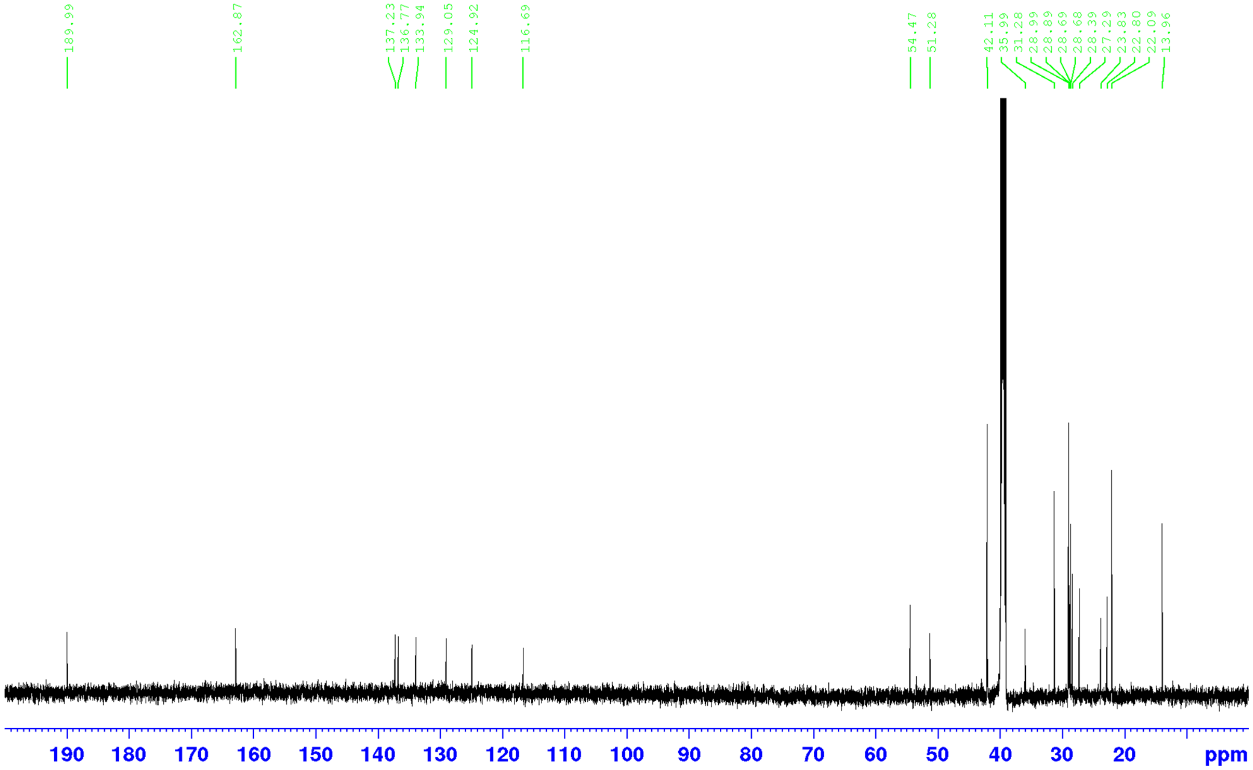

3-(2-(4-(Dodecylsulfonamido)-[1,1'-biphenyl]-3-yl)-2-oxoacetamido)-*N*,*N*-dimethylpropan-1-aminium chloride (**14c**)

^{1}H NMR (600 MHz, DMSO-d_6):

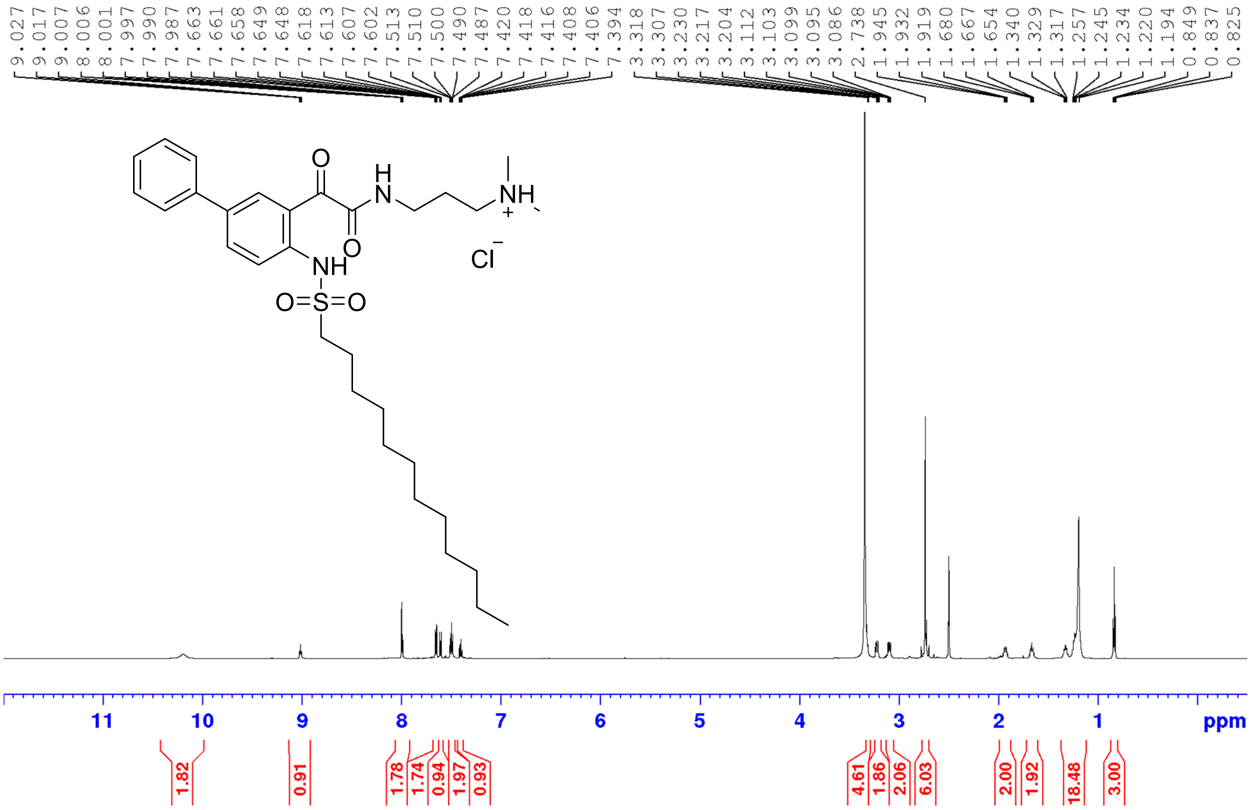

^{13}C NMR (150 MHz, DMSO-d_6):

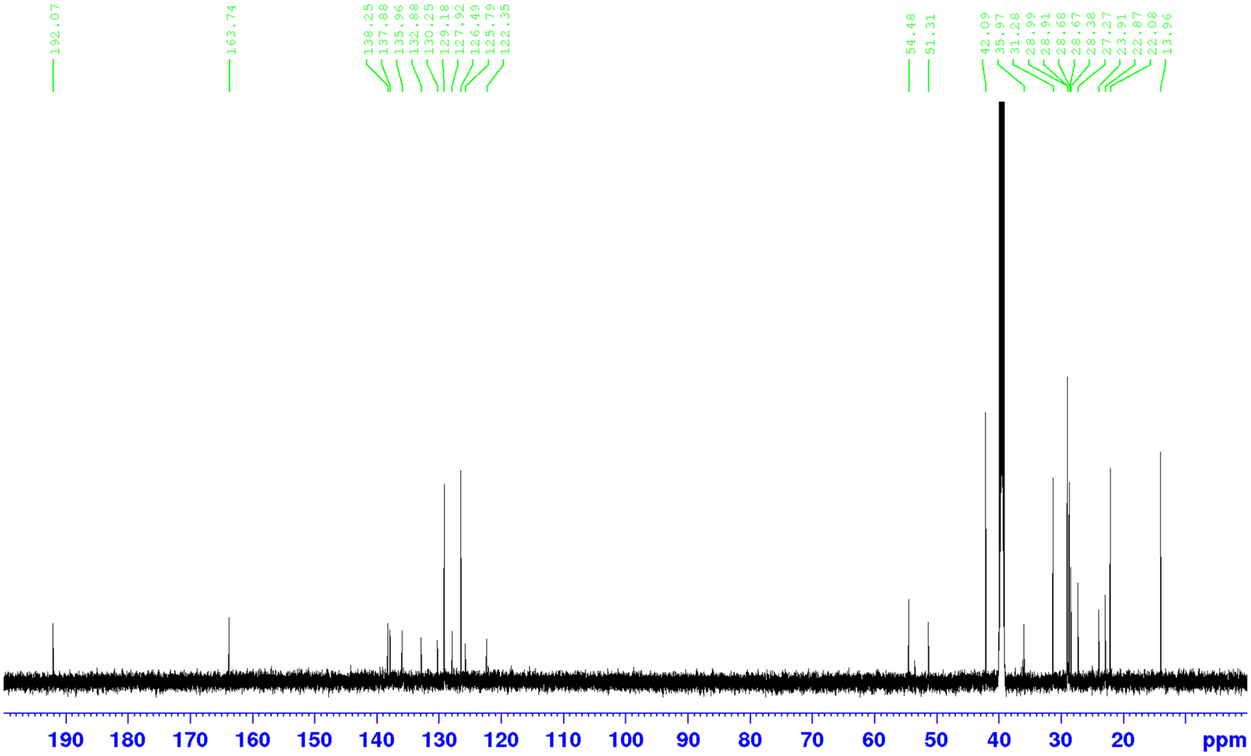

3-(2-(4-(Dodecylsulfonamido)-[1,1':4',1''-terphenyl]-3-yl)-2-oxoacetamido)-*N*,*N*-dimethylpropan-1-aminium chloride (**14d**)

^{1}H NMR (600 MHz, DMSO-d_6):

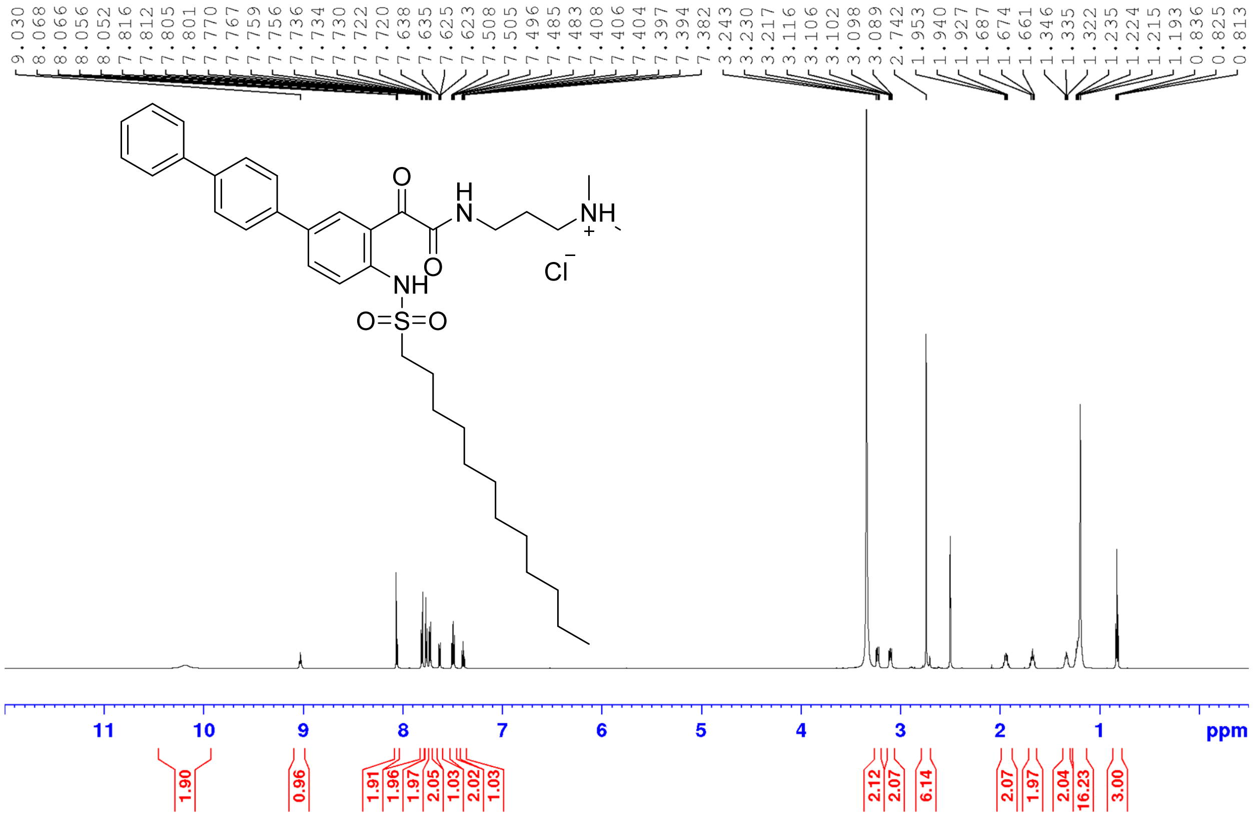

^{13}C NMR (150 MHz, DMSO-d_6):

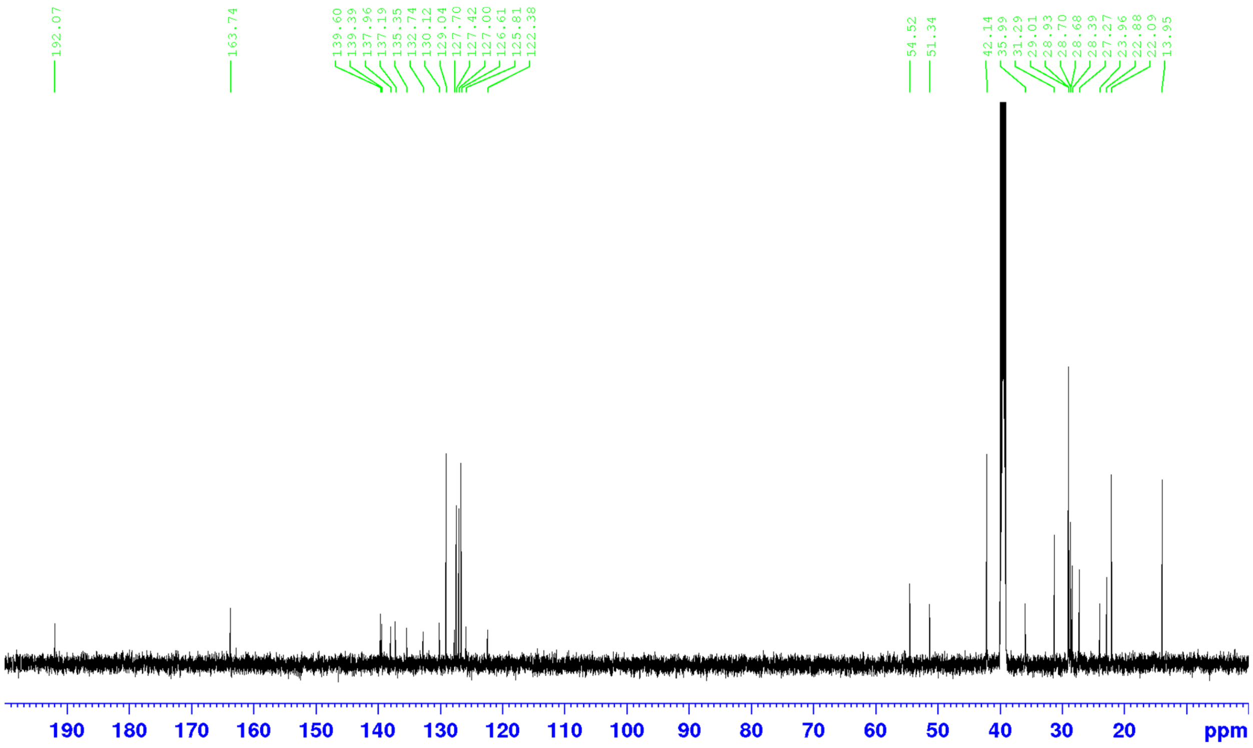

3-(2-(2-(Hexadecylsulfonamido)phenyl)-2-oxoacetamido)-*N*,*N*-dimethylpropan-1-aminium chloride (**15a**)

^{1}H NMR (600 MHz, DMSO-*d*$_6$):

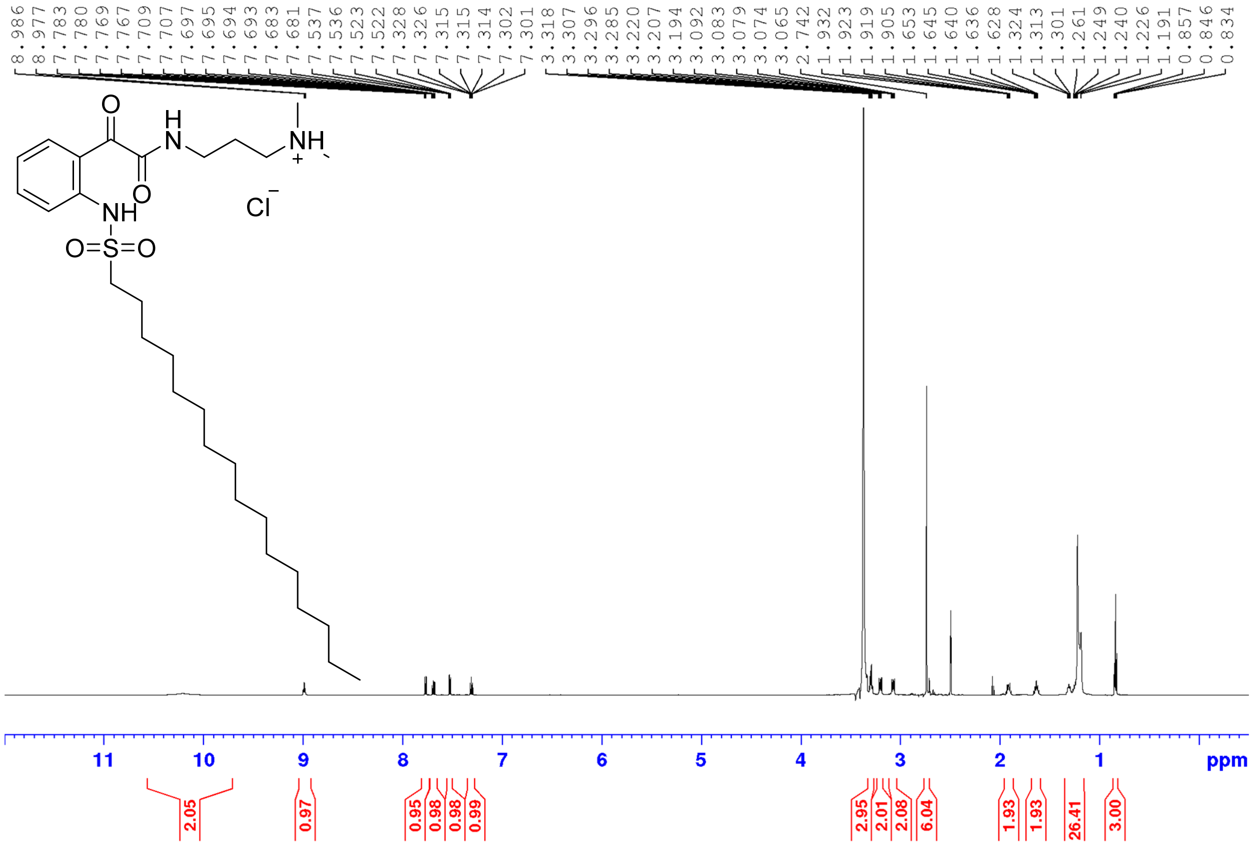

^{13}C NMR (150 MHz, DMSO-*d*$_6$):

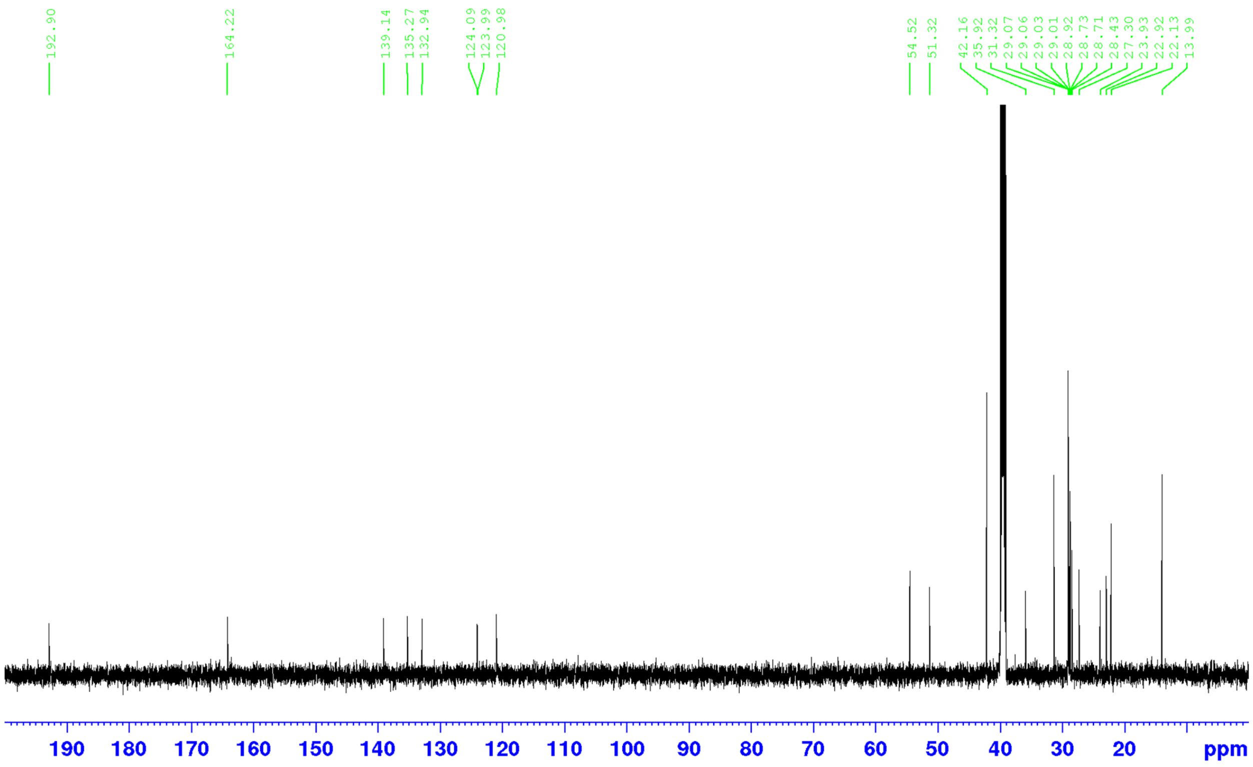

3-(2-(5-Bromo-2-(hexadecylsulfonamido)phenyl)-2-oxoacetamido)-*N*,*N*-dimethylpropan-1-aminium chloride (**15b**)

^{1}H NMR (600 MHz, DMSO-d_6):

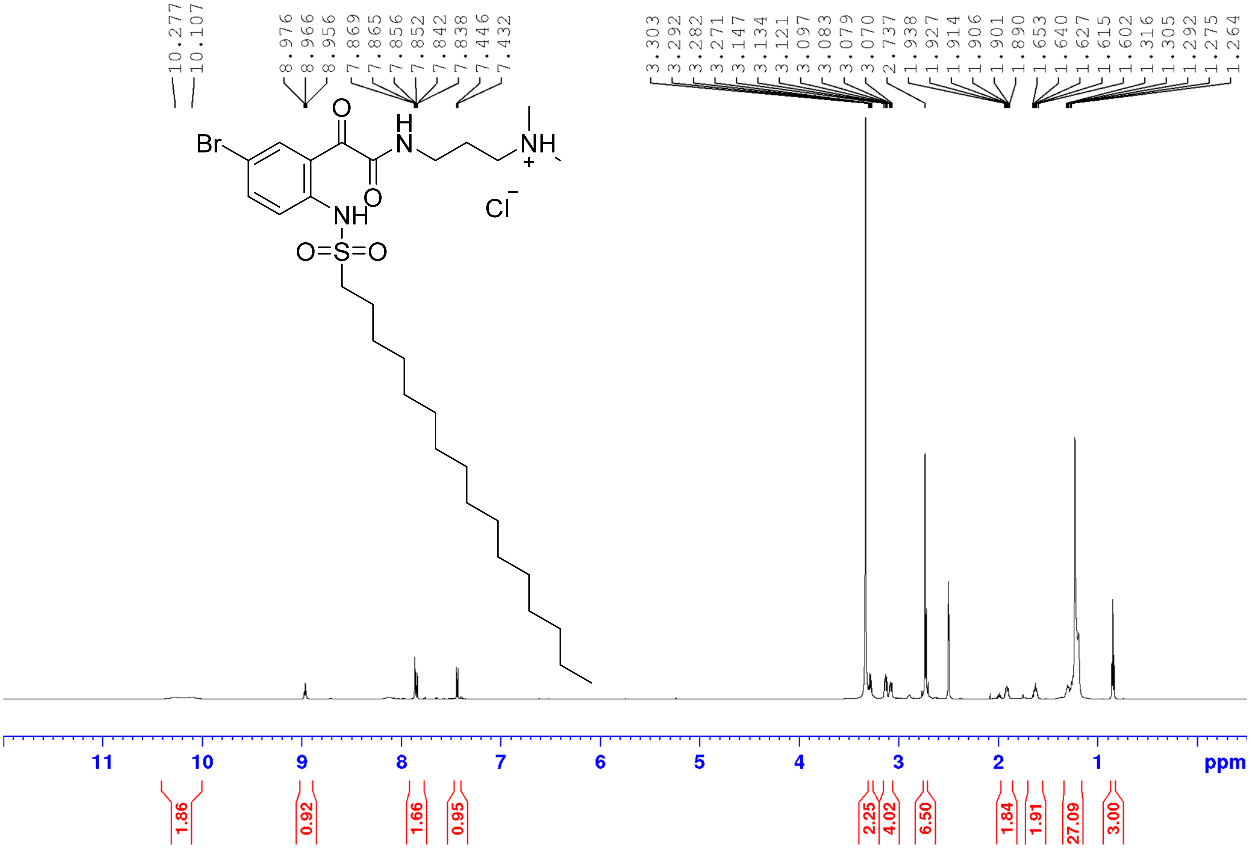

^{13}C NMR (150 MHz, DMSO-d_6):

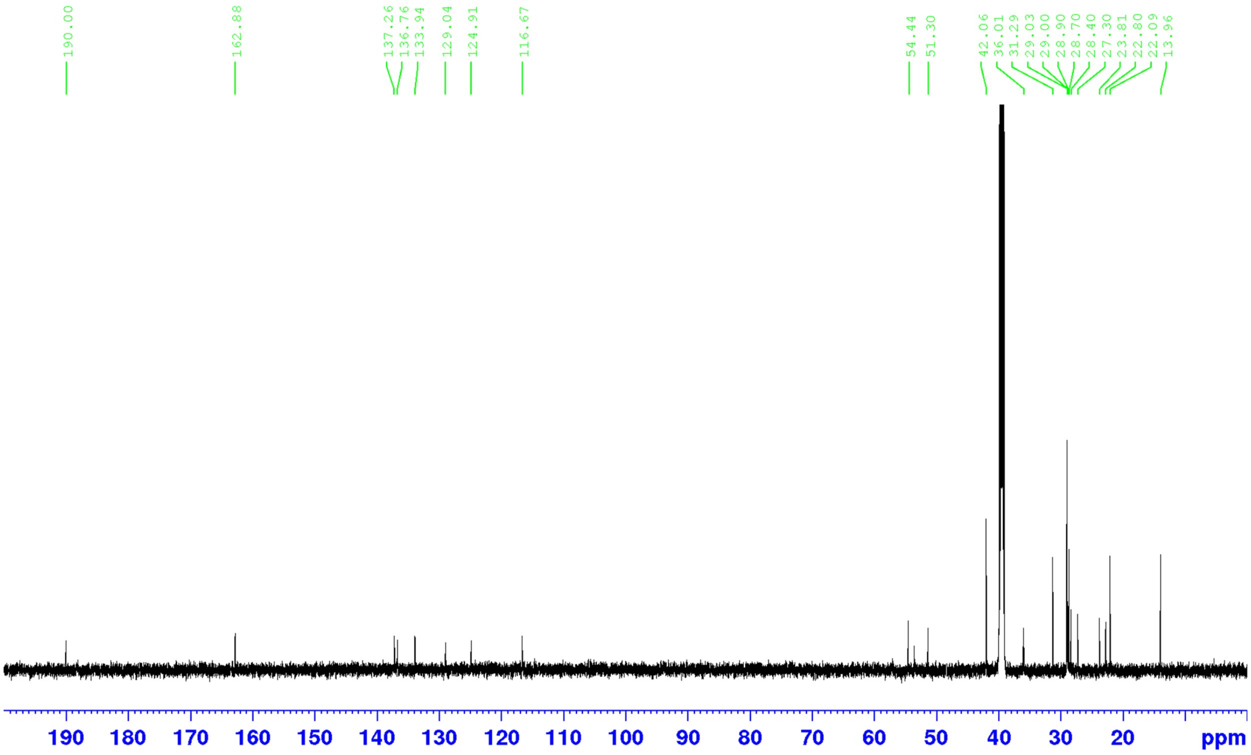

3-(2-(4-(Hexadecylsulfonamido)-[1,1'-biphenyl]-3-yl)-2-oxoacetamido)-*N*,*N*-dimethylpropan-1-aminium chloride (**15c**)

^{1}H NMR (600 MHz, DMSO-d_6):

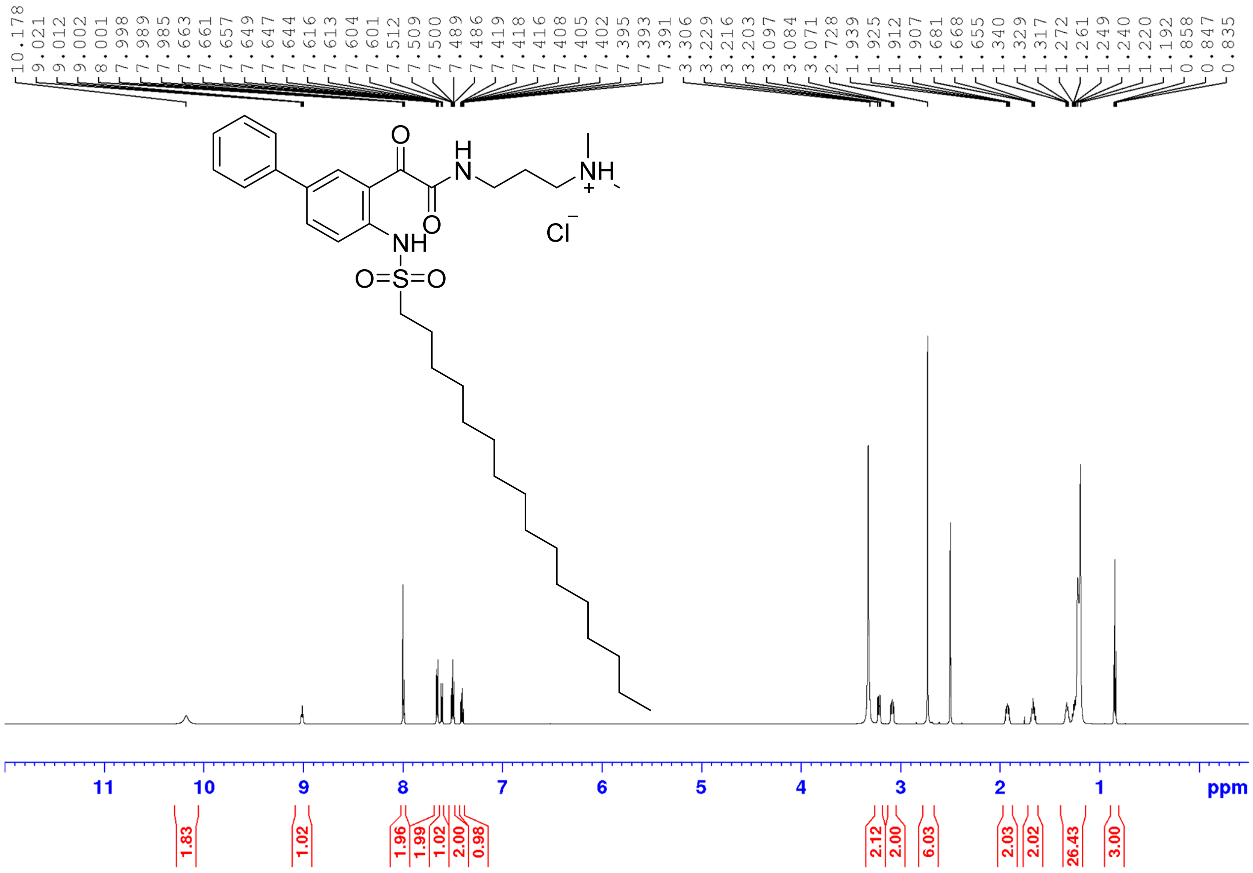

^{13}C NMR (150 MHz, DMSO-d_6):

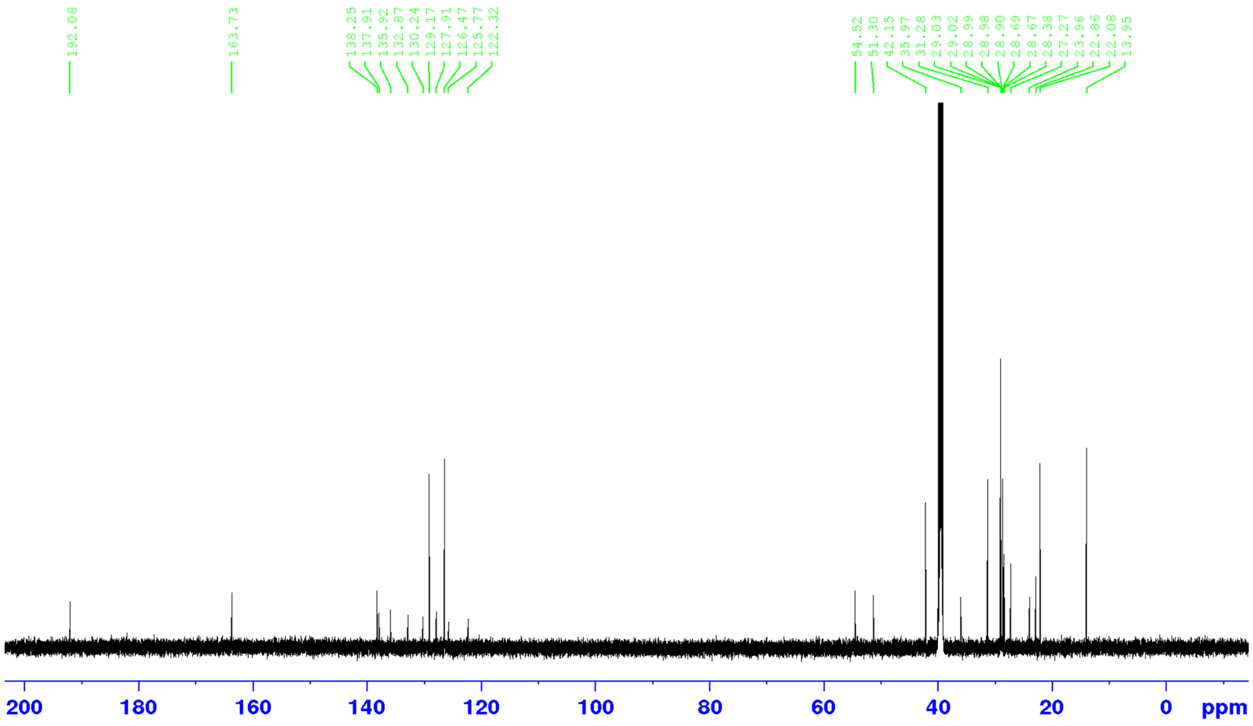

N,*N*,*N*-Trimethyl-3-(2-(4-(octylsulfonamido)-[1,1':4',1"-terphenyl]-3-yl)-2-oxoacetamido)propan-1-aminium iodide (**16d**)

^{1}H NMR (600 MHz, DMSO-d_6):

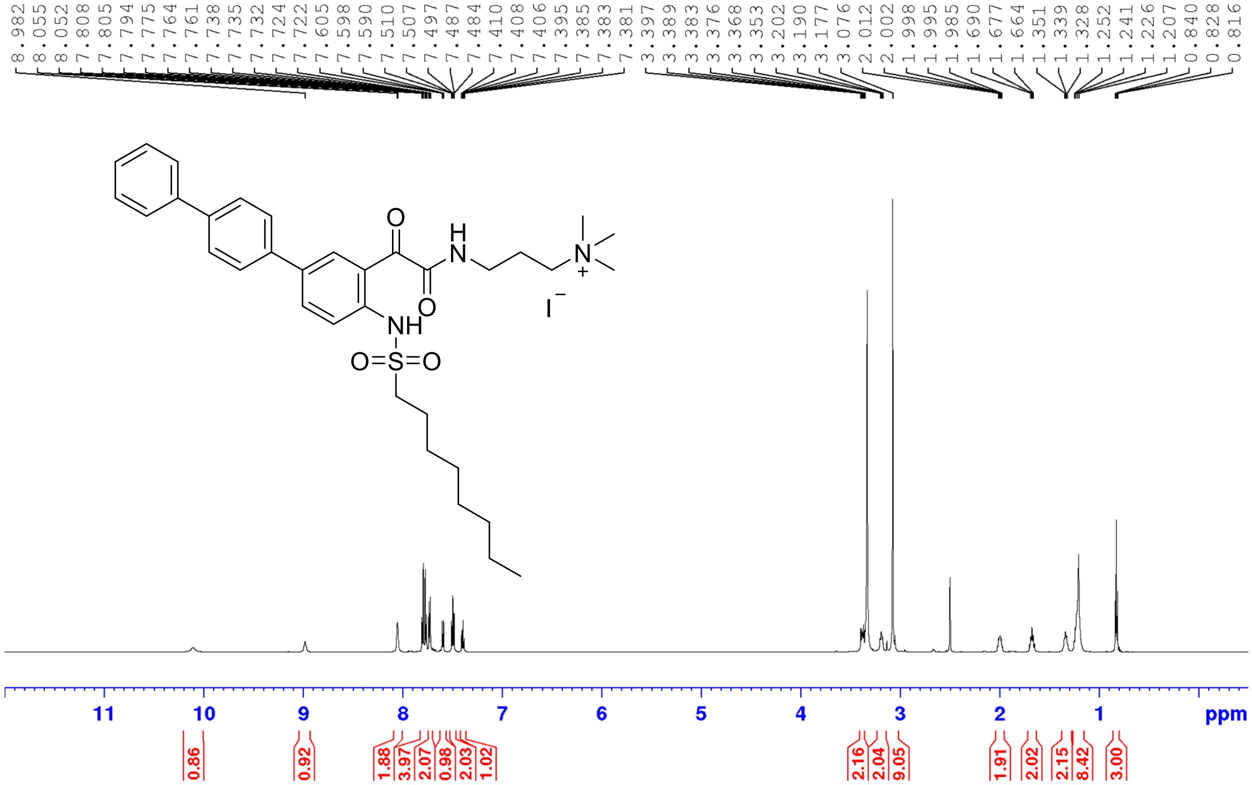

^{13}C NMR (150 MHz, DMSO-d_6):

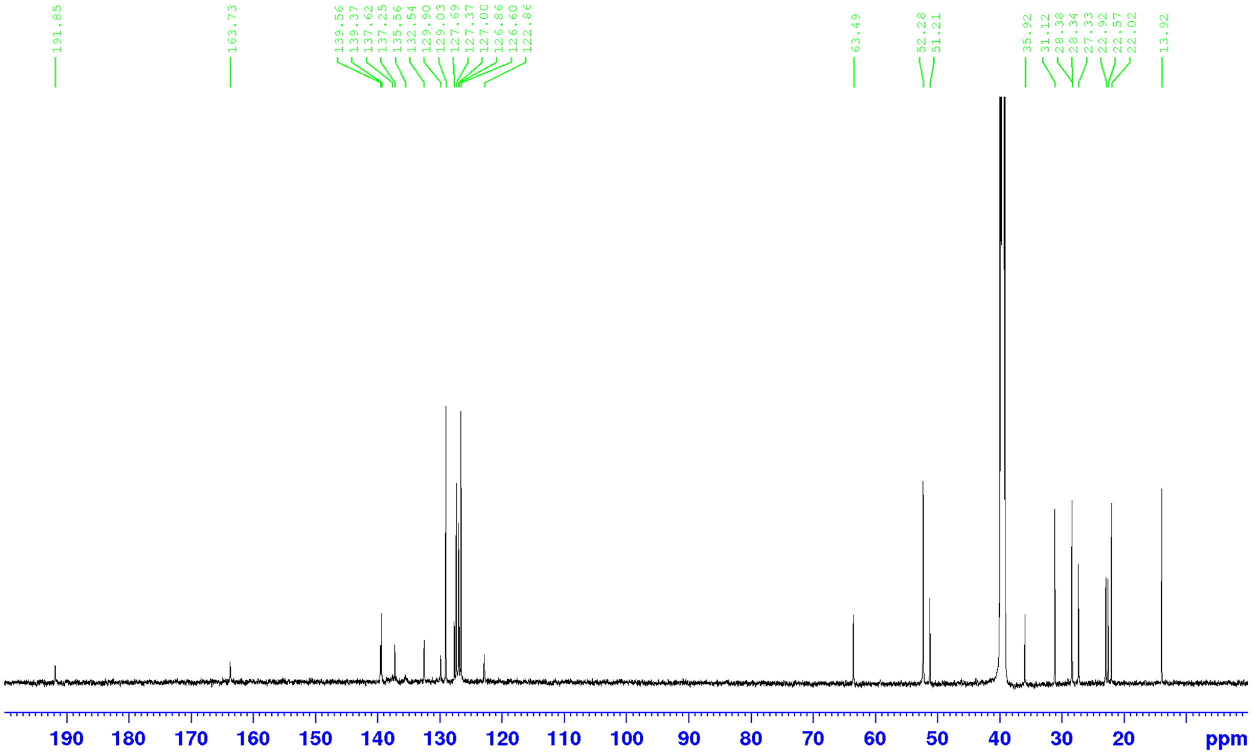

3-(2-(2-(Dodecylsulfonamido)phenyl)-2-oxoacetamido)-*N*,*N*,*N*-trimethylpropan-1-aminium iodide (**17a**)

^{1}H NMR (600 MHz, DMSO-d_6):

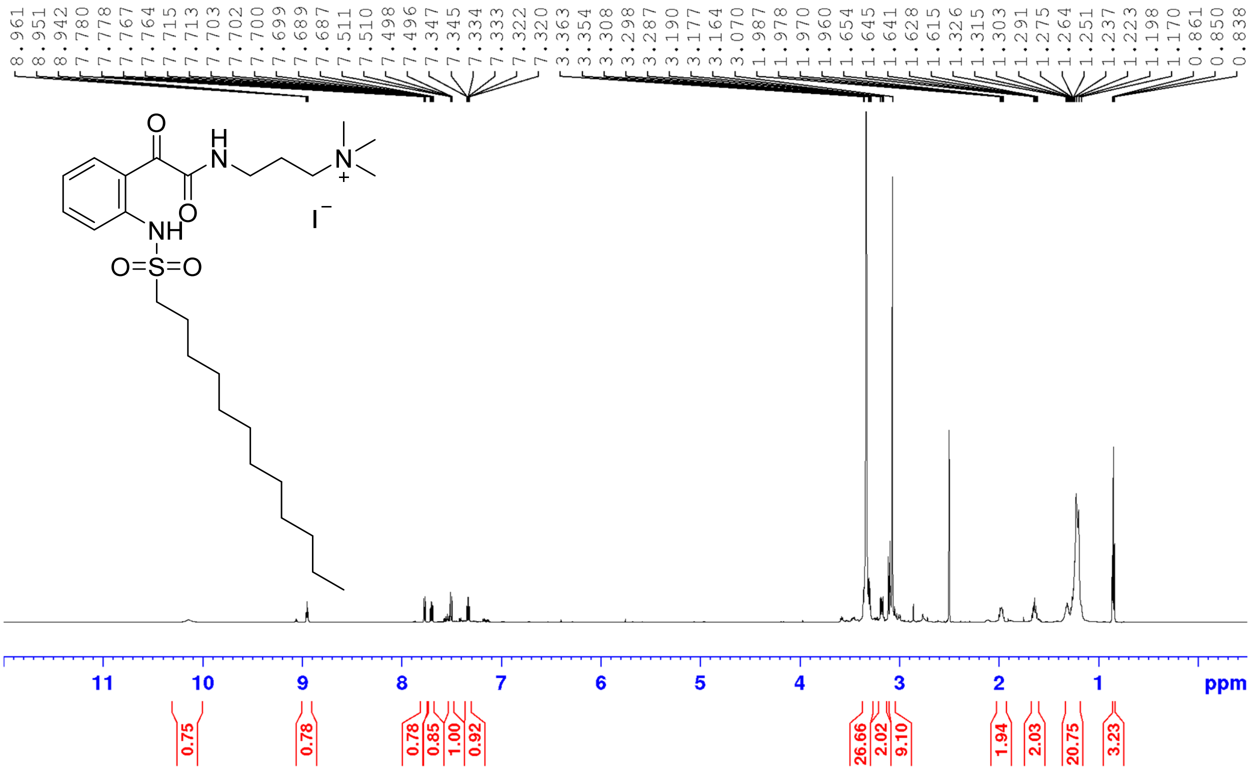

^{13}C NMR (150 MHz, DMSO-d_6):

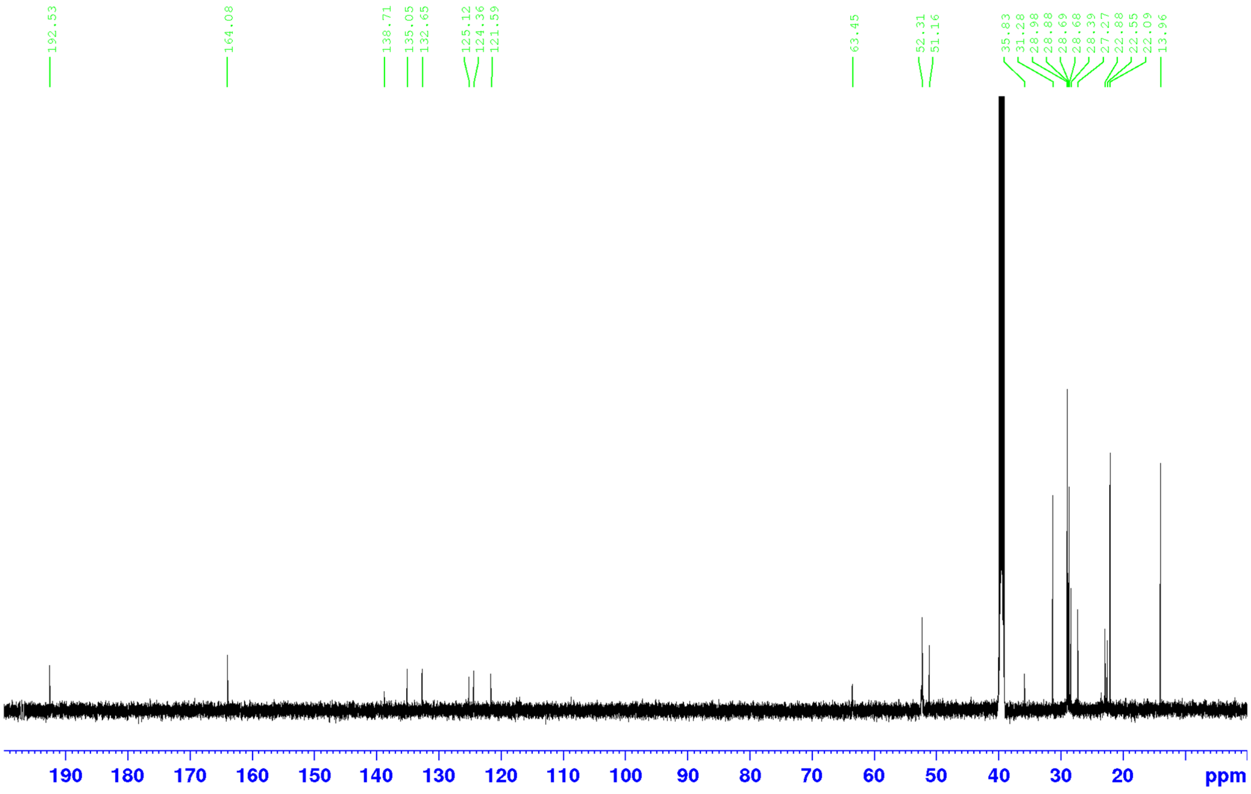

3-(2-(5-Bromo-2-(dodecylsulfonamido)phenyl)-2-oxoacetamido)-*N*,*N*,*N*-trimethylpropan-1-aminium iodide (**17b**)

^{1}H NMR (600 MHz, DMSO-d_6):

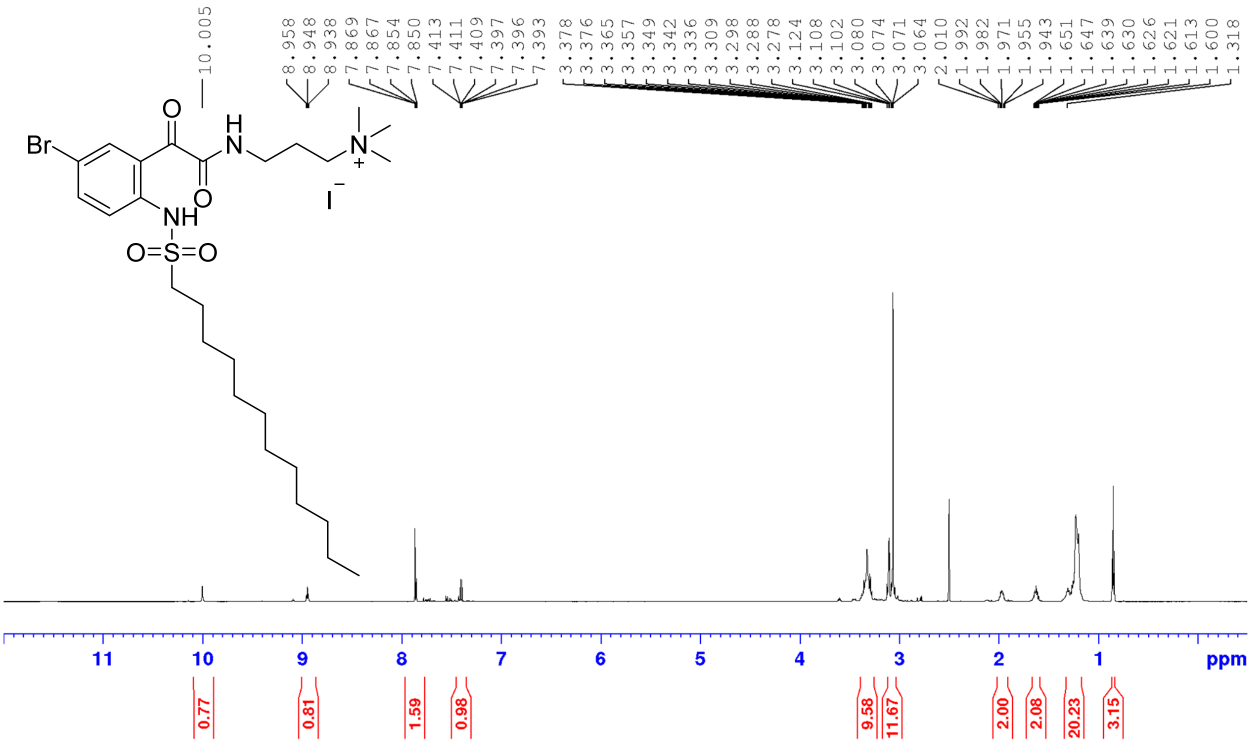

^{13}C NMR (150 MHz, DMSO-d_6):

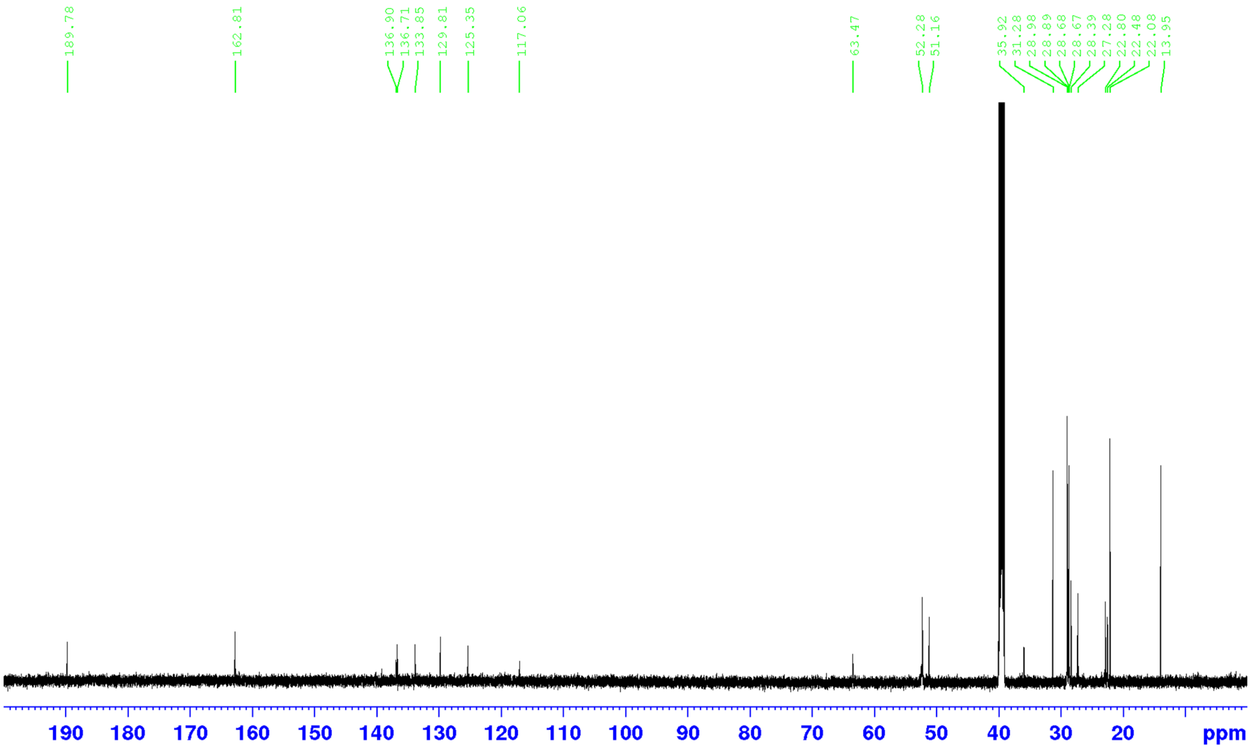

3-(2-(4-(Dodecylsulfonamido)-[1,1'-biphenyl]-3-yl)-2-oxoacetamido)-*N*,*N*,*N*-trimethylpropan-1-aminium iodide (**17c**)

^{1}H NMR (600 MHz, DMSO-d_6):

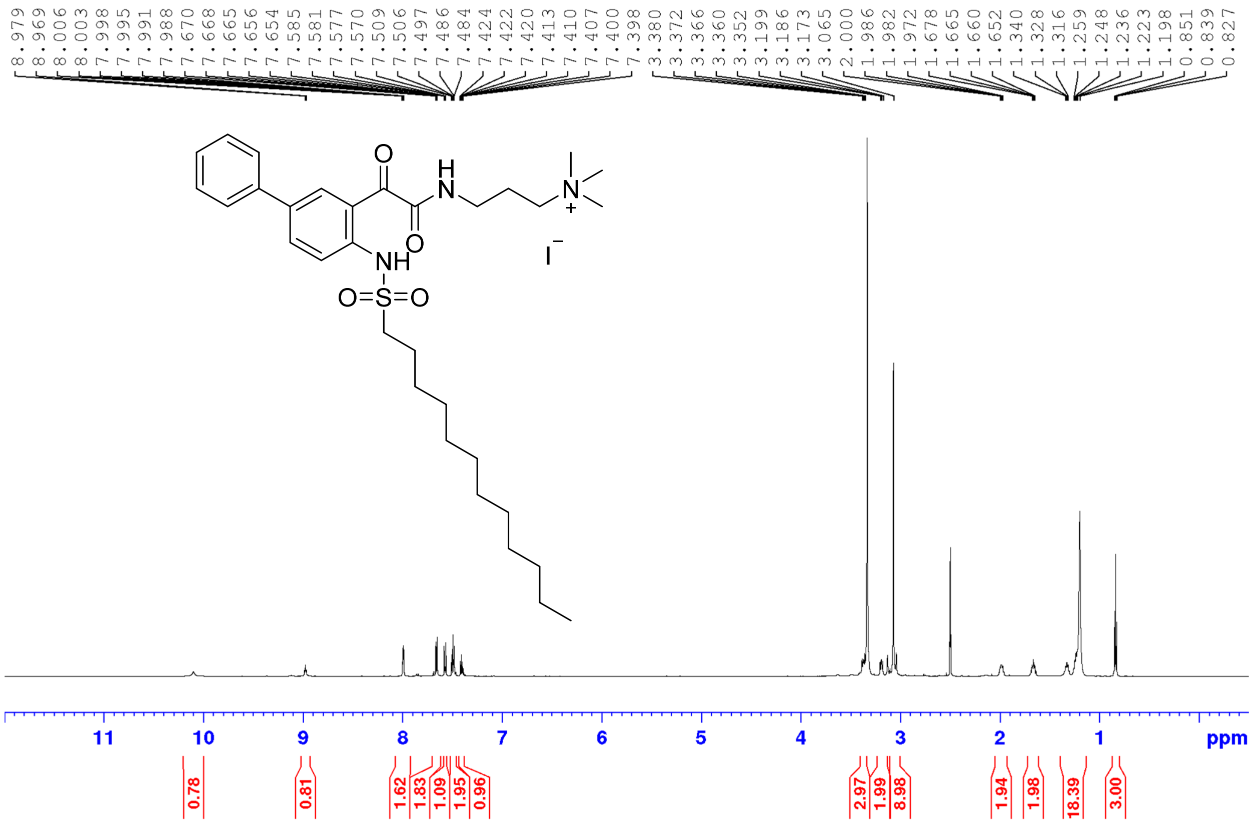

^{13}C NMR (150 MHz, DMSO-d_6):

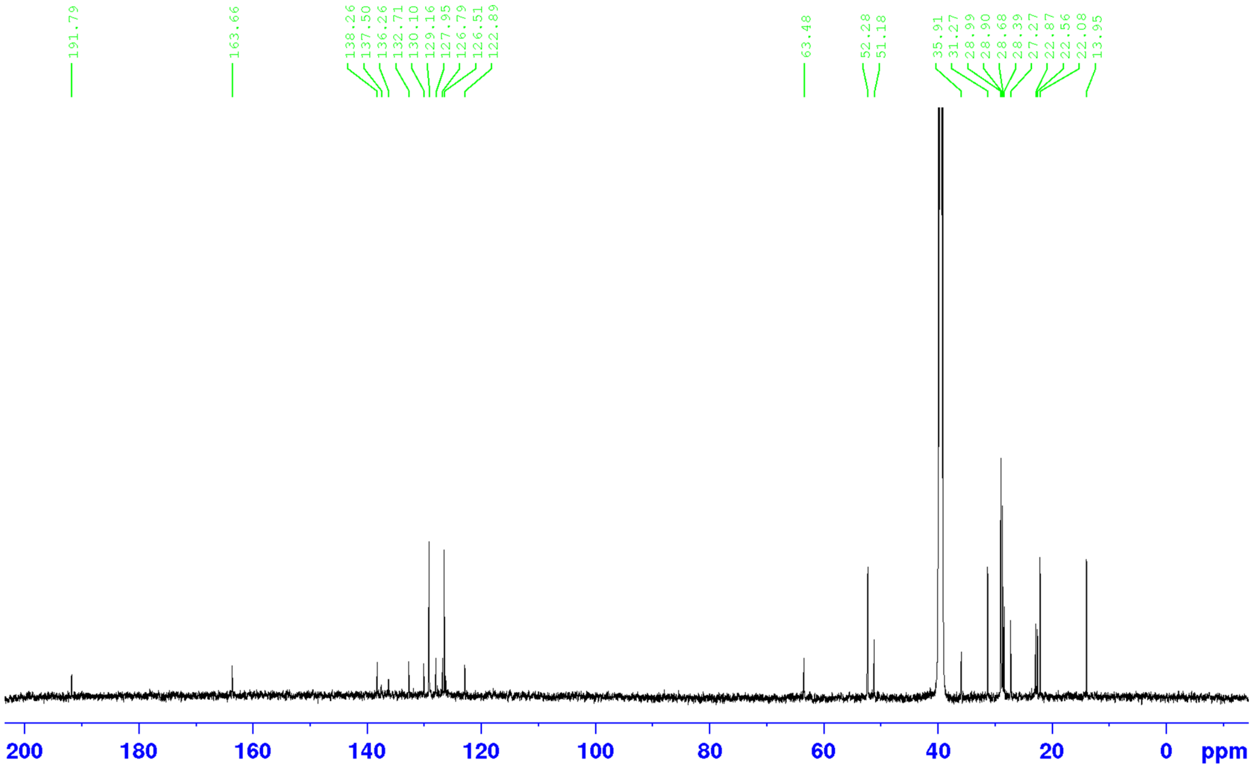

3-(2-(4-(Dodecylsulfonamido)-[1,1':4',1"-terphenyl]-3-yl)-2-oxoacetamido)-*N*,*N*,*N*-trimethylpropan-1-aminium iodide (**17d**)

^{1}H NMR (600 MHz, DMSO-d_6):

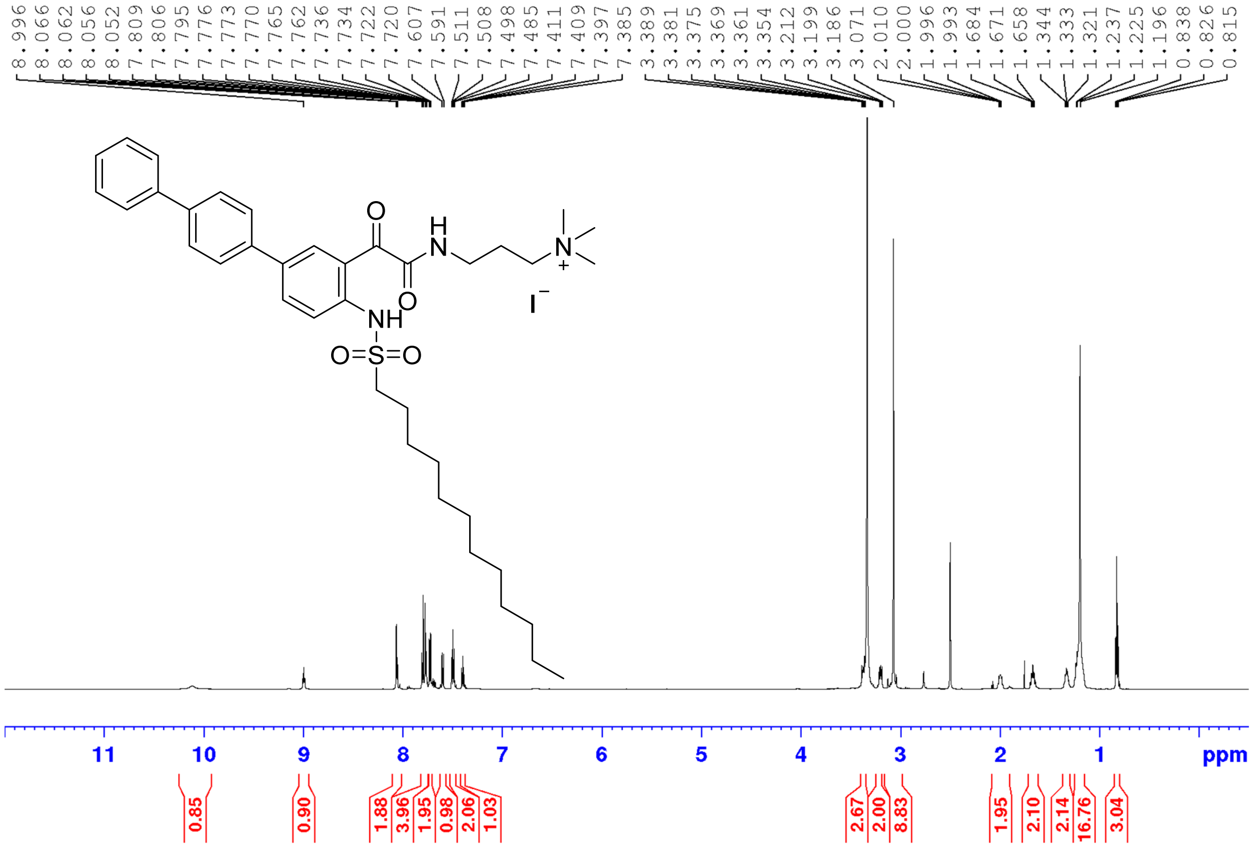

^{13}C NMR (150 MHz, DMSO-d_6):

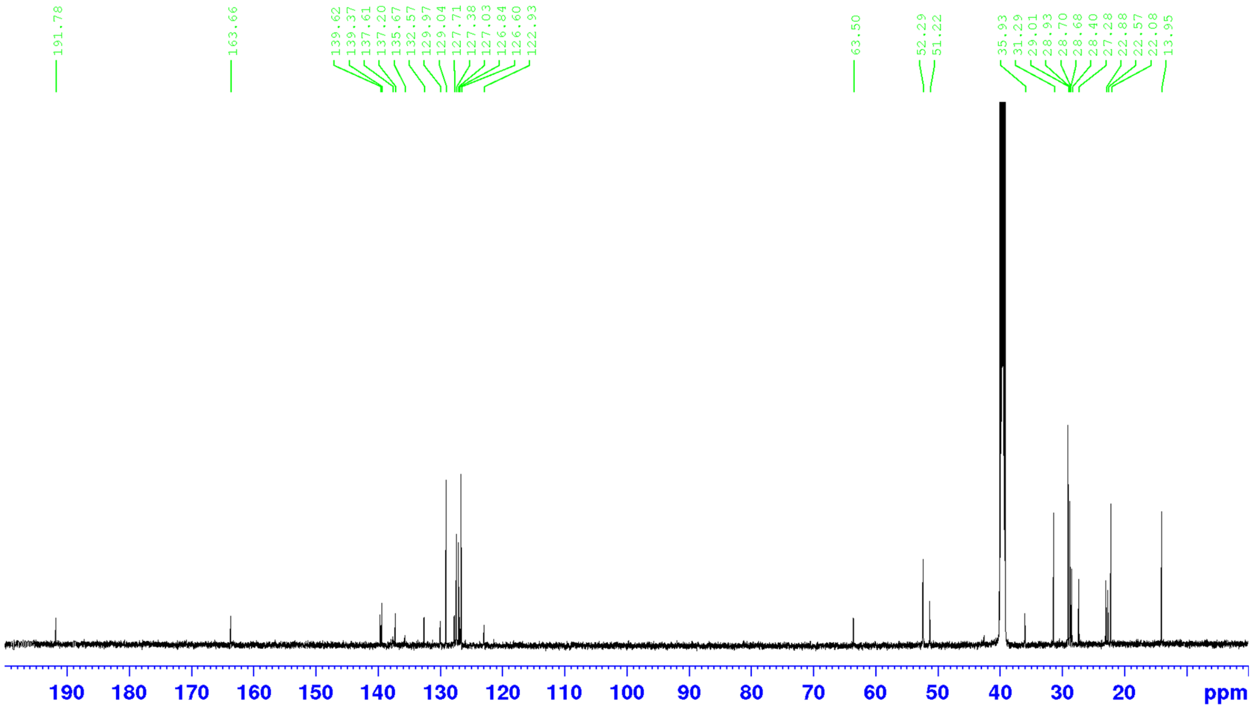

3-(2-(2-(Hexadecylsulfonamido)phenyl)-2-oxoacetamido)-*N*,*N*,*N*-trimethylpropan-1-aminium iodide (**18a**)

^{1}H NMR (400 MHz, DMSO-d_6):

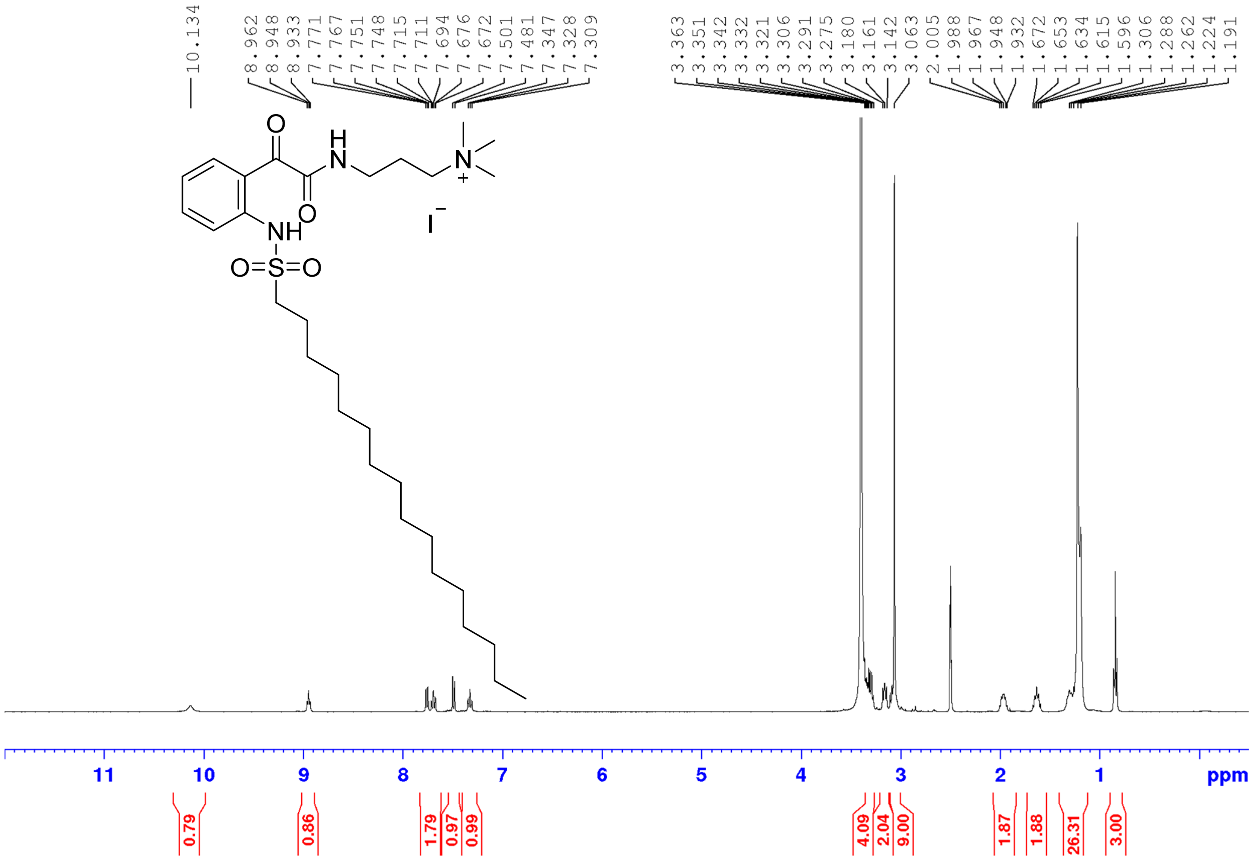

^{13}C NMR (150 MHz, DMSO-d_6):

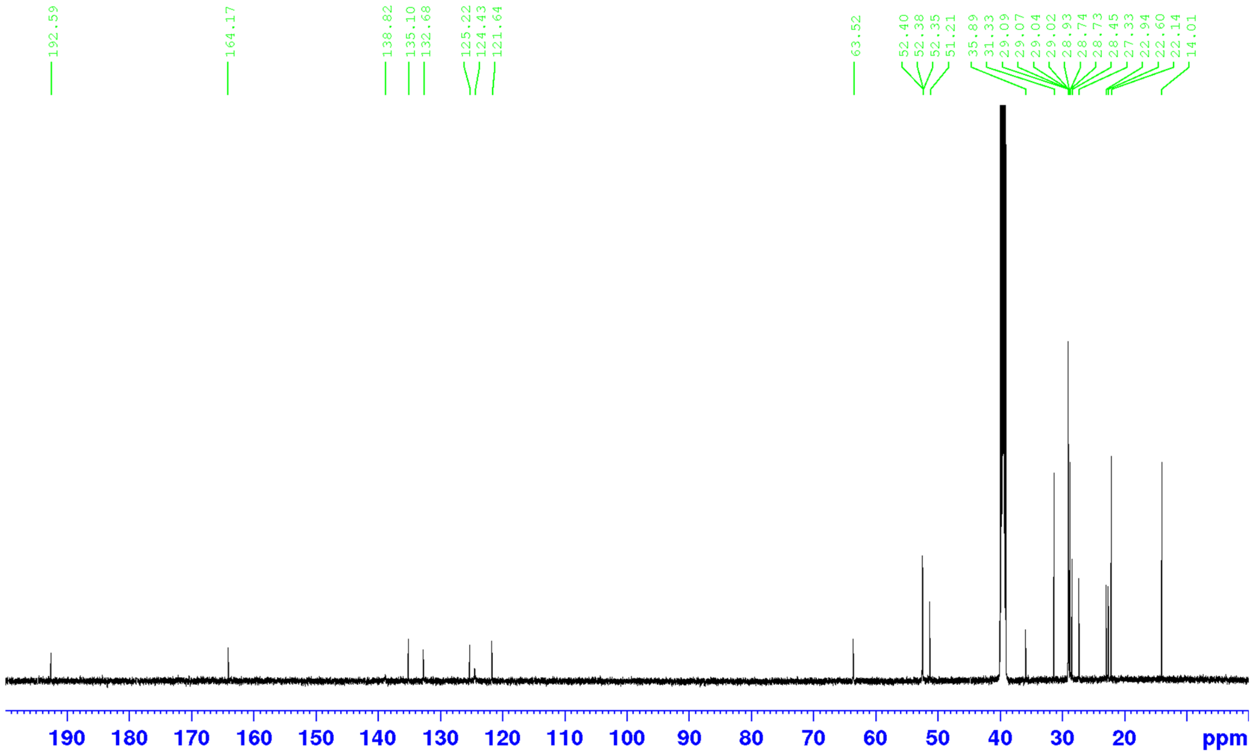

3-(2-(5-Bromo-2-(hexadecylsulfonamido)phenyl)-2-oxoacetamido)-*N*,*N*,*N*-trimethylpropan-1-aminium iodide (**18b**)

^{1}H NMR (600 MHz, DMSO-d_6):

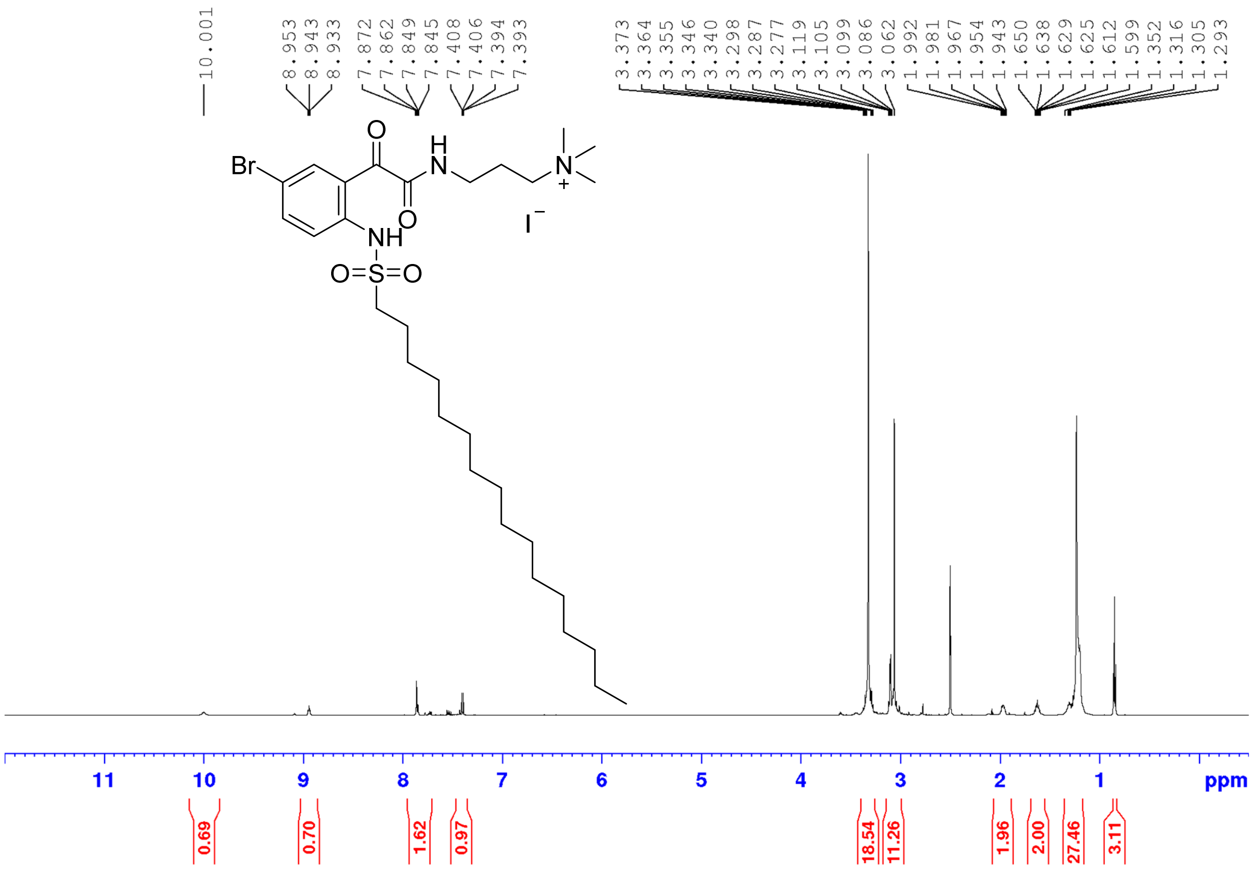

^{13}C NMR (150 MHz, DMSO-d_6):

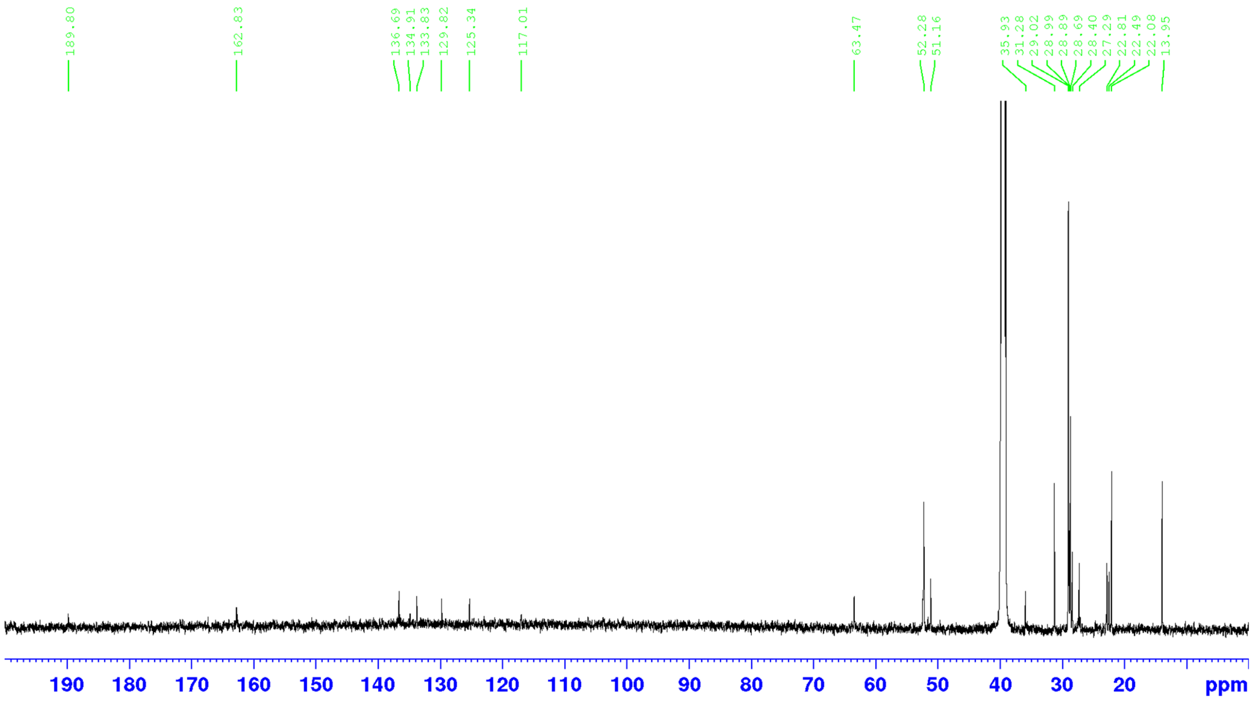

3-(2-(4-(Hexadecylsulfonamido)-[1,1'-biphenyl]-3-yl)-2-oxoacetamido)-*N*,*N*,*N*-trimethylpropan-1-aminium iodide (**18c**)

^{1}H NMR (600 MHz, DMSO-d_6):

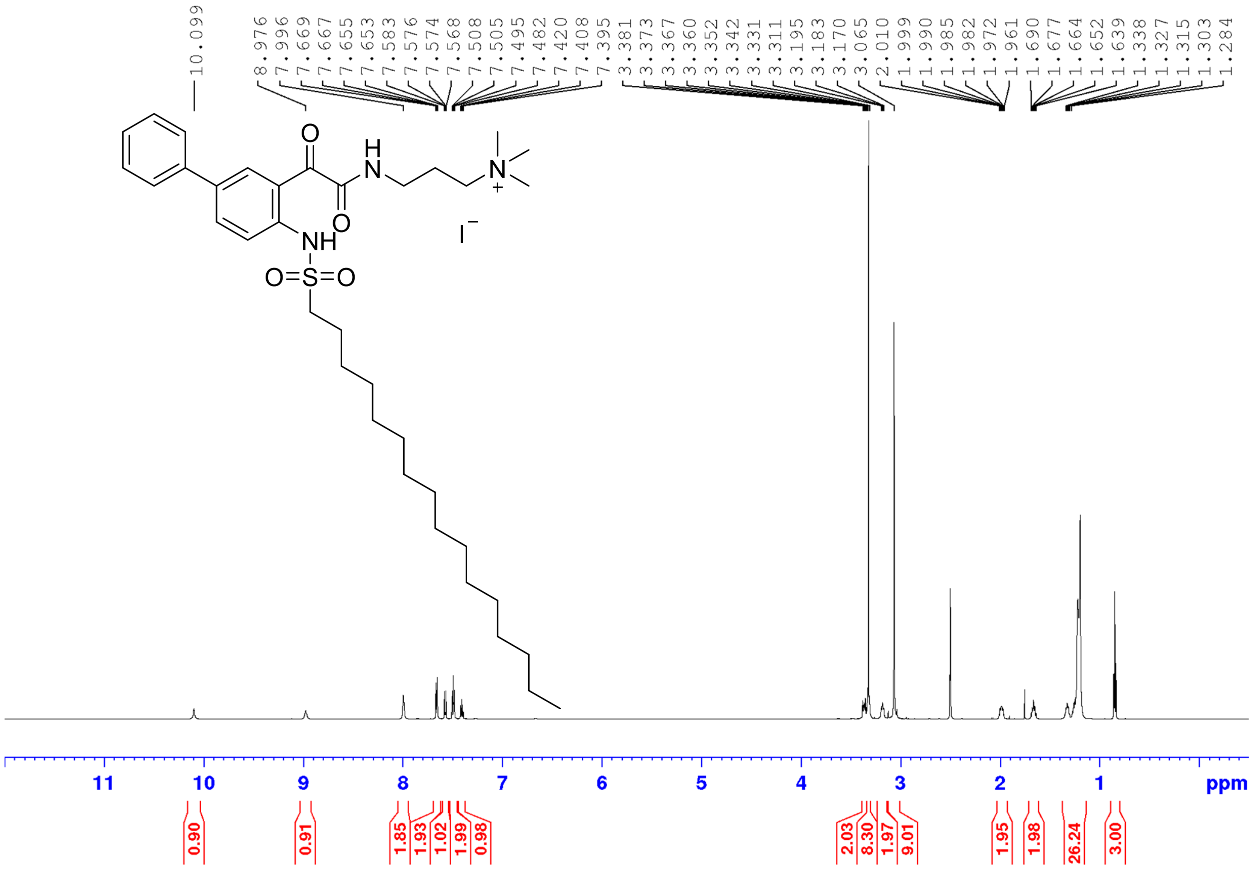

^{13}C NMR (150 MHz, DMSO-d_6):

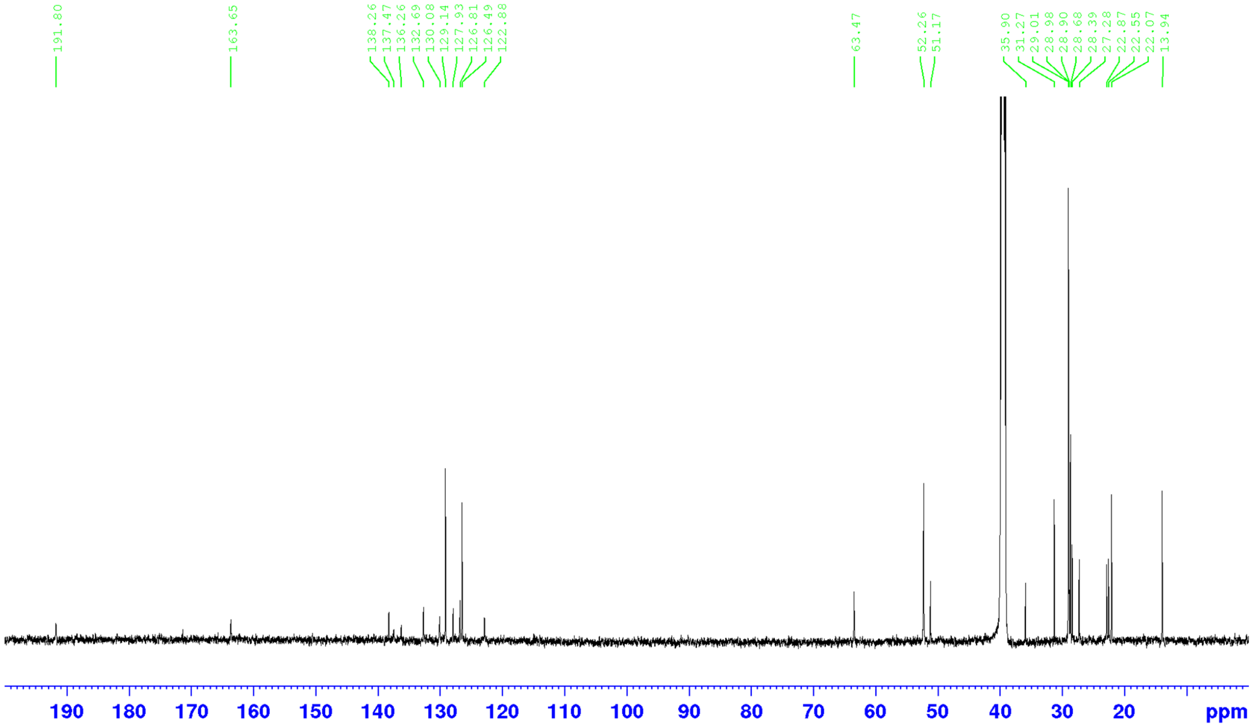

5-([1,1'-Biphenyl]-3-yl)indoline-2,3-dione (**19**)

^{1}H NMR (400 MHz, DMSO-d_6):

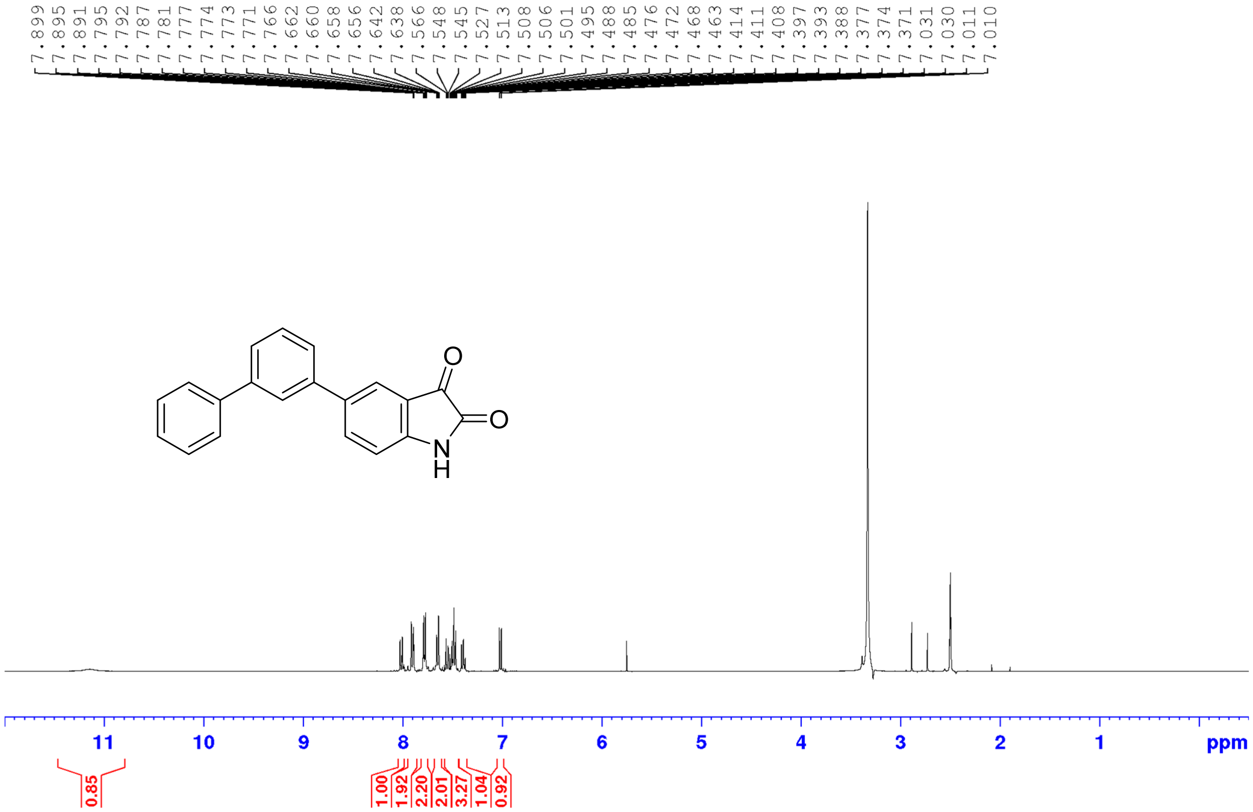

5-([1,1'-Biphenyl]-3-yl)-1-(octylsulfonyl)indoline-2,3-dione (**20**)

^{1}H NMR (400 MHz, DMSO-d_6):

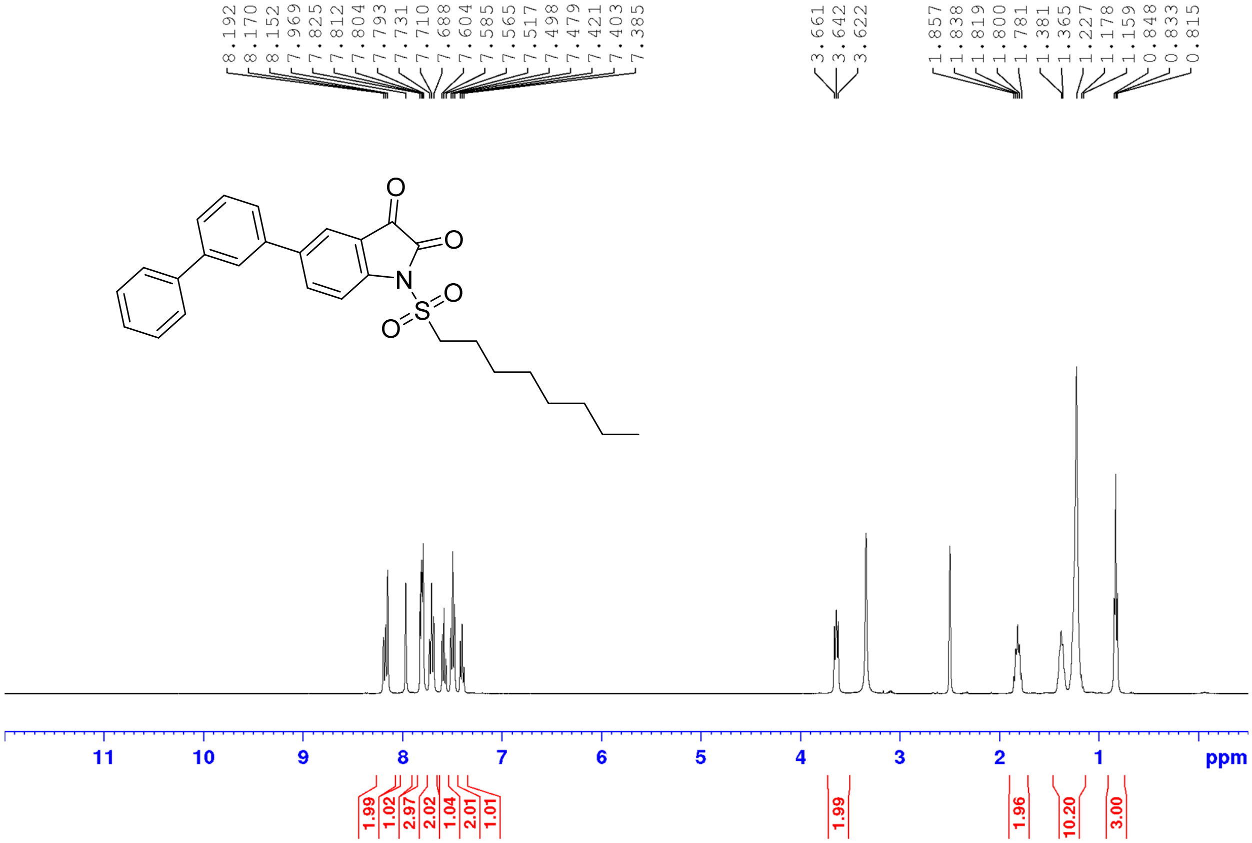

^{13}C NMR (100 MHz, DMSO-d_6):

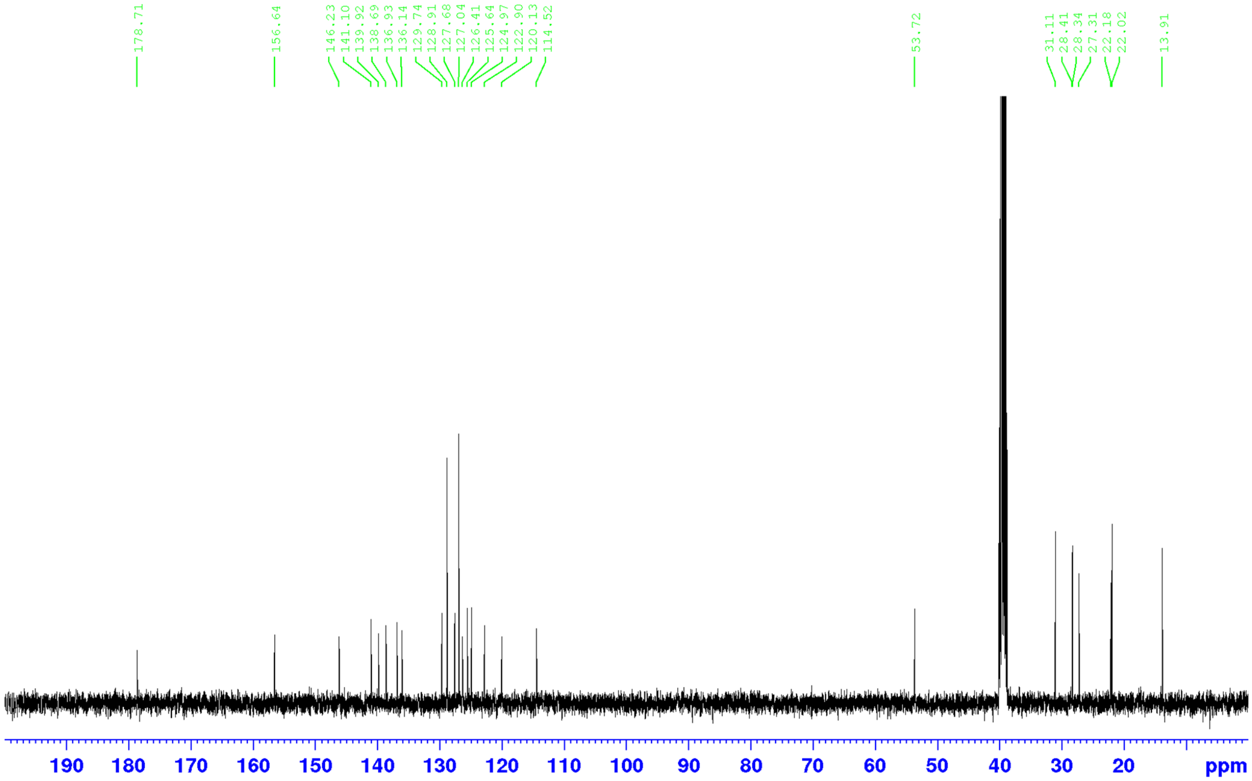

N-(3-(Dimethylamino)propyl)-2-(4-(octylsulfonamido)-[1,1':3',1''-terphenyl]-3-yl)-2-oxoacetamide (**21**)

^{1}H NMR (400 MHz, $CDCl_3$):

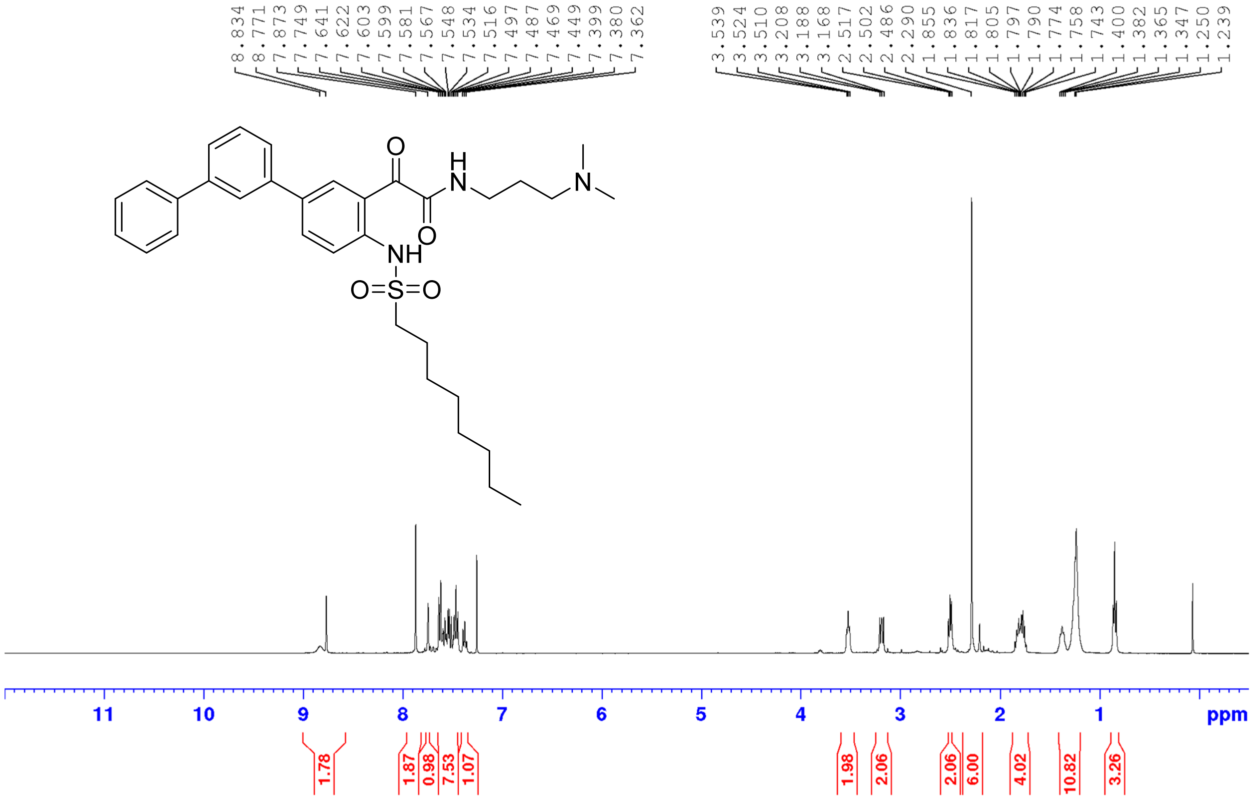

^{13}C NMR (100 MHz, $CDCl_3$):

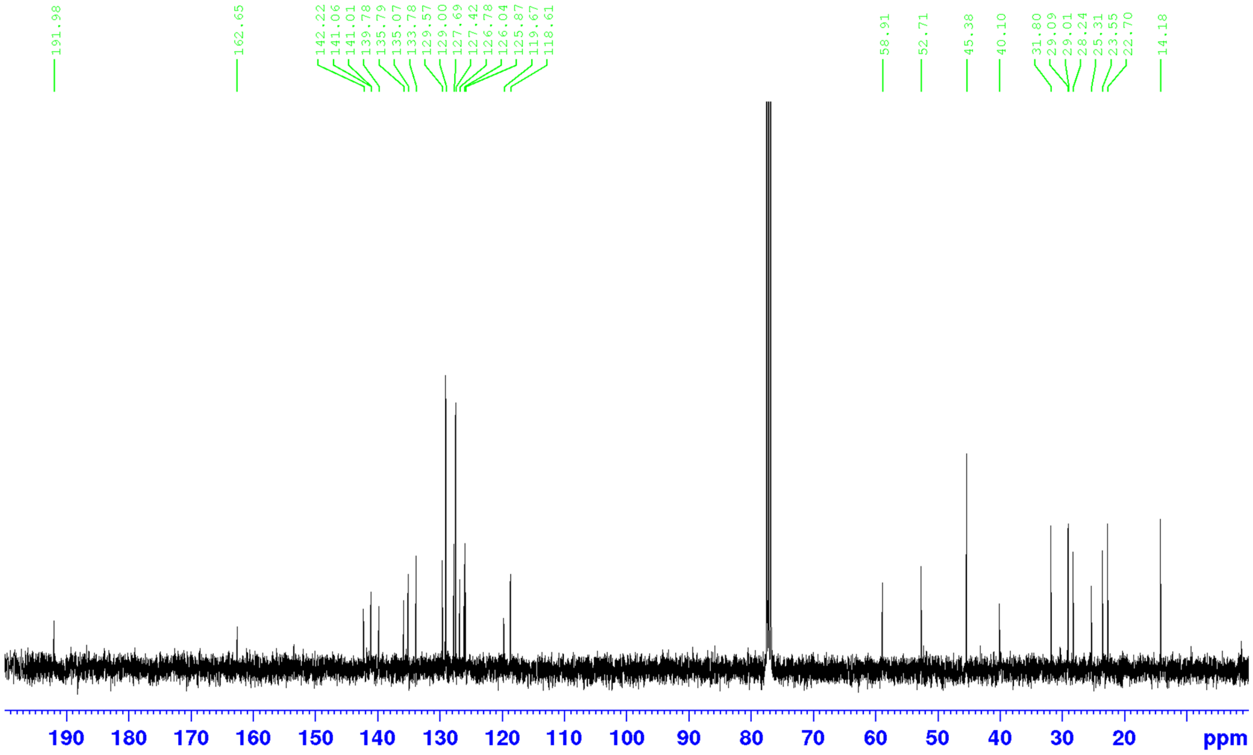

N,*N*-Dimethyl-3-(2-(4-(octylsulfonamido)-[1,1':3',1''-terphenyl]-3-yl)-2-oxoacetamido)propan-1-aminium chloride (**22**)

^{1}H NMR (600 MHz, DMSO-d_6):

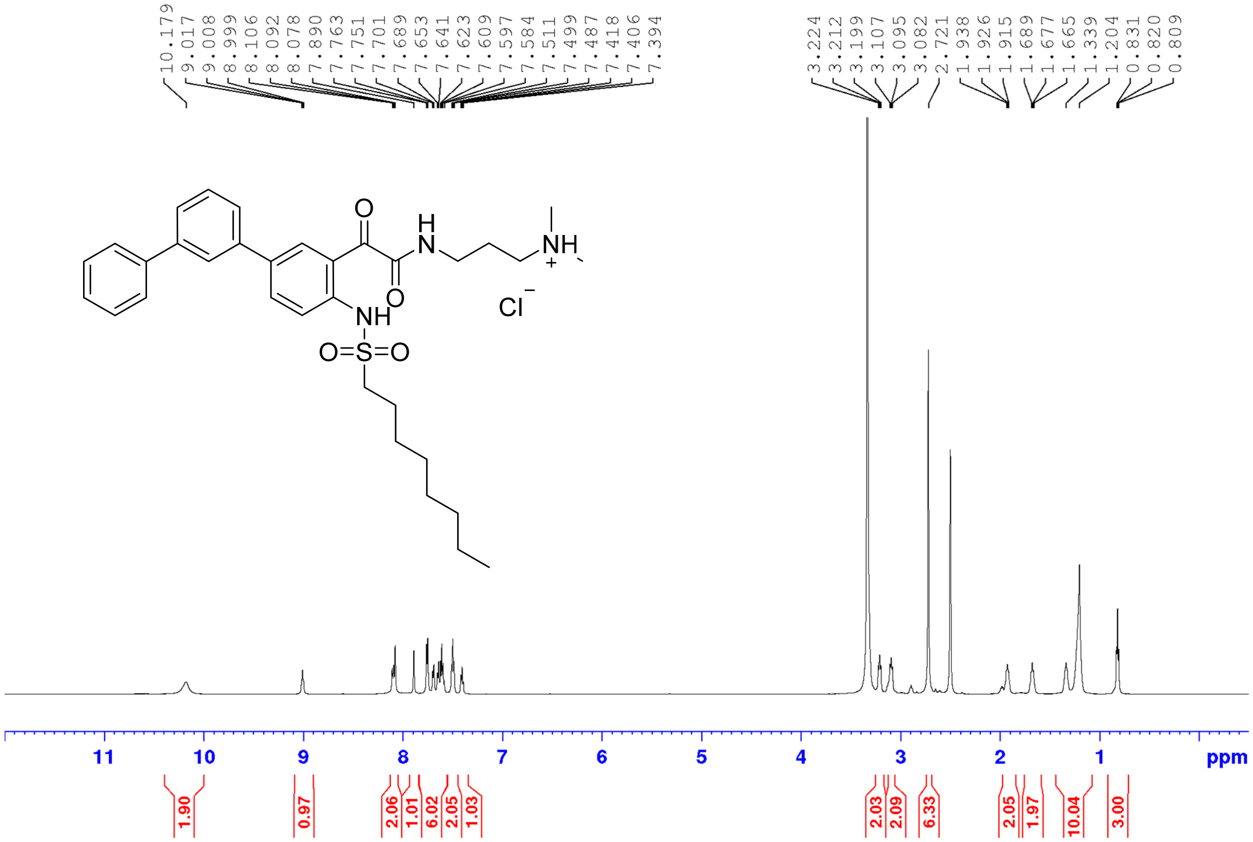

^{13}C NMR (150 MHz, DMSO-d_6):

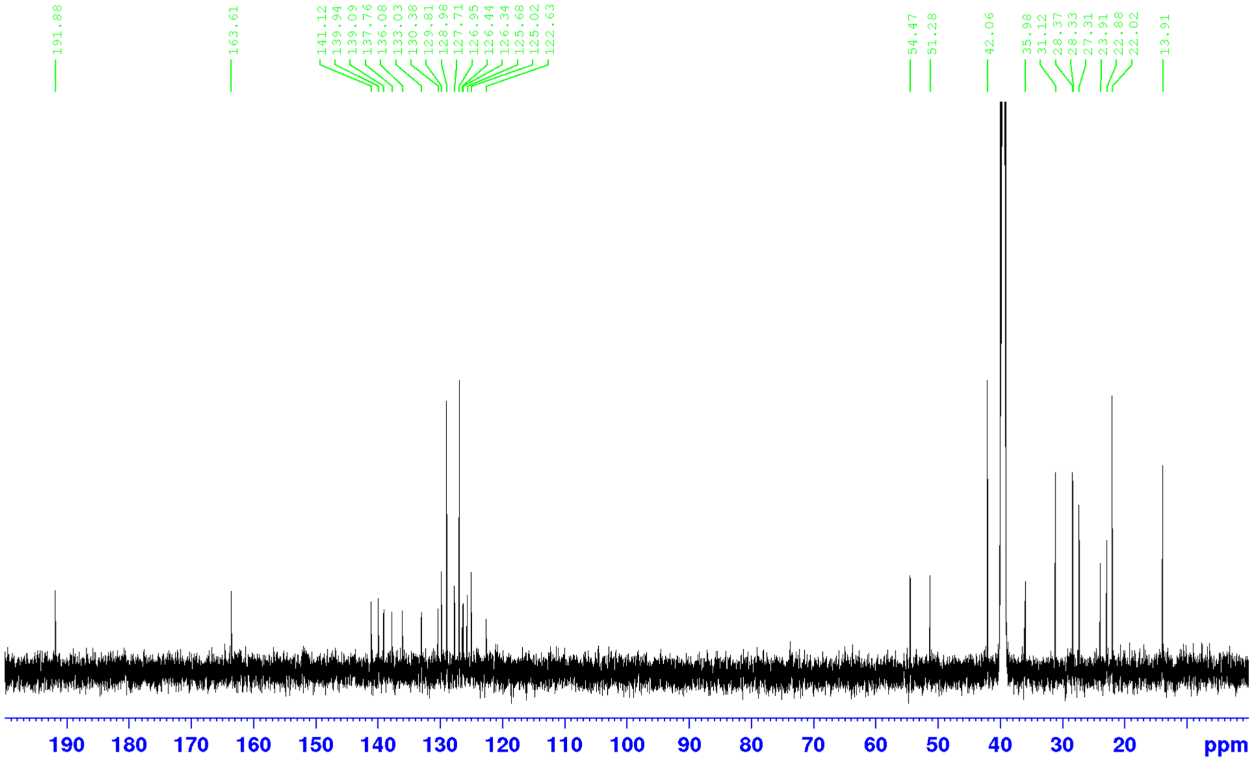

N,*N*,*N*-Trimethyl-3-(2-(4-(octylsulfonamido)-[1,1':3',1"-terphenyl]-3-yl)-2-oxoacetamido)propan-1-aminium iodide (**23**)

^{1}H NMR (600 MHz, DMSO-d_6):

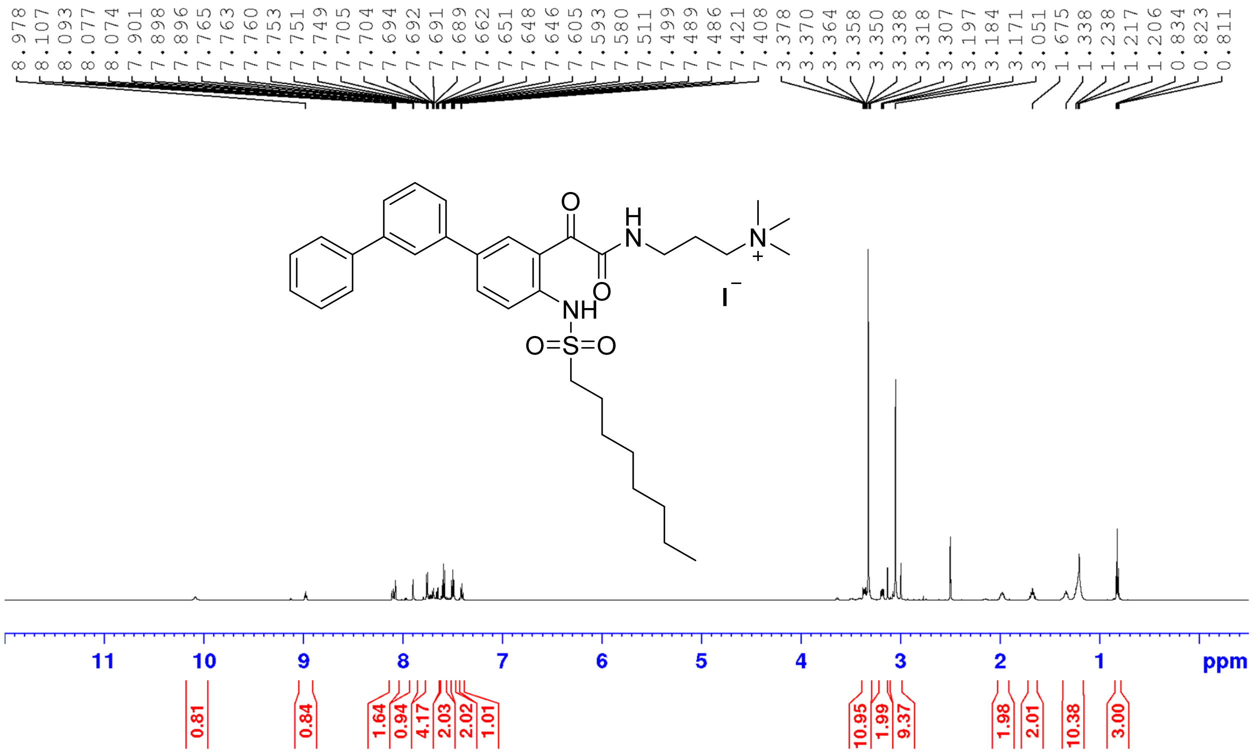

^{13}C NMR (150 MHz, DMSO-d_6):

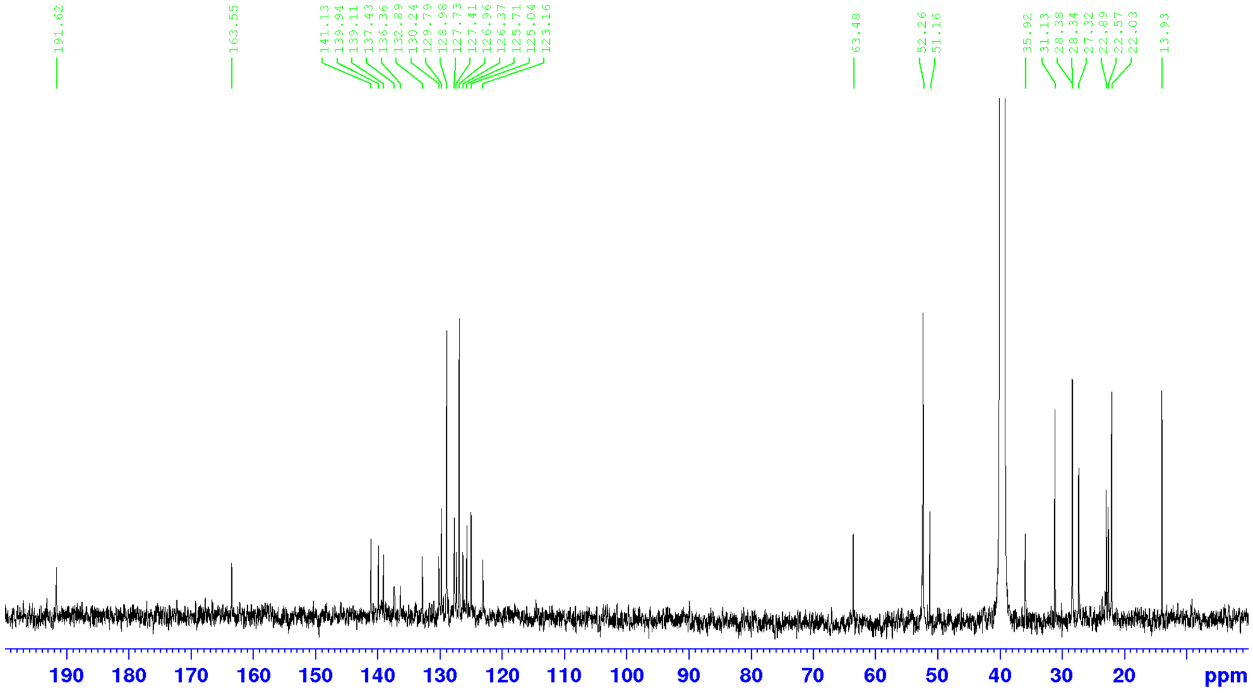

tert-Butyl (3-(2-(4-(octylsulfonamido)-[1,1':4',1"-terphenyl]-3-yl)-2-oxoacetamido)propyl)carbamate (**24d**)

^{1}H NMR (400 MHz, CDCl$_3$):

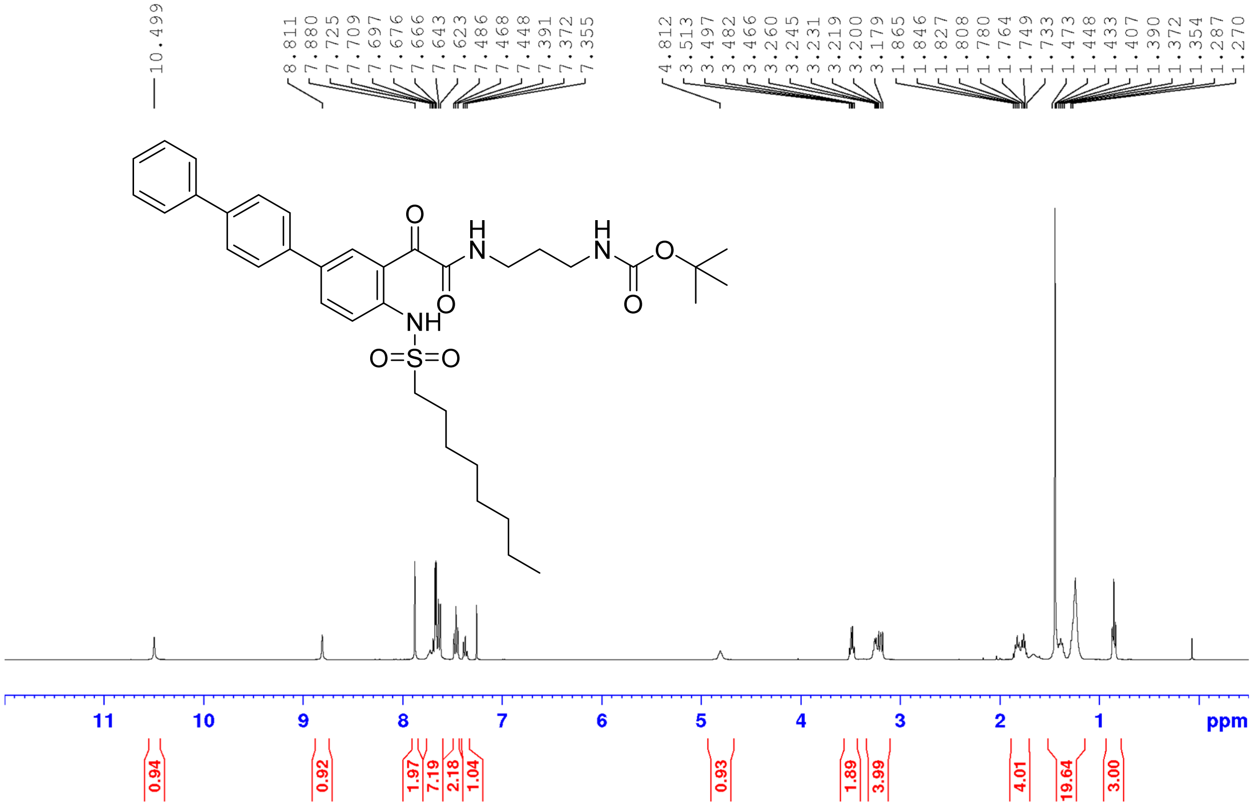

^{13}C NMR (100 MHz, CDCl$_3$):

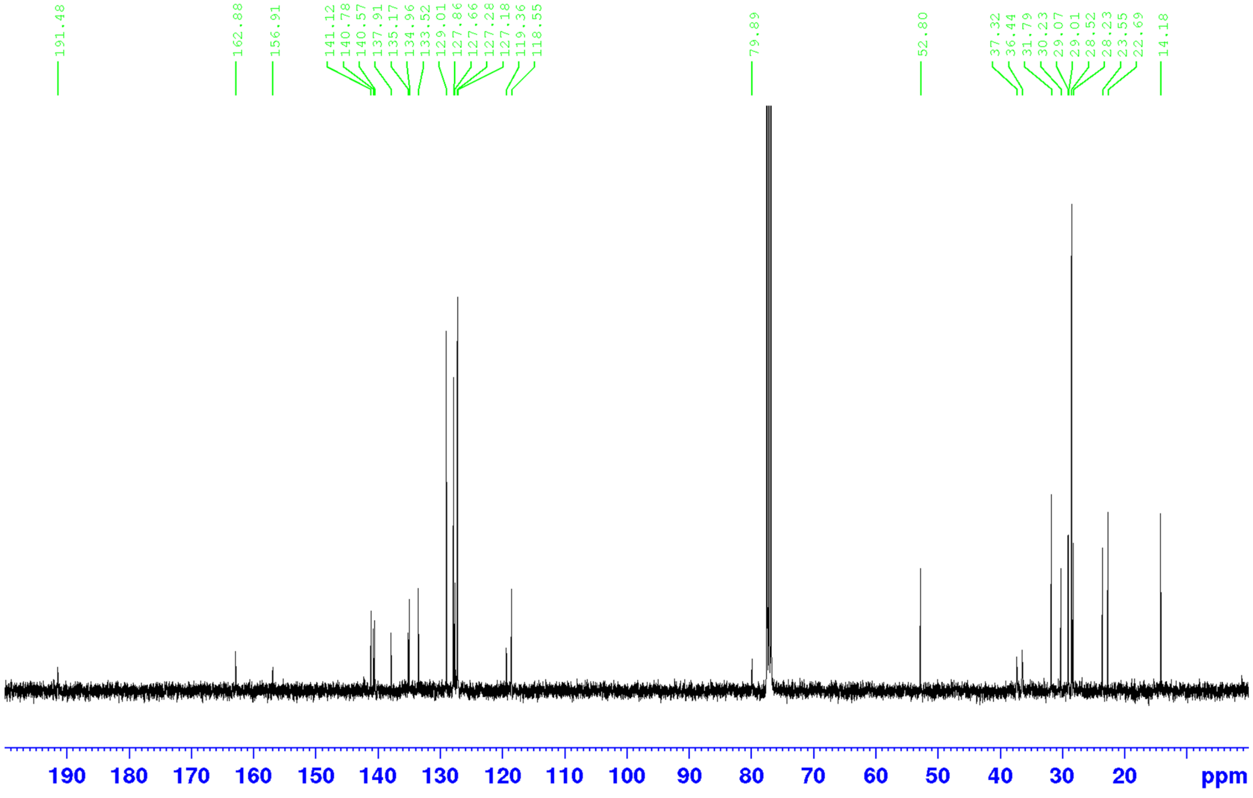

tert-Butyl (3-(2-(2-(dodecylsulfonamido)phenyl)-2-oxoacetamido)propyl)carbamate (**25a**)

^{1}H NMR (400 MHz, $CDCl_3$):

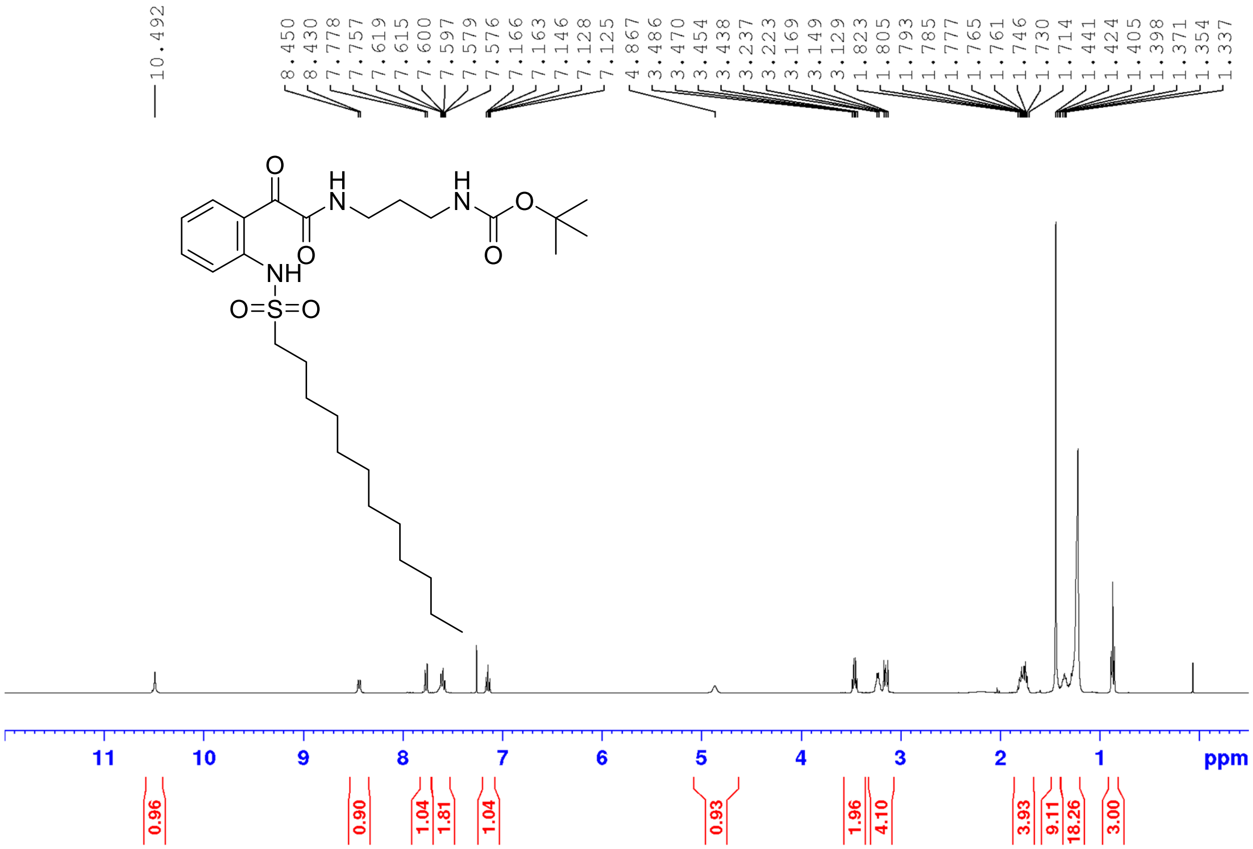

^{13}C NMR (100 MHz, $CDCl_3$):

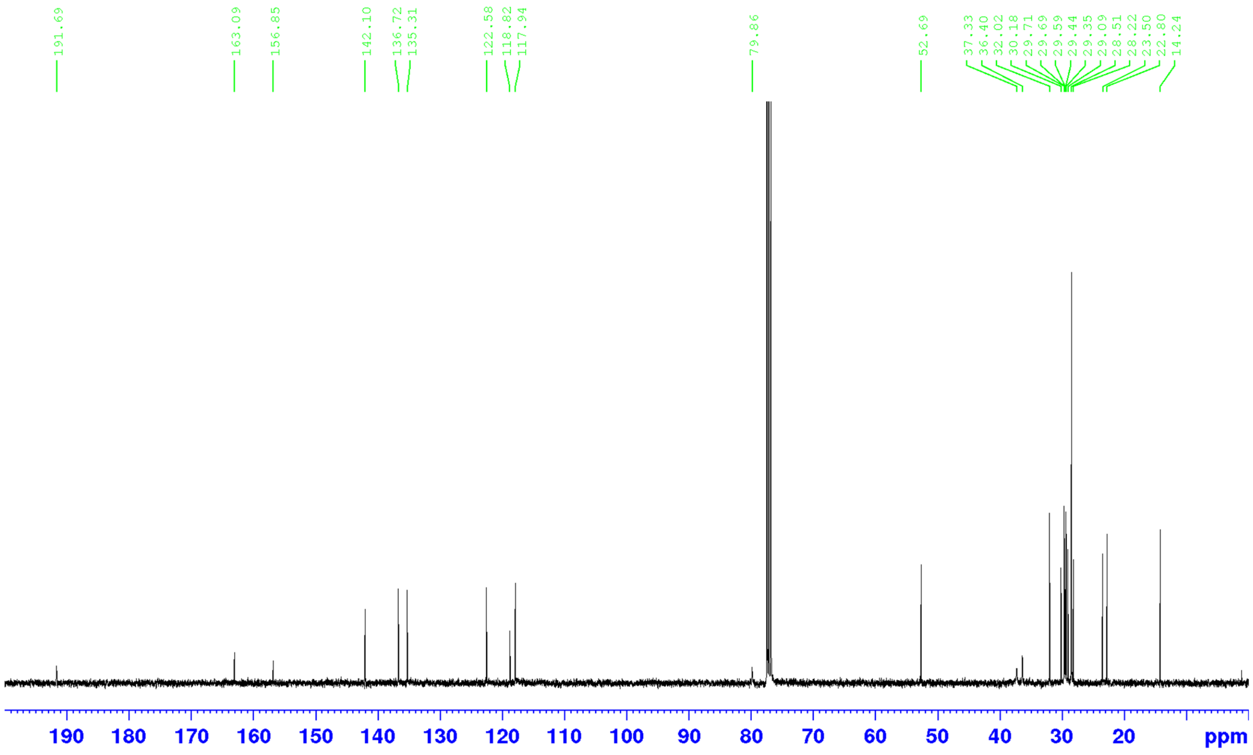

tert-Butyl (3-(2-(5-bromo-2-(dodecylsulfonamido)phenyl)-2-oxoacetamido)propyl)carbamate (**25b**)

^{1}H NMR (400 MHz, $CDCl_3$):

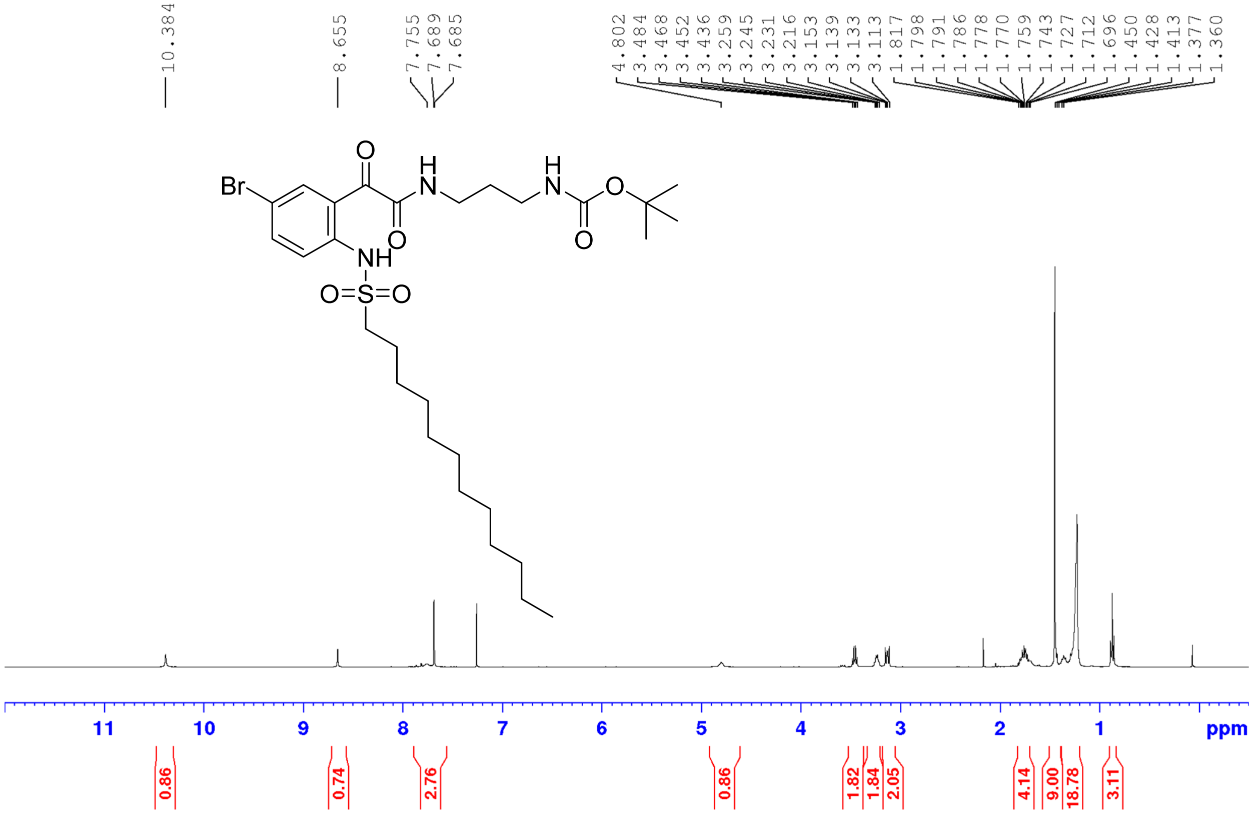

^{13}C NMR (100 MHz, $CDCl_3$):

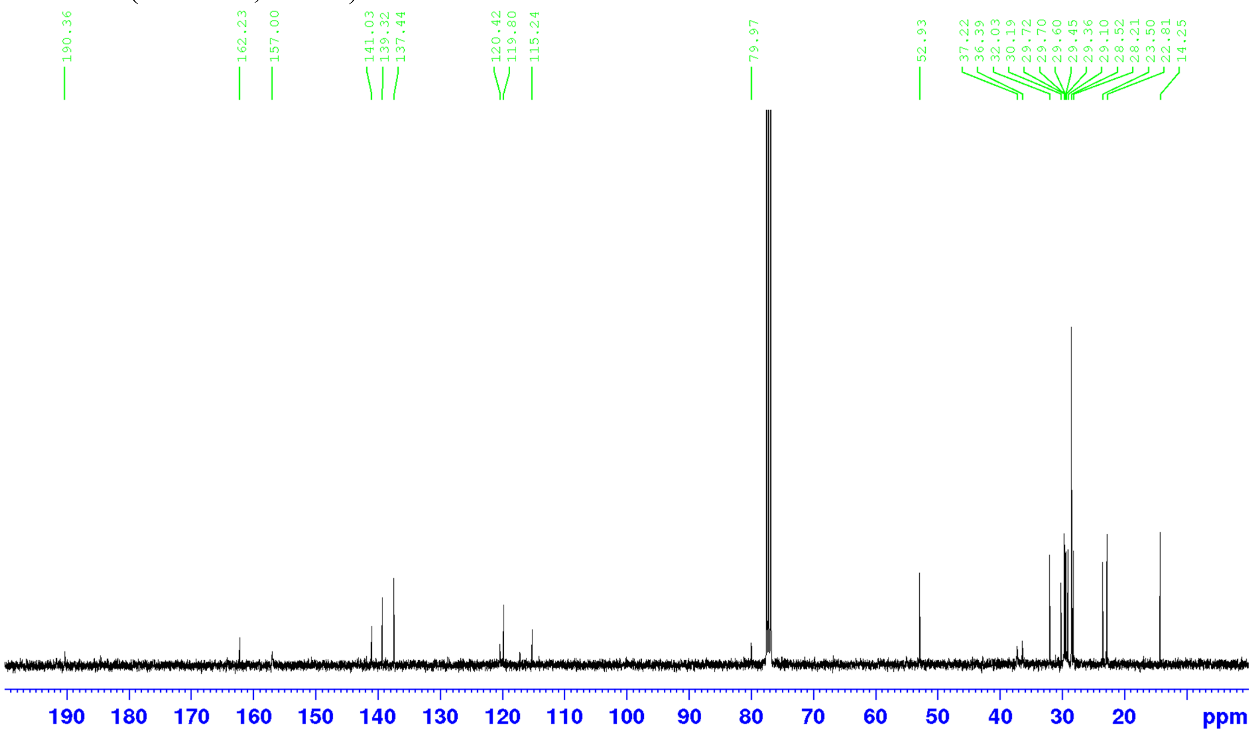

tert-Butyl (3-(2-(4-(dodecylsulfonamido)-[1,1'-biphenyl]-3-yl)-2-oxoacetamido)propyl)carbamate (**25c**)

^{1}H NMR (400 MHz, $CDCl_3$):

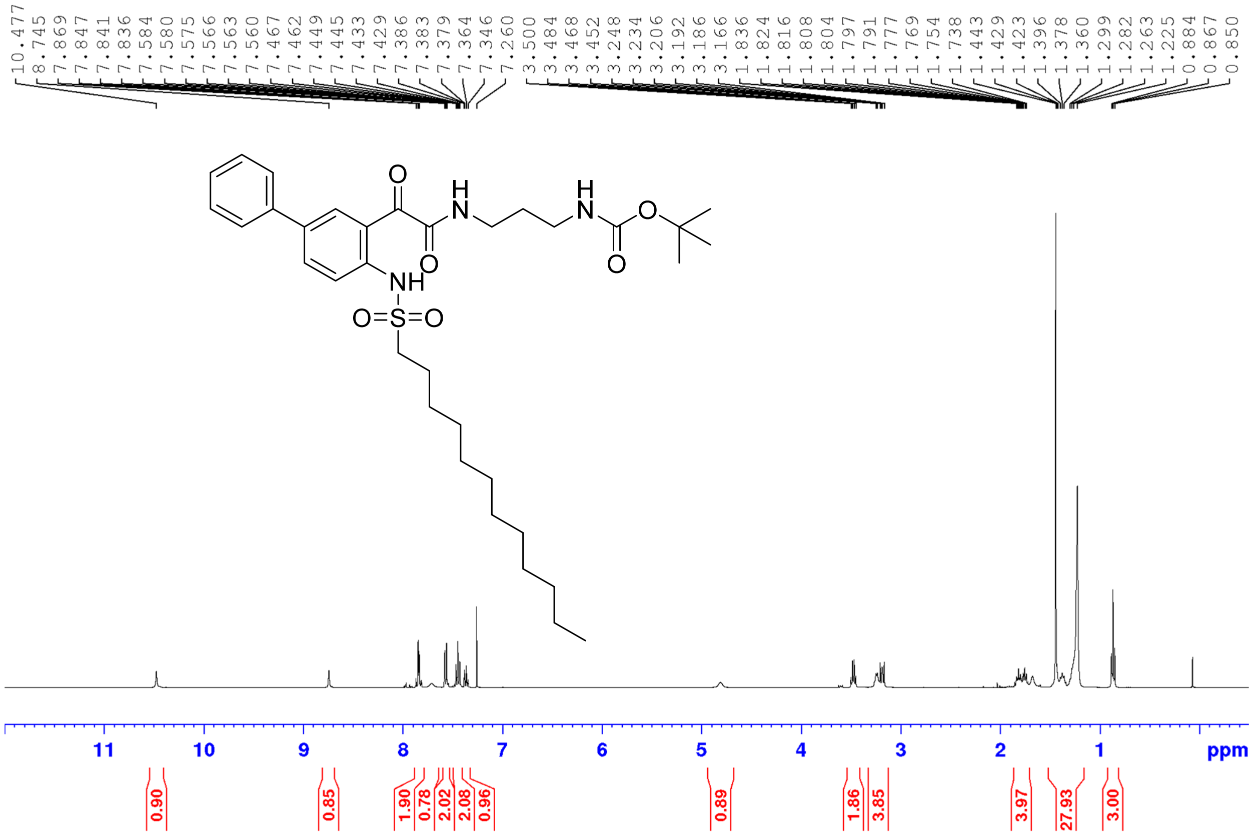

^{13}C NMR (100 MHz, $CDCl_3$):

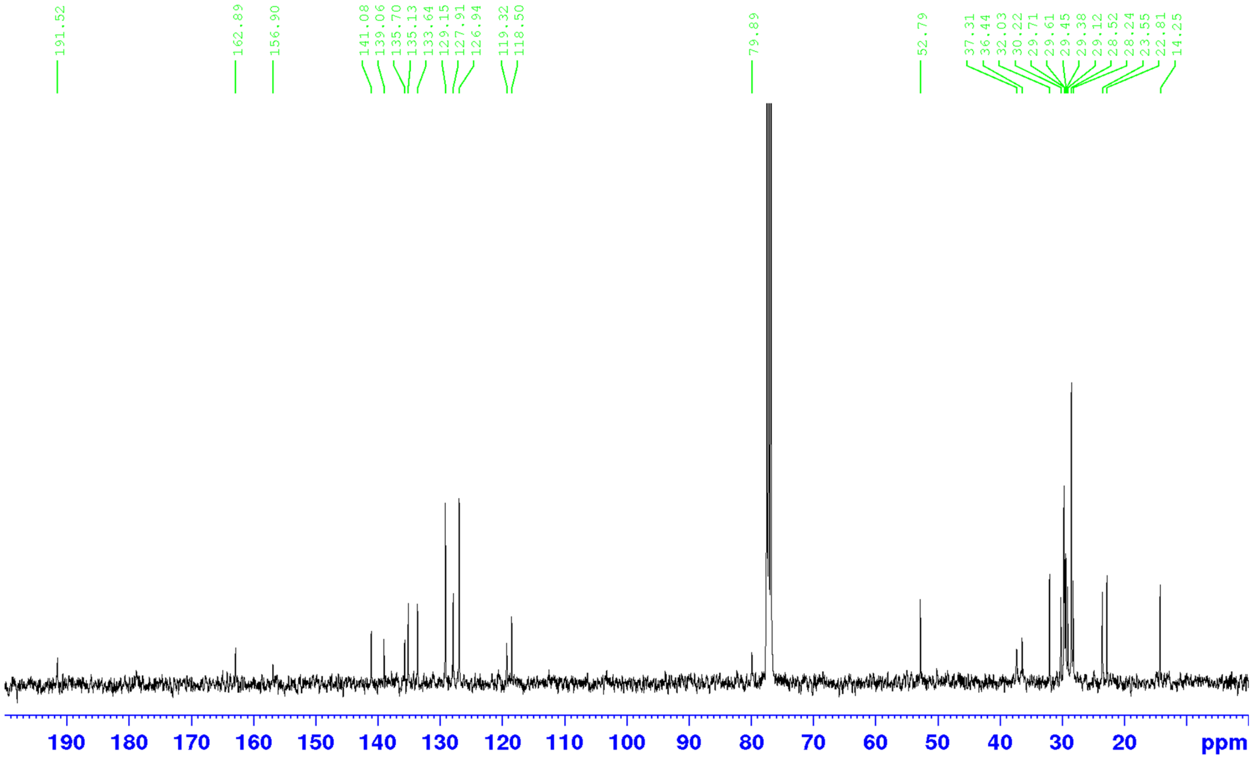

tert-Butyl (3-(2-(2-(hexadecylsulfonamido)phenyl)-2-oxoacetamido)propyl)carbamate (**26a**)

^{1}H NMR (400 MHz, $CDCl_3$):

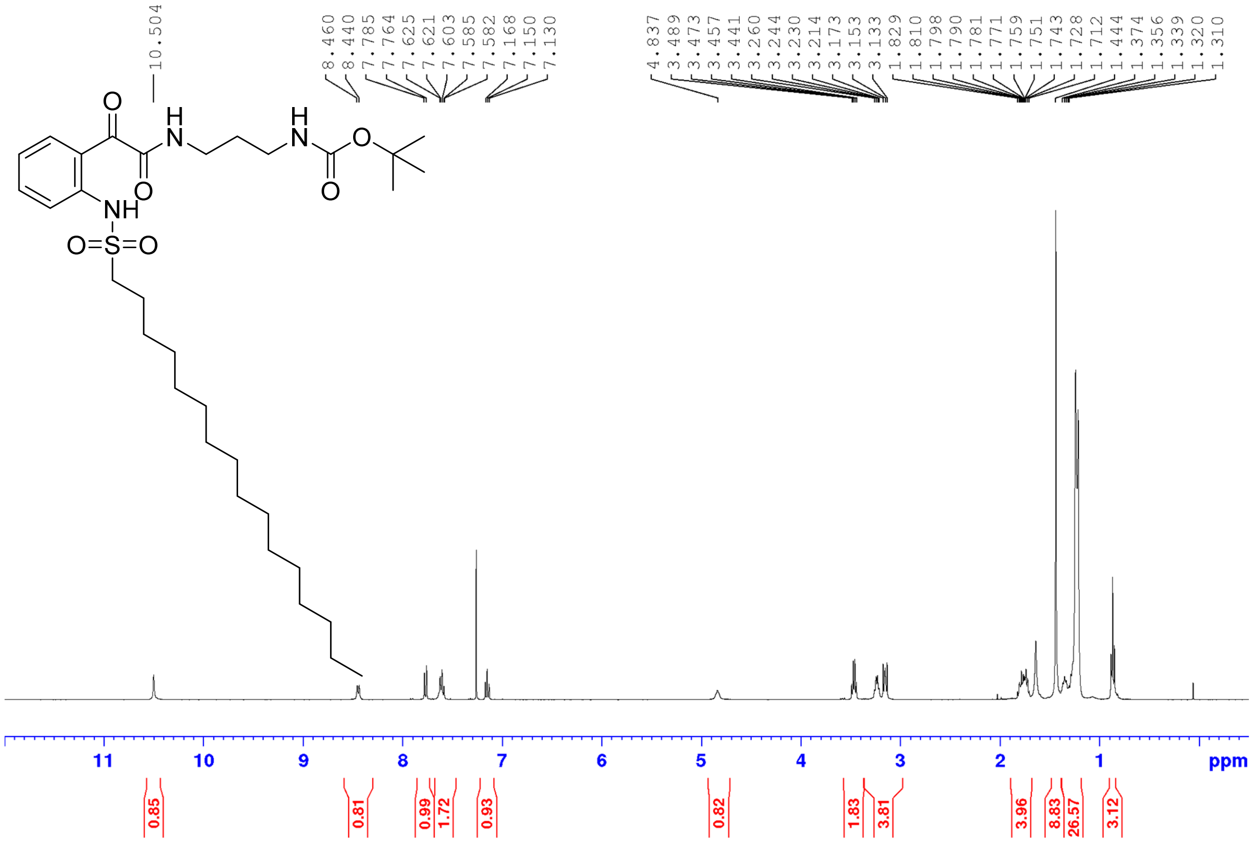

^{13}C NMR (150 MHz, $CDCl_3$):

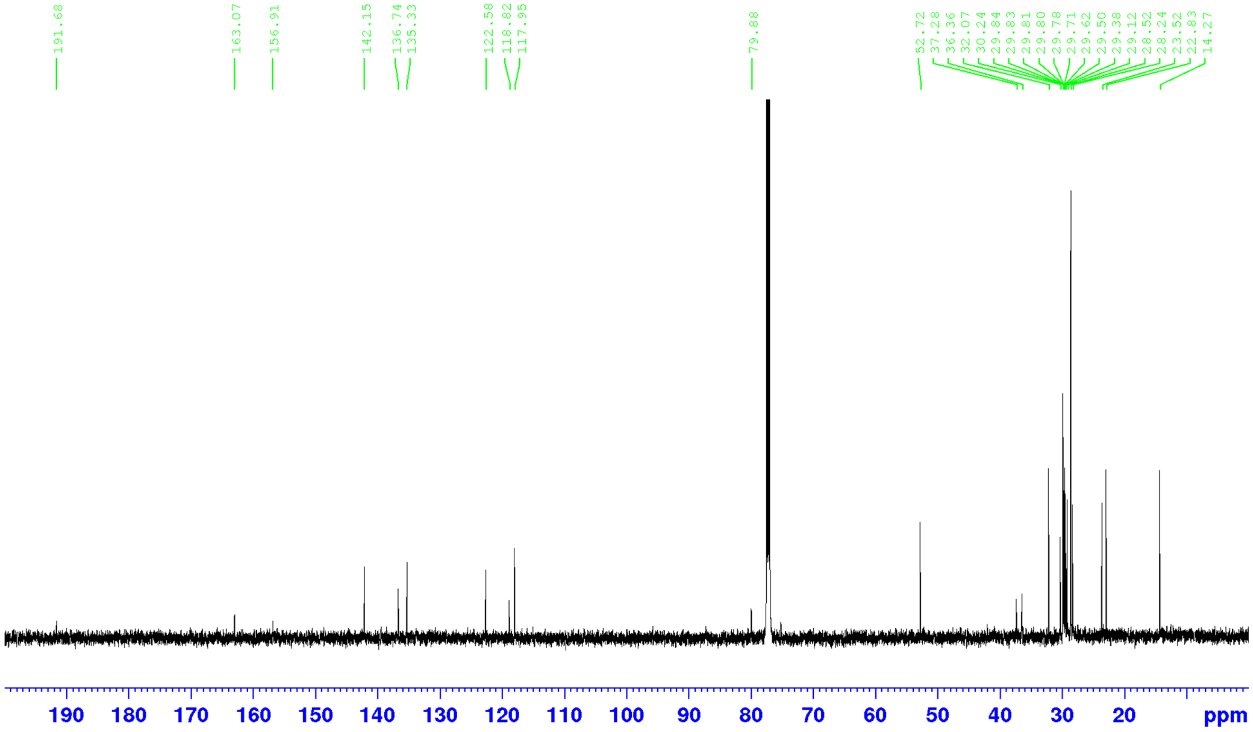

tert-Butyl (3-(2-(5-bromo-2-(hexadecylsulfonamido)phenyl)-2-oxoacetamido)propyl)carbamate (**26b**)

^{1}H NMR (400 MHz, $CDCl_3$):

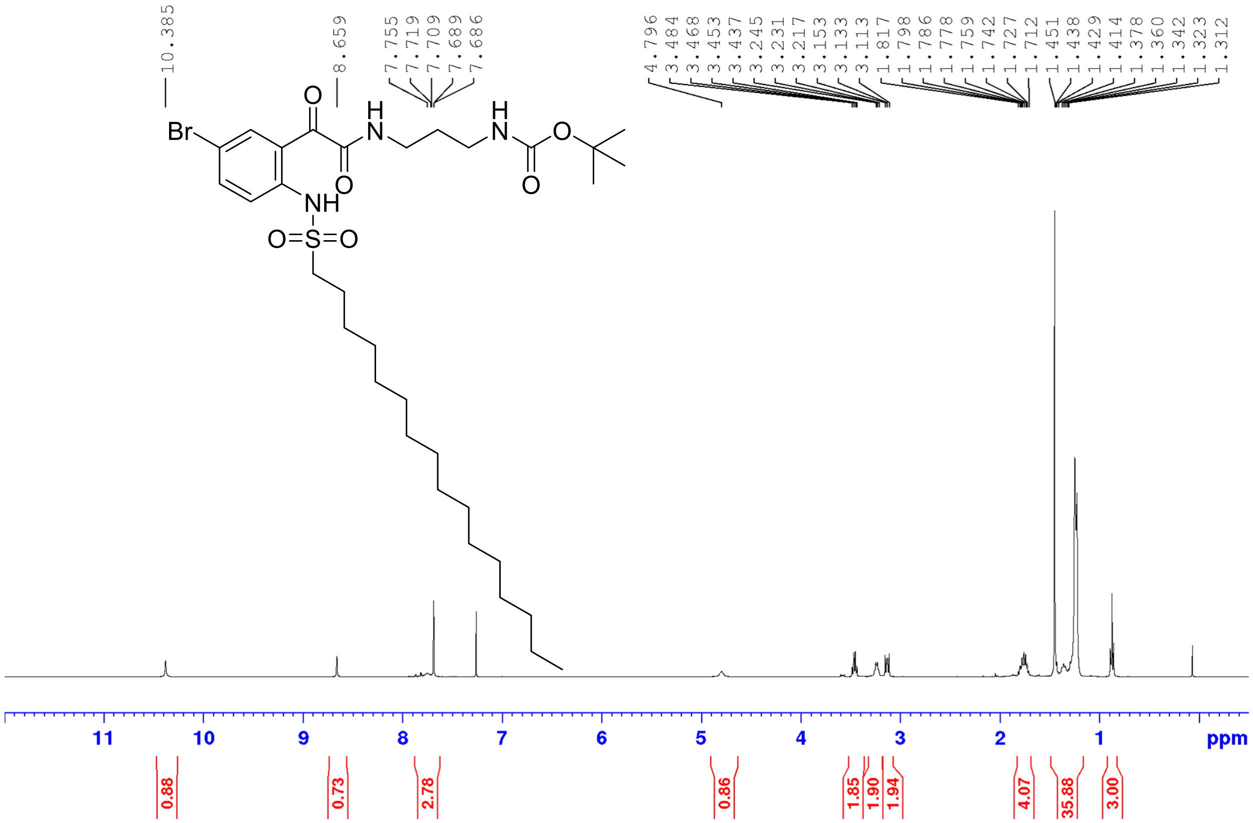

^{13}C NMR (100 MHz, $CDCl_3$):

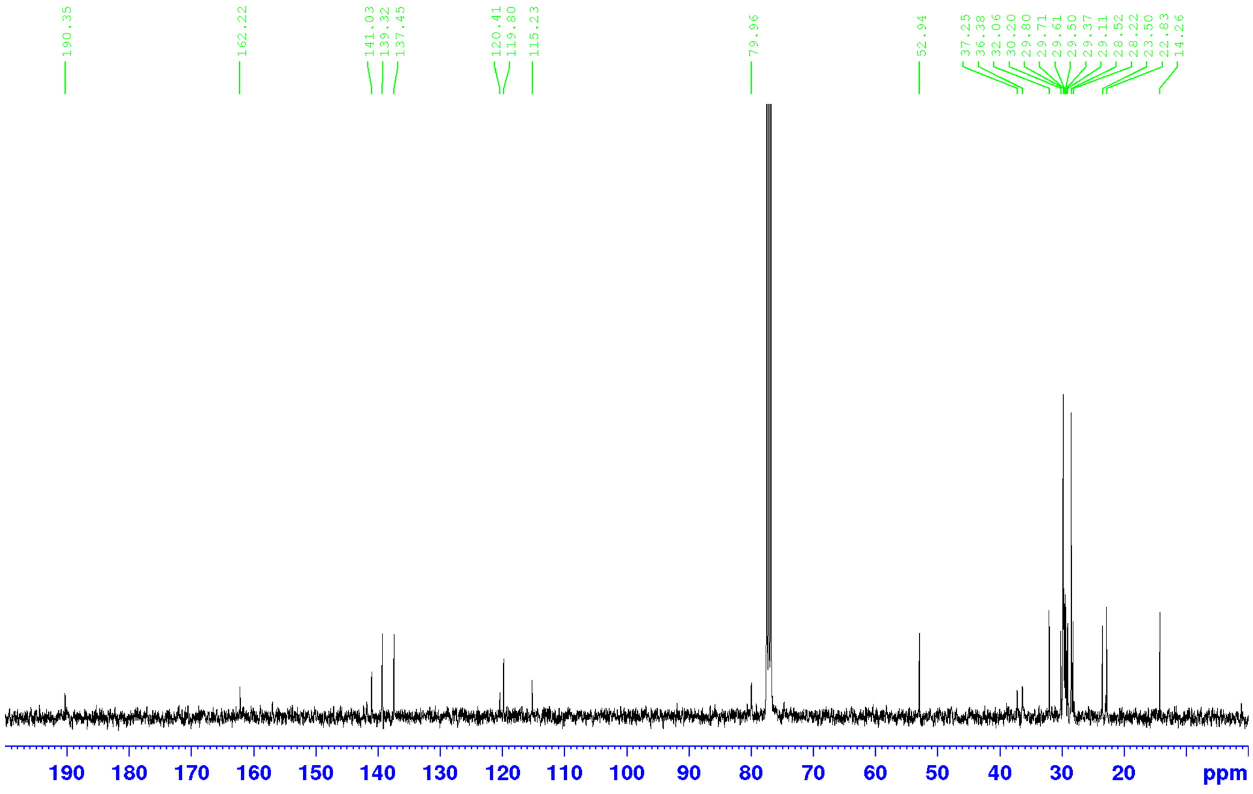

tert-Butyl (3-(2-(4-(hexadecylsulfonamido)-[1,1'-biphenyl]-3-yl)-2-oxoacetamido)propyl)carbamate (**26c**)

^{1}H NMR (400 MHz, $CDCl_3$):

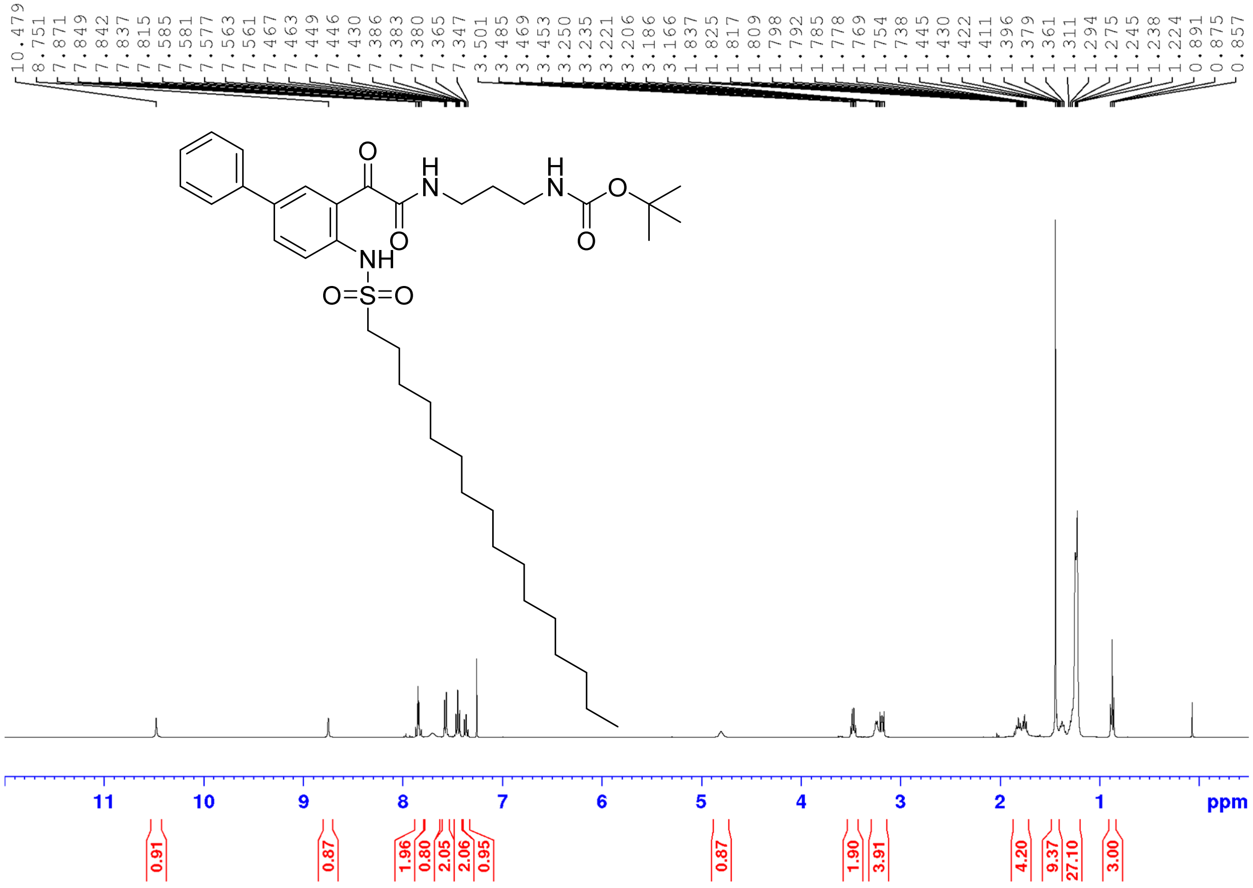

^{13}C NMR (100 MHz, $CDCl_3$):

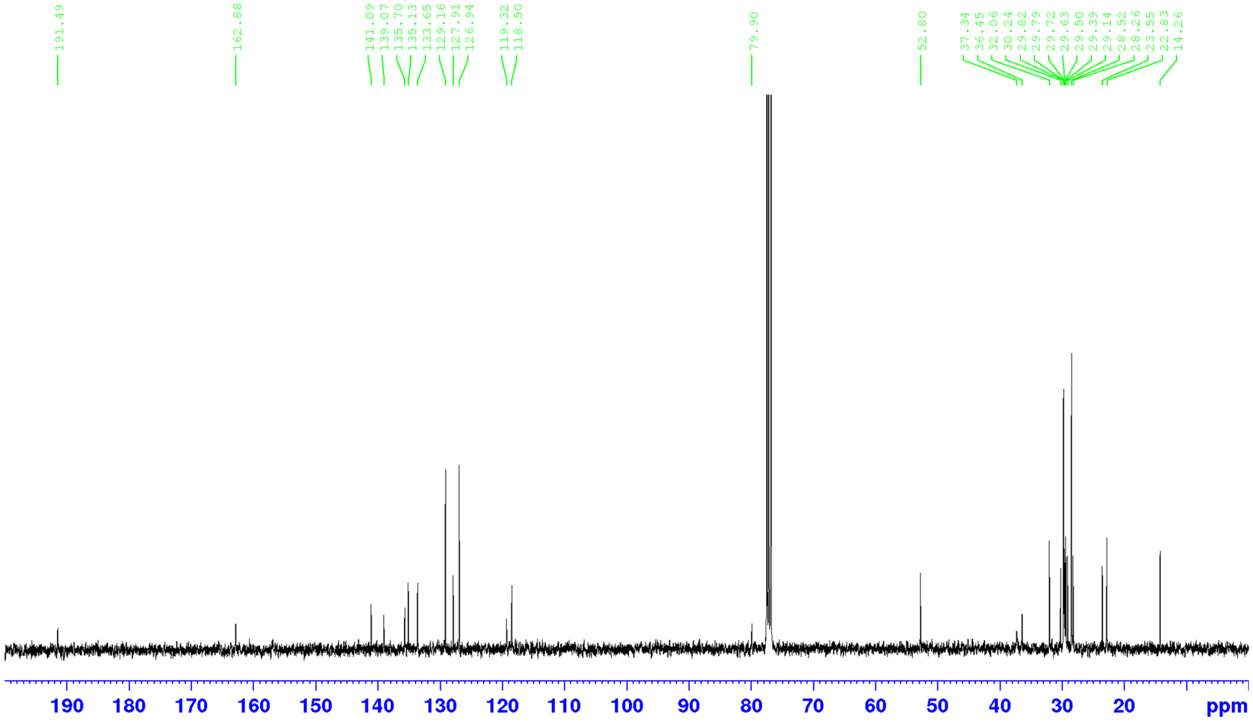

N-(3-aminopropyl)-2-(4-(octylsulfonamido)-[1,1':4',1''-terphenyl]-3-yl)-2-oxoacetamide hydrochloride (**27d**)

^{1}H NMR (400 MHz, DMSO-d_6):

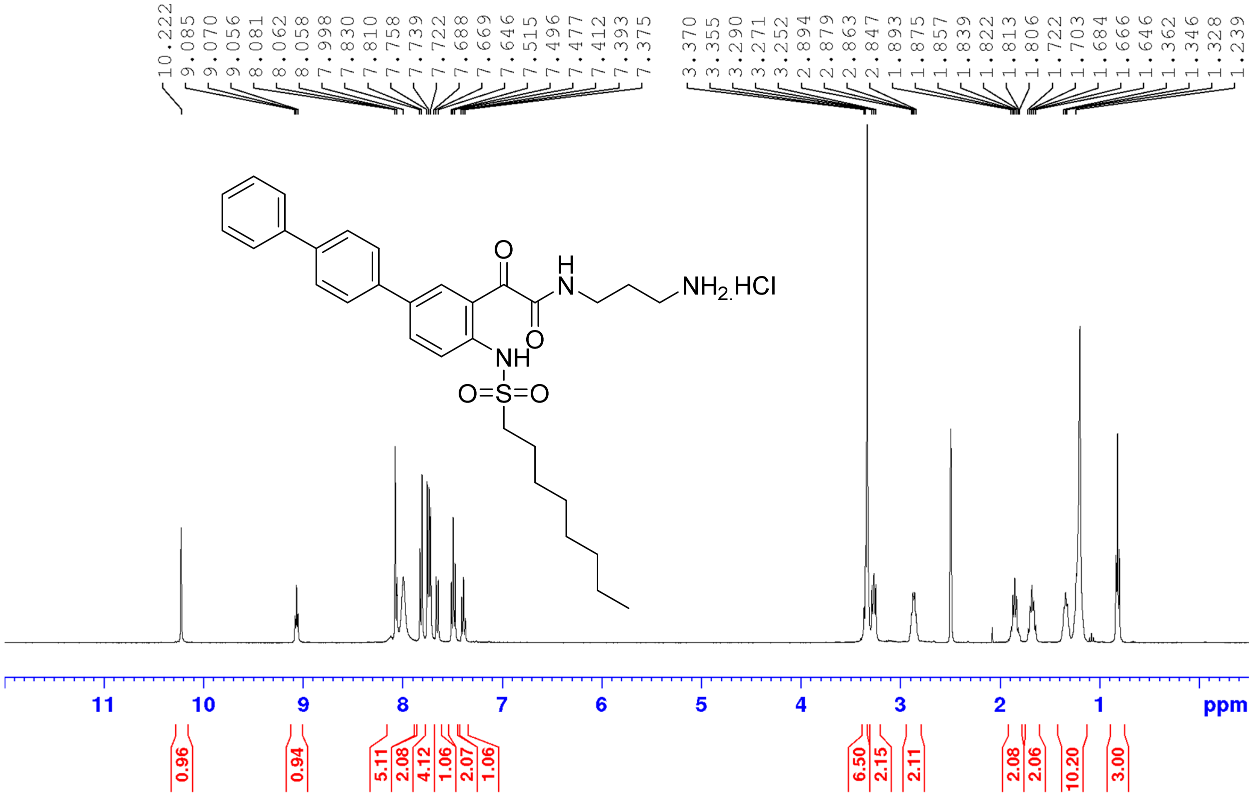

^{13}C NMR (150 MHz, DMSO-d_6):

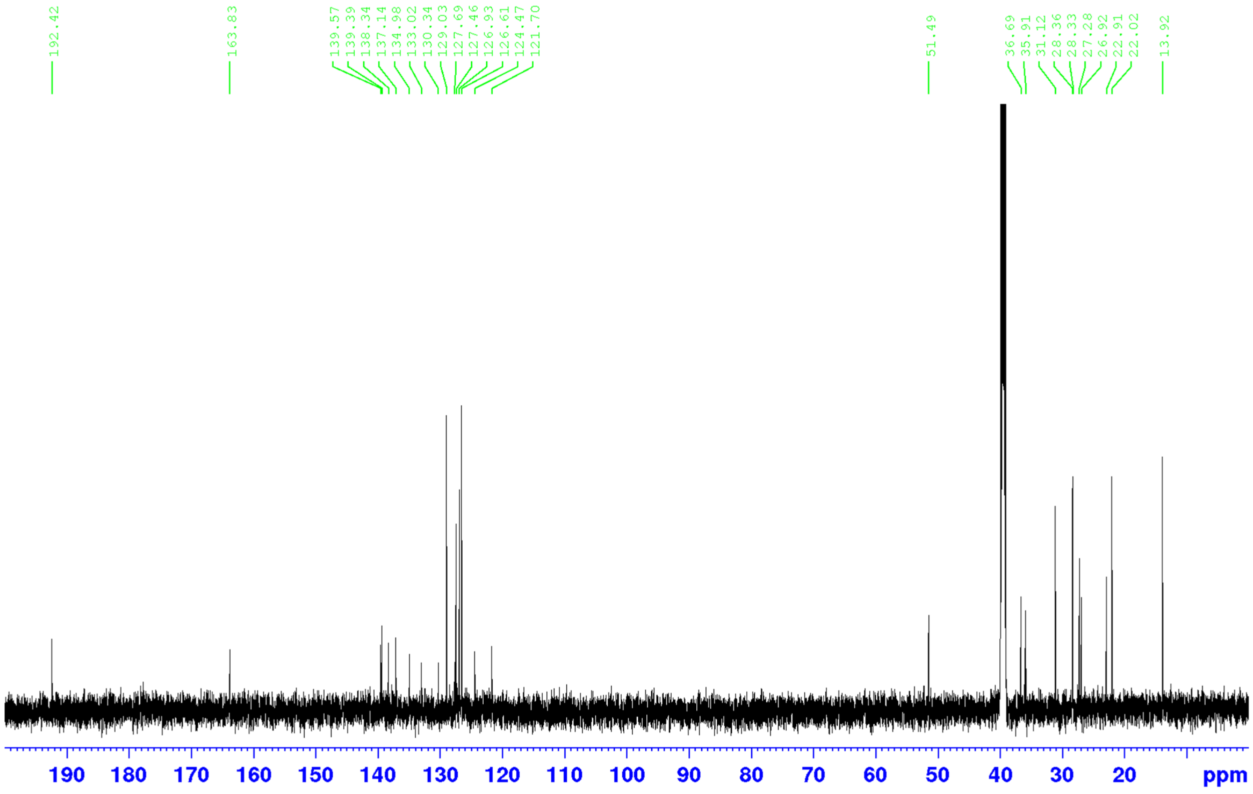

N-(3-Aminopropyl)-2-(2-(dodecylsulfonamido)phenyl)-2-oxoacetamide hydrochloride (**28a**)

^{1}H NMR (400 MHz, DMSO-d_6):

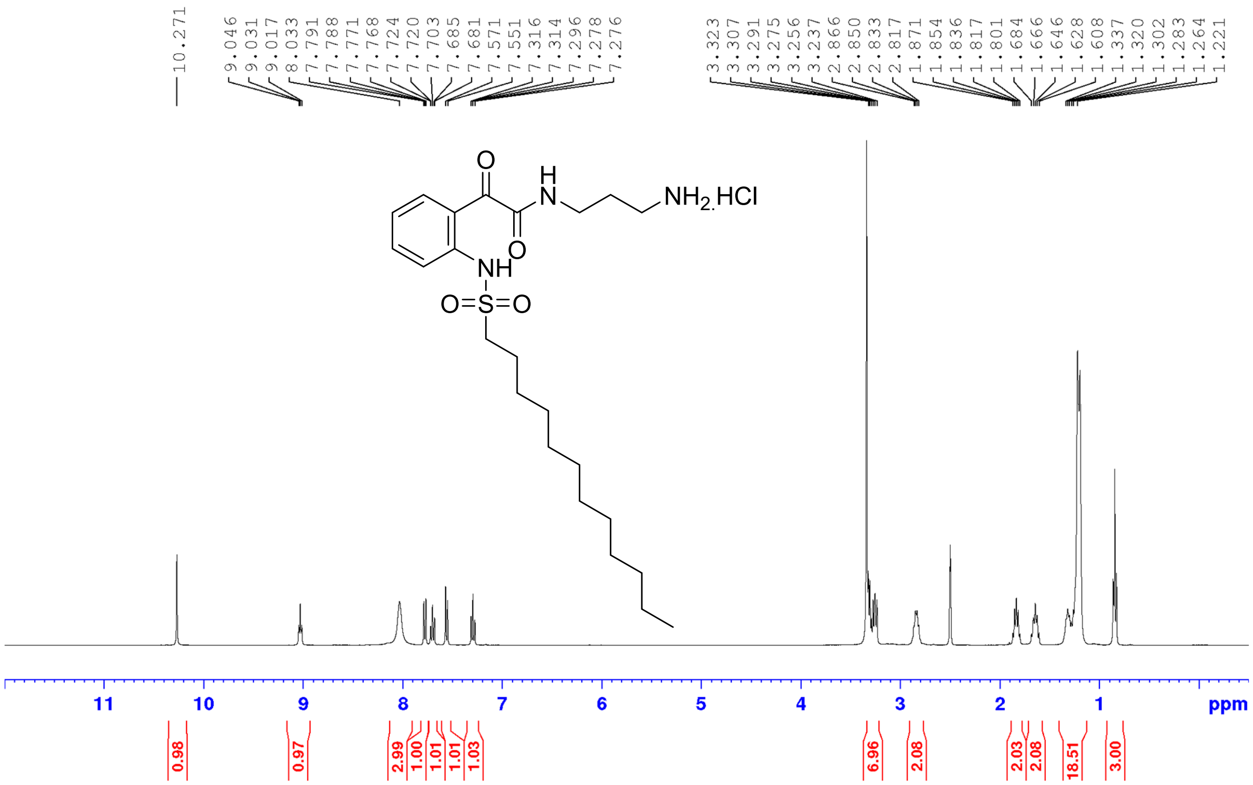

^{13}C NMR (100 MHz, DMSO-d_6):

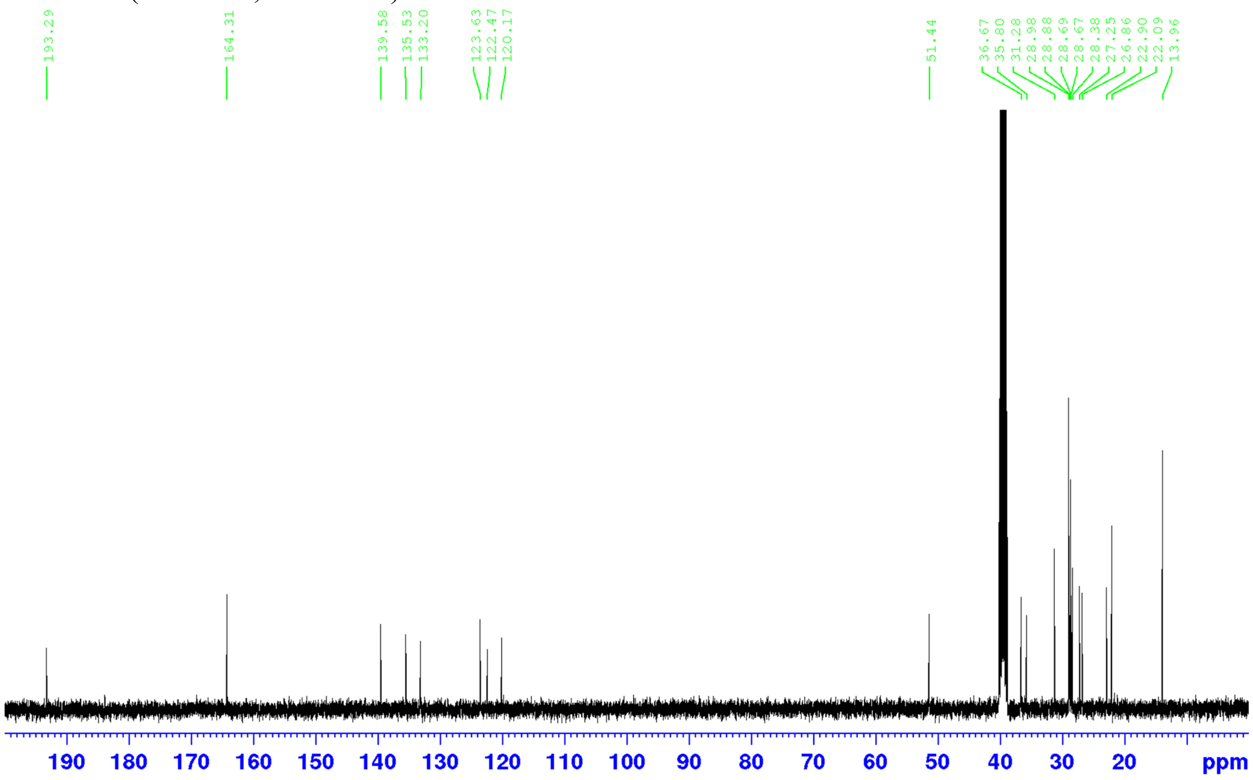

N-(3-Aminopropyl)-2-(5-bromo-2-(dodecylsulfonamido)phenyl)-2-oxoacetamide hydrochloride (**28b**)

^{1}H NMR (400 MHz, DMSO-d_6):

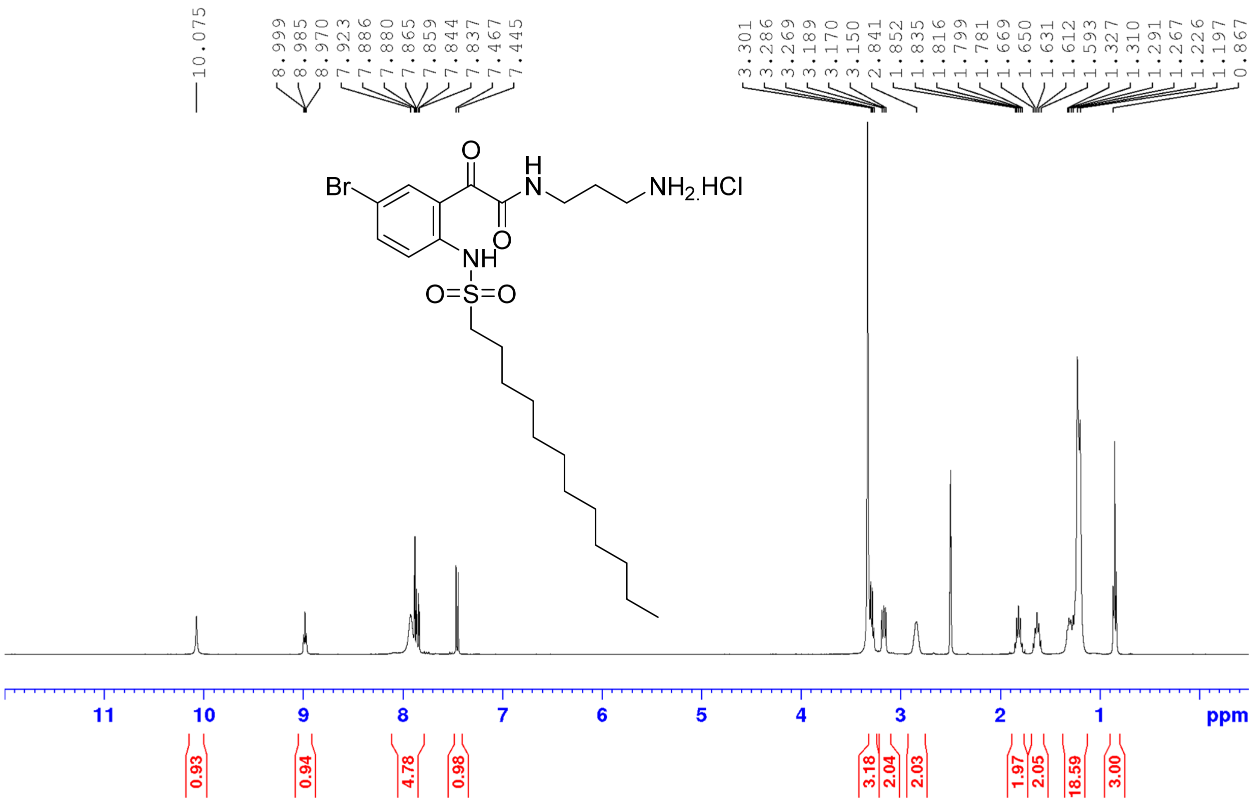

^{13}C NMR (100 MHz, DMSO-d_6):

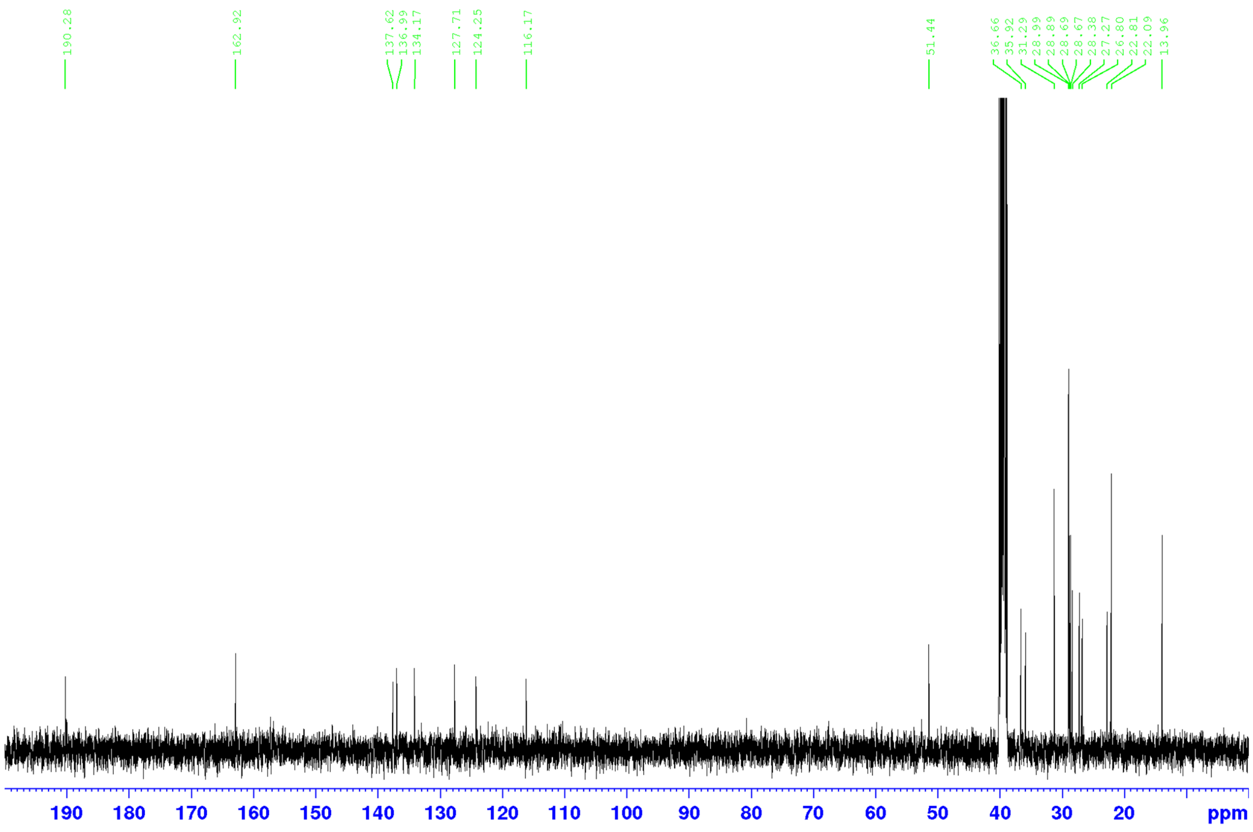

N-(3-aminopropyl)-2-(4-(dodecylsulfonamido)-[1,1'-biphenyl]-3-yl)-2-oxoacetamide hydrochloride (**28c**)

^{1}H NMR (600 MHz, DMSO-d_6):

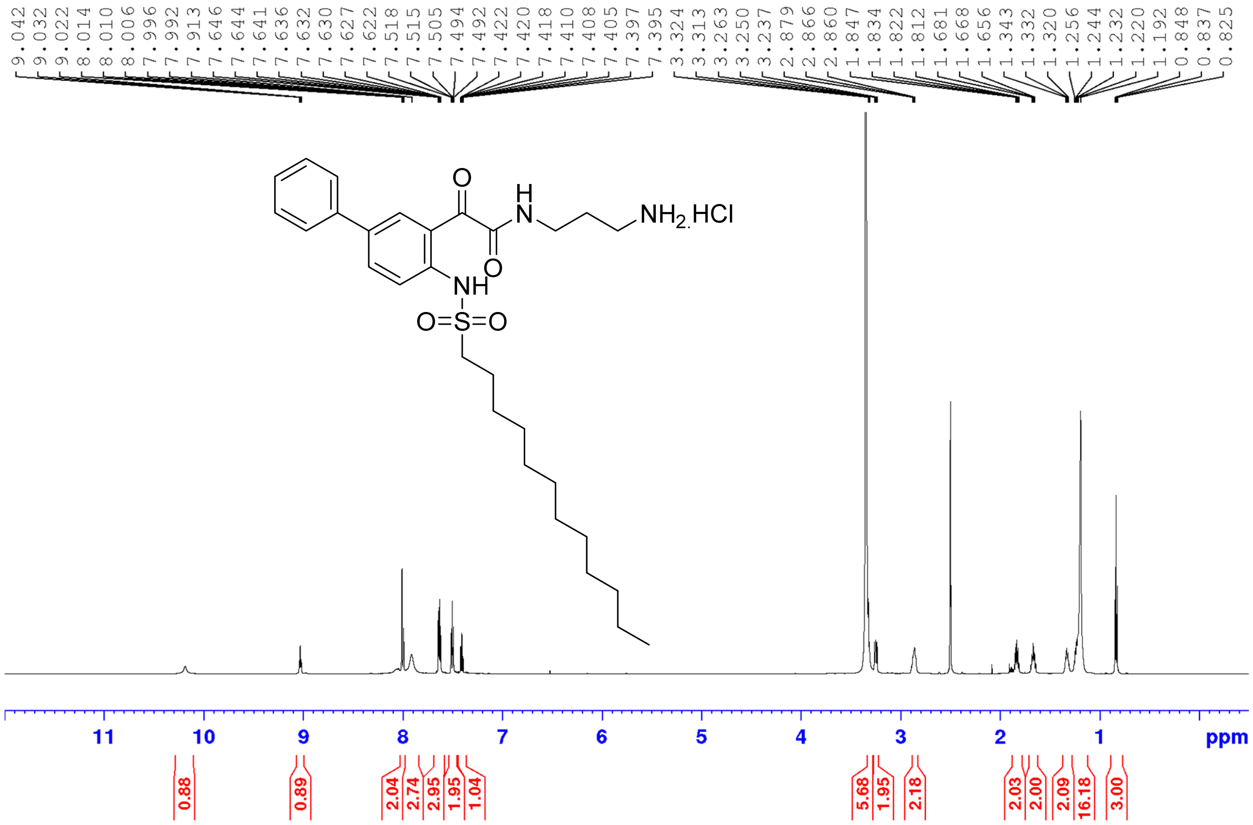

^{13}C NMR (150 MHz, DMSO-d_6):

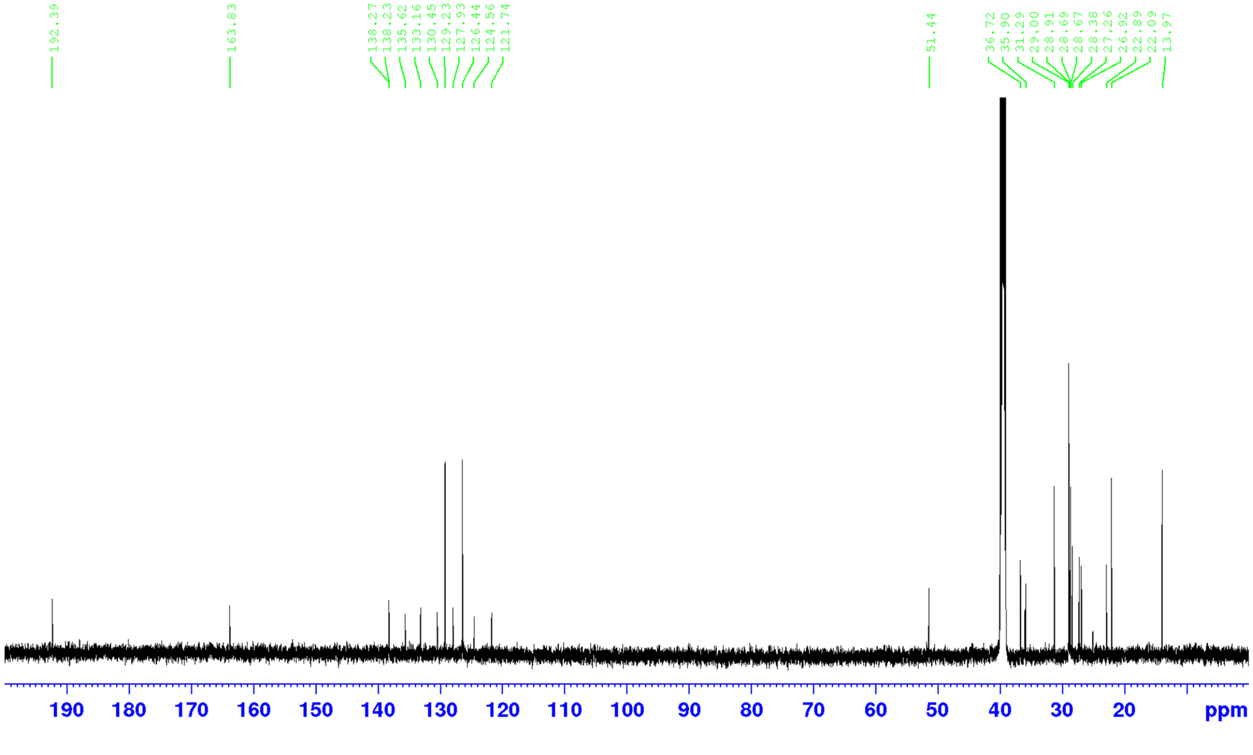

N-(3-Aminopropyl)-2-(2-(hexadecylsulfonamido)phenyl)-2-oxoacetamide hydrochloride (**29a**)

^{1}H NMR (400 MHz, DMSO-d_6):

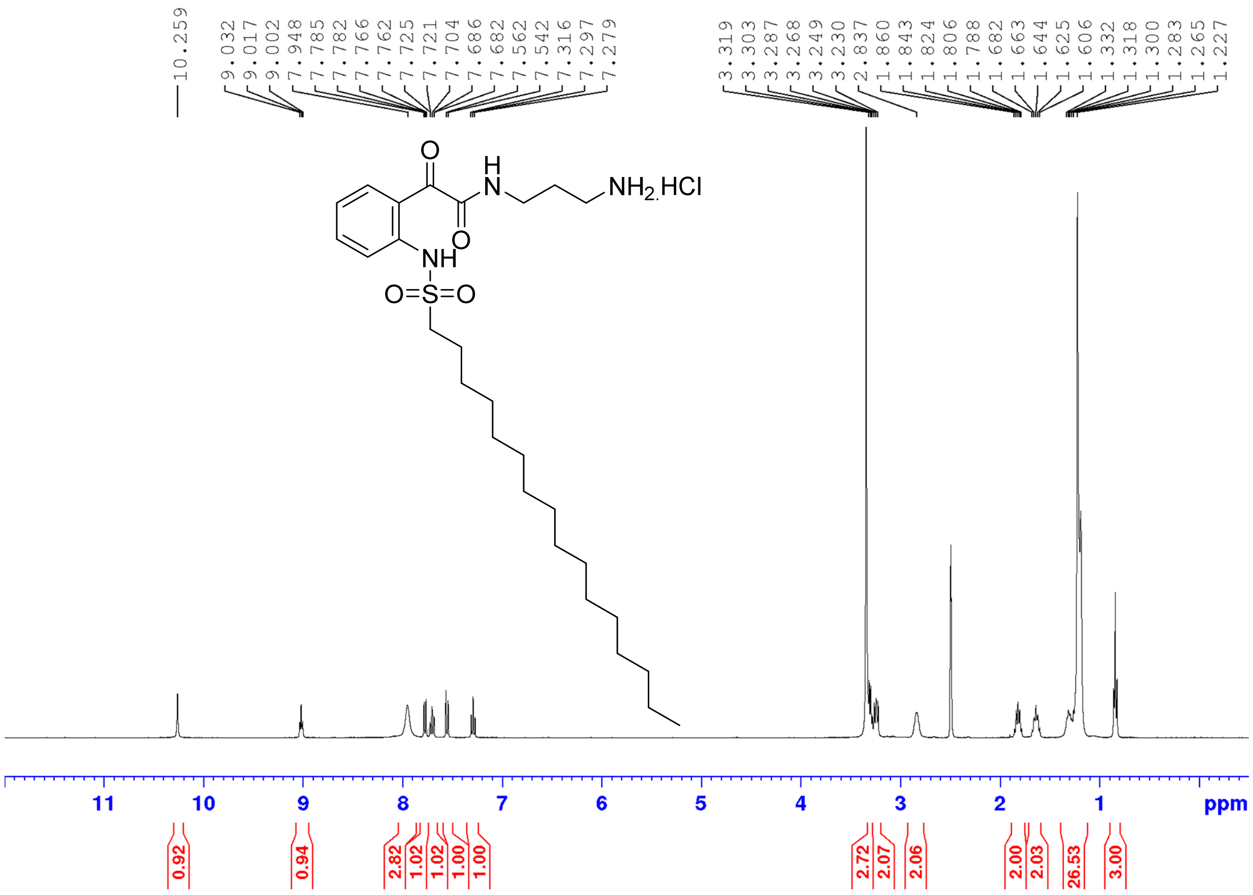

^{13}C NMR (100 MHz, DMSO-d_6):

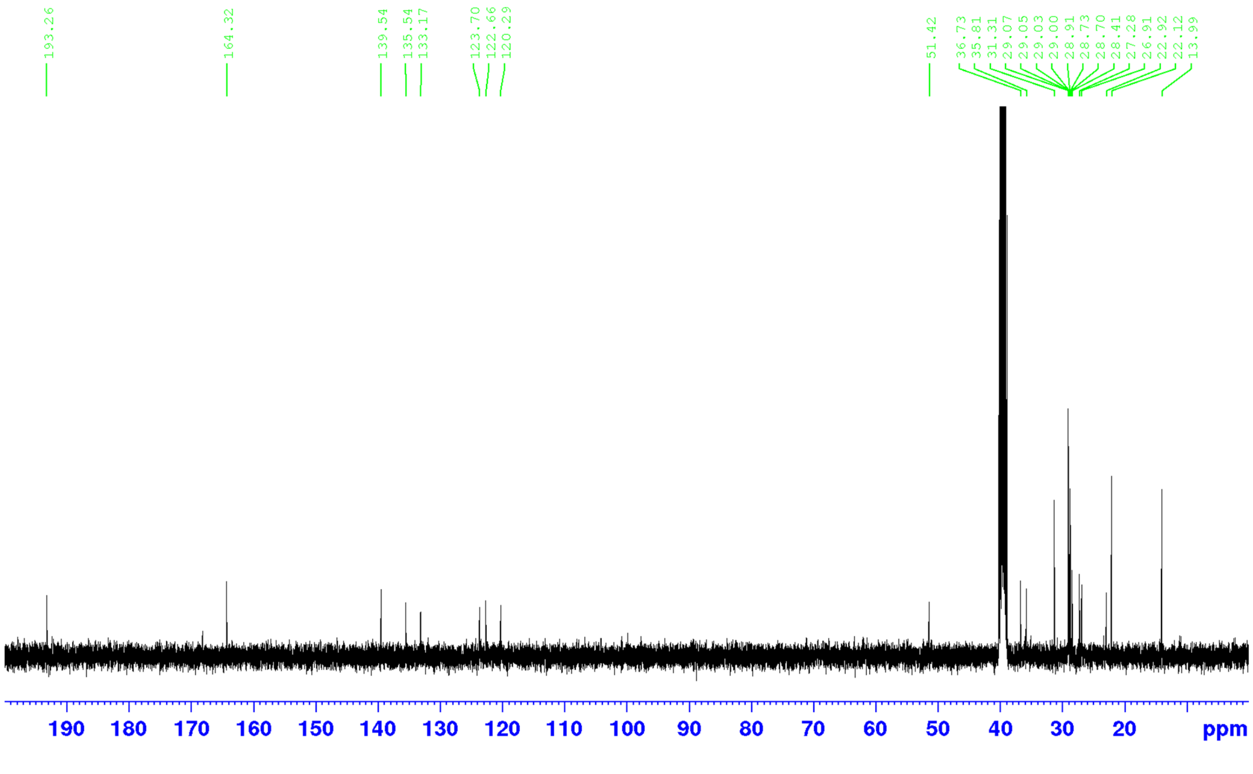

N-(3-Aminopropyl)-2-(5-bromo-2-(hexadecylsulfonamido)phenyl)-2-oxoacetamide hydrochloride (**29b**)

^{1}H NMR (400 MHz, DMSO-d_6):

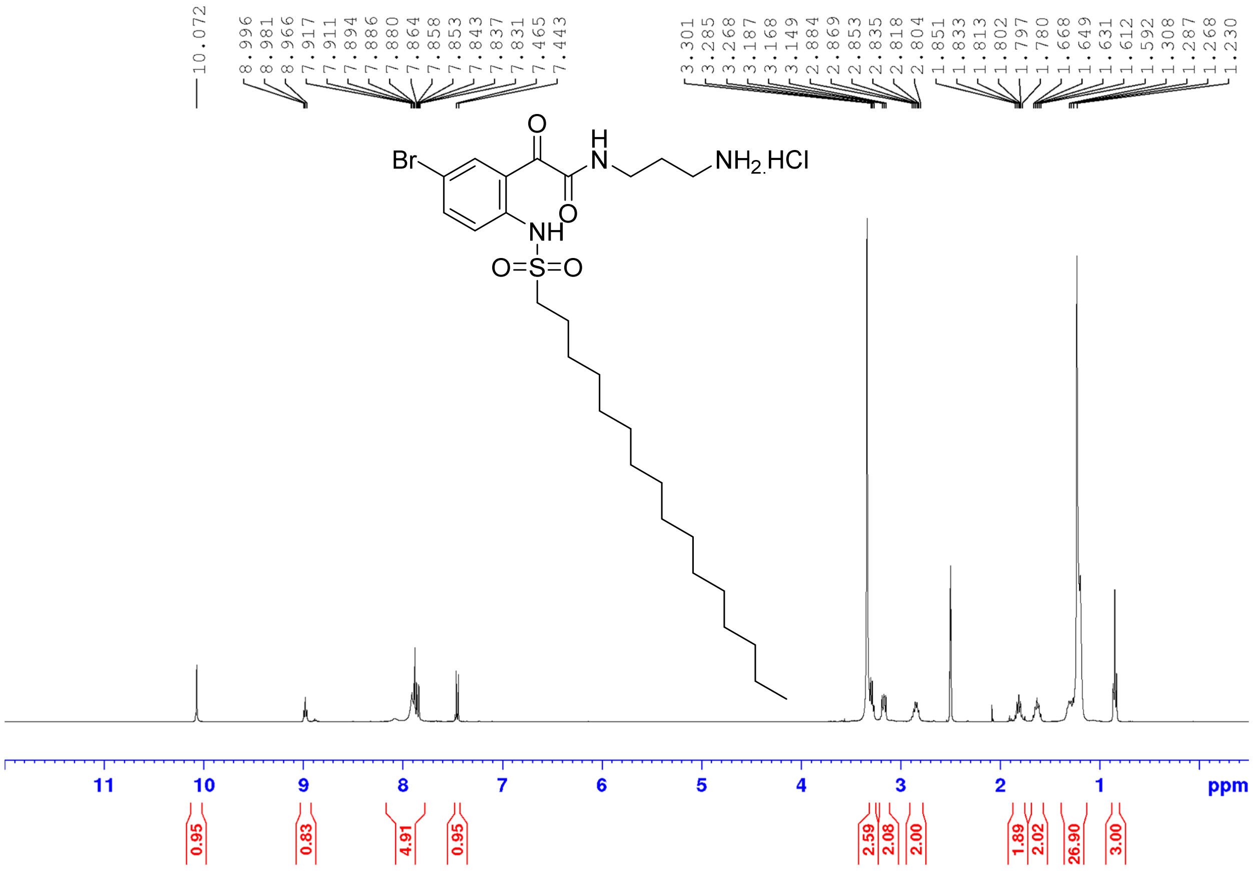

^{13}C NMR (100 MHz, DMSO-d_6):

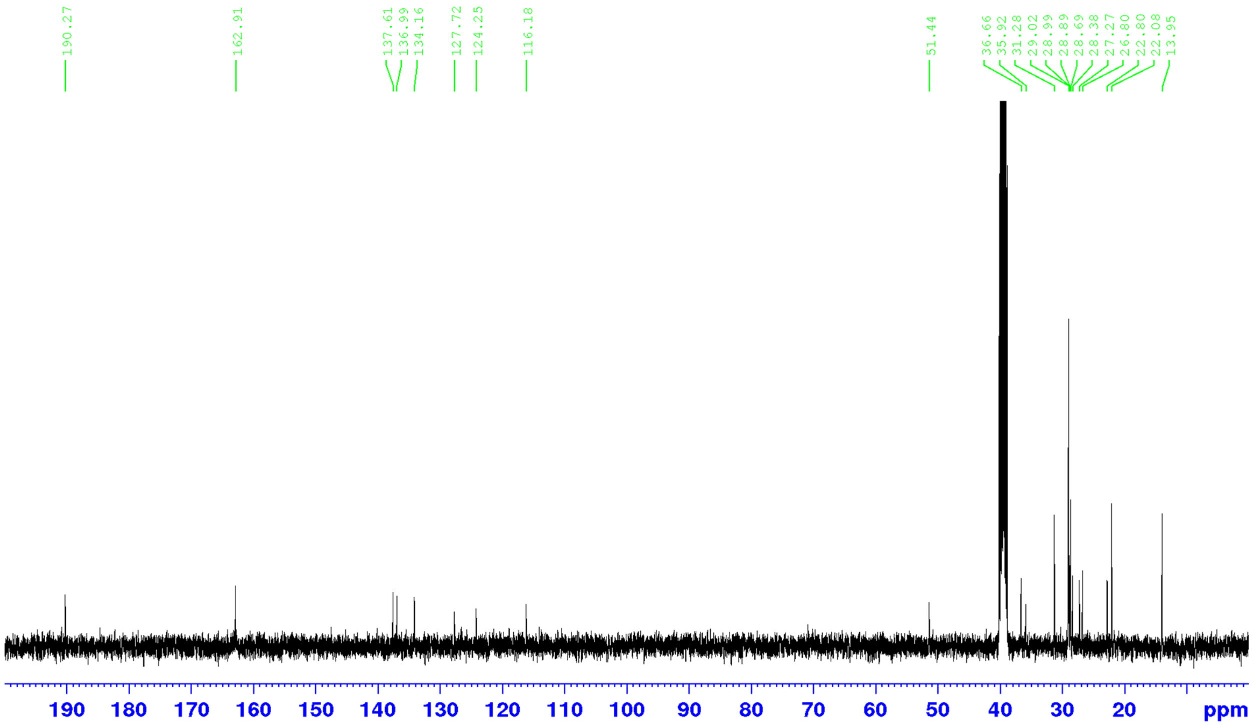

N-(3-Aminopropyl)-2-(4-(hexadecylsulfonamido)-[1,1'-biphenyl]-3-yl)-2-oxoacetamide hydrochloride (**29c**)

^{1}H NMR (400 MHz, DMSO-*d*$_6$):

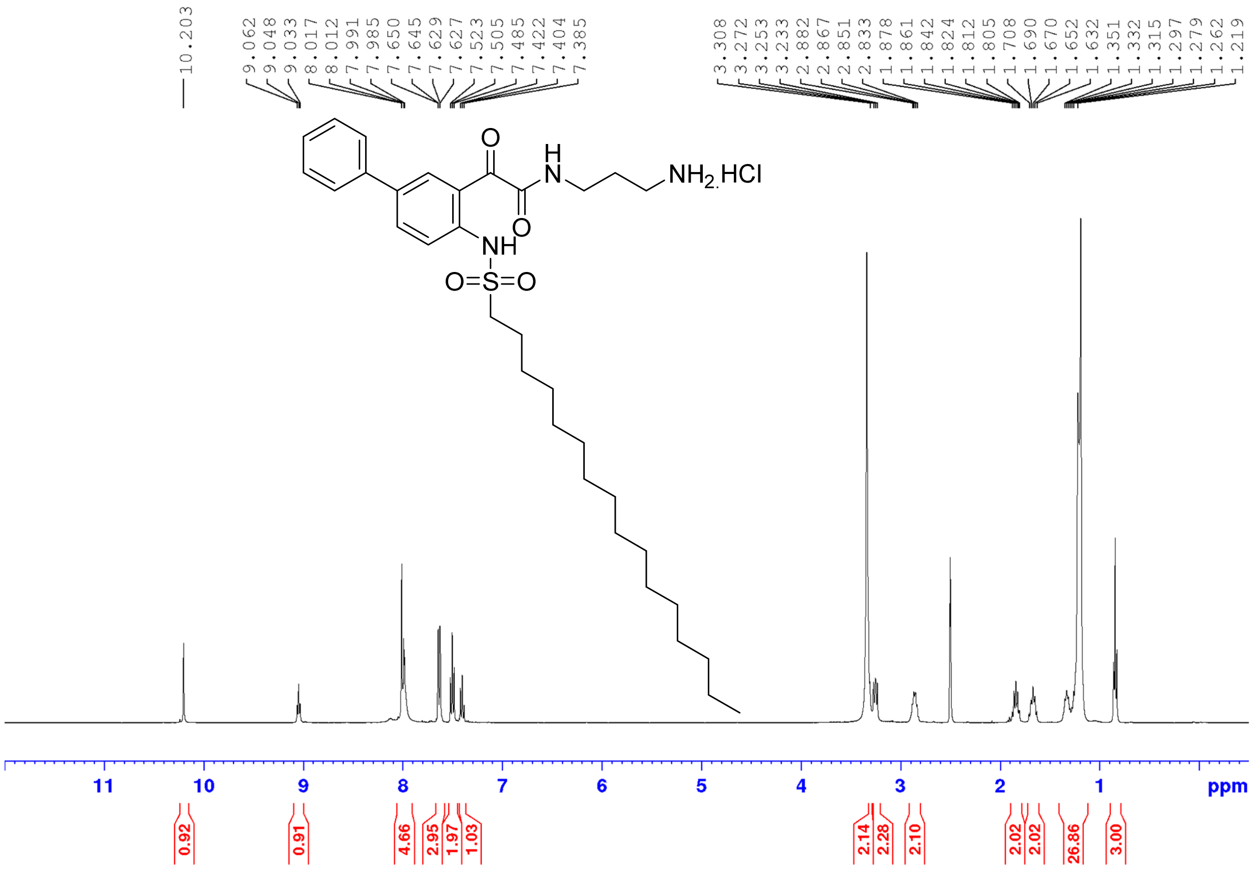

^{13}C NMR (100 MHz, DMSO-*d*$_6$):

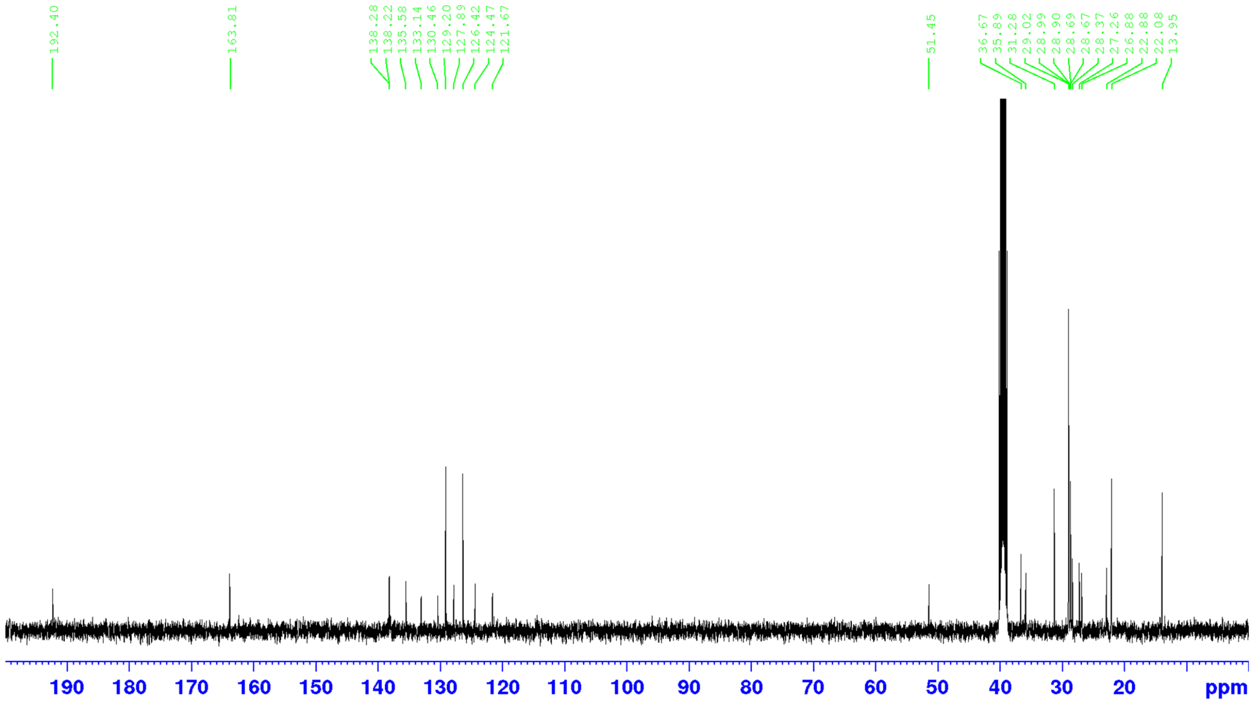

(*E*)-1-*tert*-Butyl-*N*-(*N'*-((*tert*-butyloxidanyl)carbonyl)-*N*-(3-(2-(4-(octylsulfonamido)-[1,1':4',1''-terphenyl]-3-yl)-2-oxoacetamido)propyl)carbamimidoyl)-1-oxidanecarboxamide (**30d**)

^{1}H NMR (400 MHz, $CDCl_3$):

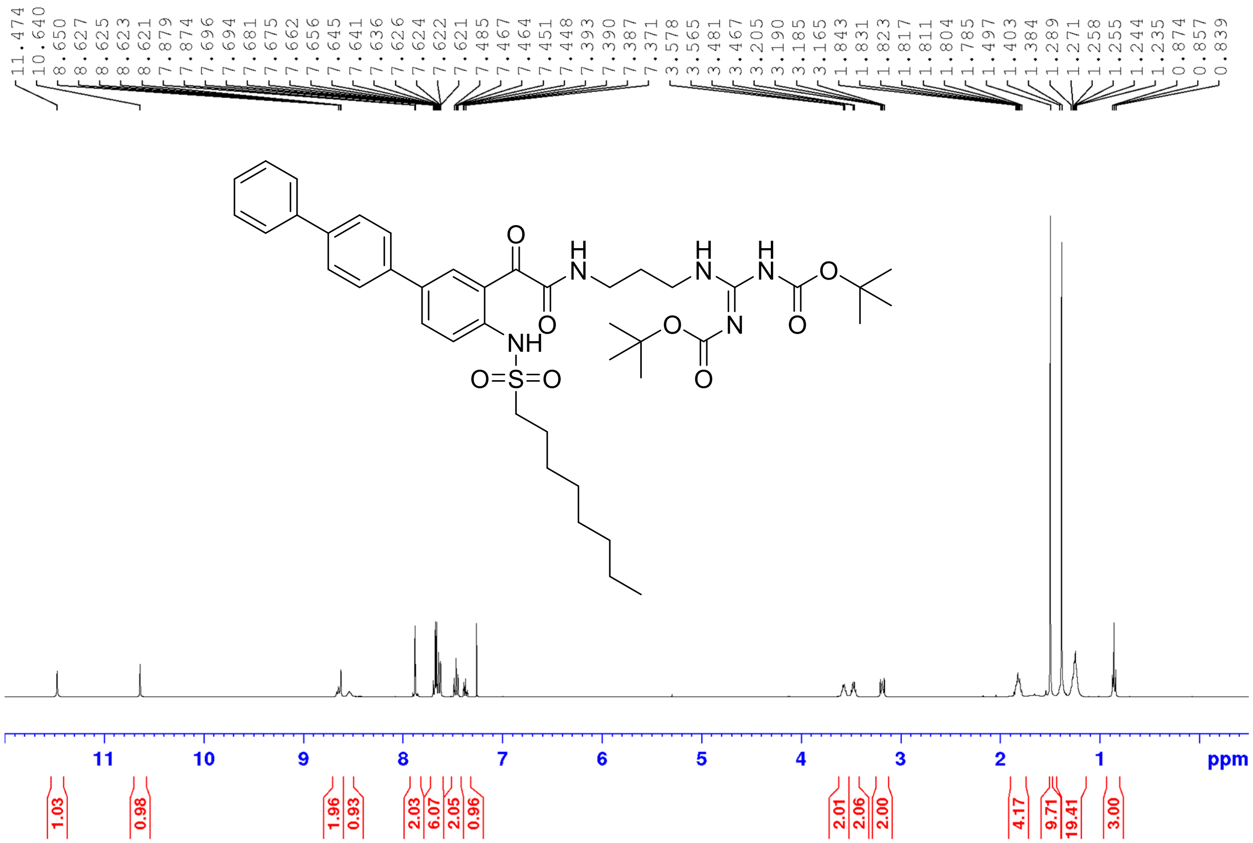

^{13}C NMR (100 MHz, $CDCl_3$):

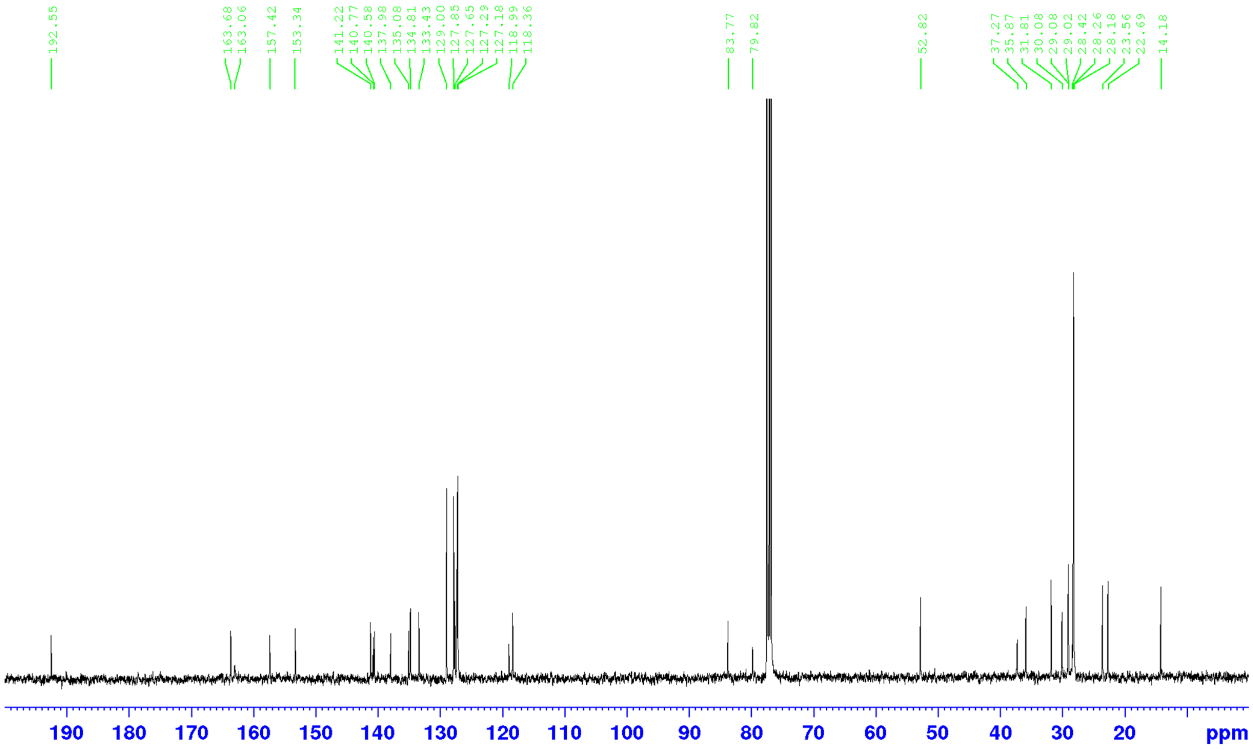

(*E*)-1-*tert*-Butyl-*N*-(*N'*-((tert-butyloxidanyl)carbonyl)-*N*-(3-(2-(2-(dodecylsulfonamido)phenyl)-2-oxoacetamido)propyl)carbamimidoyl)-1-oxidanecarboxamide (**31a**)

^{1}H NMR (600 MHz, $CDCl_3$):

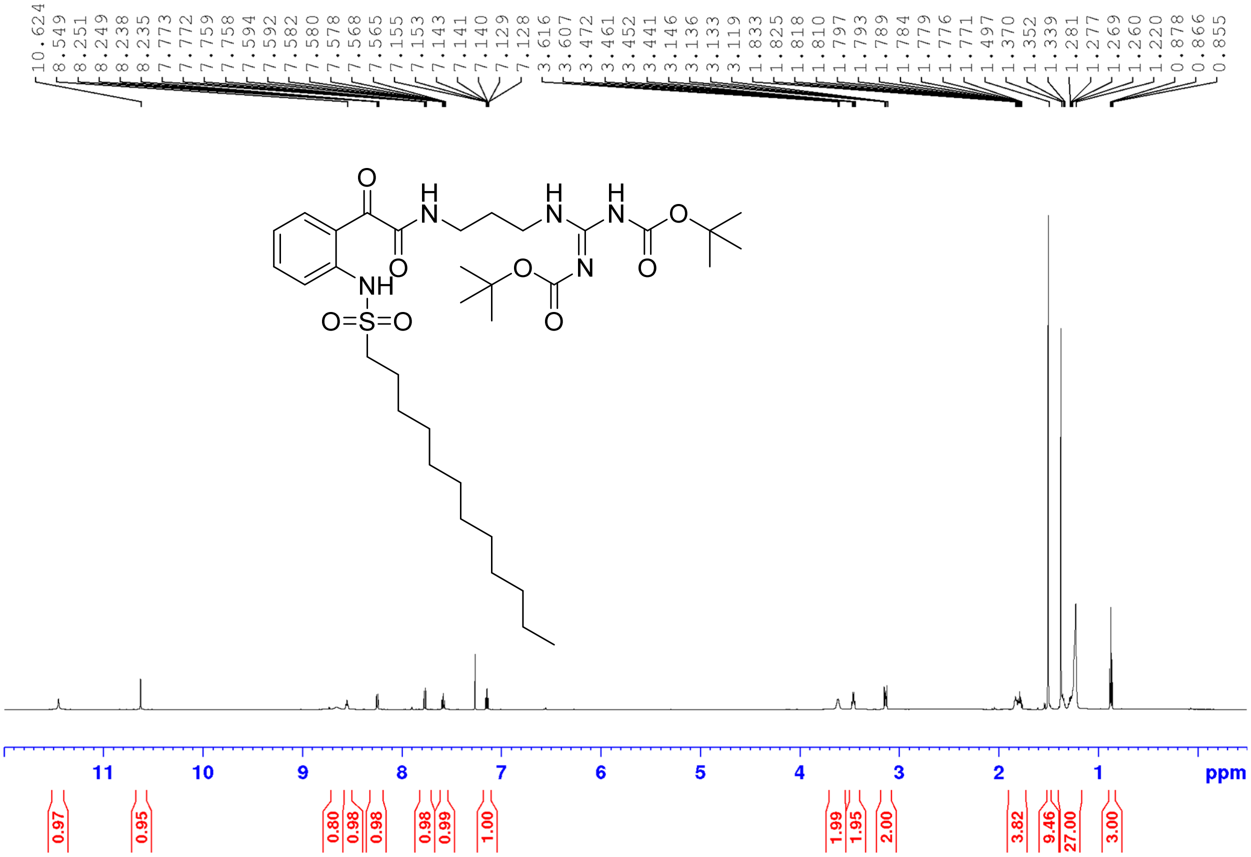

^{13}C NMR (150 MHz, $CDCl_3$):

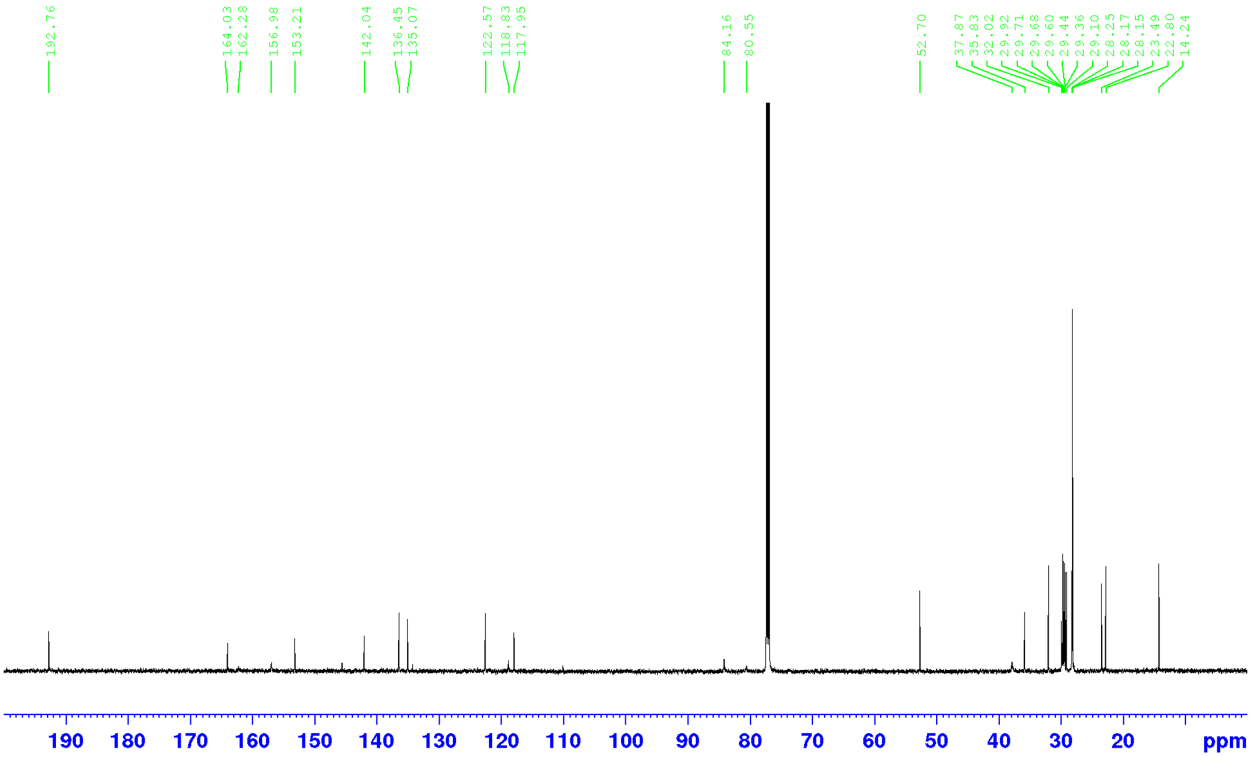

(*E*)-1-*tert*-Butyl-*N*-(*N'*-((*tert*-butyloxidanyl)carbonyl)-*N*-(3-(2-(5-bromo-2-(dodecylsulfonamido) phenyl)-2-oxoacetamido)propyl)carbamimidoyl)-1-oxidanecarboxamide (**31b**)

^{1}H NMR (400 MHz, $CDCl_3$):

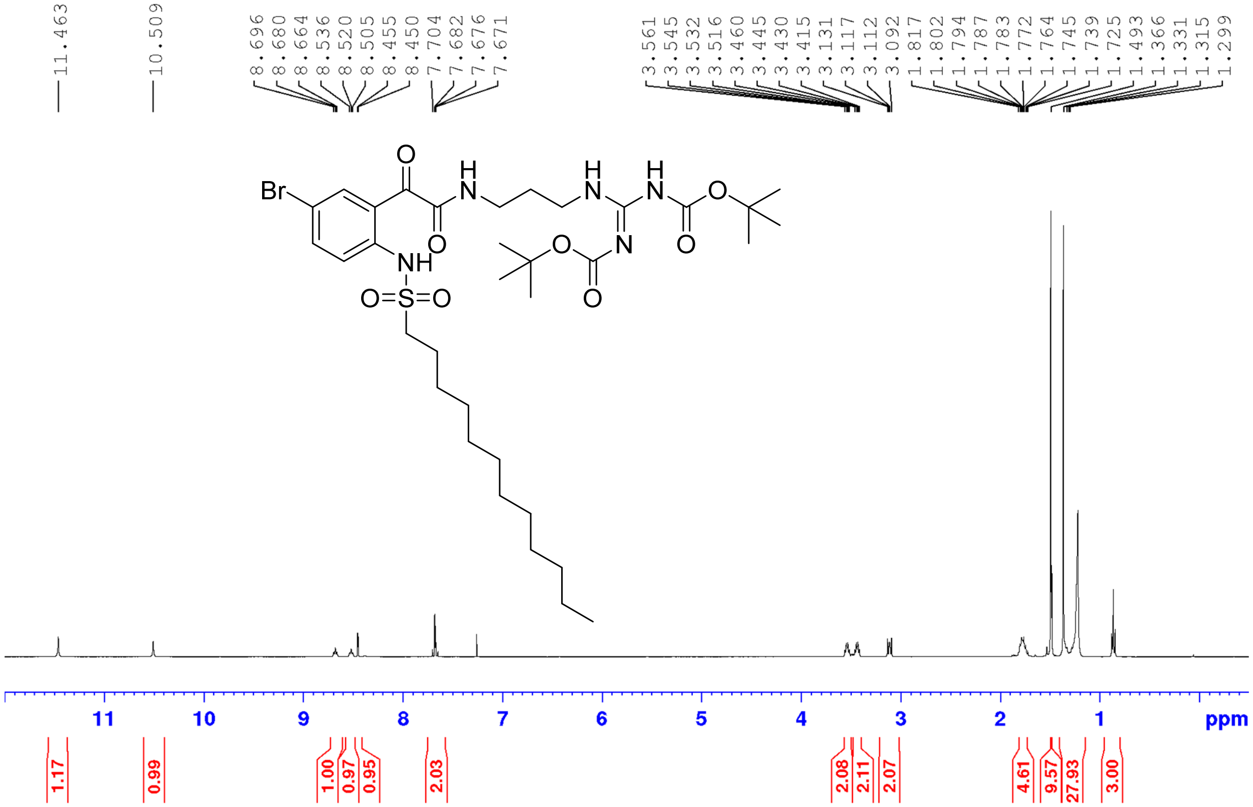

^{13}C NMR (100 MHz, $CDCl_3$):

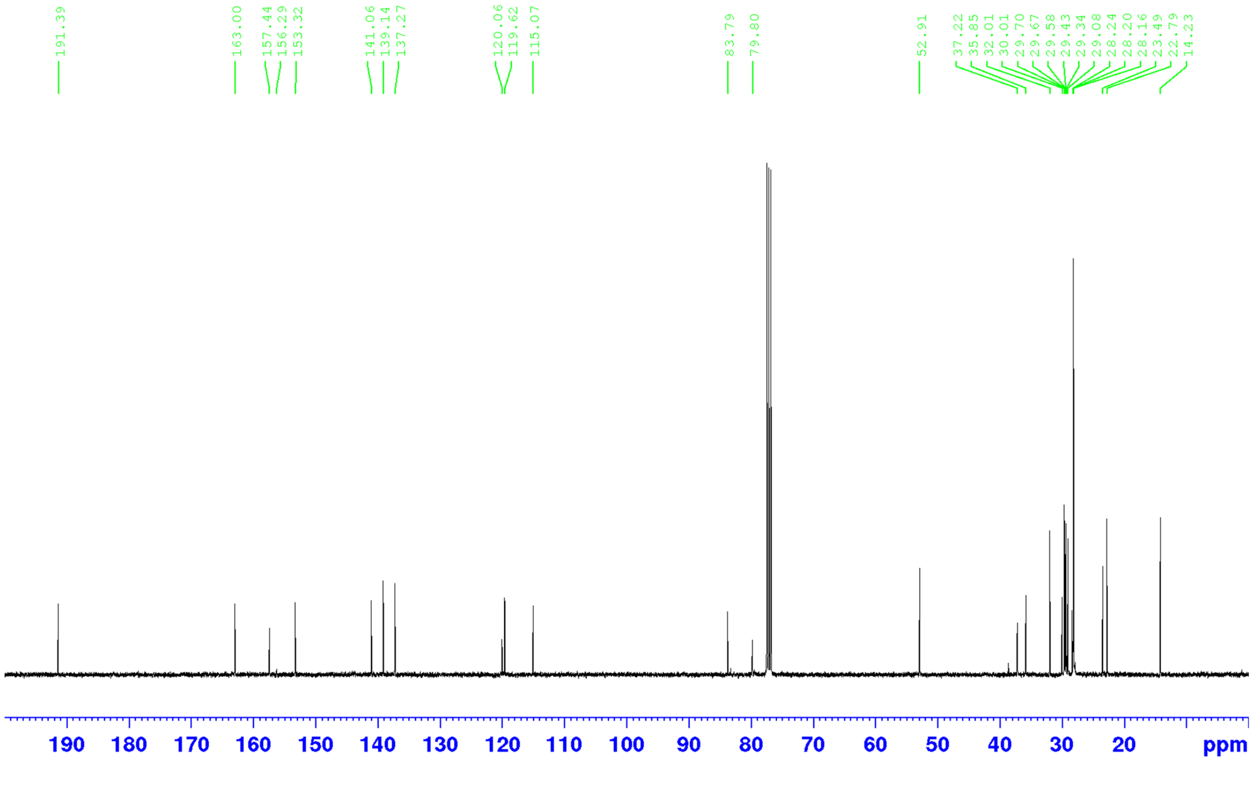

(*E*)-1-*tert*-Butyl-*N*-(*N'*-((*tert*-butyloxidanyl)carbonyl)-*N*-(3-(2-(4-(dodecylsulfonamido)-[1,1'-biphenyl]-3-yl)-2-oxoacetamido)propyl)carbamimidoyl)-1-oxidanecarboxamide (**31c**)

^{1}H NMR (400 MHz, $CDCl_3$):

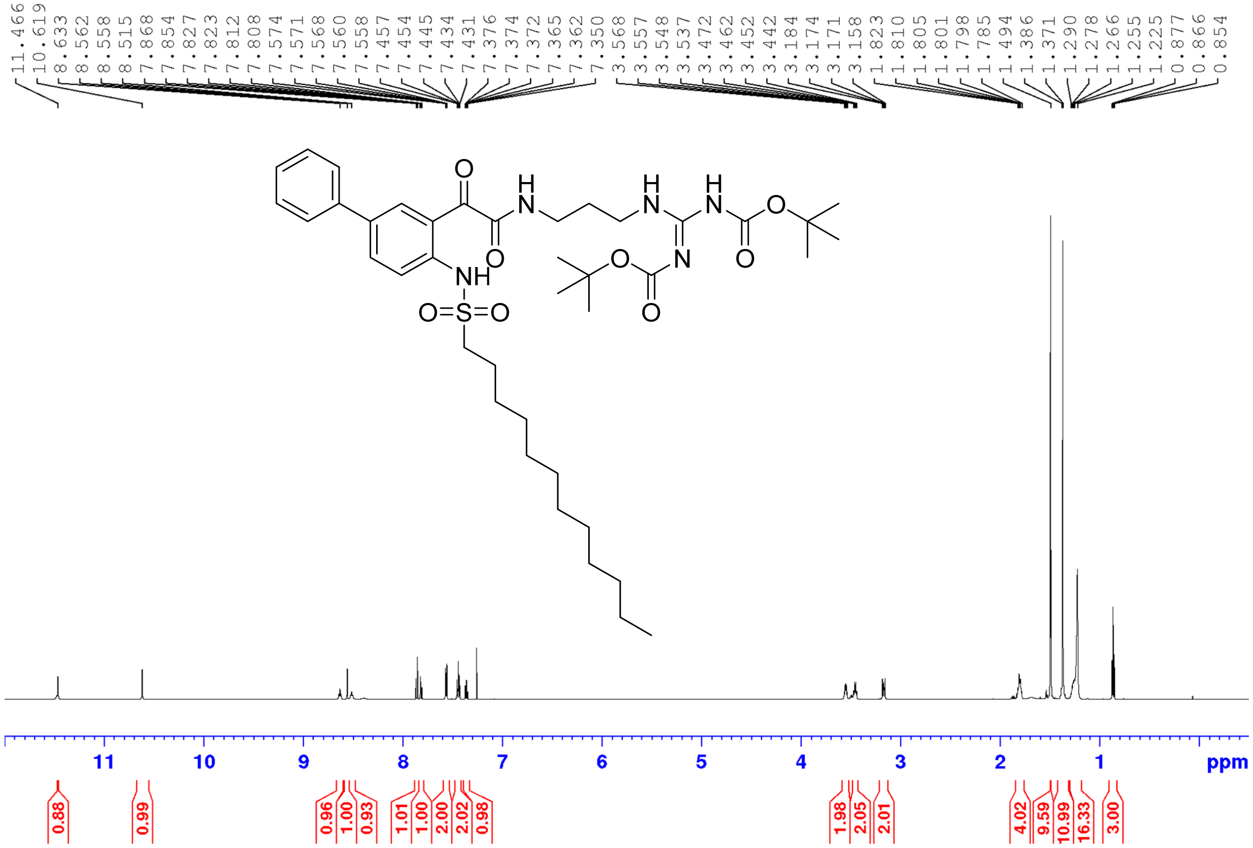

^{13}C NMR (100 MHz, $CDCl_3$):

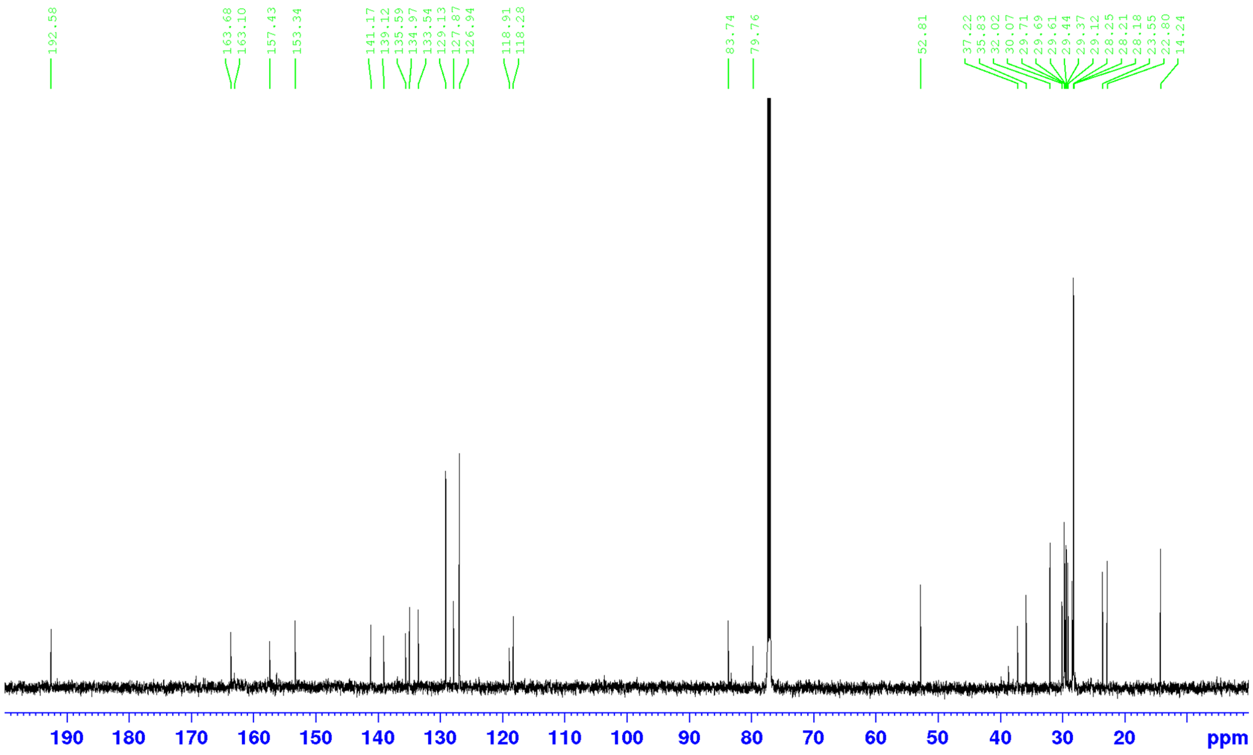

(*E*)-1-*tert*-Butyl-*N*-(*N'*-((*tert*-butyloxidanyl)carbonyl)-*N*-(3-(2-(2-(hexadecylsulfonamido)phenyl)-2-oxoacetamido)propyl)carbamimidoyl)-1-oxidanecarboxamide (**32a**)

^{1}H NMR (400 MHz, $CDCl_3$):

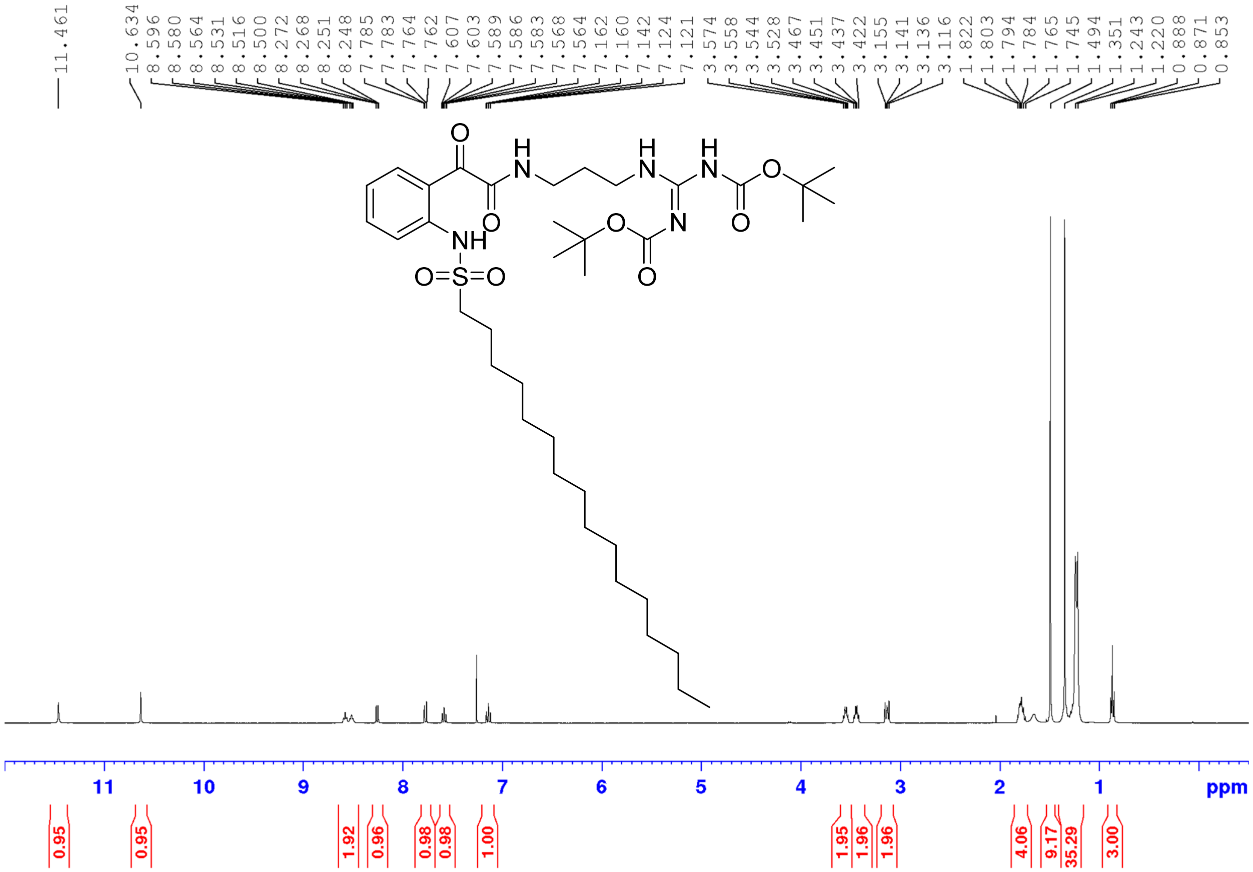

^{13}C NMR (100 MHz, $CDCl_3$):

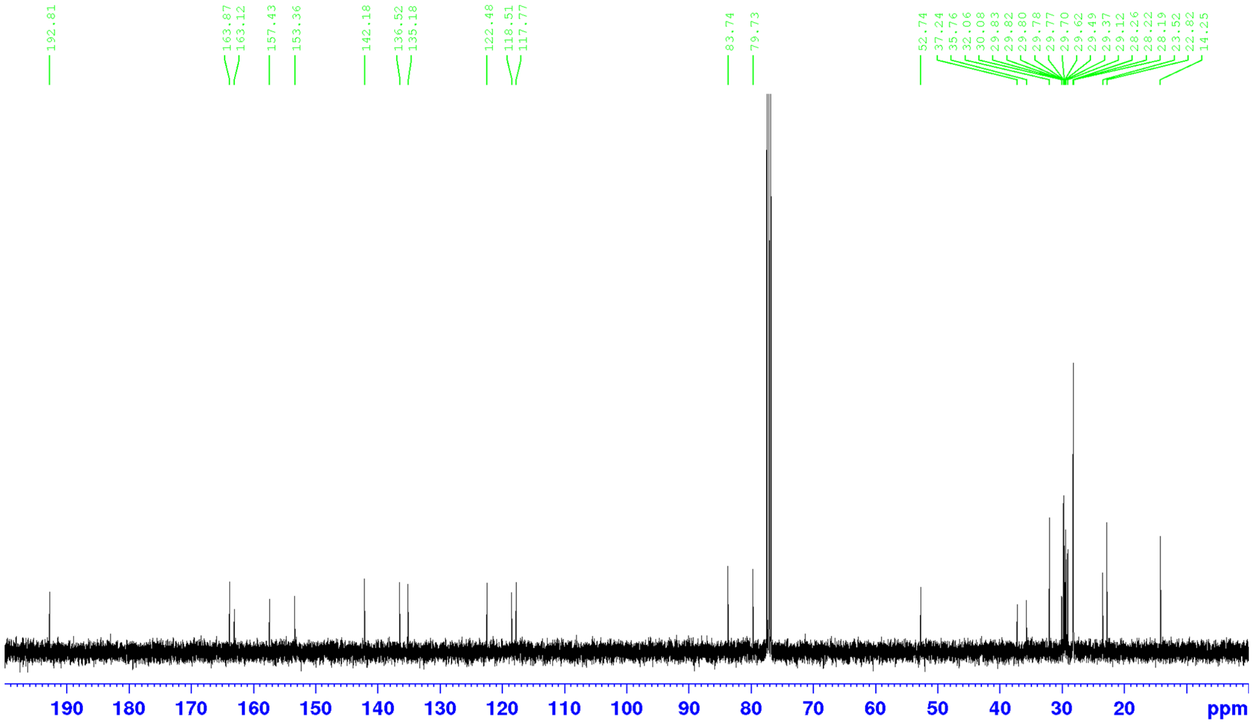

(*E*)-1-*tert*-Butyl-*N*-(*N'*-((*tert*-butyloxidanyl)carbonyl)-*N*-(3-(2-(5-bromo-2-(hexadecylsulfonamido) phenyl)-2-oxoacetamido)propyl)carbamimidoyl)-1-oxidanecarboxamide (**32b**)

^{1}H NMR (600 MHz, $CDCl_3$):

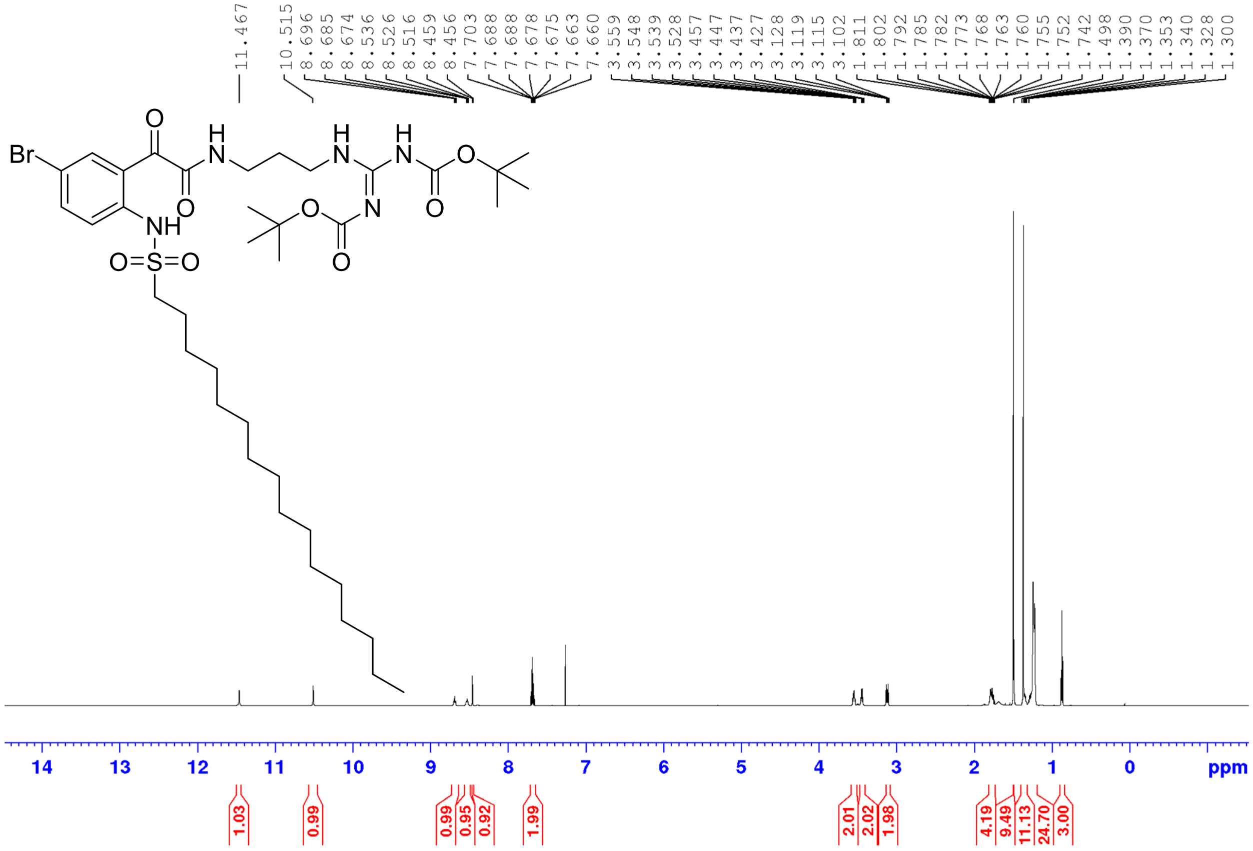

^{13}C NMR (150 MHz, $CDCl_3$):

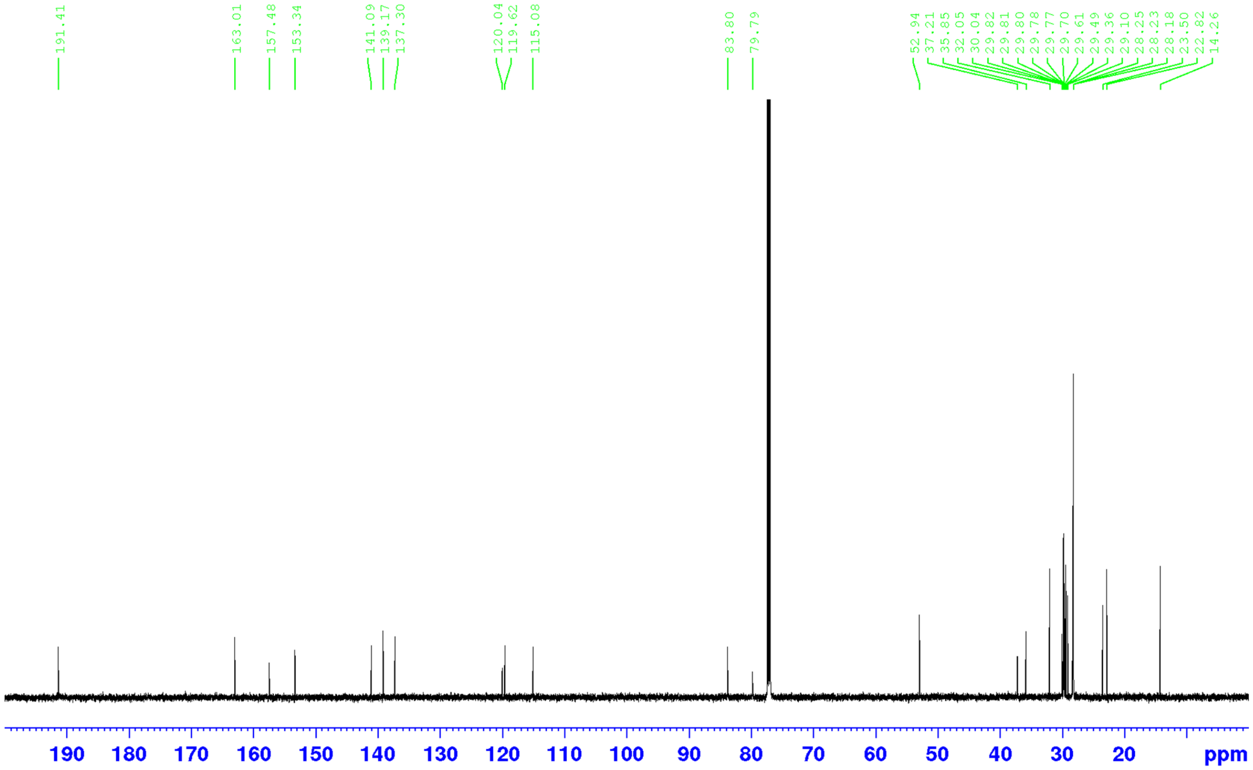

(*E*)-1-*tert*-Butyl-*N*-(*N'*-((*tert*-butyloxidanyl)carbonyl)-*N*-(3-(2-(4-(hexadecylsulfonamido)-[1,1'-biphenyl]-3-yl)-2-oxoacetamido)propyl)carbamimidoyl)-1-oxidanecarboxamide (**32c**)

^{1}H NMR (600 MHz, $CDCl_3$):

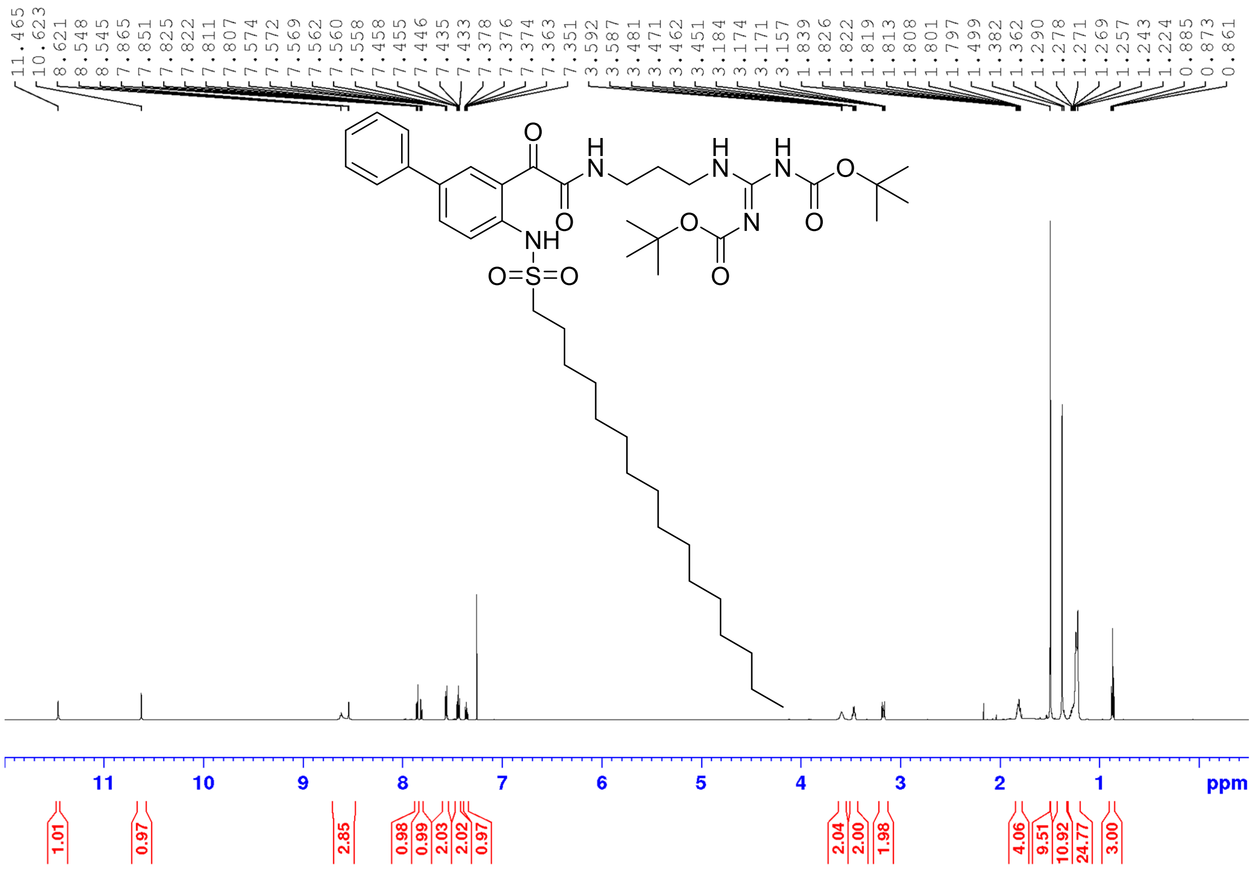

^{13}C NMR (150 MHz, $CDCl_3$):

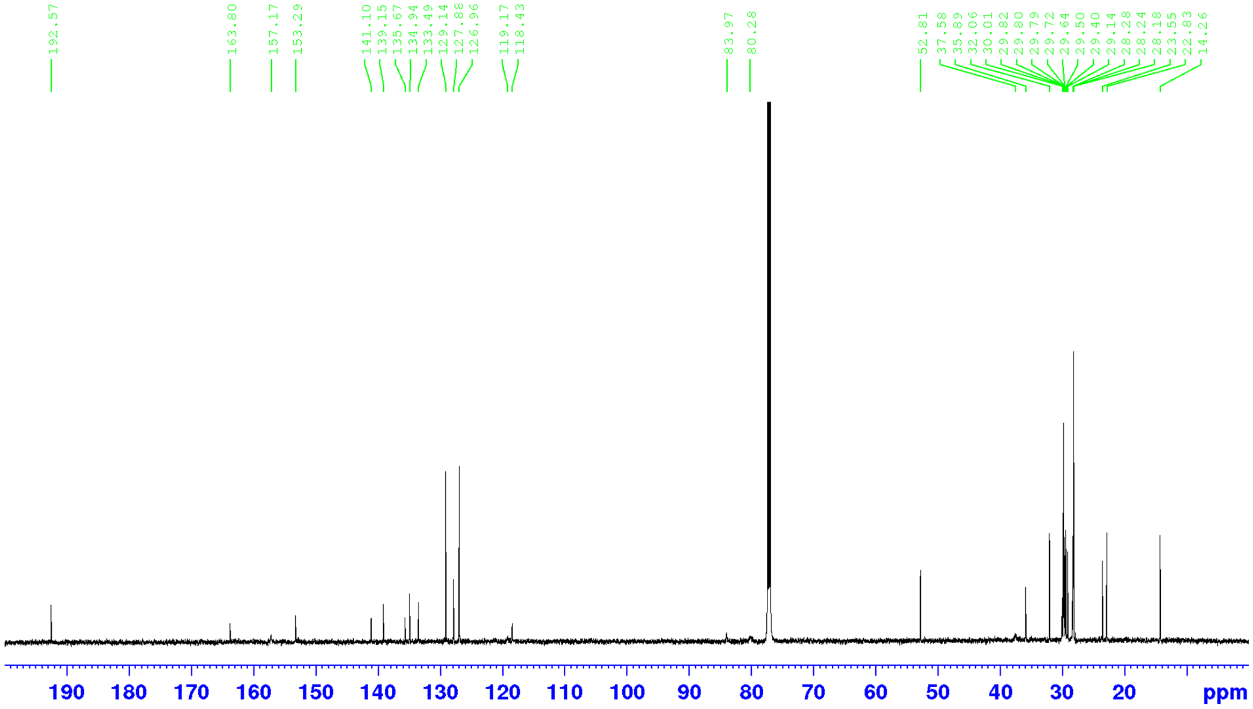

N-(3-Guanidinopropyl)-2-(4-(octylsulfonamido)-[1,1':4',1''-terphenyl]-3-yl)-2-oxoacetamide hydrochloride (**33d**)

^{1}H NMR (600 MHz, DMSO-d_6):

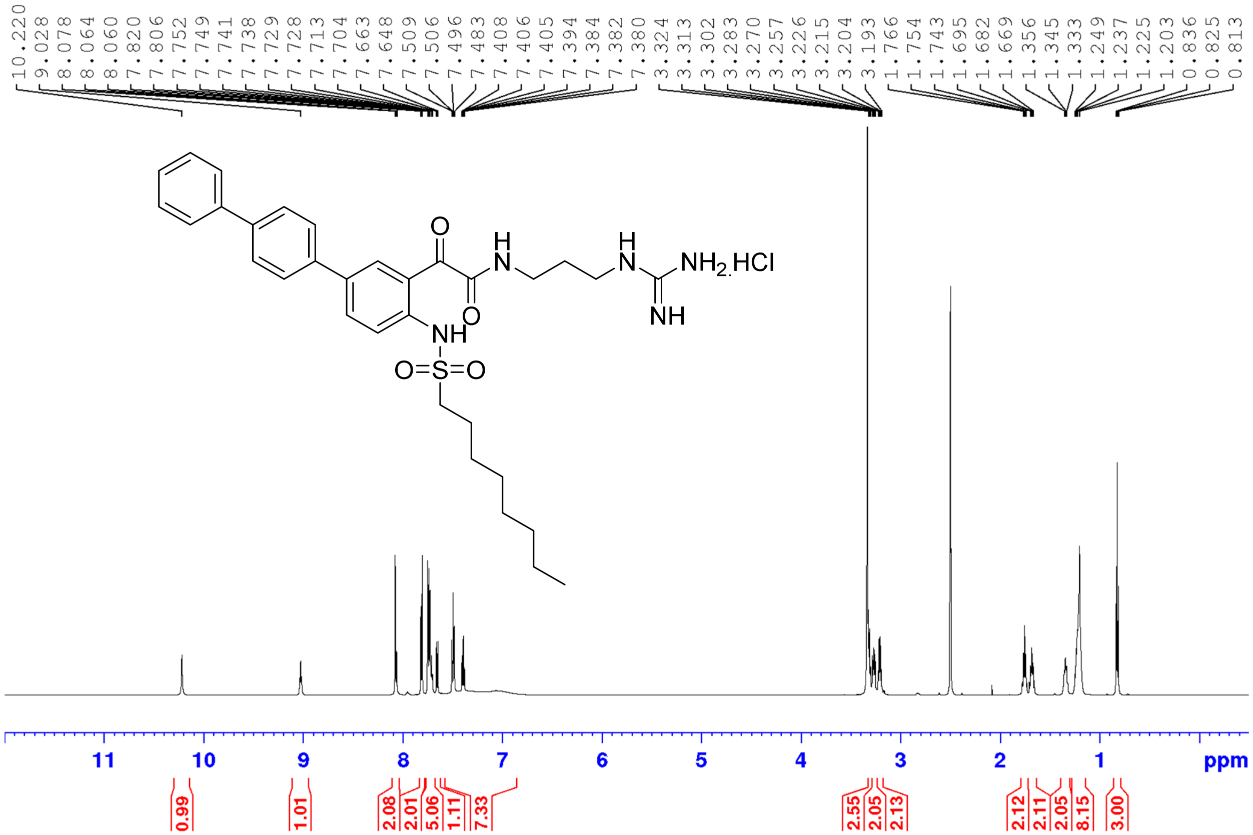

^{13}C NMR (150 MHz, DMSO-d_6):

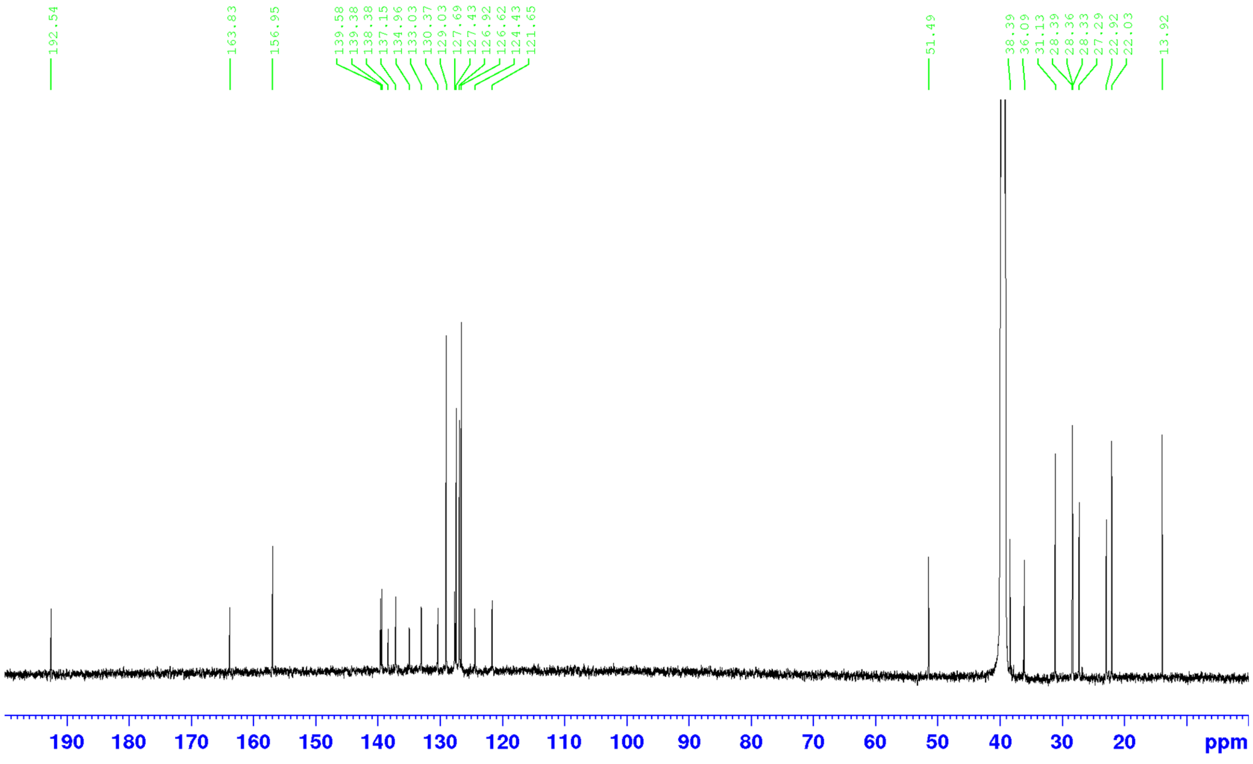

2-(2-(Dodecylsulfonamido)phenyl)-*N*-(3-guanidinopropyl)-2-oxoacetamide hydrochloride (**34a**)

^{1}H NMR (600 MHz, DMSO-*d*$_6$):

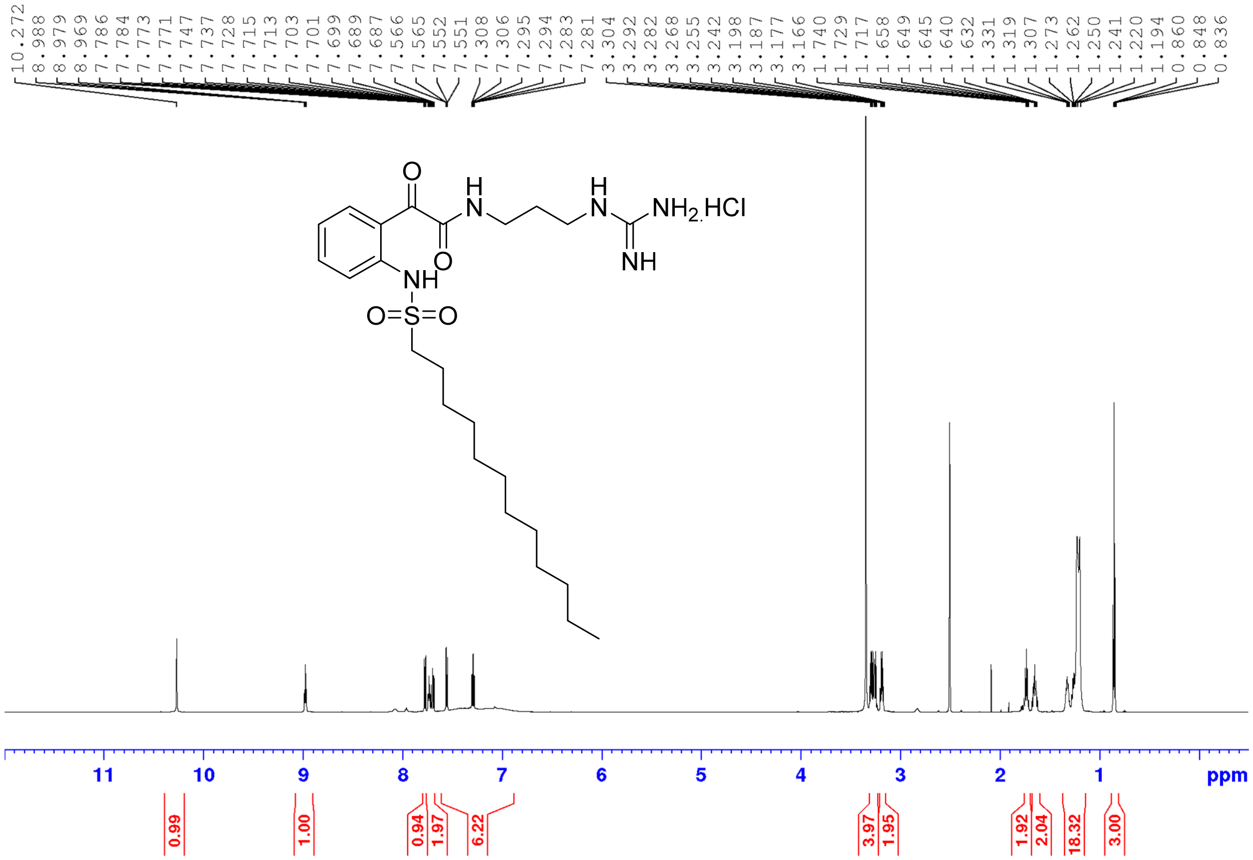

^{13}C NMR (150 MHz, DMSO-*d*$_6$):

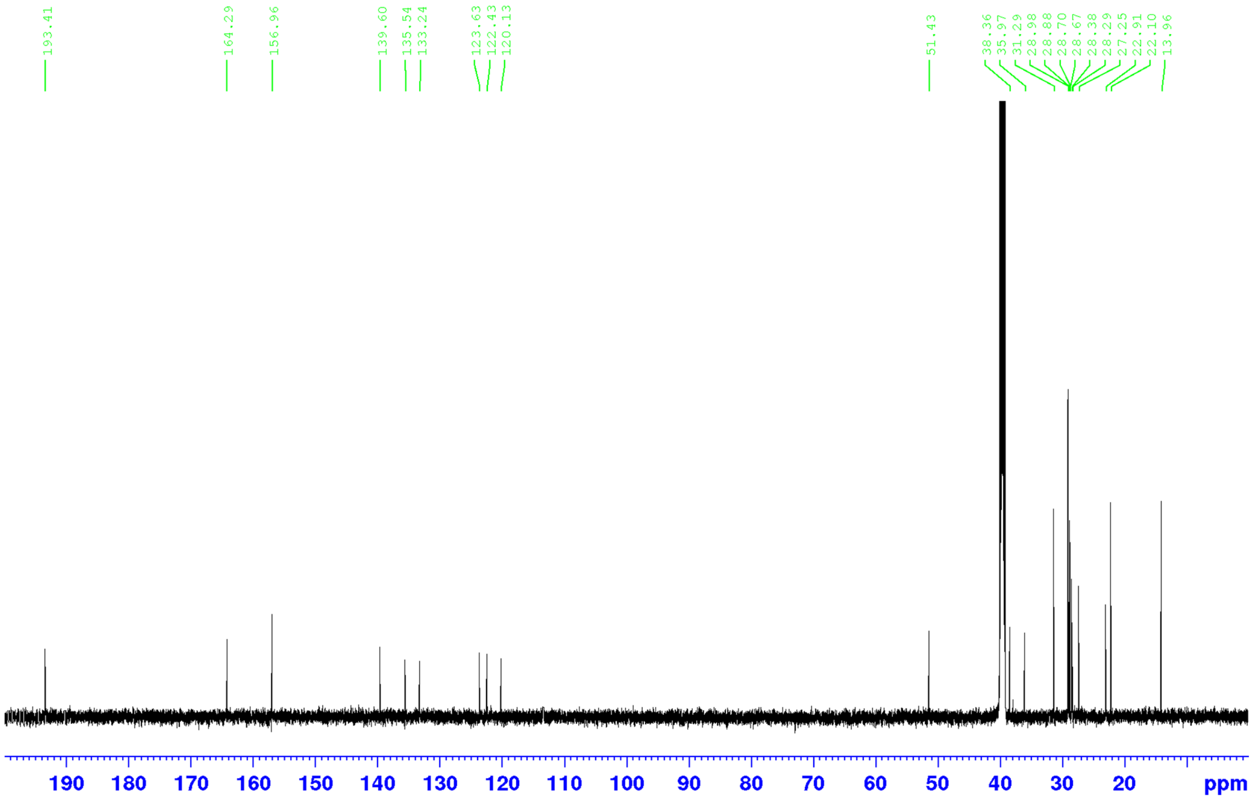

2-(5-Bromo-2-(dodecylsulfonamido)phenyl)-*N*-(3-guanidinopropyl)-2-oxoacetamide hydrochloride (**34b**)

^{1}H NMR (600 MHz, DMSO-*d*$_6$):

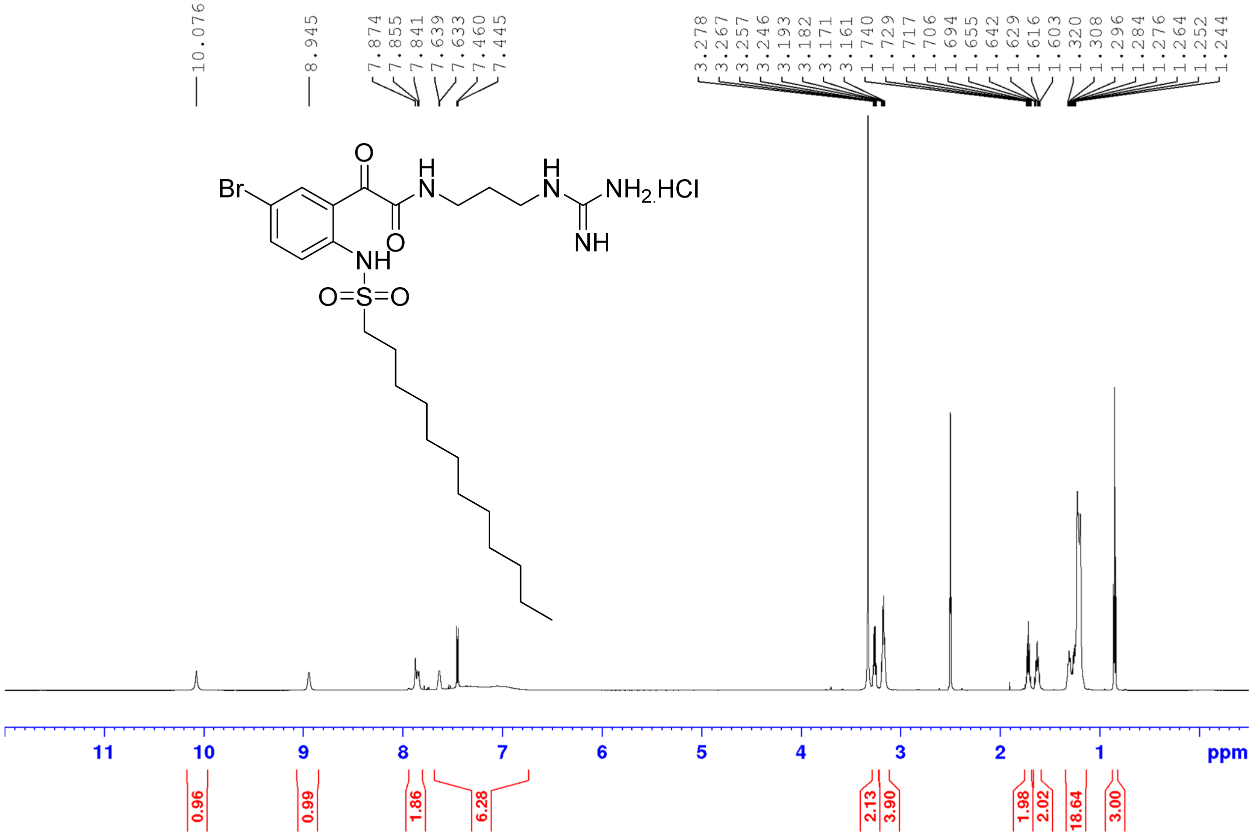

^{13}C NMR (150 MHz, DMSO-*d*$_6$):

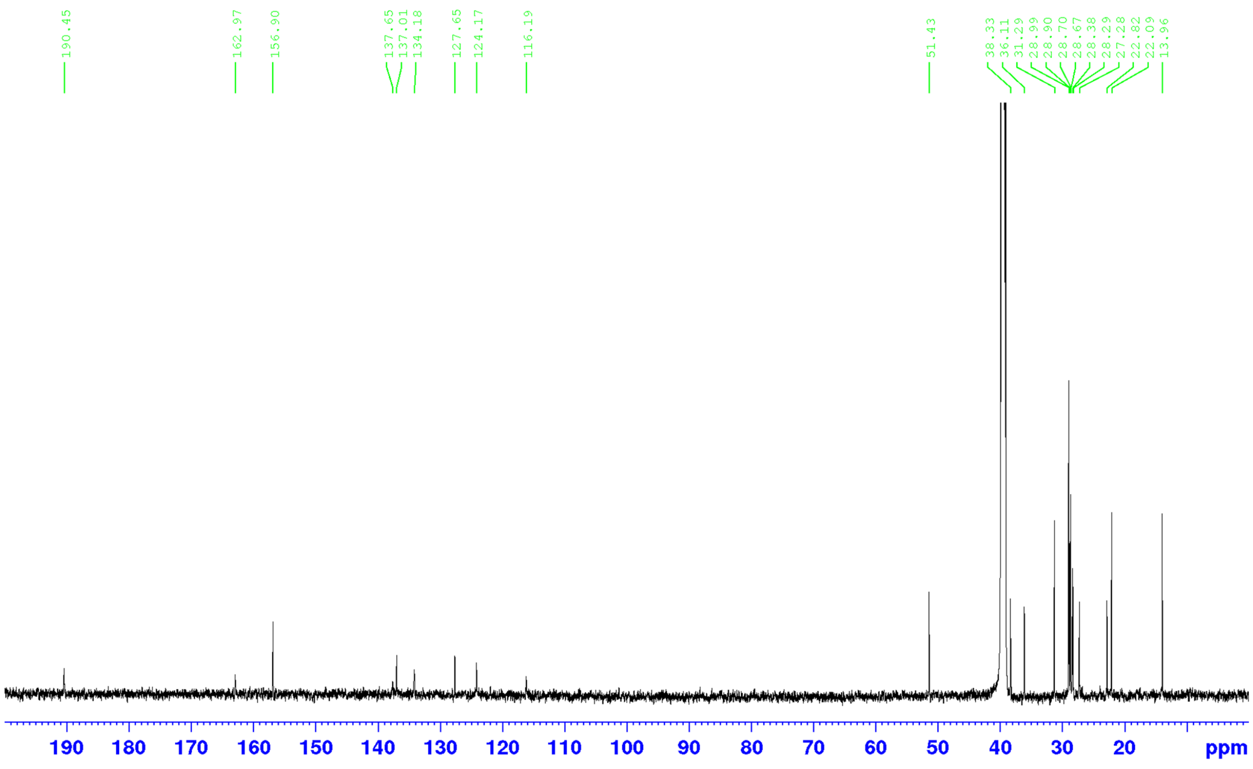

2-(4-(Dodecylsulfonamido)-[1,1'-biphenyl]-3-yl)-*N*-(3-guanidinopropyl)-2-oxoacetamide hydrochloride (**34c**)

^{1}H NMR (600 MHz, DMSO-d_6):

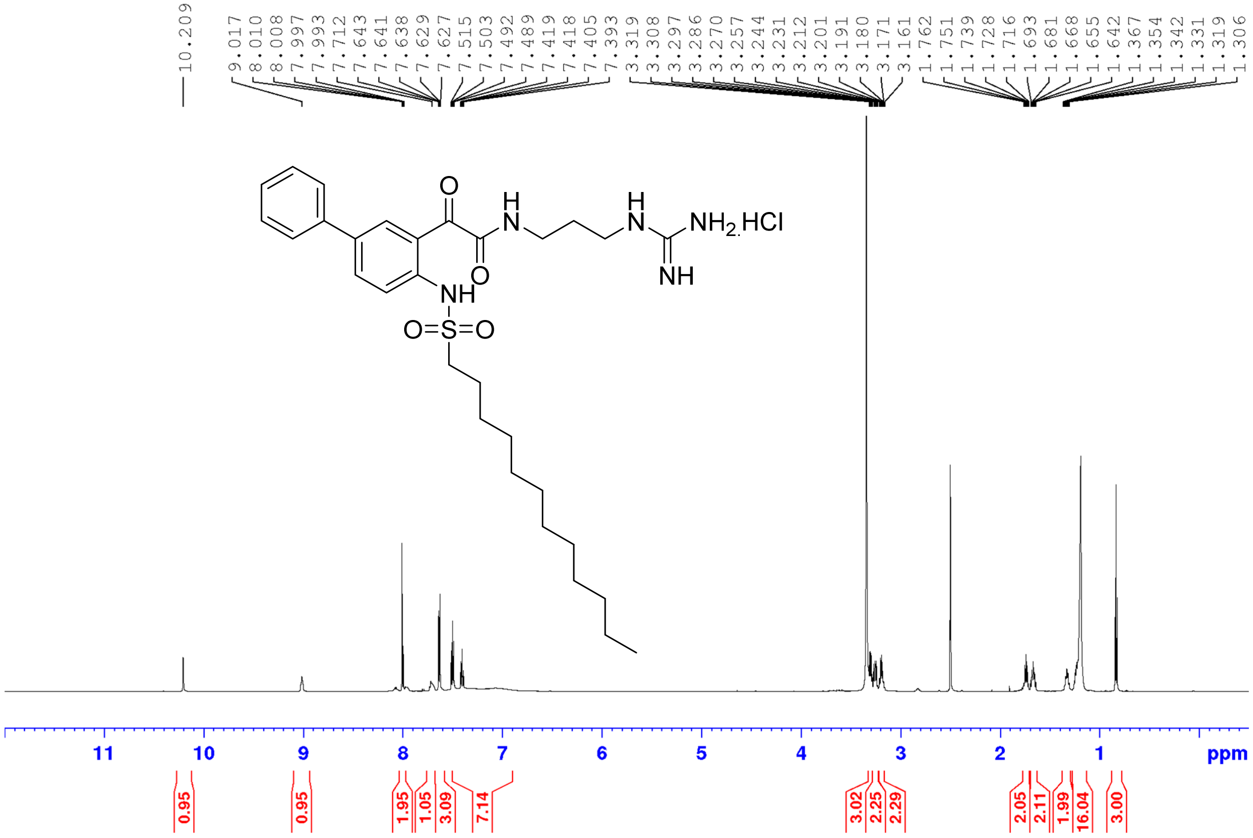

^{13}C NMR (150 MHz, DMSO-d_6):

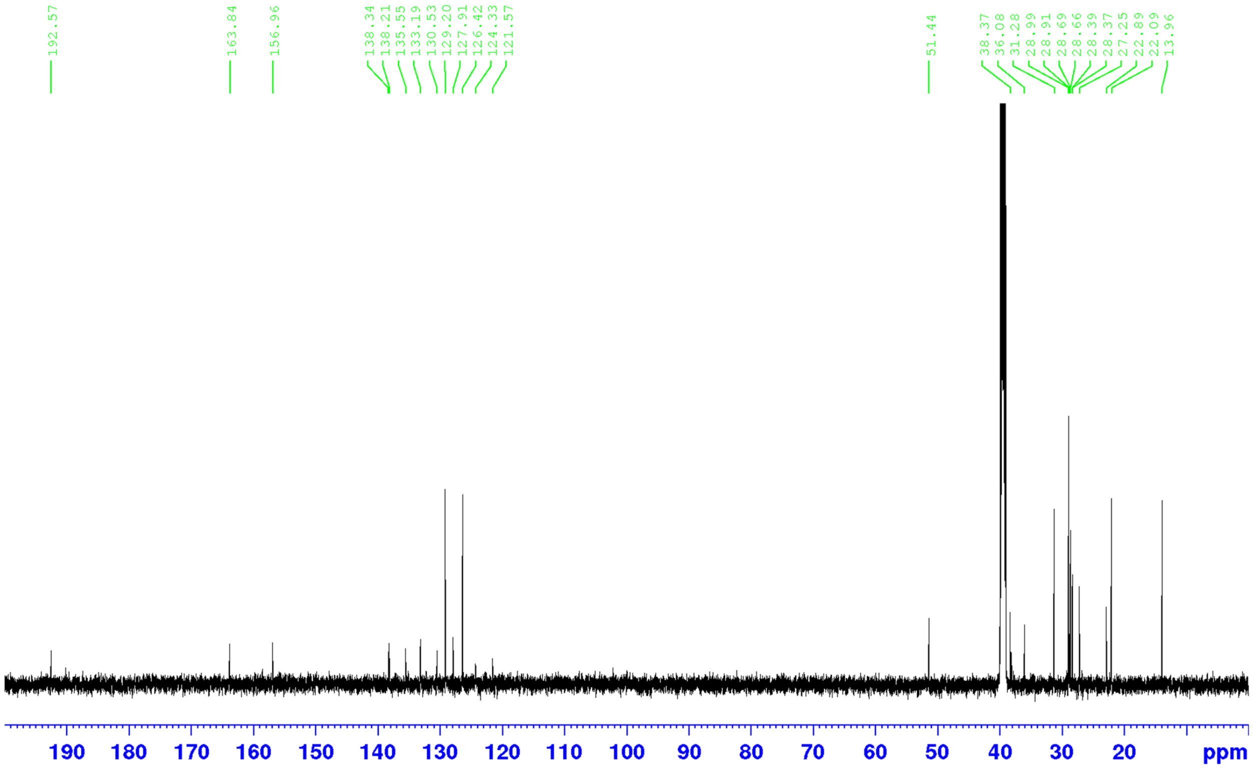

N-(3-Guanidinopropyl)-2-(2-(hexadecylsulfonamido)phenyl)-2-oxoacetamide hydrochloride (**35a**)

^{1}H NMR (600 MHz, DMSO-d_6):

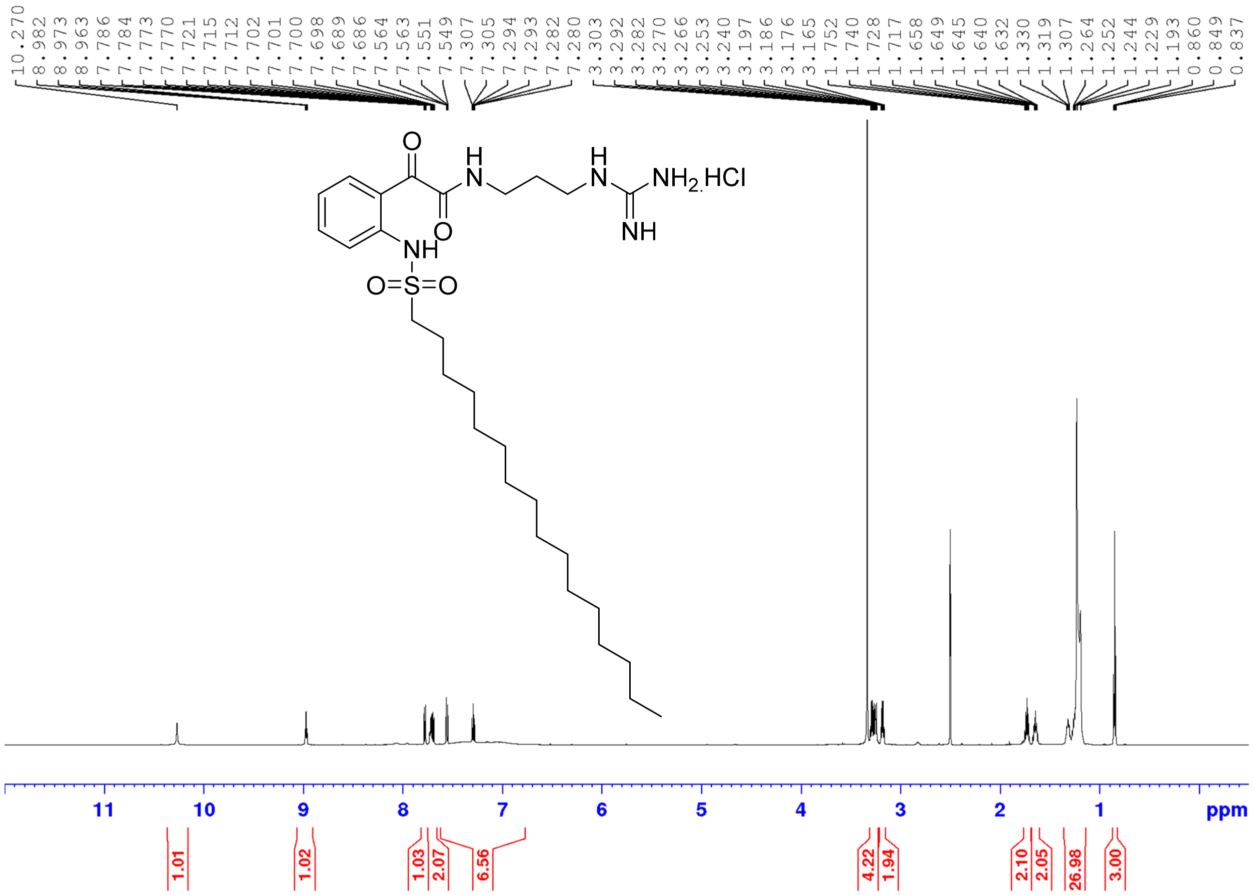

^{13}C NMR (150 MHz, DMSO-d_6):

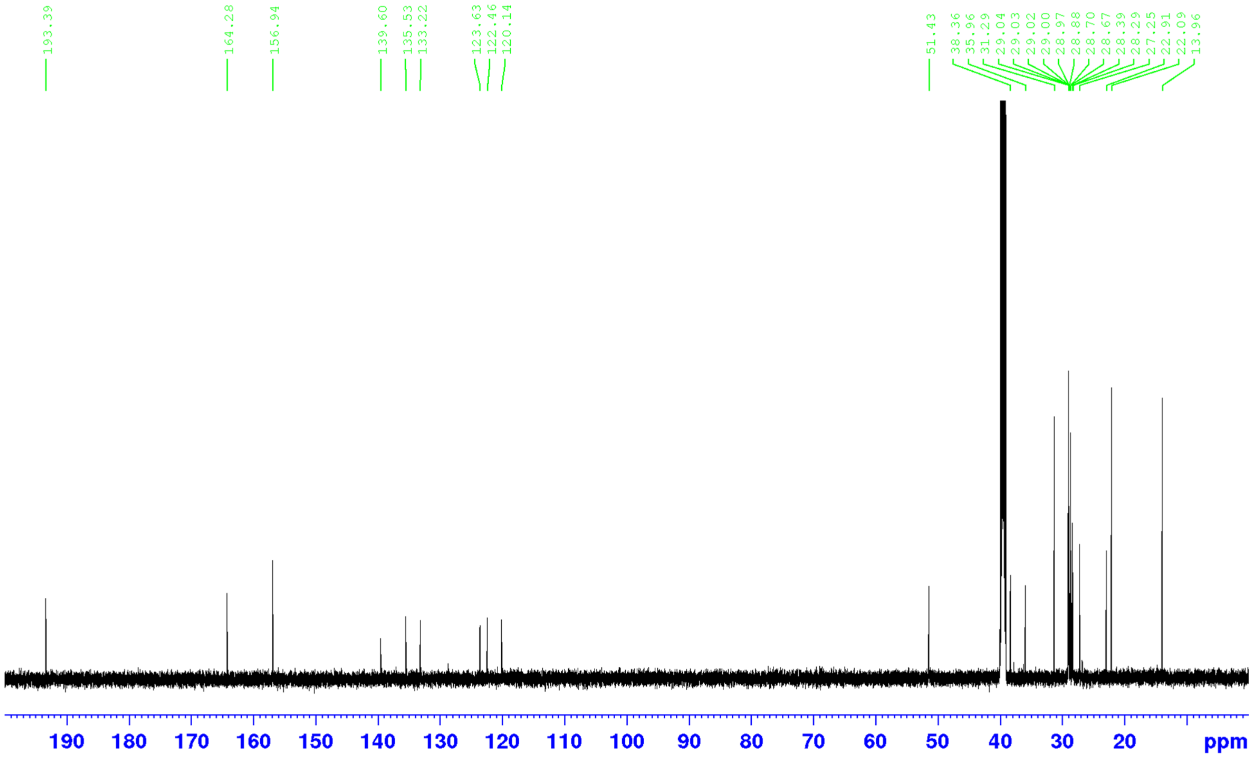

2-(5-Bromo-2-(hexadecylsulfonamido)phenyl)-*N*-(3-guanidinopropyl)-2-oxoacetamide hydrochloride (**35b**)

^{1}H NMR (400 MHz, DMSO-d_6):

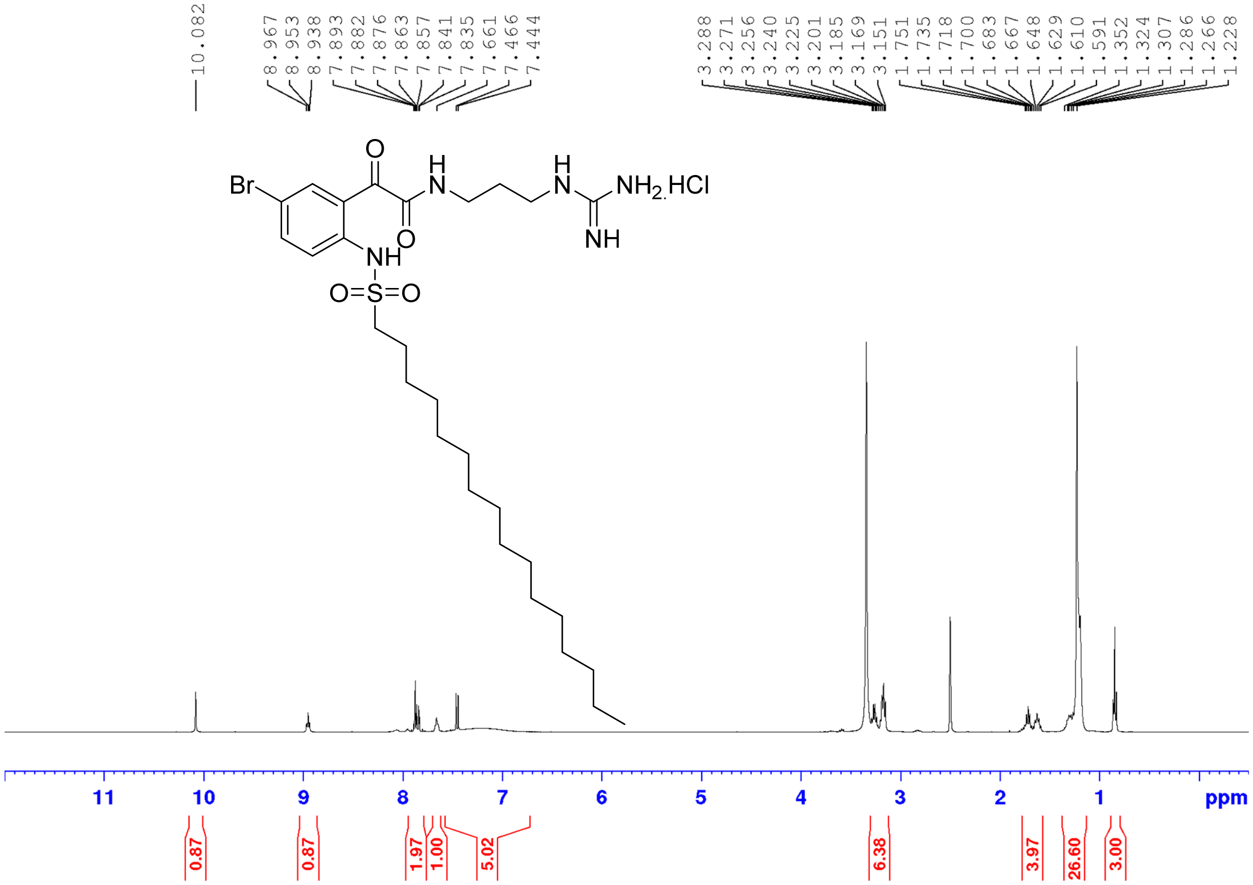

^{13}C NMR (100 MHz, DMSO-d_6):

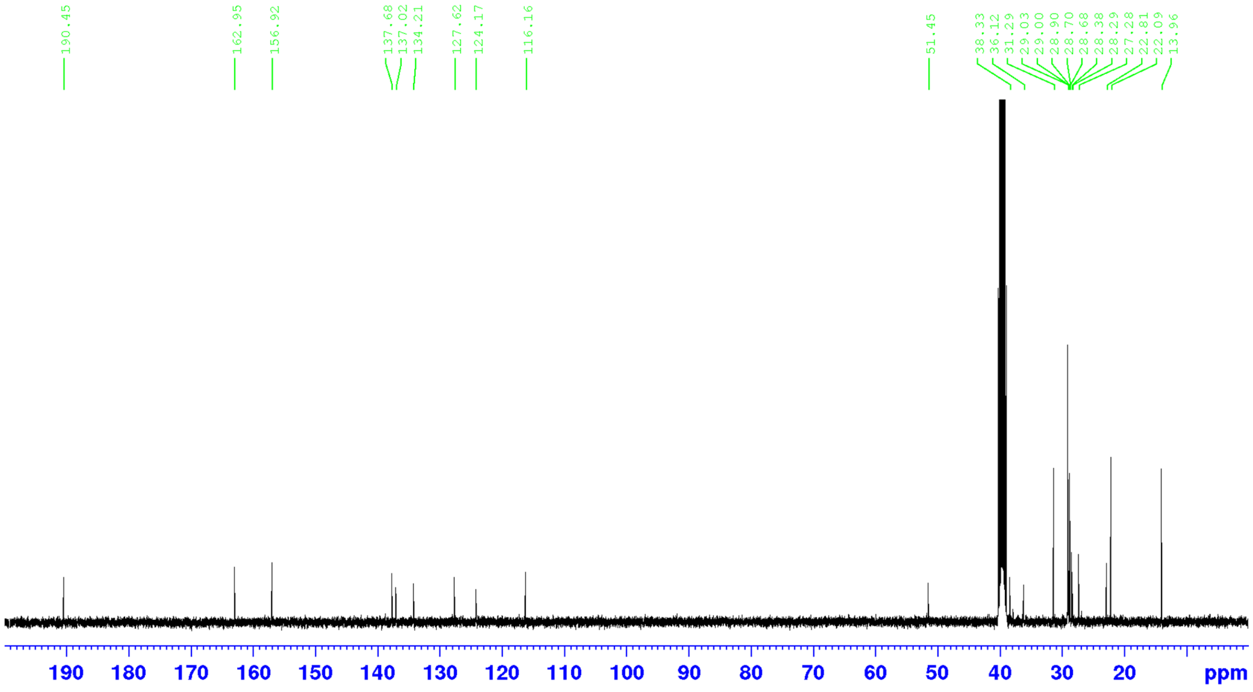

N-(3-Guanidinopropyl)-2-(4-(hexadecylsulfonamido)-[1,1'-biphenyl]-3-yl)-2-oxoacetamide hydrochloride (**35c**)

^{1}H NMR (400 MHz, DMSO-d_6):

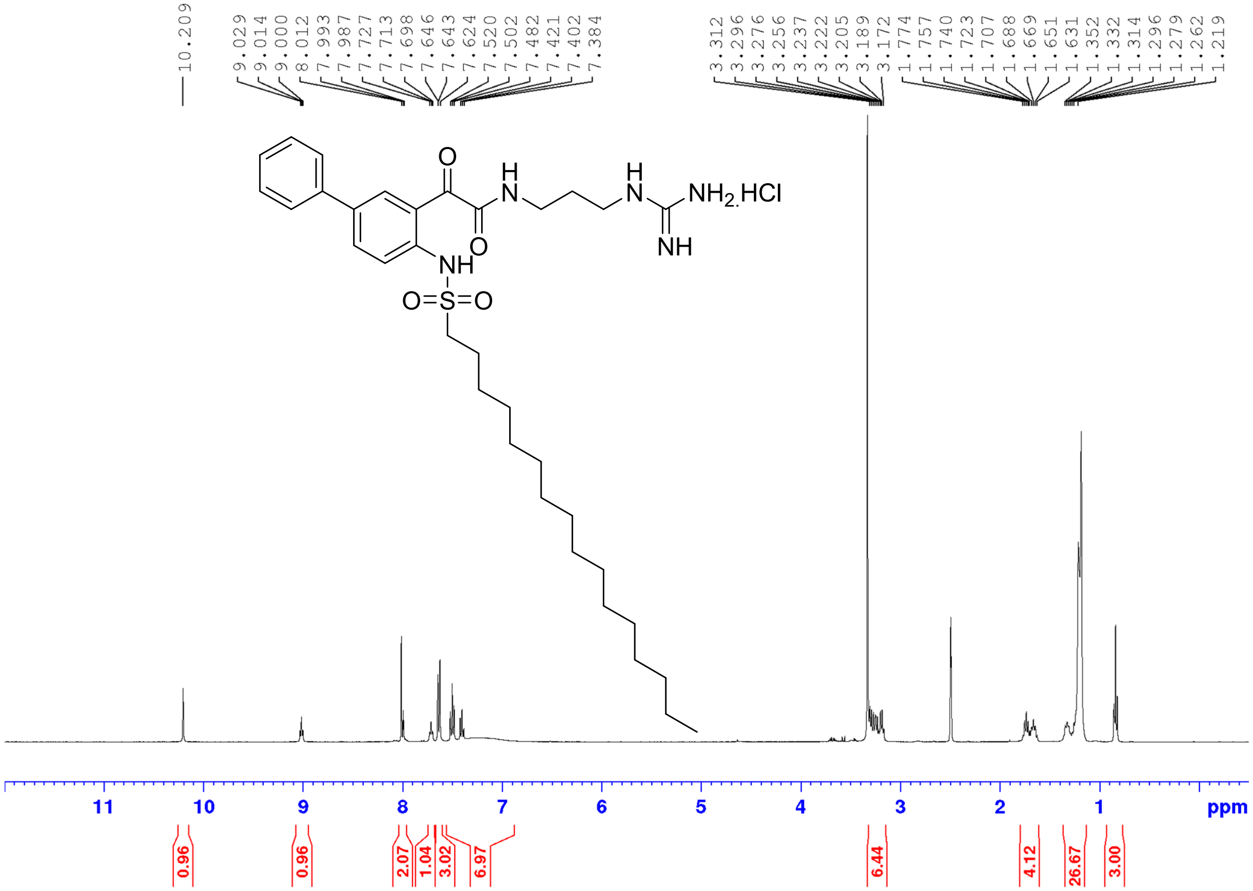

^{13}C NMR (100 MHz, DMSO-d_6):

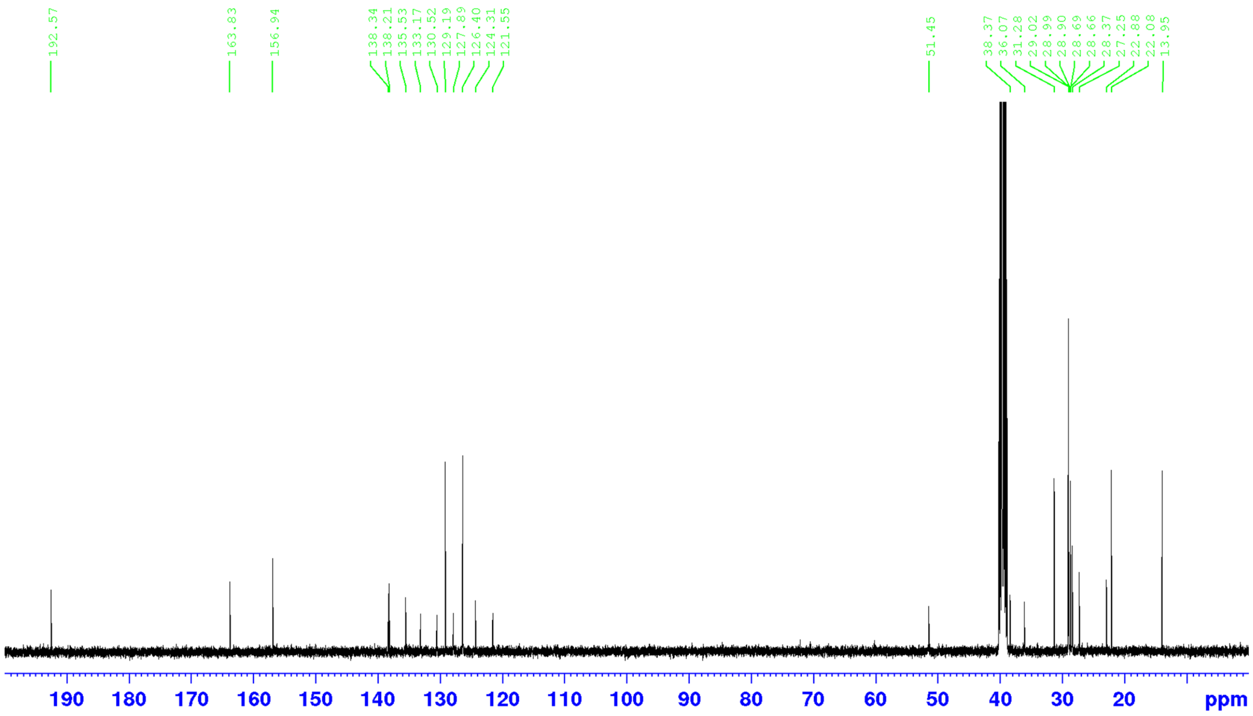

Supplement: Supplementary file 1 [file ijms-22-07344-s001.zip › ijms-1261216-supplementary.pdf]
